# Divergent Synthesis of Chondroitin Sulfate Disaccharides and Identification of Sulfate Motifs that Inhibit Triple Negative Breast Cancer

**DOI:** 10.1038/srep14355

**Published:** 2015-09-24

**Authors:** Zhong Wei Poh, Chin Heng Gan, Eric J. Lee, Suxian Guo, George W. Yip, Yulin Lam

**Affiliations:** 1 Department of Chemistry, National University of Singapore (NUS), Singapore; 2 NUS Graduate School for Integrative Sciences and Engineering (NGS), Singapore; 3 Department of Anatomy, National University of Singapore (NUS), Singapore

## Abstract

Glycosaminoglycans (GAGs) regulate many important physiological processes. A pertinent issue to address is whether GAGs encode important functional information via introduction of position specific sulfate groups in the GAG structure. However, procurement of pure, homogenous GAG motifs to probe the “sulfation code” is a challenging task due to isolation difficulty and structural complexity. To this end, we devised a versatile synthetic strategy to obtain all the 16 theoretically possible sulfation patterns in the chondroitin sulfate (CS) repeating unit; these include rare but potentially important sulfated motifs which have not been isolated earlier. Biological evaluation indicated that CS sulfation patterns had differing effects for different breast cancer cell types, and the greatest inhibitory effect was observed for the most aggressive, triple negative breast cancer cell line MDA-MB-231.

Glycosaminoglycans (GAGs) are heterogeneous polysaccharides comprising of repeating uronic acid and amino sugar disaccharide units. These macromolecules can be covalently attached to core proteins to form proteoglycan side chains, or located in the extracellular matrix and intracellular secretory granules^1^,^2^,^3^. GAGs have gained interest as potential therapeutic agents in cancer treatment, with studies showing their involvement in various pathobiological cancer stages^4^,^5^, and interactions with various effective molecules such as growth factors and cytokines^6^,^7^. Overexpression of chondroitin sulfate (CS) has been identified in various cancer phenotypes such as prostate, testicular, gastric, pancreatic and breast cancer^8^,^9^,^10^,^11^,^12^. For instance, compositional analysis of GAG side chains isolated from malignant breast tissues indicate an elevation in CS expression^13^,^14^,^15^, with an increase in CS-A and CS-E sulfation sequences and a decrease in CS-C and CS-D^16^,^17^,^18^. These indicate that the sulfate groups present on CS might play an important role in the cellular processes involved in the progression of breast cancer^7^,^8^,^19^,^20^.

To investigate the molecular interactions of CS, chemical synthesis provides a viable alternative to prepare pure, homogenous CS sequences via careful control on the site(s) of sulfation to probe structural activity relationship. Notable work has been achieved by various groups in the synthesis of different CS analogues, such as CS-A, CS-C, CS-D, CS-E, CS-R, CS-K, CS-L, CS-M^21^,^22^,^23^,^24^,^25^,^26^,^27^,^28^,^29^,^30^,^31^,^32^,^33^,^34^ and some of these analogues have been studied for their biological effects^24^,^35^,^36^. However, based on the current synthetic strategies reported, not all the sulfation patterns possible in the CS repeating unit can be obtained. We opined that CS sulfation motifs which are not commonly expressed could encode important regulatory information. Thus, we envisioned a synthetic strategy which would allow for the synthesis of all the sulfation patterns possible in CS.

In CS, sulfation may occur on the C-2, C-3 positions of D-glucuronic acid and the C-4′, C-6′ positions of D-N-acetyl galactosamine (Fig. 1) thus accounting for a total of 16 disaccharide possibilities. As with other saccharide synthesis, many key protection steps are required to control the site of sulfation in the desired analogue^37^,^38^,^39^. Our synthetic strategy utilizes the benzyl ether and ester protecting groups as orthogonal handles to direct regioselective sulfation in the final product. Prior research work has demonstrated that the C-2 ester directing group is pivotal to direct *β*-stereoselective glycosylation^25^,^34^. Since C-2 may contain a sulfate group, ester protected hydroxyl groups are thus required as both sulfation and non-sulfation sites depending on the target compound. With this in mind, we modified some of the currently available CS precursors^24^,^31^,^35^,^38^,^40^,^41^ to obtain glycosyl donors **D1**–**D4** and acceptors **A1**–**A4**; these building blocks enable the synthesis of all 16 sulfation patterns theoretically possible in the CS repeating unit.

## Results and Discussion

### Synthesis of monomeric building blocks

**Figure 2:**
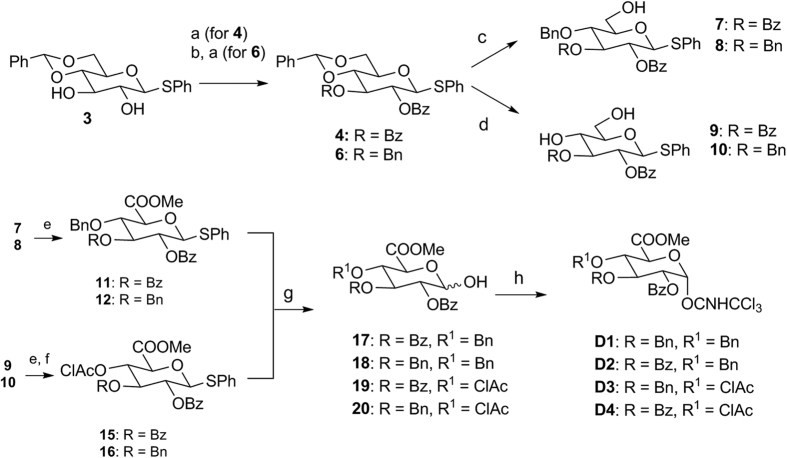
Synthesis of glycosyl donors D1–D4. Reagents and conditions: (**a**) Bz_2_O, DMAP, CH_2_Cl_2_, **4**: 88%; (**b**) Bu_2_SnO, toluene, 90 ^o^C, then BnBr, CsF, DMF, **6**: 57% (3 steps); (**c**) BH_3_.THF, CoCl_2_, **7**: 72%, **8**: 75%; (**d**) TFA, CH_2_Cl_2_/H_2_O, **9**: 84%, **10**: 88% (**e**) TEMPO, BAIB, CH_2_Cl_2_/H_2_O, then MeI, NaHCO_3_, TBAI, DMF, 50 ^o^C, **11**: 48%, **12**: 55%, (**f**) Cl_2_Ac_2_O, pyridine, CH_2_Cl_2_, **15**: 45%, **16**: 48% (3 steps); (**g**) NIS, TFA, CH_2_Cl_2_/H_2_O, **17**: 74%, **18**: 70%, **19**: 77%, **20**: 75%; (**h**) Cl_3_CCN, DBU, CH_2_Cl_2_, **D1**: 68%, **D2**: 75%, **D3**: 70%, **D4**: 78%.

To obtain the glycosyl donors, intermediate **3** (Fig. 2) was subjected to different protection steps in a divergent mode. C-2 ester protection was required for all 4 donors to direct *β*-stereoselective glycosylation, and the C-3 hydroxyl group was protected either as an ester or a benzyl ether via the dibutyl tin oxide mediated approach^42^. For **D1** and **D2**, ester protected hydroxyl groups were denoted as sulfation sites; hence an orthogonal benzyl ether protecting group was required on C-4, achieved by the use of CoCl_2_ and BH_3_.THF to direct complete regio-reductive ring opening of the benzylidene acetal protecting group^43^,^44^ (intermediates **7** and **8**). Conversely, **D3** and **D4** mark benzyl ether protected hydroxyl groups as sulfation sites and hence require the orthogonal ester protection at C-4; chloroacetyl ester was chosen as this group could be selectively cleaved to allow for the synthesis of longer CS fragments when required^45^,^46^. Intermediates **7**, **8**, **9**, **10** were subjected to C-6 oxidation and carboxylate methylation, followed by C-4 chloroacetylation (for intermediates **15** and **16**). This was followed by anomeric thiophenol deprotection and attachment of the trichloroacetimidate glycosyl auxiliary to furnish donors **D1–D4**.

**Figure 3:**
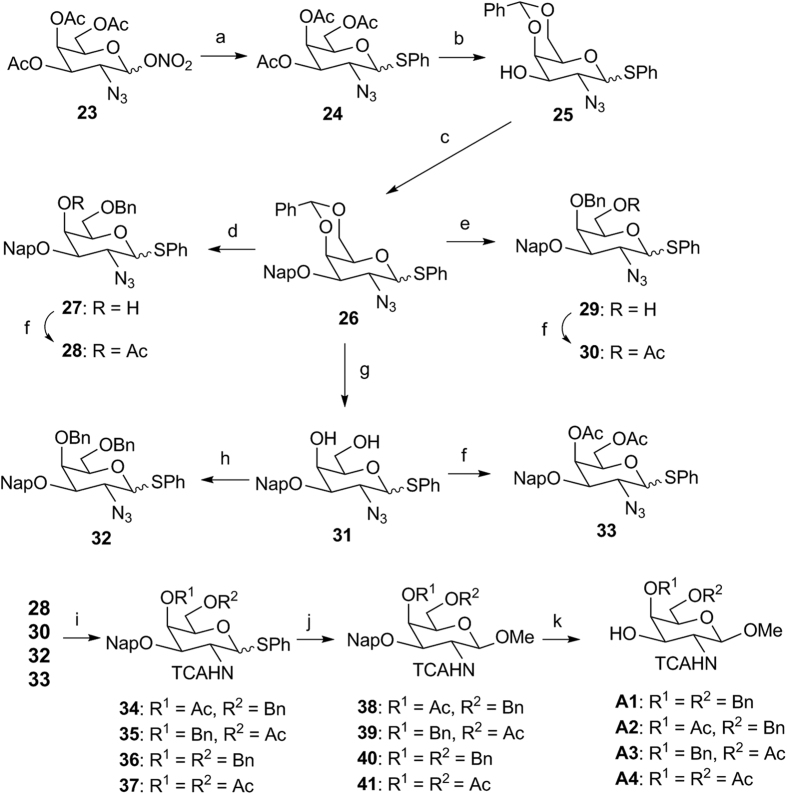
Synthesis of glycosyl acceptors A1–A4. Reagents and conditions: (**a**) NaOAc, AcOH, 80 ^o^C, then PhSH, BF_3_.OEt_2_, CH_2_Cl_2_, 40 ^o^C, 78%; (**b**) NaOMe, MeOH, then PhCH(OMe)_2_, CSA, MeCN, 60 °C, 74%; (**c**) NapBr, NaH, DMF, 81%; (d) TES, TfOH, CH_2_Cl_2_, −78 ^o^C, 78%; (**e**) TES, PhBCl_2_, CH_2_Cl_2_, −78 ^o^C, 68%; (**f**) Ac_2_O, DMAP, CH_2_Cl_2_, **28**: 88%, **30**: 89%, **33**: 85%; (**g**) 80% AcOH, 80 °C, 90%; (**h**) BnBr, NaH, DMF, 75%; (**i**) Zn, NH_4_Cl, EtOH/H_2_O, 80 ^o^C, then Cl_3_COCl, TEA, THF, **34**: 54%, **35**: 58%, **36**: 55%, **37**: 60%; (**j**) NIS/TMSOTf, MeOH, CH_2_Cl_2_, −10 ^o^C, **38**: 75%, **39**: 70%, **40**: 74%, **41**: 68%; (**k**) DDQ, H_2_O, CH_2_Cl_2_, **A1**: 70%, **A2**: 69%, **A3**: 72%, **A4**: 68%.

To obtain the glycosyl acceptors, known intermediate **23**^47^,^48^,^49^ was modified to introduce key protecting groups in common intermediate **26** (Fig. 3). Regio-reductive ring opening of the benzylidene acetal in **26** enabled the formation of the benzyl ether on either the C-4′ position or C-6′ position depending on the choice of Lewis acid used. Ring opening using triethylsilane/TfOH system enabled the formation of the benzyl ether on the C-6′ position in complete regioselectivity^50^ (intermediate **27**), ascertained by 2D NMR. Alternatively, the benzyl ether could be obtained on the C-4′ position with complete regioselectivity via triethylsilane/PhBCl_2_ reductive system^50^ (intermediate **29**). Protection of the corresponding hydroxyl groups as esters formed intermediates **28** and **30**. The benzylidene acetal could also be cleaved via acidic hydrolysis, with both hydroxyl groups protected as benzyl ethers or esters (**32** and **33**). The C-2′ azide in intermediates **28**, **30**, **32** and **33** were next converted to the *N*-trichloroacetyl group (TCAHN), this C-2 participating group directed *β*-stereoselective glycosylation of the methyl ether at the anomeric position in the subsequent step. Finally, cleavage of the C-3′ naphthyl ether via DDQ oxidation furnished glycosyl acceptors **A1**–**A4**.

### Glycosylation of monomeric building blocks

**Figure 4:**
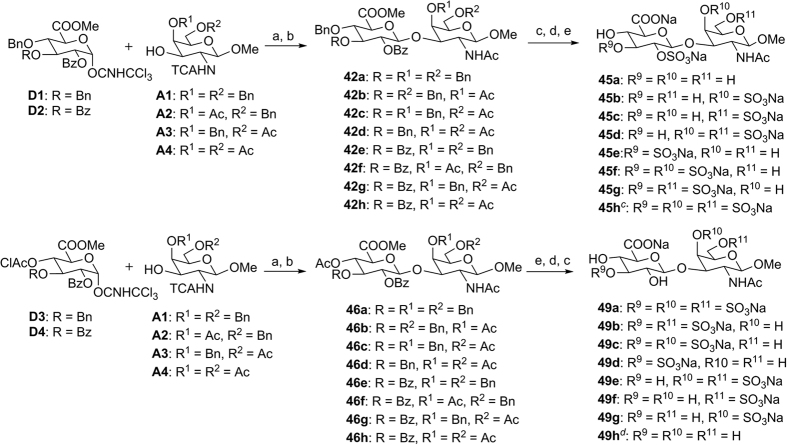
Synthesis of CS disaccharide analogues 45a–45 h and 49a–49 h. Reagents and conditions: (**a**) TMSOTf, 4 Å MS, CH_2_Cl_2_, −10 °C, 64–75%^*b*^; (**b**) Bu_3_SnH, ABCN, toluene, 100 °C, 69–80%^*b*^; (**c**) LiOH, H_2_O_2_, THF/H_2_O, −5 °C to r.t., then NaOH, MeOH, 0 °C to r.t., 72–79%^*b*^; (**d**) SO_3_.TEA, DMF, 50 °C, 68–77%^*b*^; (**e**) H_2_ gas, Pd/C, CH_2_Cl_2_/MeOH/H_2_O, 85–95%^*b*^; ^*b*^For detailed product yields refer to Supplementary Information; ^*c*^**45h**: additional step (**f**): BnBr, NaHCO_3_, DMF, 50 °C, after step (**c**); ^*d*^**49h**: only step (**c**) was required to convert **46h** to **49h**.

With glycosyl donors **D1**–**D4** and glycosyl acceptors **A1**–**A4** on hand, any sulfation pattern required in the final CS disaccharide can be obtained by the judicious choice of donor and acceptor building blocks. **D1**–**D4** were first glycosylated with **A1**–**A4** using TMSOTf catalyst to form the protected disaccharides (Fig. 4). The C-2 participating ester group present in **D1**–**D4** enabled exclusive formation of the *β*-product^25^. Upon glycosylation, the trichloroacetyl group was reduced to the acetyl group by radical mediated tributylstannane reduction. Any C-4 chloroacetyl groups present were also reduced to the form acetyl esters (intermediates **46a**–**46h**).

For intermediates **42a**–**42h**, the ester protecting groups were next liberated via basic hydrolysis and the free hydroxyl groups reacted with the sulfating agent. SO_3_.TEA was utilised to enable complete sulfation of the C-2 hydroxyl group in the glucuronic acid moiety; 5 equiv. sulfating agent was required per –OH to ensure complete sulfation of the desired sites. Fortunately, the C-6 carboxylate group generated from the ester deprotection step did not affect the sulfation step. With the sulfate groups attached at the required positions, global deprotection by hydrogenation of the remaining benzyl ether groups proceeded in the final step to furnish 8 distinct CS disaccharides **45a**–**45h**. Due to the high negative charge in tetrasulfated disaccharide **45h**, an additional step was introduced to protect the C-6 carboxylate group as a benzyl ester, which facilitated product isolation during sulfation.

For intermediates **46a**–**46h**, the benzyl ethers were first cleaved via hydrogenation and the free hydroxyl groups were reacted with SO_3_.TEA. Subsequently, global deprotection of the remaining ester protecting groups via basic hydrolysis^51^ furnished another 8 CS disaccharides **49a**–**49h.** Through this strategy, all 16 CS disaccharides were synthesized, which include those already reported^21^,^30^,^52^,^53^,^54^,^55^, in addition to novel sulfation motifs. By the incorporation of orthogonal protecting groups in the monomeric building blocks, we were able to direct site specific sulfation of the CS disaccharide to obtain all the possible isomers, which were characterised by NMR and high resolution mass spectrometry (ESI) techniques. The complete CS disaccharide library thus enables us to probe the “sulfation code” of CS in biological systems via structural activity relationship studies.

### Evaluating the CS disaccharide library on breast cancer cell viability

To achieve this, we proceeded to test the effect of CS sulfation patterns on breast cancer cell viability. The synthesized CS disaccharides were tested on 4 different human breast cell lines. This included the non-tumorigenic breast epithelial cell line MCF-12A, to evaluate compound cytotoxicity, and 3 breast cancer cell lines: MCF-7, T47D and MDA-MB-231. MCF-7 and T47D are low grade breast cancer cells which express the estrogen receptor and hence can be targeted using hormonal therapy^56^,^57^,^58^,^59^. MDA-MB-231 cells are high grade triple negative breast cancer cells (TNBC) which do not express the estrogen receptor, progesterone receptor nor the human epidermal growth factor receptor 2^60^,^61^. TNBC tumor subtypes show low response to chemotherapy and are more challenging to treat due to the lack of known therapeutic targets, thus resulting in higher patient mortality^62^,^63^,^64^.

The biological effect of each CS disaccharide was investigated by incubating the cells with the CS disaccharide for 72 hours, prior to addition of the MTS reagent to determine number of viable cells after treatment period. 4 different CS disaccharide concentrations were tested (0.1 μg/mL, 1 μg/mL, 10 μg/mL and 100 μg/mL). We first screened the 16 CS disaccharides on MCF-12A cells, and the results indicated that there was no significant change in cell viability (Supplementary Fig. 1). Hence these 16 CS disaccharides were not cytotoxic to normal breast cells.

**Figure 5:**
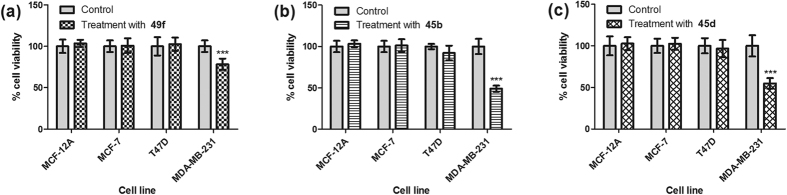
Cell viability assay results for MCF-12A, MCF-7, T47D and MDA-MB-231 cells. % cell viability after 72 hours treatment with 100 μg/mL CS disaccharide (**a**) **49f**, (**b**) **45b**, (**c**) **45d**; Data represents the mean ± SD (n = 6) with reference to non-treatment group (control), analyzed using one-way ANOVA with post-hoc Dunnett’s test, ***p < 0.01.

Interestingly, when the 16 CS disaccharides were tested on the more aggressive MDA-MB-231 cell line, a statistically significant decrease in cell viability was observed (via one way ANOVA analysis) for CS disaccharides **49f**, **45b** and **45d** at 100 μg/mL concentration (Fig. 5; Supplementary Fig. 2 and 3). These inhibitory effects suggest that the sulfate groups present on CS could encode important regulatory information for cellular processes involved in breast cancer survival. The results from the preliminary CS disaccharide screening also indicate that both the number and position of the sulfate groups present in the CS disaccharide have an effect on MDA-MB-231 cell viability. The non-sulfated and fully sulfated CS disaccharides, **49h** and **45h**, have no effect on cell viability suggesting that the presence of some sulfate groups are required for CS to elicit an inhibitory effect on MDA-MB-231 cells but saturating all the possible sulfation sites would lead to a loss of activity.

We next proceeded to screen the CS disaccharides on low grade breast cancer cells MCF-7 and T47D. The MTS results showed no change in the number of viable cells after treatment with the CS disaccharides, indicating that all 16 CS disaccharides had no significant effect on MCF-7 cells (Supplementary Fig. 4). The same observation was noted in T47D cells (Supplementary Fig. 5).

**Figure 6:**
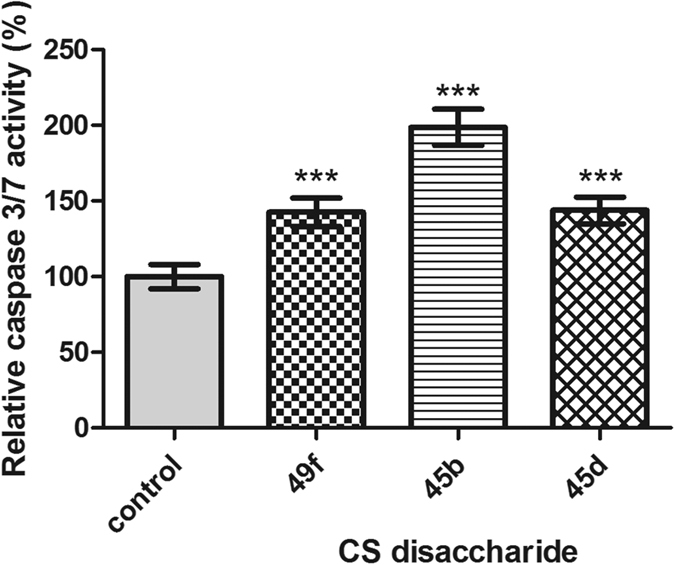
Caspase-Glo 3/7 assay results for MDA-MB-231 cells. Relative caspase 3/7 activity after 72 h treatment with 100 μg/mL CS disaccharide **49f**, **45b** and **45d**. Data represents the mean ± SD (n = 6) with reference to non-treatment group (control), analyzed using one-way ANOVA with post-hoc Dunnett’s test, ***p < 0.001.

To further evaluate the active CS disaccharides (**49f**, **45b** and **45d**), apoptosis assays were subsequently conducted with the Caspase-Glo 3/7 kit which monitored the amount of caspase-3 and -7 activities present in the MDA-MB-231 cells after treatment with the respective CS disaccharides. Results from the caspase assay showed an increase in luminescence when MDA-MB-231 cells were treated with CS disaccharides **49f**, **45b** and **45d** (Fig. 6), indicating an increase in caspase-3 and -7 activities. This suggests that the CS disaccharides could induce death of breast cancer cells via apoptosis. Interestingly, the largest decrease in cancer cell viability and highest amount of caspase activity were seen in the CS disaccharide **45b**–treated group.

## Conclusion

In summary, a versatile synthetic strategy has been devised for the chemical synthesis of all the sulfation patterns possible in the CS repeating unit. A total of 16 different CS disaccharides have been synthesized; these include analogues currently available as well as novel sulfation motifs. Biological evaluation indicated that CS sulfation patterns had differential effects on different types of breast cancer cells. High grade breast tumor cells (MDA-MB-231) showed significant reduction in cell viability upon treatment with CS disaccharides **49f**, **45b** and **45d** while low grade breast tumor cells (MCF-7, T47D) and normal breast cells (MCF-12A) were unaffected. Apoptosis assay suggests that these CS disaccharides could induce apoptosis. Since longer CS sequences could provide stronger activities than the disaccharides^24^,^65^, further studies are presently ongoing to synthesize and evaluate CS oligosaccharides with the active sulfation profiles for their effect on MDA-MD-231 cells.

## Methods

### Chemical synthesis of CS disaccharides

Detailed experimental procedures and compound characterization data can be found in the supplementary information, available in the online version of the paper.

### MTS Assay

The breast cells were plated onto a 96-well plate and cultured for 24 h. After 24 h, the cells were treated with the desired CS disaccharide at 4 different concentrations: 0.1 μg/mL, 1 μg/mL, 10 μg/mL and 100 μg/mL. A control group was included where only the drug vehicle was used; 6 replicates were made for each data set (n = 6). Cells were treated with each compound for 72 hours, and then washed with phosphate-buffered saline (PBS). CellTiter 96® AQueous One Solution (MTS reagent) was added to each well. Absorbance readings (λ = 490 nm) were taken after 3 h, and the data analyzed using one-way Analysis of Variance (ANOVA) with post-hoc Dunnett’s test. Statistical significance was defined as p < 0.05.

### Apoptosis Assay

MDA-MB-231 cells were plated on a 6-well plate and treated with the selected CS disaccharide at 100 μg/mL concentration for 48 h. A control set was included where only the drug vehicle was used. After 48 h, the cells were collected by trypsinization, and reseeded into a white opaque 96-well plate to facilitate luminescence measurement (n = 6). After 24 h, 100 μL of Caspase-Glo® 3/7 reagent was added to each well, and then allowed to incubate for 1 h at room temperature in the dark. Luminescence readings were then measured.

## Additional Information

**How to cite this article**: Poh, Z.W. *et al.* Divergent Synthesis of Chondroitin Sulfate Disaccharides and Identification of Sulfate Motifs that Inhibit Triple Negative Breast Cancer. *Sci. Rep.* **5**, 14355; doi: 10.1038/srep14355 (2015).

## Supplementary Material

Supplementary Information

## Figures and Tables

**Figure 1:**
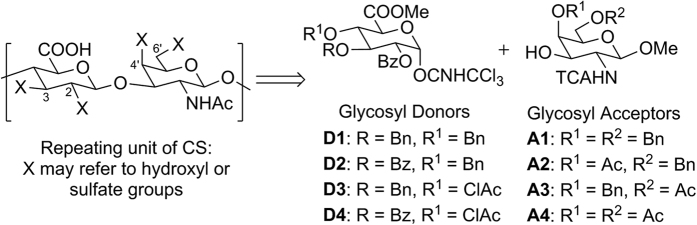
Glycosyl donors and acceptors for CS disaccharide library synthesis.
