# Supplementary material for: Divergent Synthesis of Chondroitin Sulfate Disaccharides and Identification of Sulfate Motifs that Inhibit Triple Negative Breast Cancer
Source: Sci Rep. 2015 Sep 24;5:14355. doi: 10.1038/srep14355 (PMC5155627; doi:10.1038/srep14355)

SUPPLEMENTARY INFORMATION

Divergent Synthesis of Chondroitin Sulfate Disaccharides and Identification of Sulfate Motifs that Inhibit Triple Negative Breast Cancer

Zhong Wei Poh,[†,§] Chin Heng Gan,[†] Eric J. Lee,[†] Suxian Guo,[‡] George W. Yip,[‡,*] and Yulin Lam[†,§,*]

[†] Department of Chemistry, National University of Singapore, 3 Science Drive 3, Singapore 117543

[‡] Department of Anatomy, Yong Loo Lin School of Medicine, National University of Singapore, 4 Medical Drive, Block MD10, Singapore 117594

[§] NUS Graduate School for Integrative Sciences and Engineering (NGS), 28 Medical Drive, Singapore 117456, Singapore

Content

	Page
Cell viability assay results (Supplementary Figures 1-5)	3-5
Synthesis of compounds **2** - **10**	6-8
Synthesis of compounds **11** - **20**	8-9
Synthesis of compounds **D1** – **D4**	9
Synthesis of compounds **22** – **33**	10-12
Synthesis of compounds **34** – **41**	12-13
Synthesis of compounds **A1** – **A4**	13-14
General procedure for the synthesis of protected disaccharide	14
Synthesis of CS disaccharides **45a** – **45g**	14-15
Synthesis of CS disaccharide **45h**	16
Synthesis of CS disaccharides **49a** – **49h**	17-18
Cell viability assay	19
Apoptosis assay	19
Characterization data, ^{1}H and ^{13}C NMR spectra of **2**-**3** and **5**	20-22
2D COSY, 2D HMBC, 2D HSQC of compound **5**	23-25
Characterization data, ^{1}H and ^{13}C NMR spectra of **7**-**14**, **17**-**20** and **D1**-**D4**	26-41
Characterization data, ^{1}H and ^{13}C NMR spectra of **22**-**27**	42-47
2D COSY, 2D HMBC, 2D HSQC of compound **27**	48-50
Characterization data, ^{1}H and ^{13}C NMR spectra of **29**	51
2D COSY, 2D HMBC, 2D HSQC of compound **29**	52-53
Characterization data, ^{1}H and ^{13}C NMR spectra of **31**-**32**, **34**-**41** and **A1**-**A4**	54-67
Characterization data, ^{1}H and ^{13}C NMR spectra of protected disaccharide **D1-A2**	68
Characterization data, ^{1}H and ^{13}C NMR spectra of **42b**-**45b** and protected disaccharide **D1-A3**	69-73
Characterization data, ^{1}H and ^{13}C NMR spectra of **42c**-**45c** and protected disaccharide **D1-A4**	74-78
Characterization data, ^{1}H and ^{13}C NMR spectra of **42d**-**45d** and protected disaccharide **D2-A1**	79-83
Characterization data, ^{1}H and ^{13}C NMR spectra of **42e**-**45e** and protected disaccharide **D2-A2**	84-88
Characterization data, ^{1}H and ^{13}C NMR spectra of **42f**-**45f** and protected disaccharide **D2-A3**	89-93
Characterization data, ^{1}H and ^{13}C NMR spectra of **42g**-**45g** and protected disaccharide **D2-A4**	94-98
Characterization data, ^{1}H and ^{13}C NMR spectra of **42h**-**43h**, **43h'** and **44h**-**45h**	99-103
Characterization data, ^{1}H and ^{13}C NMR spectra of protected disaccharide **D1-A1**	104
Characterization data, ^{1}H and ^{13}C NMR spectra of **42a**-**45a** and protected disaccharide **D3-A1**	105-109
Characterization data, ^{1}H and ^{13}C NMR spectra of **46a**, **48a**-**49a** and protected disaccharide **D3-A2**	110-113
Characterization data, ^{1}H and ^{13}C NMR spectra of **46b**, **48b**-**49b** and protected disaccharide **D3-A3**	114-117
Characterization data, ^{1}H and ^{13}C NMR spectra of **46c**, **48c**-**49c** and protected disaccharide **D3-A4**	118-121
Characterization data, ^{1}H and ^{13}C NMR spectra of **46d**, **48d**-**49d** and protected disaccharide **D4-A1**	122-125
Characterization data, ^{1}H and ^{13}C NMR spectra of **46e**, **48e**-**49e** and protected disaccharide **D4-A2**	126-129
Characterization data, ^{1}H and ^{13}C NMR spectra of **46f**, **48f**-**49f** and protected disaccharide **D4-A3**	130-133
Characterization data, ^{1}H and ^{13}C NMR spectra of **46g**, **48g**-**49g** and protected disaccharide **D4-A4**	134-137
Characterization data, ^{1}H and ^{13}C NMR spectra of **46h** and **49h**	138-139

Cell viability assay results

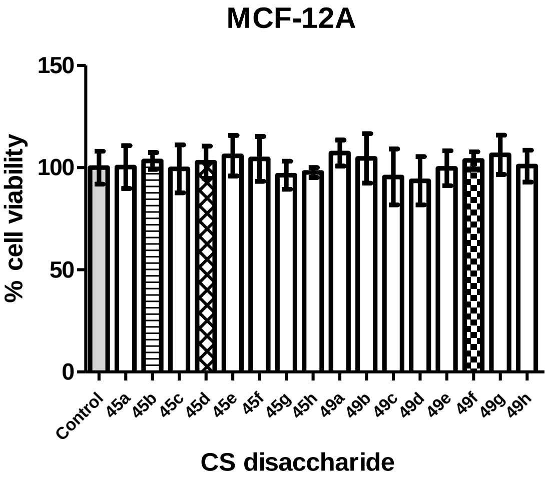

Supplementary Figure 1. Cell viability assay results for MCF-12A cell line after 72h treatment with 100 µg/mL CS disaccharide (all 16 CS disaccharides). Data represents the mean ± SD (n=6) with reference to non-treatment group (control), analyzed using one-way Analysis of Variance (ANOVA) with post-hoc Dunnett's test, ***p < 0.001

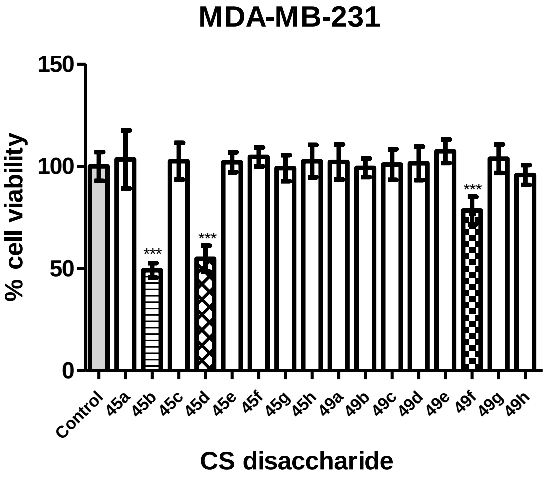

Supplementary Figure 2. Cell viability assay results for MDA-MB-231 cell line after 72h treatment with 100 µg/mL CS disaccharide (all 16 CS disaccharides). Data represents the mean ± SD (n=6) with reference to non-treatment group (control), analyzed using one-way Analysis of Variance (ANOVA) with post-hoc Dunnett's test, ***p < 0.001.

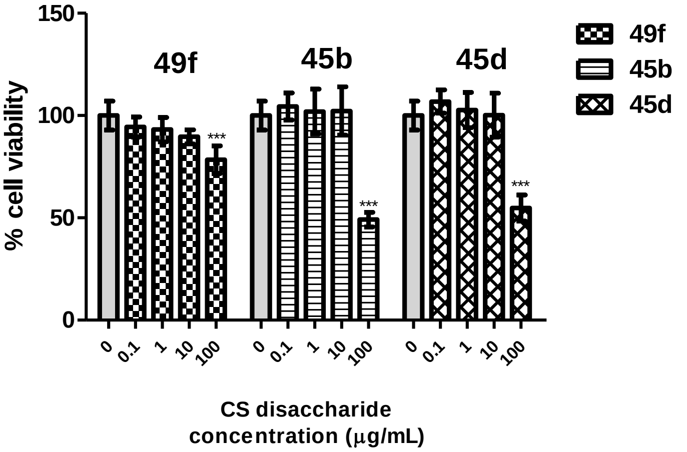

Supplementary Figure 3. Cell viability assay results for MDA-MB-231 cell line after 72h treatment with **49f**, **45b** and **45d** at 0.1µ/mL, 1µ/mL, 10µ/mL and 100µ/mL CS disaccharide concentration. Data represents the mean ± SD (n=6) with reference to non-treatment group (control), analyzed using one-way Analysis of Variance (ANOVA) with post-hoc Dunnett's test, ***p < 0.001.

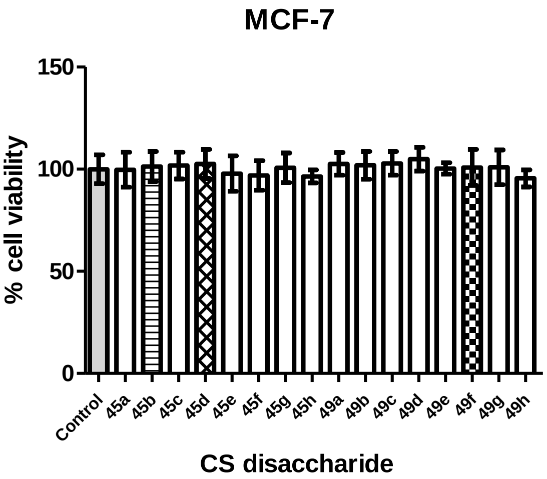

Supplementary Figure 4. Cell viability assay results for MCF-7 cell line after 72h treatment with 100 µg/mL CS disaccharide (all 16 CS disaccharides). Data represents the mean ± SD (n=6) with reference to non-treatment group (control), analyzed using one-way Analysis of Variance (ANOVA) with post-hoc Dunnett's test, ***p < 0.001.

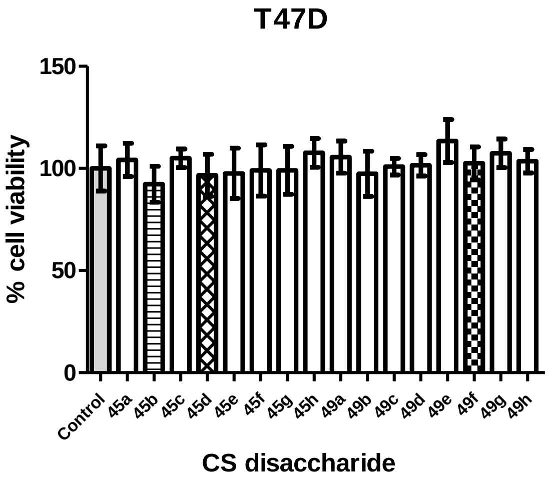

Supplementary Figure 5. Cell viability assay results for T47D cell line after 72h treatment with 100 µg/mL CS disaccharide (all 16 CS disaccharides). Data represents the mean ± SD (n=6) with reference to non-treatment group (control), analyzed using one-way Analysis of Variance (ANOVA) with post-hoc Dunnett's test, ***p < 0.001.

Experimental

General

All commercially available reagents purchased from Sigma Aldrich and Alfa Aesar were used without further purification. Solvents such as hexane, ethyl acetate, dichloromethane, and methanol were pre-distilled prior to usage. Moisture-sensitive reactions were conducted under N_2 environment using commercially obtained anhydrous solvents and activated molecular sieves. Thin layer chromatography (TLC) was performed using Merck silica gel 60 F_{254} pre-coated glass plates and visualised under UV light prior to charring with 10% concentrated sulphuric acid in ethanol. Flash column chromatography was carried out with silica gel (Merck, 230 – 400 mesh); gel filtration chromatography was carried out using Sephadex® G-10 and Sephadex® C-25. ^{1}H and ^{13}C NMR spectrums were recorded on Bruker Avance 500 (DRX500) and Bruker Avance 500 (AV500) at 298K. All *J*-values are reported in Hz and chemical shift (δ) reported in parts per million (ppm) relative to tetramethylsilane (TMS). Mass spectra were determined by high resolution mass spectrometry (HRMS) electrospray ionization (ESI).

Supplementary Figure 6. Synthesis of intermediates 7, 8, 9, 10

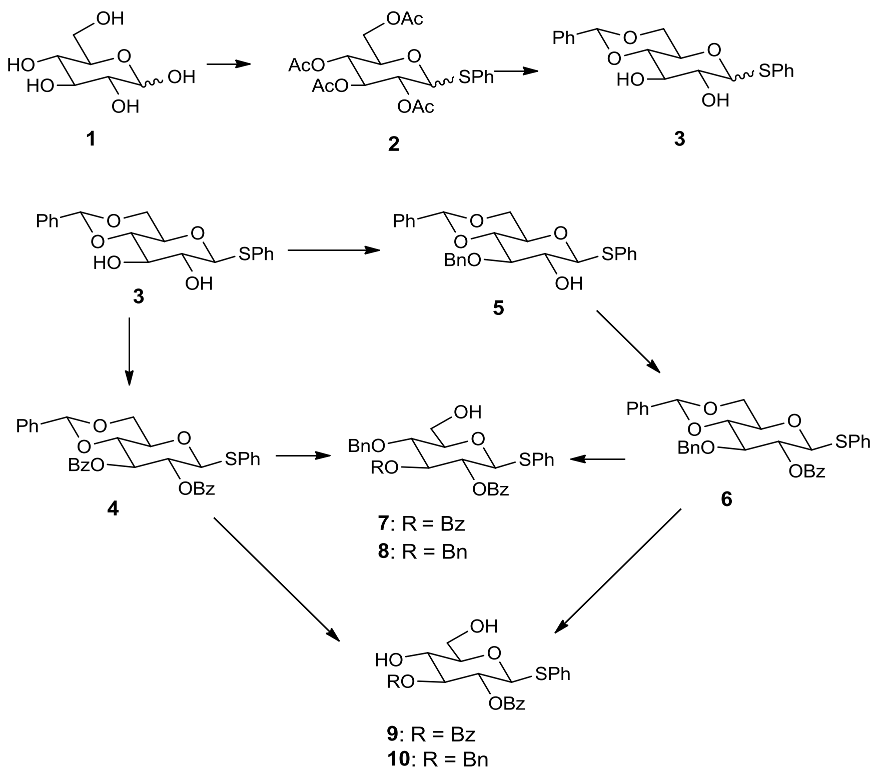

Compound 2: D-(+)-glucose (Compound **1**) (6 g, 33.3 mmol) and DMAP (101.8 mg, 0.83 mmol) were added to pyridine (16.1 mL, 200 mmol) and Ac_2O (18.9 mL, 200 mmol) and stirred at room temperature for 2 h. When TLC analysis (hexane/EtOAc, 1:1 v/v) showed completion of reaction, the reaction mixture was diluted with EtOAc and washed with 5M HCl (3x), followed by saturated $NaHCO_3$ (3x) and brine (1x). The organic layer was dried over Na_2SO_4, filtered and concentrated to a white solid, which was redissolved in CH_2Cl_2 (80 mL), followed by the addition of $BF_3{\cdot}OEt_2$ (12.3 mL, 100 mmol) and PhSH (10.3 mL, 100 mmol). The reaction mixture was

stirred for 16 h at 35°C. When TLC analysis (hexane/EtOAc, 2:1 v/v) showed completion of the reaction, the reaction mixture was diluted with CH_2Cl_2, washed with $NaHCO_3$ (3x) and brine (1x), dried over Na_2SO_4, filtered, and concentrated. The product was purified by flash column chromatography (hexane/EtOAc, 4:1 towards 3:1 towards 2:1) to afford compound **2** as a white solid (11.88 g, 81%, 2 steps).

Compound 3: To a solution of compound **2** (10.77 g, 24.48 mmol) in methanol (100 mL) and CH_2Cl_2 (10 mL) was added NaOMe (529.4 mg, 9.8 mmol) and the mixture was stirred at room temperature for 1 h. When TLC analysis (hexane/EtOAc, 2:1 v/v) showed completion of reaction, the reaction mixture was quenched with Amberlyst-15 and stirred for another 15 min. When the pH was about 3 – 4, the mixture was filtered and concentrated to form a yellow oil, which was redissolved in MeCN (87.4 mL) and DMF (8.7 mL), followed by the addition of $PhCH(OMe)_2$ (7.4 mL, 48.96 mmol) and camphorsulfonic acid (1.86 g, 9.80 mmol). The reaction mixture was stirred for 16 h at 55°C. When TLC analysis (hexane/EtOAc, 1:1 v/v) showed completion of reaction, the reaction mixture was quenched with TEA (2.8 mL), followed by evaporation of solvent. The product was purified by flash column chromatography (hexane/EtOAc, 1.5:1 towards 1:1 towards 1:2) to afford compound **3** as a white solid (7.1 g, 80%, 2 steps).

Compound 4: To a solution of compound **3** (6.52 g, 18.1 mmol) in CH_2Cl_2 (70 mL) was added Bz_2O (10.2 g, 45.3 mmol), DMAP (5.53 g, 45.3 mmol) and the reaction mixture was stirred for 2 h at room temperature. When TLC analysis (hexane/EtOAc, 6:1 v/v) showed completion of reaction, the reaction mixture was diluted with CH_2Cl_2 and washed with 5M HCl (3x), followed by saturated $NaHCO_3$ (3x) and brine (1x). The organic layer was dried over Na_2SO_4, filtered and concentrated to give compound **4** as a white solid which was immediately used for the next reaction to obtain compound **7** or compound **9**.

Compound 5: To a solution of compound **3** (9.50 g, 26.4 mmol) in toluene (92.4 mL) was added dibutyltin oxide (7.23 g, 29 mmol) and the reaction mixture was stirred for 3 h at 110°C. The solvent was next evaporated off to form a yellow oil which was redissolved in DMF (100 mL), followed by the addition of benzyl bromide (3.77 mL, 31.7 mmol) and cesium fluoride (5.21 g, 34.31 mmol). The reaction mixture was stirred for 16 h at room temperature. After TLC analysis (hexane/EtOAc, 4:1 v/v) indicated completion of the reaction, the mixture was diluted with CH_2Cl_2 and washed with water (3x) and brine (1x), dried over Na_2SO_4, filtered, and concentrated. The product was purified by flash column chromatography (hexane/CH_2Cl_2, 6:1 towards 100% CH_2Cl_2) to afford compound **5** as a white solid (7.72 g, 65%, 2 steps).

Compound 6: Compound **5** (7.72 g, 17.1 mmol) was treated as described for the preparation of compound **4** except that 1.2 equivalents of Bz_2O and DMAP were used. Compound **6** was obtained as a white solid which was immediately used for the next reaction to obtain compound **8** or compound **10**.

Compound 7: Crude compound **4** was dissolved in 1M $BH_3{\cdot}THF$ (54.3mL) and $CoCl_2$ (7.05g, 54.3mmol) was added in 3 portions. The reaction was stirred for 2 h at room temperature. When TLC analysis (hexane/EtOAc, 3:1 v/v) showed completion of reaction, the reaction was diluted with EtOAc and quenched with saturated $NaHCO_3$. The solid precipitates were filtered off, and the organic layer washed with saturated $NaHCO_3$ (3x) and brine (1x). The organic layer was dried over anhydrous Na_2SO_4, filtered and concentrated. The product was purified by flash column chromatography (hexane/EtOAc, 5:1 towards 3:1) to afford compound **7** as a white solid (6.51 g, 63%, 2 steps).

Compound 8: Crude compound **6** was treated as described for the preparation of compound **7** to afford compound **8** as a white solid (6.1 g, 64%, 2 steps).

Compound 9: Crude compound **4** was dissolved in CH_2Cl_2 (100 mL) and TFA (2.75 mL) and water (5 mL) was added. The reaction mixture was stirred for 4 h at room temperature. When TLC analysis (hexane/EtOAc, 1:1 v/v) showed completion of reaction, the reaction mixture was diluted with CH_2Cl_2, washed saturated $NaHCO_3$ (3x) and brine (1x). The organic layer was dried over Na_2SO_4, filtered, and concentrated. The product was purified by flash column chromatography (hexane/EtOAc, 3:1 towards 1:1) to afford compound **9** as a white solid (6.4 g, 74%, 2 steps).

Compound 10: Crude compound **6** was treated as described for the preparation of compound **9** to afford compound **10** as a white solid (5.98 g, 75%, 2 steps).

Supplementary Figure 7. Synthesis of glycosyl donors D1, D2, D3, D4

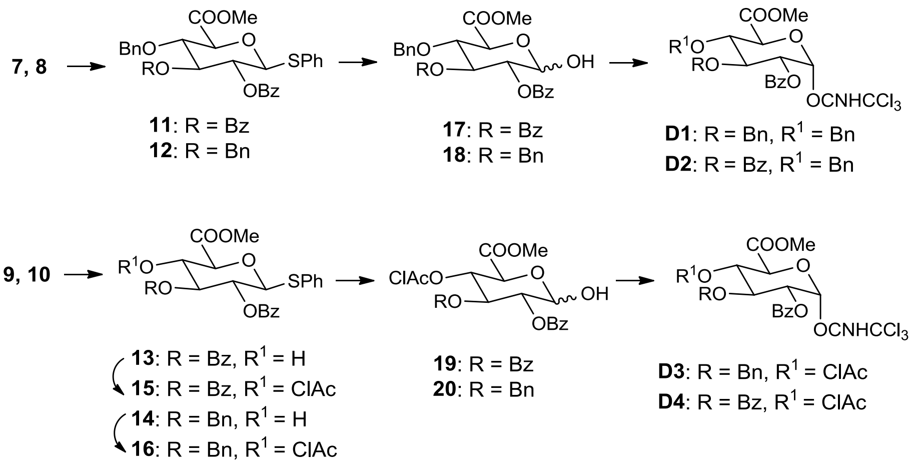

Compound 11: Compound **7** (3.67 g, 6.43 mmol) was dissolved in CH_2Cl_2 (70 mL) followed by the addition of TEMPO (201 mg, 1.29 mmol), BAIB (5.18 g, 16.1 mmol) and H_2O (35 mL), and the reaction mixture was stirred at room temperature for 4 h. When TLC analysis (hexane/EtOAc, 1:1, v/v) showed completion of reaction, the reaction mixture was quenched with saturated $Na_2S_2O_3$, diluted with CH_2Cl_2 and washed with saturated $Na_2S_2O_3$ (3x), brine (1x). The organic layer was dried over Na_2SO_4, filtered and concentrated to form a brown oil, which was redissolved in DMF (40 mL), followed by the addition of MeI (0.6 mL, 9.64 mmol), $NaHCO_3$ (2.7 g, 32.2 mmol) and TBAI (59.4 mg, 0.16 mmol), and stirred at 50°C for 1 h. When TLC analysis (hexane/EtOAc, 3:1 v/v) showed completion of reaction, the reaction mixture was diluted with diethyl ether, washed with water (3x), brine (1x), dried over Na_2SO_4, filtered, and concentrated. The product was purified by flash column chromatography (hexane/EtOAc, 5:1 towards 3:1) to afford compound **11** as a white solid (1.85 g, 48%, 2 steps).

Compound 12: Compound **8** (3.54 g, 6.36 mmol) was treated as described for the preparation of compound **11** to afford compound **12** as a white solid (2.04 g, 55%, 2 steps).

Compound 13: Compound **9** (5.06 g, 10.54 mmol) was treated as described for the preparation of compound **11** to afford compound **13** as a white solid (2.68 g, 50%, 2 steps).

Compound 14: Compound **10** (3.22 g, 6.9 mmol) was treated as described for the preparation of **compound 11** to afford compound **14** as a white solid (1.77 g, 52%, 2 steps).

Compound 15: Compound **13** (2.68 g, 5.27 mmol) was dissolved in CH_2Cl_2 (30 mL) followed by the addition of chloroacetic anhydride (1.8 g, 10.54 mmol) and pyridine (4.25 mL, 52.7 mmol) and the reaction mixture was stirred at room temperature for 1 h. When TLC analysis (hexane/EtOAc, 2:1, v/v) showed completion of reaction, the reaction mixture was diluted with CH_2Cl_2 and washed with 5M HCl (3x), followed by saturated $NaHCO_3$ (3x) and brine (1x). The organic layer was dried over Na_2SO_4, filtered and concentrated to compound **15** as a white solid which was immediately used for the next reaction to obtain compound **19**.

Compound 16: Compound **14** (1.77 g, 3.58 mmol) was treated as described for the preparation of compound **15** to afford compound **16** as a white solid which was immediately used for the next reaction to obtain compound **20**.

Compound 17: Compound **11** (1.85 g, 3.09 mmol) was dissolved in CH_2Cl_2 (30 mL) followed by the addition of NIS (1.04 g, 4.64 mmol) and TFA (473 µL, 6.18 mmol) and H_2O (5 mL). The reaction was stirred at room temperature for 16 h. When TLC analysis (hexane/EtOAc, 2:1, v/v) showed completion of reaction, the reaction mixture was diluted with CH_2Cl_2 and washed with saturated $Na_2S_2O_3$ (3x) and brine (1x). The organic layer was dried over anhydrous Na_2SO_4, filtered and concentrated. The product was purified by flash column chromatography (hexane/EtOAc, 4:1 towards 2:1) to afford compound **17** as a white solid (1.16 g, 74%).

Compound 18: Compound **12** (2.04 g, 3.49 mmol) was treated as described for the preparation of compound **17** to afford compound **18** as a white solid (1.2 g, 70%).

Compound 19: Crude compound **15** was treated as described for the preparation of compound **17** to afford compound **19** as a white solid (1.72 g, 68%, 2 steps).

Compound 20: Crude compound **16** was treated as described for the preparation of compound **17** to afford compound **20** as a white solid (1.22 g, 69%, 2 steps).

Donor D1: Compound **18** (250 mg, 0.51 mmol) was dissolved in CH_2Cl_2 (5 mL) followed by the addition of DBU (30.5 µL, 0.2 mmol) and trichloroacetonitrile (511.4 µL, 5.1 mmol) and the reaction mixture was stirred for 4 hours at room temperature. When TLC analysis (hexane/EtOAc, 4:1, v/v) showed completion of reaction, the solvent was evaporated and the compound was purified by flash column chromatography (hexane/EtOAc, 4:1) to afford donor **D1** as a white foam (220.9 mg, 68%).

Donor D2: Compound **17** (250 mg, 0.494 mmol) was treated as described for the preparation of donor **D1** to afford donor **D2** as a white foam (241.1 mg, 75%).

Donor D3: Compound **20** (250 mg, 0.522 mmol) was treated as described for the preparation of donor **D1** to afford donor **D3** as a white foam (227.7 mg, 70%).

Donor D4: Compound **19** (250 mg, 0.507 mmol) was treated as described for the preparation of donor **D1** to afford donor **D4** as a white foam (252 mg, 78%).

Supplementary Figure 8. Synthesis of intermediates 28, 30, 32, 33

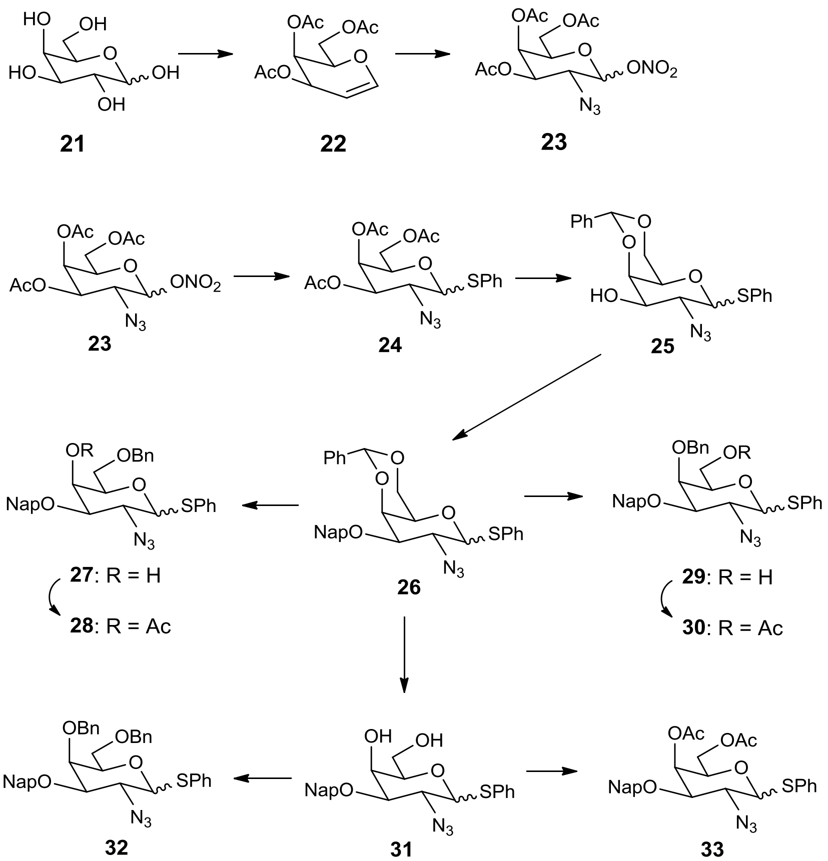

Compound 22: D-(+)-galactose (Compound **21**) (6.00 g, 33.33 mmol) and DMAP (101.80 mg, 0.83 mmol) were added to pyridine (16.1 mL, 200 mmol) and Ac_2O (18.9 mL, 200 mmol) and stirred at room temperature for 2 h. When TLC analysis (hexane/EtOAc, 1:1 v/v) showed completion of reaction, the reaction mixture was diluted with EtOAc and washed with 5M HCl (3x), followed by saturated $NaHCO_3$ (3x) and brine (1x). The organic layer was dried over Na_2SO_4, filtered and concentrated to a white solid, which was redissolved in EtOAc (80 mL), cooled to 0 °C and treated with HBr (41.1 mL). The reaction mixture was allowed to warm to room temperature and stirred for 1 h. When TLC analysis (hexane/EtOAc, 2:1 v/v) showed completion of reaction, the mixture was quenched with saturated $NaHCO_3$, diluted with EtOAc and washed with saturated $NaHCO_3$ (3x) and brine (1x). The organic layer was dried over anhydrous Na_2SO_4, filtered and concentrated. The crude product was redissolved in EtOAc (44 mL), followed by the addition of Zn (11.96 g, 183 mmol), NaOAc (13.04 g, 150 mmol), $CuSO_4$ (259.0 mg, 1.04 mmol), AcOH (31 mL) and H_2O (12.2 mL). The mixture was stirred for 2 h at room temperature. When TLC analysis (hexane/EtOAc, 2:1 v/v) showed completion of reaction, the mixture was filtered, diluted with EtOAc, washed with saturated $NaHCO_3$ (3x) and brine (1x). The organic layer was dried over Na_2SO_4, filtered and concentrated. The product was purified by flash column chromatography (hexane/EtOAc, 3:1 towards 2.5:1) to afford compound **22** as a pale yellow oil (4.99 g, 55%, 3 steps).

Compound 23: A solution of compound **22** (5.60 g, 20.59 mmol) in MeCN (50 mL) was added dropwise to a solution of NaN_3 (2.01 g, 65.05 mmol) and CAN (33.87 g, 61.77 mmol) in MeCN (50 mL) at -25°C. The reaction mixture was allowed to warm to -15°C and stirred for 18 h. When TLC analysis (hexane/EtOAc, 2:1 v/v) showed

completion of reaction, the mixture was diluted with diethyl ether, washed with water (2x) and brine (1x). The organic layer was dried over Na_2SO_4, filtered and concentrated. The product was purified by flash column chromatography (hexane/EtOAc, 4:1 towards 3:1) to afford **23** as a yellow solid (3.49 g, 45%).

Compound 24: Compound **23** (2.76 g, 7.34 mmol) was dissolved in AcOH (20 mL), followed by addition of NaOAc (1.2 g, 1.47 mmol). The mixture was stirred for 1 h at 100°C. When TLC analysis (hexane/EtOAc, 2:1 v/v) showed completion of reaction, the mixture was diluted with EtOAc, washed with saturated $NaHCO_3$ (3x) and brine (1x). The organic layer was dried over Na_2SO_4, filtered and concentrated to form a yellow oil, which was redissolved in CH_2Cl_2 (30 mL), followed by the addition of $BF_3{\cdot}OEt_2$ (2.72 mL, 22 mmol) and PhSH (2.56 mL, 25 mmol). The reaction mixture was stirred for 16 h at 35°C. When TLC analysis (hexane/EtOAc, 3:1 v/v) showed completion of reaction, the mixture was diluted with CH_2Cl_2, washed with $NaHCO_3$ (3x) and brine (1x). The organic layer was dried over Na_2SO_4, filtered, and concentrated. The product was purified by flash column chromatography (hexane/EtOAc, 6:1 towards 3:1) to afford compound **24** as a yellow oil (2.42 g, 78%, 2 steps).

Compound 25: To a solution of compound **24** (5.28 g, 12.28 mmol) in methanol (80 mL) and CH_2Cl_2 (8 mL) was added NaOMe (265.3 mg, 4.91 mmol) and the mixture was stirred at room temperature for 1 h. When TLC analysis (hexane/EtOAc, 2:1 v/v) showed completion of reaction, the reaction mixture was quenched with Amberlyst-15 and stirred for another 15 min. When the pH was about 3 – 4, the mixture was filtered and concentrated to form a pale brown oil, which was redissolved in MeCN (100 mL), followed by the addition of $PhCH(OMe)_2$ (3.69 mL, 24.56 mmol) and camphorsulfonic acid (934 mg, 4.91 mmol). The reaction mixture was stirred for 16 h at 55°C. When TLC analysis (hexane/EtOAc, 3:1 v/v) showed completion of reaction, the mixture was quenched with TEA (1.5 mL), followed by evaporation of solvent. The product was purified by flash column chromatography (hexane/EtOAc, 6:1 towards 3:1 towards 1:1) to afford compound **25** as a yellow oil (3.5 g, 74%, 2 steps).

Compound 26: Compound **25** (2.44 g, 6.32 mmol) and NapBr (1.68 g, 7.59 mmol) were dissolved in DMF (24 ml) before NaH (304 mg, 7.59 mmol) was added in 3 portions to the reaction mixture. Reaction mixture was stirred for 3 h at room temperature. When TLC analysis (hexane/EtOAc, 3:1 v/v) showed completion of reaction, the mixture was diluted in diethyl ether and washed with H_2O (3x) and brine (1x). The organic layer was dried over Na_2SO_4, filtered, and concentrated. The product was purified by flash column chromatography (hexane/CH_2Cl_2, 3:1 towards 1:1 towards 100% CH_2Cl_2) to afford compound **26** as a white solid (2.69 g, 81%).

Compound 27: To a solution of compound **26** (2 g, 3.8 mmol) in CH_2Cl_2 (40 mL) was added 4Å molecular sieves (1 g). The mixture was stirred at room temperature for 30 mins, before cooling to -78°C. Triethylsilane (1.97 mL, 12.35 mmol) and TfOH (962 µL, 10.9 mmol) were then added dropwise into the reaction mixture, and the solution was stirred for another 2 h at -78°C. When TLC analysis (hexane/EtOAc, 3:1 v/v) showed completion of reaction, the mixture was quenched with TEA (2 mL) and MeOH (3 mL), followed by evaporation of solvent. The product was purified by flash column chromatography (hexane/EtOAc, 4:1 towards 3:1) to afford compound **27** as a yellow oil (1.56 g, 78%).

Compound 28: To a solution of compound **27** (3 g, 5.69 mmol) in CH_2Cl_2 (40 mL) was added DMAP (1.04 g, 8.54 mmol) and Ac_2O (643 µL, 6.82 mmol) and reaction mixture was stirred at room temperature for 2 h. When TLC analysis (hexane/EtOAc, 3:1 v/v) showed completion of reaction, the reaction mixture was diluted with CH_2Cl_2

and washed with 5M HCl (3x), followed by saturated $NaHCO_3$ (3x) and brine (1x). The organic layer was dried over Na_2SO_4, filtered and concentrated to form compound **28** as a yellow oil which was immediately used for the next reaction to obtain compound **34**.

Compound 29: Compound **26** (2.6 g, 4.95 mmol) was treated as described for the preparation of compound **27** except that triethylsilane (2.57 mL, 16.1 mmol) and $PhBCl_2$ (1.84 mL, 14.2 mmol) were used, to afford compound **29** as a yellow oil (1.78 g, 68%).

Compound 30: Compound **29** (1.78 g, 3.37 mmol) was treated as described for the preparation of **28** to form compound **30** as a yellow oil which was immediately used for the next reaction to obtain compound **35**.

Compound 31: Compound **26** (1.5 g, 2.86 mmol) was dissolved in AcOH/H_2O (16 mL/3 mL) and stirred at 100°C for 2 h. When TLC analysis (hexane/EtOAc, 1:1 v/v) showed completion of reaction, the mixture was diluted with EtOAc and washed with saturated $NaHCO_3$ (3x) and brine (1x). The organic layer was dried over Na_2SO_4, filtered, and concentrated. The product was purified by flash column chromatography (hexane/EtOAc, 3:1 towards 1:1) to afford compound **31** as a white solid (1.13 g, 90%).

Compound 32: To a solution of compound **31** (2.2 g, 5.02 mmol) in DMF (30 mL) was added BnBr (1.44 mL, 12 mmol) and NaH (480 mg, 12 mmol). The reaction was stirred at room temperature for 3 h. When TLC analysis (hexane/EtOAc, 6:1 v/v) showed completion of reaction, the mixture was diluted with diethyl ether and washed with water (3x) and brine (1x). The organic layer was dried over Na_2SO_4, filtered, and concentrated. The product was purified by flash column chromatography (hexane/EtOAc, 12:1 towards 8:1) to afford compound **32** as a yellow oil (2.33 g, 75%).

Compound 33: Compound **31** (2 g, 4.57 mmol) was treated as described for the preparation of **28** except that 2.4 equivalents of Ac_2O and DMAP were used, to afford compound **33** as a yellow oil which was immediately used for the next reaction to obtain compound **37**.

Supplementary Figure 9. Synthesis of glycosyl acceptors A1, A2, A3, A4

28, 30

32, 33

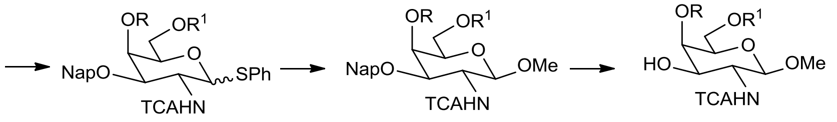

34: R = Ac, R^1 = Bn
35: R = Bn, R^1 = Ac
36: R = R^1 = Bn
37: R = R^1 = Ac

38: R = Ac, R^1 = Bn
39: R = Bn, R^1 = Ac
40: R = R^1 = Bn
41: R = R^1 = Ac

A1: R = R^1 = Bn
A2: R = Ac, R^1 = Bn
A3: R = Bn, R^1 = Ac
A4: R = R^1 = Ac

Compound 34: To a solution of crude compound **28** in EtOH/H_2O (40 mL/13 mL) was added Zn (492 mg, 7.57 mmol) and NH_4Cl (703 mg, 13.26 mmol) and the mixture stirred was at 80°C for 2 h. When TLC analysis (hexane/EtOAc, 6:1 v/v) showed completion of reaction, the reaction was diluted with EtOAc, quenched with diluted saturated $NaHCO_3$ and the solid precipitates were filtered off. The filtrate was washed with saturated $NaHCO_3$ (3x) and brine (1x), and the organic layer was dried over Na_2SO_4, filtered, and concentrated. The yellow oil was redissolved in THF (40 mL) and cooled to 0°C, before TEA (2.5 mL, 17.1 mmol) and CCl_3COCl (1.9 ml, 17.1

mmol) were added. The mixture was stirred for 30 min at 0°C. When TLC analysis (hexane/EtOAc, 4:1 v/v) showed completion of reaction, water was added and THF was evaporated. The compound was extracted using EtOAc, washed with saturated $NaHCO_3$ (3x) and brine (1x), dried over Na_2SO_4, filtered and concentrated. The product was purified by flash column chromatography (hexane/EtOAc, 6:1 towards 4:1) to afford compound **34** as a yellow oil (2.09 g, 48%, 3 steps).

Compound 35: Crude compound **30** was treated as described for the preparation of compound **34** to afford compound **35** as a yellow oil (1.21 g, 52%, 3 steps).

Compound 36: Compound **32** (2.51g, 4.07 mmol) was treated as described for the preparation of compound **34** to afford compound **36** as a yellow oil (1.65 g, 55%, 2 steps).

Compound 37: Crude compound **33** was treated as described for the preparation of compound **34** to afford compound **37** as a yellow oil (1.49 g, 51%, 3 steps).

Compound 38: Compounds **34** (3.8 g, 5.51 mmol) was dissolved in CH_2Cl_2 (70 mL) and cooled to -20°C. NIS (2.48g, 11 mmol) pre-dissolved in 10 mL MeOH was added, followed by TMSOTf (1.99 mL, 11 mmol). The reaction was stirred for 2 h at -10°C. When TLC analysis (hexane/EtOAc, 3:1 v/v) showed completion of reaction, the reaction was quenched with TEA (2.5 mL), diluted with CH_2Cl_2 and washed with saturated $Na_2S_2O_3$ (3x), followed by brine (1x). The organic layer was dried over Na_2SO_4, filtered, and concentrated. The product was purified by flash column chromatography (hexane/EtOAc, 5:1 towards 3:1) to afford compound **38** as a yellow oil (2.52 g, 75%).

Compound 39: Compound **35** (3.28 g, 4.76 mmol) was treated as described for the preparation of compound **38** to afford compound **39** as a yellow oil (2.04 g, 70%).

Compound 40: Compound **36** (2.5 g, 3.4 mmol) was treated as described for the preparation of compound **38** to afford compound **40** as a yellow oil (1.66 g, 74%).

Compound 41: Compound **37** (1.8 g, 2.81 mmol) was treated as described for the preparation of compound **38** to afford compound **41** as a yellow oil (1.07 g, 68%).

Acceptor 1 (A1): To a solution of compound **40** (1.51 g, 2.3 mmol) dissolved in CH_2Cl_2 (50 mL) was added DDQ (626 mg, 2.76 mmol) and 3 mL H_2O, and the reaction was stirred for 14 h at room temperature. When TLC analysis (hexane/EtOAc, 2:1 v/v) showed completion of reaction, the reaction was diluted with CH_2Cl_2 and washed with saturated $NaHCO_3$ (3x) and brine (1x). The organic layer was dried over Na_2SO_4, filtered and concentrated. The product was purified by flash column chromatography (hexane/EtOAc, 4:1 towards 2:1) to afford compound **A1** as a white foam (920 mg, 77%).

Acceptor 2 (A2): Compound **38** (2 g, 3.27 mmol) was treated as described for the preparation of **A1** to afford **A2** as a white foam (1.14 g, 74%).

Acceptor 3 (A3): Compound **39** (1.1 g, 1.8 mmol) was treated as described for the preparation of **A1** to afford **A3** as a white foam (610 mg, 72%).

Acceptor 4 (A4): Compound **41** (1 g, 1.78 mmol) was treated as described for the preparation of **A1** to afford **A4** as a white foam (563 mg, 75%).

Supplementary Figure 10. Synthesis of protected disaccharides

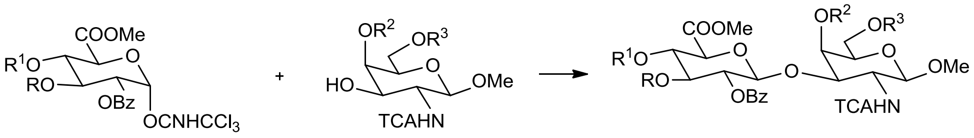

Glycosyl Donor
D1: R = R^1= Bn
D2: R = Bz, R^1= Bn
D3: R = Bn, R^1= ClAc
D4: R = Bz, R^1= ClAc

Glycosyl Acceptor
A1: R^2 = R^3 = Bn
A2: R^2 = Ac, R^3 = Bn
A3: R^2 = Bn, R^3 = Ac
A4: R^2 = R^3 = Ac

Protected dimer
D1-A1 (72%) **D2-A1** (70%)
D1-A2 (68%) **D2-A2** (72%)
D1-A3 (69%) **D2-A3** (74%)
D1-A4 (64%) **D2-A4** (65%)

D3-A1 (75%) **D4-A1** (72%)
D3-A2 (70%) **D4-A2** (69%)
D3-A3 (71%) **D4-A3** (74%)
D3-A4 (65%) **D4-A4** (67%)

General procedure for the synthesis of protected disaccharide: Glycosyl donor (0.385 mmol) and glycosyl acceptor (0.321 mmol) was dissolved in CH_2Cl_2 (8 mL) and 4Å molecular sieves (400 mg) was added. The mixture was stirred at room temperature for 30 min, before cooling to -20°C. TMSOTf (0.064 mmol) was added and the reaction stirred for 2 h at -10°C. When TLC analysis showed completion of reaction, TEA (0.1 mmol) was added and the reaction filtered, followed by evaporation of solvent. The product was purified by flash column chromatography to afford the expected disaccharides (Yields listed in Supplementary Figure 10).

Supplementary Figure 11. Synthesis of CS disaccharides 45a - 45g

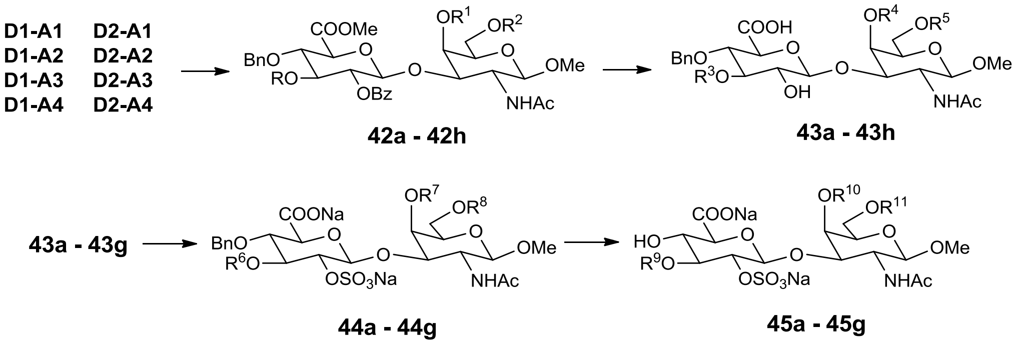

Disaccharide	Product		Product	
**D1-A1**	**42a** (72%)	R = R^1 = R^2 = Bn	**43a** (85%)	R^3 = R^4 = R^5 = Bn
	**44a** (75%)	R^6 = R^7 = R^8 = Bn	**45a** (92%)	R^9 = R^{10} = R^{11} = H
**D1-A2**	**42b** (75%)	R = R^2 = Bn, R^1 = Ac	**43b** (83%)	R^3 = R^5 = Bn, R^4 = H
	**44b** (72%)	R^6 = R^8 = Bn, R^7 = SO_3Na	**45b** (93%)	R^9 = R^{11} = H, R^{10} = SO_3Na
**D1-A3**	**42c** (79%)	R = R^1 = Bn, R^2 = Ac	**43c** (88%)	R^3 = R^4 = Bn, R^5 = H
	**44c** (73%)	R^6 = R^7 = Bn, R^8 = SO_3Na	**45c** (95%)	R^9 = R^{10} = H, R^{11} = SO_3Na

**D1-A4**	**42d** (69%)	R = Bn, $R^1 = R^2$ = Ac	**43d** (86%)	R^3 = Bn, $R^4 = R^5$ = H
	**44d** (68%)	R^6 = Bn, $R^7 = R^8 = SO_3Na$	**45d** (95%)	R^9 = H, $R^{10} = R^{11} = SO_3Na$
**D2-A1**	**42e** (78%)	R = Bz, $R^1 = R^2$ = Bn	**43e** (86%)	R^3 = H, $R^4 = R^5$ = Bn
	**44e** (75%)	$R^6 = SO_3Na$, $R^7 = R^8$ = Bn	**45e** (92%)	$R^9 = SO_3Na$, $R^{10} = R^{11}$ = H
**D2-A2**	**42f** (75%)	R = Bz, R^1 = Ac, R^2 = Bn	**43f** (84%)	R^3 = H, R^4 = H, R^5 = Bn
	**44f** (74%)	$R^6 = R^7 = SO_3Na$, R^8 = Bn	**45f** (94%)	$R^9 = R^{10} = SO_3Na$, R^{11} = H
**D2-A3**	**42g** (77%)	R = Bz, R^1 = Bn, R^2 = Ac	**43g** (88%)	R^3 = H, R^4 = Bn, R^5 = H
	**44g** (77%)	$R^6 = R^8 = SO_3Na$, R^7 = Bn	**45g** (91%)	$R^9 = R^{11} = SO_3Na$, R^{10} = H
**D2-A4**	**42h** (78%)	R = Bz, $R^1 = R^2$ = Ac	**43h** (85%)	$R^3 = R^4 = R^5$ = H

Supplementary Table 1: Structures and yields of key intermediates in the Supplementary Figure 11

General procedure for the synthesis of 42a – 42h: The protected disaccharide (0.25 mmol) was dissolved in toluene (8 mL) and Bu_3SnH (1.5 mmol) and ABCN (109 mg) were added. The reaction was heated to 100°C for 4 h. When TLC analysis showed completion of reaction, the solvent was evaporated off and the product was purified by flash column chromatography to afford **42a** – **42h** (Yields listed in Supplementary Table 1).

General procedure for the synthesis of 43a – 43h: Precursor **42a** – **42h** (0.2 mmol) was dissolved in THF (3.5 mL) and cooled to 0°C, before addition of H_2O_2 (0.4 mL) and LiOH (0.8 mL, 1 M). The reaction was stirred for 16 h at room temperature, after which, methanol (2.26 mL) was added and the reaction was cooled to 0°C before addition of NaOH (0.9 mL). The reaction was stirred at room temperature for 6 h. When TLC analysis showed completion of reaction, the reaction was quenched with Amberlyst-15 and stirred for another 15 min. At pH 3 – 4, the mixture was filtered and solvent was evaporated off. The product was purified by flash column chromatography to afford **43a** – **43h** (Yields listed in Supplementary Table 1).

General procedure for the synthesis of 44a – 44g: Precursor **43a** – **43g** (0.08 mmol) was dissolved in DMF (1 mL) and $SO_3 \cdot$TEA (5 equivalent per –OH present in precursor) was added. The reaction was stirred at 50°C for 36 h. When TLC analysis showed completion of reaction, the reaction was quenched with methanol (0.5 mL) and concentrated. The crude product was first purified using Sephadex LH-20 (CH_2Cl_2/methanol, 1:1) before purification by flash column chromatography. Purified product was next passed through Sephadex C-25 Na^+ resin (methanol/H_2O, 1:1) to afford **44a** – **44g** (Yields listed in Supplementary Table 1).

General procedure for the synthesis of 45a – 45g: Precursor **44a** – **44g** (0.03 mmol) was dissolved in H_2O/MeOH (1 mL/1 mL) and 10% Pd on carbon (100 mg) was added. The reaction was placed under H_2 atmosphere and stirred for 16 h at room temperature. When the reaction was complete, the mixture was filtered through Celite and purified using Sephadex G-10 (100% H_2O). Purified product was passed through Sephadex C-25 Na resin (100% H_2O) to afford **45a** – **45g** (Yields listed in Supplementary Table 1).

Supplementary Figure 12. Synthesis of CS disaccharide 45h

43h →

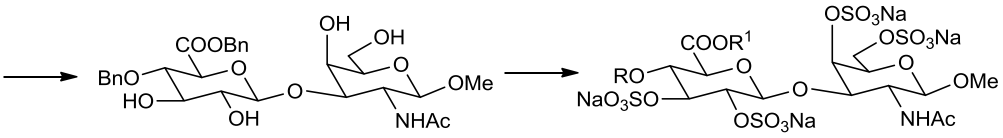

43h'

44h: R = R[1] = Bn

45h: R = H, R[1] = Na

Compound 43h': To a solution of **43h** (37 mg, 0.071 mmol) in DMF (0.5 mL) was added BnBr (12.7μL, 0.107 mmol) and $NaHCO_3$ (30 mg, 0.355 mmol) and the reaction stirred at 50°C for 2 h. When the reaction was complete, the solvent was evaporated off. The product was purified by Sephadex LH-20 (CH_2Cl_2/methanol, 1:1) to afford **43h'** (37.7 mg, 90%).

Compound 44h: Compound **43h'** (37.7 mg, 0.064 mmol) was treated as described for the preparation of **44a** – **44g** with a longer reaction period of 48 h to afford **44h** (43.5 mg, 68%).

Compound 45h: Compound **44h** (43.5 mg, 0.044 mmol) was treated as described for the preparation of **45a** – **45g** to afford **45h** (33 mg, 90%).

Donor / Acceptor	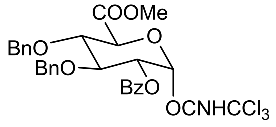 **D1**	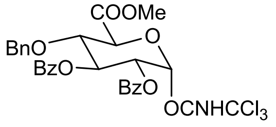 **D2**
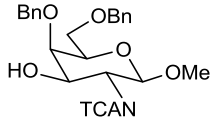 **A1**	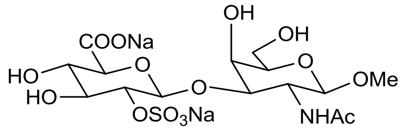 **45a**	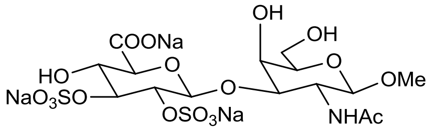 **45e**
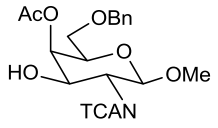 **A2**	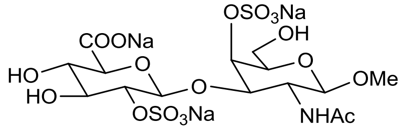 **45b**	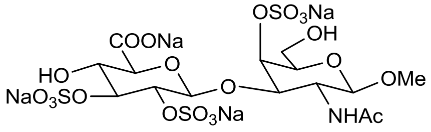 **45f**
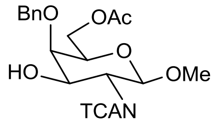 **A3**	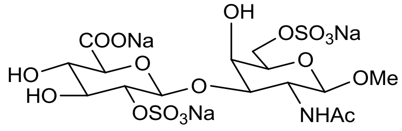 **45c**	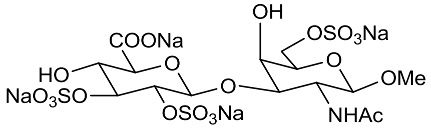 **45g**
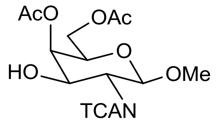 **A4**	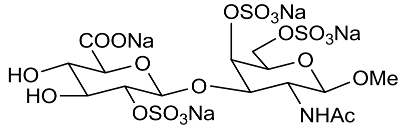 **45d**	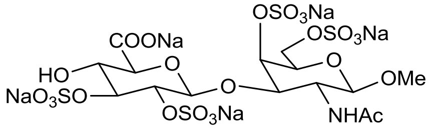 **45h**

Supplementary Table 2: Structures of CS disaccharides **45a – 45h**

Supplementary Figure 13. Synthesis of CS disaccharides 49a - 49h

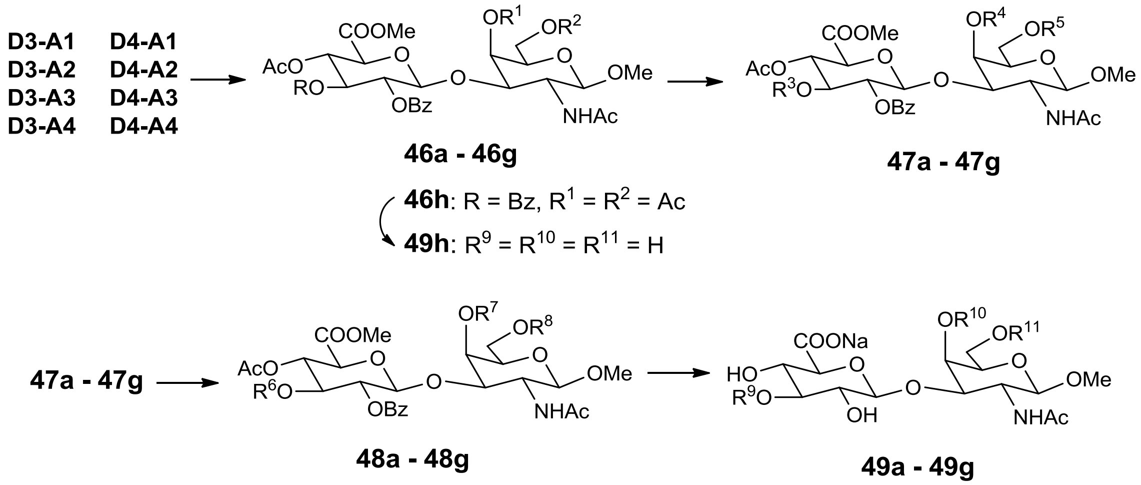

Disaccharide	Product		Product	
**D3-A1**	**46a** (72%)	R = R^1 = R^2 = Bn	**47a**	R^3 = R^4 = R^5 = H
	**48a** (72%)	R^6 = R^7 = R^8 = SO_3Na	**49a** (83%)	R^9 = R^{10} = R^{11} = SO_3Na
**D3-A2**	**46b** (75%)	R = R^2 = Bn, R^1 = Ac	**47b**	R^3 = R^5 = H, R^4 = Ac
	**48b** (74%)	R^6 = R^8 = SO_3Na, R^7 = Ac	**49b** (85%)	R^9 = R^{11} = SO_3Na, R^{10} = H
**D3-A3**	**46c** (72%)	R = R^1 = Bn, R^2 = Ac	**47c**	R^3 = R^4 = H, R^5 = Ac
	**48c** (70%)	R^6 = R^7 = SO_3Na, R^8 = Ac	**49c** (88%)	R^9 = R^{10} = SO_3Na, R^{11} = H
**D3-A4**	**46d** (69%)	R = Bn, R^1 = R^2 = Ac	**47d**	R^3 = H, R^4 = R^5 = Ac
	**48d** (72%)	R^6 = SO_3Na, R^7 =R^8 = Ac	**49d** (90%)	R^9 = SO_3Na, R^{10} = R^{11} = H
**D4-A1**	**46e** (78%)	R = Bz, R^1 = R^2 = Bn	**47e**	R^3 = Bz, R^4 = R^5 = H
	**48e** (75%)	R^6 = Bz, R^7 = R^8 = SO_3Na	**49e** (87%)	R^9 = H, R^{10} = R^{11} = SO_3Na
**D4-A2**	**46f** (77%)	R = Bz, R^1 = Ac, R^2 = Bn	**47f**	R^3 = Bz, R^4 = Ac, R^5 = H
	**48f** (72%)	R^6 = Bz, R^7 = Ac, R^8 = SO_3Na	**49f** (86%)	R^9 = R^{10} = H, R^{11} = SO_3Na
**D4-A3**	**46g** (75%)	R = Bz, R^1 = Bn, R^2 = Ac	**47g**	R^3 = Bz, R^4 = H, R^5 = Ac
	**48g** (71%)	R^6 = Bz, R^7 = SO_3Na, R^8 = Ac	**49g** (88%)	R^9 = R^{11} = H, R^{10} = SO_3Na
**D4-A4**	**46h** (80%)	R = Bz, R^1 = R^2 = Ac	**49h** (92%)	R^9 = R^{10} = R^{11} = H

Supplementary Table 3: Structures and yields of key intermediates in the Supplementary Figure 13

General procedure for the synthesis of 46a – 46h: The protected disaccharide (0.25 mmol) was dissolved in toluene (8 mL) and Bu_3SnH (2 mmol) and ABCN (145 mg) was added. The reaction was heated to 100°C for 4 h. When TLC analysis showed completion of reaction, the solvent was evaporated off and the product was purified by flash column chromatography to afford **46a** – **46h** (Yields listed in Supplementary Table 3).

General procedure for the synthesis of 47a – 47g: Precursor **46a** – **46g** (0.2 mmol) was dissolved in CH_2Cl_2/methanol (2 mL/2 mL) and 10% Pd on carbon (50 mg) was added. The reaction was placed under H_2

atmosphere and stirred for 16 h at room temperature. When TLC analysis showed completion of reaction, the mixture was filtered through Celite and **47a** – **47g** was afforded as a white solid which was immediately used for the next reaction to obtain analogues **48a** – **48g**.

General procedure for the synthesis of 48a – **48g**: Precursor **47a** – **47g** was treated as described for the preparation of **44a** – **44g** to afford **48a** – **48g** respectively. **48f** and **48g** required shorter reaction times of 4 h and 12 h respectively (Yields listed in Supplementary Table 3).

General procedure for the synthesis of 49a – **49g**: Precursor **48a** – **48g** (0.03 mmol) was dissolved in THF/H_2O (0.9 mL/0.3mL) and cooled to 0°C, before addition of H_2O_2 (151 µL) and LiOH (303 µL, 1 M). The reaction was stirred for 16 h at room temperature, after which, methanol (0.5 mL) was added and the reaction was cooled to 0°C before addition of NaOH (365 µL). The reaction was stirred at room temperature for 6 h. When the reaction was complete, it was quenched with Amberlyst-15 and stirred for another 15 min. At pH 3 – 4, the mixture was filtered and solvent was evaporated off. The product was purified using Sephadex G-10 (100% H_2O). Purified product was passed through Sephadex C-25 Na resin (100% H_2O) to afford **49a** – **49g** (Yields listed in Supplementary Table 3).

Compound 49h: Compound **46h** (40mg, 0.053 mmol) was treated as described for the preparation of **49a** – **49g** to afford **49h** (21 mg, 92%).

Acceptor \ Donor	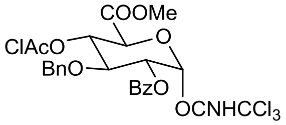 **D3**	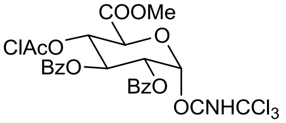 **D4**
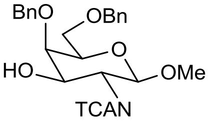 **A1**	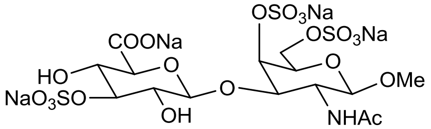 **49a**	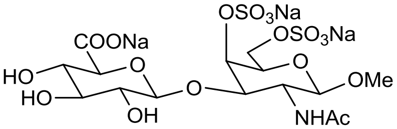 **49e**
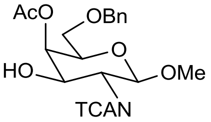 **A2**	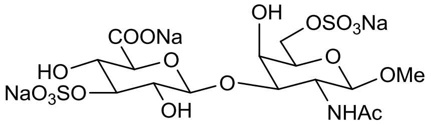 **49b**	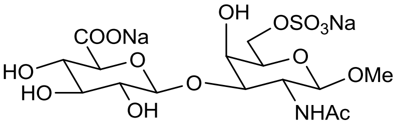 **49f**
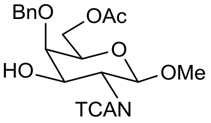 **A3**	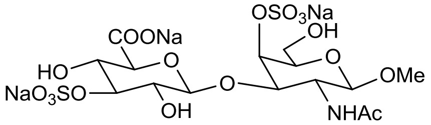 **49c**	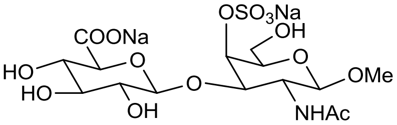 **49g**
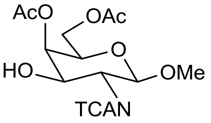 **A4**	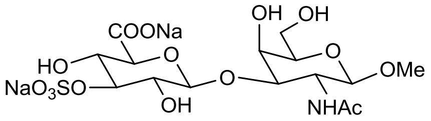 **49d**	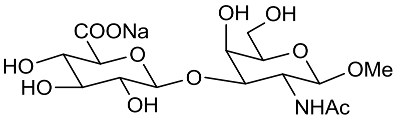 **49h**

Supplementary Table 4: Structures of CS disaccharides **49a** – **49h**

Materials

Cell culture medium RPMI 1640 containing 2.05mM L-Glutamine, Dulbecco's Modified Eagle Medium (DMEM), Dulbecco's Modified Eagle Medium: Nutrient Mixture F-12 (DMEM-F12) and Fetal Bovine Serum (FBS) were purchased from GE Healthcare Life Sciences (HyClone™). Human mammary and breast cancer cell lines MCF-12A, MCF-7, MDA-MB-231 and T47D were obtained from American Type Culture Collection (ATCC, USA), and cultured at 37°C with 5% CO_2. The growth medium used was DMEM supplemented with 10% fetal bovine serum for MCF-7, RPMI-1640 supplemented with 10% fetal bovine serum for T47D and MDA-MB-231; and DMEM-F12 supplemented with 5% FBS, 0.5µg/mL hydrocortisone, 20ng/mL EGF, 10µg/mL insulin, 40µg/mL gentamicin and 100ng/mL cholera toxin for MCF-12A. CellTiter 96® AQueous One Solution Cell Proliferation Assay (MTS assay) kit and Caspase-Glo® 3/7 Assay System were purchased from Promega (Wisconsin, USA). Absorbance readings were taken using the Tecan Infinite F200 Pro plate reader with the Tecan i-control software, while the luminescence readings were taken using the SpectraMax M5 plate reader using the SpectraMax software (integration time= 1 sec). The readings obtained were normalized with respect to the control data and statistical analysis calculated using GraphPad Prism software; level of statistical significance was set at $p <0.05$.

Cell viability assay (MTS)

The cells were plated onto a 96-well plate and cultured for 24 hours at 37°C (5% CO_2) with 100µL complete medium/well. The seeding density used was 2500 cells/well for MCF-12A; 4000 cells/well for MCF-7; 5000 cells/well for MDA-MB-231 and 6000 cells/well for T47D. After 24h growth period, the cells were treated with the desired CS disaccharide at 4 different concentrations: 0.1µg/mL, 1µg/mL, 10µg/mL and 100µg/mL. A control group was included where only the drug vehicle was used. Six biological replicates were set up for each group. Upon addition of the drug, the cells were incubated at 37°C (5% CO_2) for 72 hours. After removal of the medium and washing of the cells with phosphate-buffered saline (PBS), the CellTiter 96® AQueous One Solution (MTS reagent) was next added to each well (100µL complete medium + 20µL MTS reagent per well) and the cells were incubated at 37°C (5% CO_2) in the absence of light. Absorbance readings (λ=490nm) taken at the 3rd hour were analyzed using one-way Analysis of Variance (ANOVA) with post-hoc Dunnett's test.

Apoptosis assay

MDA-MB-231 cells were plated on a 6-well plate with a seeding density of 200,000 cells/well and cultured for 24 hours at 37°C (5% CO_2) with 2mL complete medium/well. After 24h, the cells were treated with the desired CS disaccharide at 100µg/mL drug concentration and incubated for a further 48 hours. A control set was included where only the drug vehicle was used. To facilitate luminescence measurements, the cells were then reseeded into a white opaque 96-well plate at a seeding density of 20,000 cells/well and incubated for an additional 24 hours. Thereafter, 100µL of Caspase-Glo® 3/7 reagent was added to each well (n=6). The contents in the wells were gently mixed and were incubated at room temperature in the dark. Luminescence readings taken at after 1 hr were analyzed using one-way Analysis of Variance (ANOVA) with post-hoc Dunnett's test.

Compound 2

^{1}H NMR (500 MHz, $CDCl_3$) δ 7.52 – 7.46 (m, 2H), 7.35 – 7.27 (m, 3H), 5.22 (t, *J* = 9.4 Hz, 1H), 5.03 (t, *J* = 9.8 Hz, 1H), 4.97 (t, *J* = 9.7 Hz, 1H), 4.70 (d, *J* = 10.1 Hz, 1H), 4.19 (qd, *J* = 12.2, 3.8 Hz, 2H), 3.72 (ddd, *J* = 10.0, 5.1, 2.5 Hz, 1H), 2.07 (d, *J* = 3.8 Hz, 6H), 1.99 (d, *J* = 14.4 Hz, 6H). ^{13}C NMR (500 MHz, $CDCl_3$) δ 170.5, 170.1, 169.3, 169.2, 133.1, 131.6, 128.9, 128.4, 85.7, 75.8, 73.9, 69.9, 68.2, 62.1, 20.7, 20.7, 20.5, 20.5. HRMS (ESI): *m/z*: calcd for $C_{20}H_{24}NaO_9S$ [M + Na]: 463.1033; found: 463.1041.

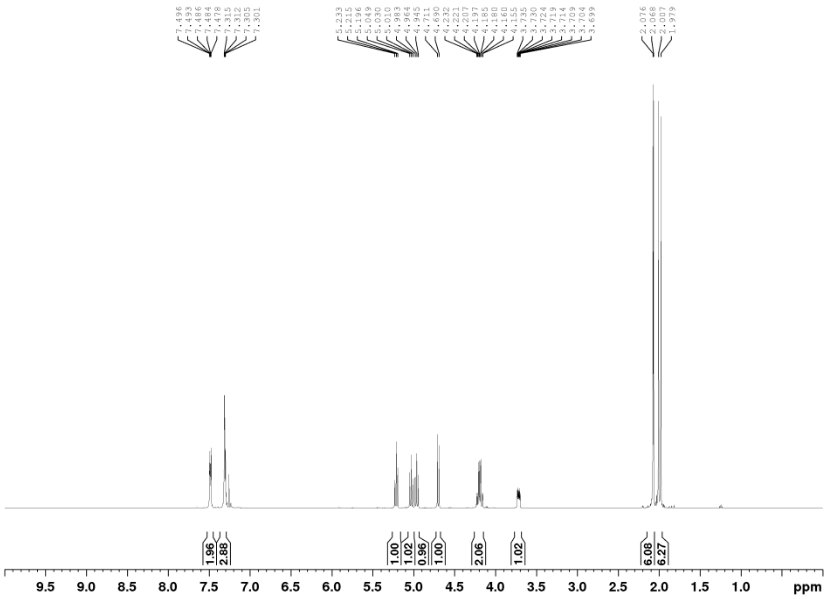

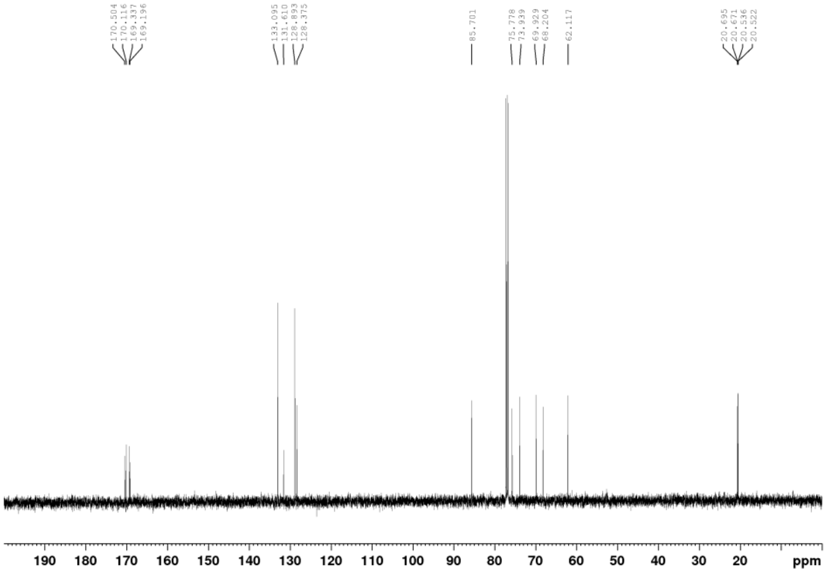

Compound 3

^{1}H NMR (500 MHz, $CDCl_3$) δ 7.58 – 7.51 (m, 2H), 7.52 – 7.45 (m, 2H), 7.41 – 7.31 (m, 6H), 5.52 (s, 1H), 4.62 (dd, *J* = 10.0, 0.8 Hz, 1H), 4.37 (dd, *J* = 10.4, 3.1 Hz, 1H), 3.88 – 3.72 (m, 2H), 3.48 (dq, *J* = 22.0, 9.1 Hz, 3H), 2.99 (s, 1H), 2.81 (s, 1H). ^{13}C NMR (500 MHz, $CDCl_3$) δ 136.8, 133.0, 131.3, 129.3, 129.1, 128.4, 128.3, 126.3, 101.9, 88.6, 80.2, 74.6, 72.6, 70.5, 68.5. HRMS (ESI): *m/z*: calcd for $C_{19}H_{20}NaO_5S$ [M + Na]: 383.0924; found: 383.0923.

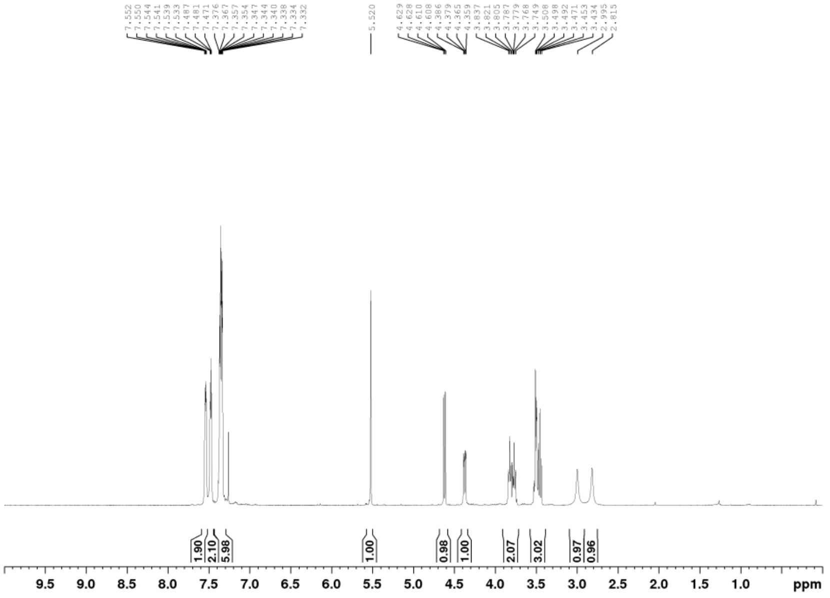

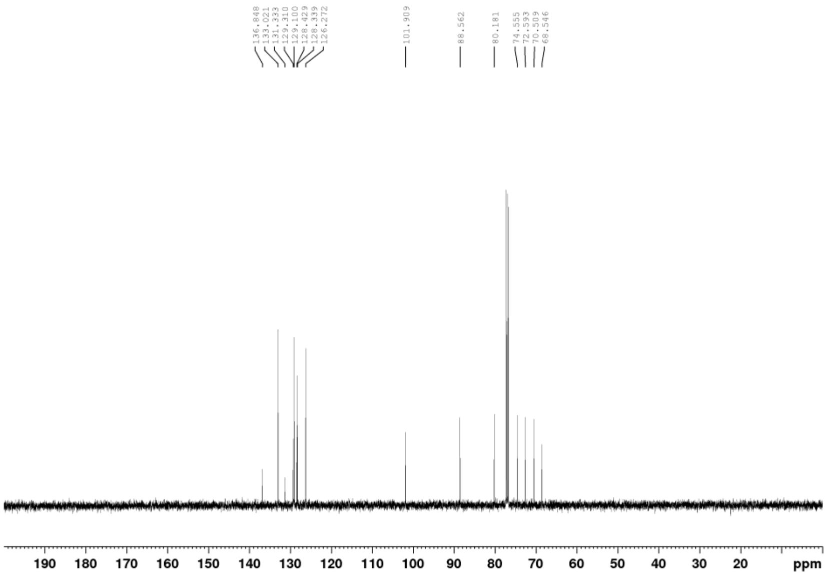

Compound 5

^{1}H NMR (500 MHz, $CDCl_3$) δ 7.52 (ddd, *J* = 27.8, 6.9, 2.5 Hz, 4H), 7.42 – 7.27 (m, 11H), 5.58 (s, 1H), 4.88 (dd, *J* = 80.6, 11.5 Hz, 2H), 4.64 (d, *J* = 9.7 Hz, 1H), 4.40 (dd, *J* = 10.5, 5.0 Hz, 1H), 3.80 (t, *J* = 10.3 Hz, 1H), 3.73 – 3.62 (m, 2H), 3.60 – 3.46 (m, 2H). ^{13}C NMR (500 MHz, $CDCl_3$) δ 138.1, 137.1, 133.4, 131.3, 129.0, 128.4, 128.3, 128.2, 128.1, 127.9, 126.0, 101.2, 88.4, 81.6, 81.1, 74.8, 72.2, 70.7, 68.6. HRMS (ESI): *m/z*: calcd for $C_{26}H_{26}NaO_5S$ [M + Na]: 473.1393; found: 473.1388.

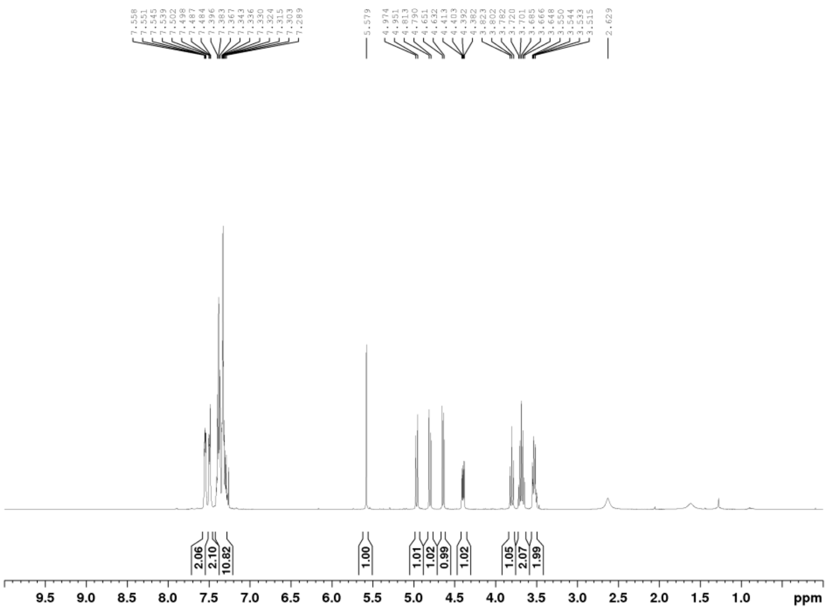

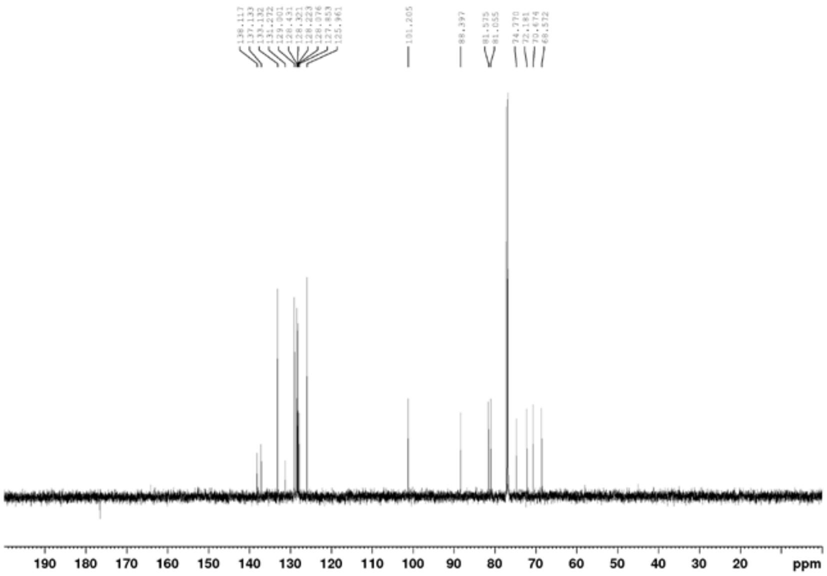

Compound 5

2D COSY

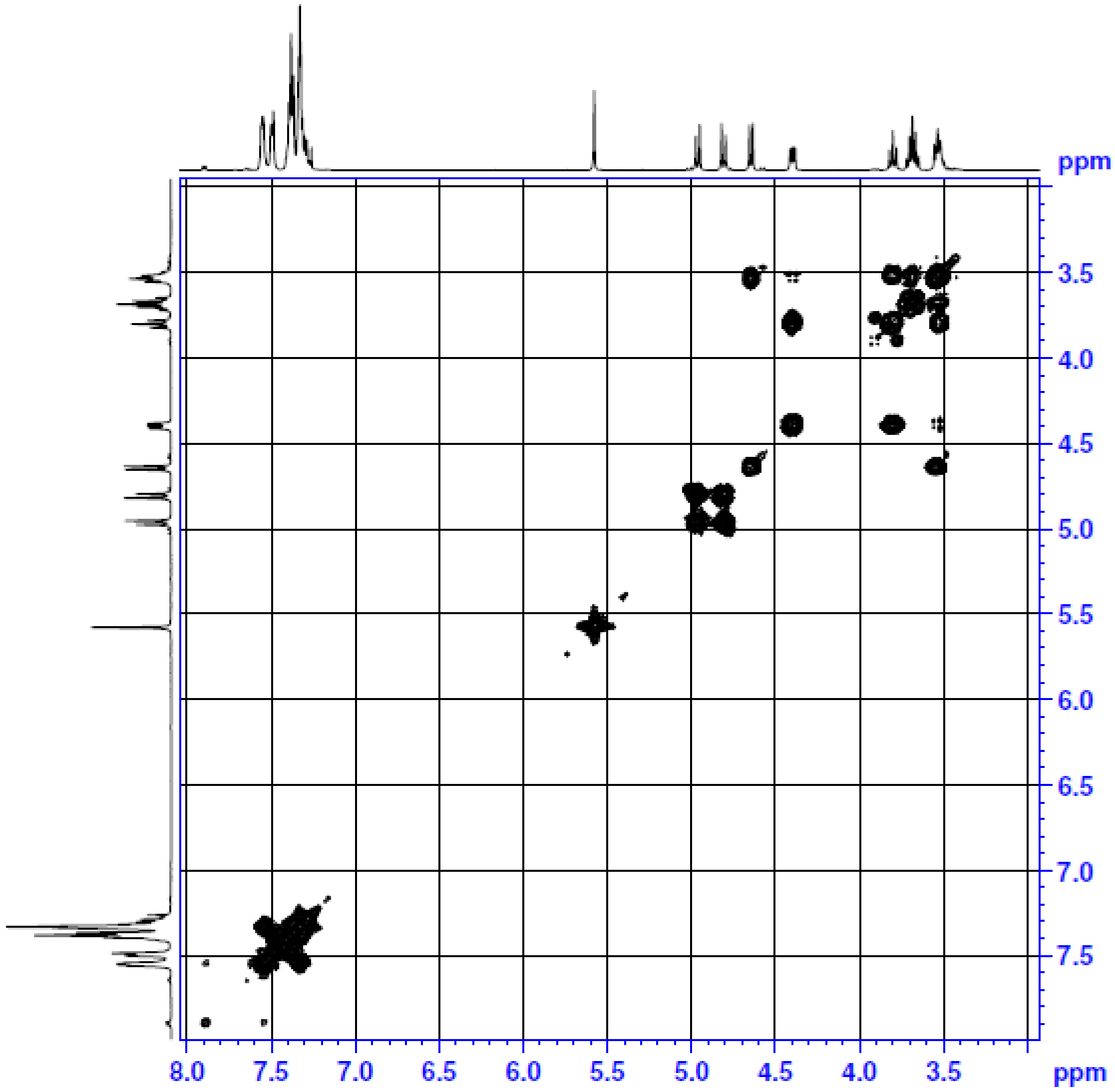

Compound 5

2D HMBC

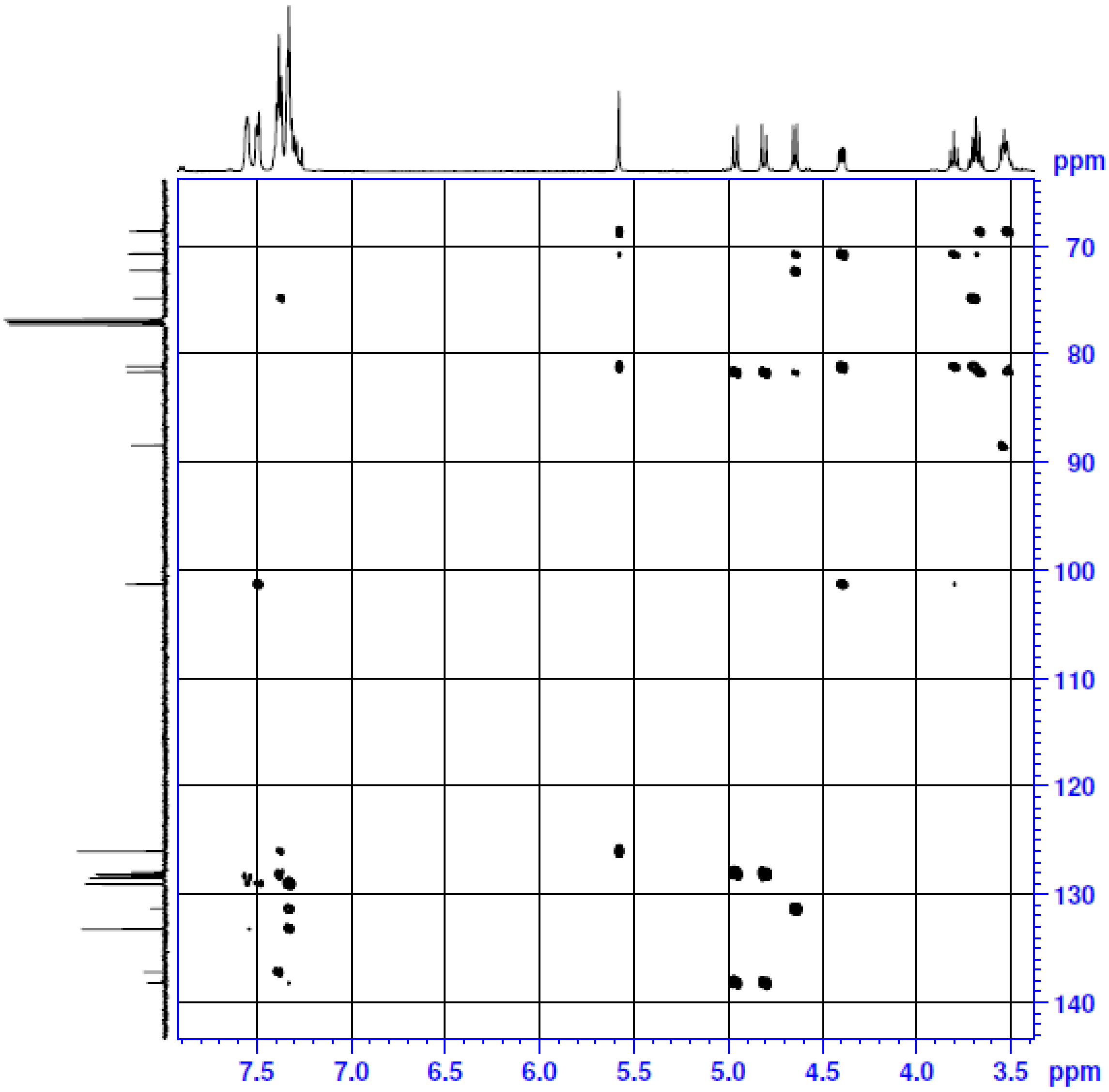

Compound 5

2D HSQC

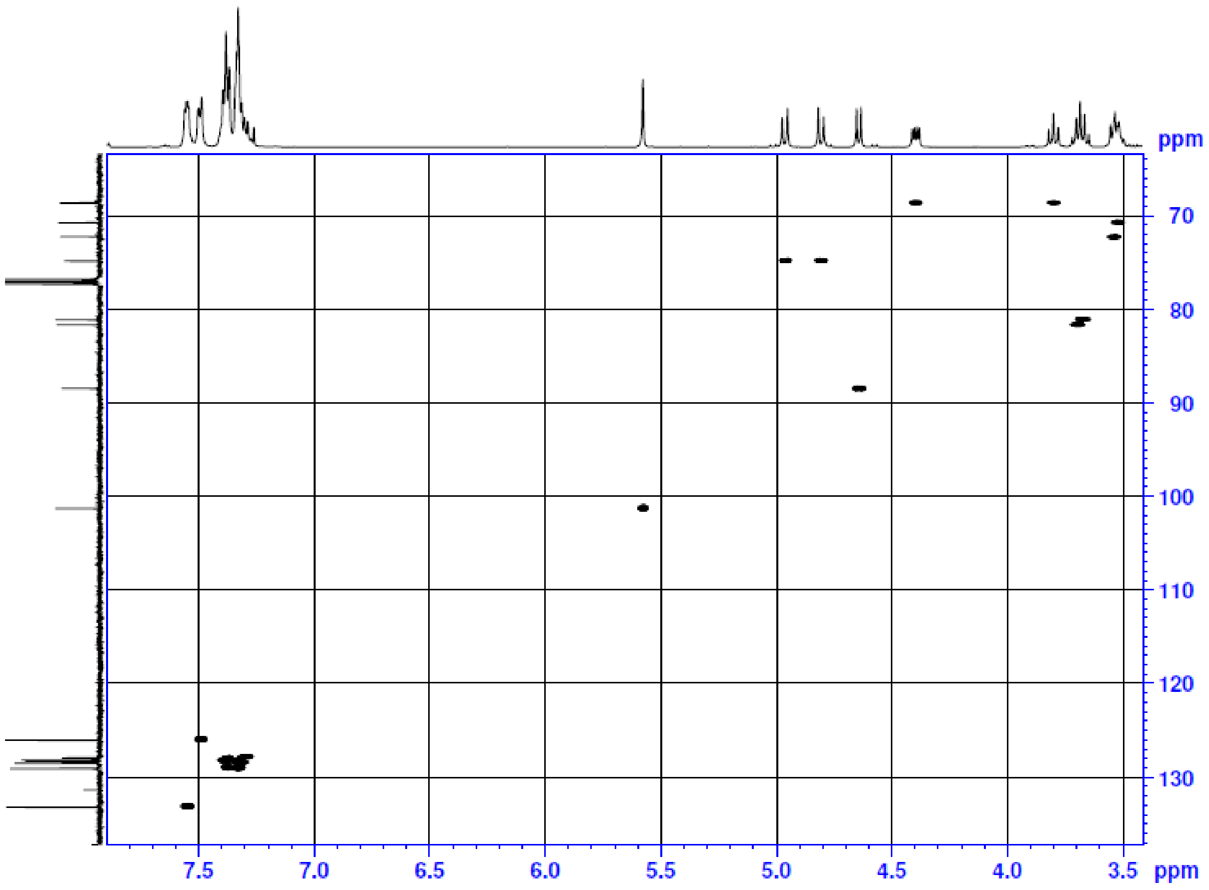

Compound 7

^{1}H NMR (500 MHz, $CDCl_3$) δ 8.04 (dd, *J* = 29.4, 7.4 Hz, 4H), 7.59 – 7.55 (m, 2H), 7.48 (dd, *J* = 14.2, 7.2 Hz, 2H), 7.33 (ddd, *J* = 14.9, 10.9, 5.1 Hz, 7H), 7.23 – 7.16 (m, 5H), 6.01 – 5.92 (m, 1H), 5.58 (t, *J* = 9.7 Hz, 1H), 5.19 (d, *J* = 10.0 Hz, 1H), 4.71 (dd, *J* = 27.9, 11.1 Hz, 2H), 4.10 – 4.01 (m, 2H), 3.93 (dd, *J* = 12.1, 3.4 Hz, 1H), 3.81 – 3.74 (m, 1H), 3.04 (s, 1H). ^{13}C NMR (500 MHz, $CDCl_3$) δ 165.4, 165.0, 136.9, 132.9, 132.8, 132.3, 131.9, 129.4, 129.3, 129.0, 128.8, 128.7, 128.0, 128.0, 127.8, 127.6, 127.5, 85.8, 79.4, 76.0, 75.1, 74.4, 70.7, 61.0. HRMS (ESI): *m/z*: calcd for $C_{33}H_{30}NaO_7S$ [M + Na]: 593.1604; found: 593.1609.

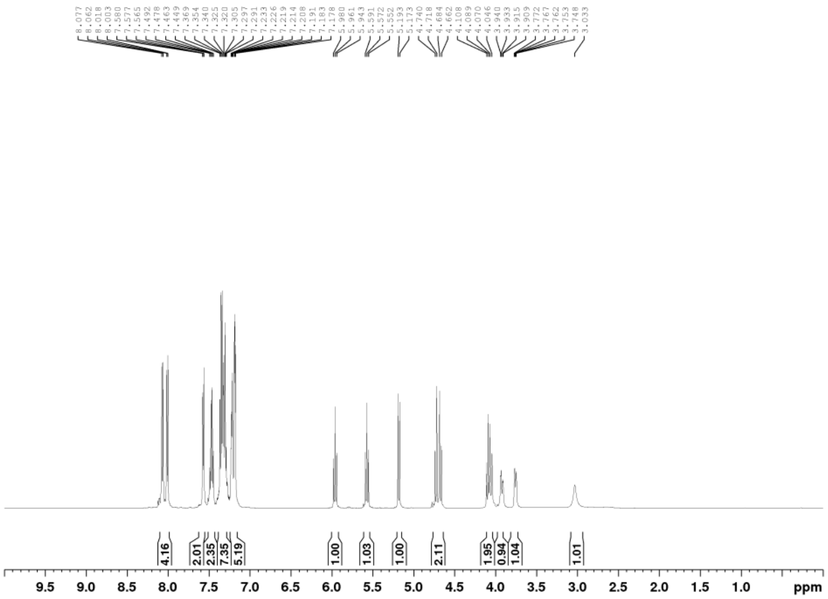

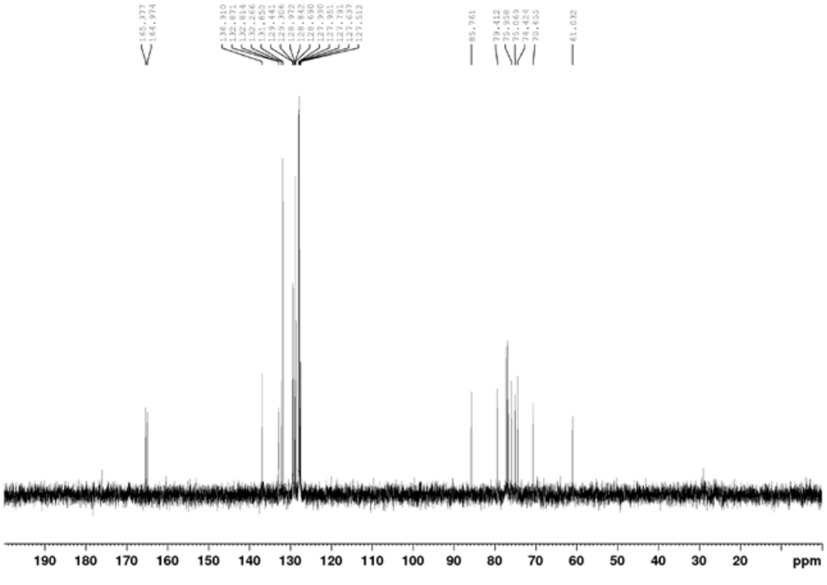

Compound 8

^{1}H NMR (500 MHz, $CDCl_3$) δ 8.11 – 8.07 (m, 2H), 7.62 (t, *J* = 7.5 Hz, 1H), 7.53 – 7.43 (m, 4H), 7.40 – 7.28 (m, 8H), 7.20 – 7.10 (m, 5H), 5.38 – 5.27 (m, 1H), 4.93 – 4.85 (m, 2H), 4.79 (s, 1H), 4.72 (d, *J* = 11.0 Hz, 2H), 3.99 – 3.82 (m, 2H), 3.82 – 3.70 (m, 2H), 3.57 (ddd, *J* = 9.6, 4.7, 2.5 Hz, 1H), 2.17 (s, 1H). ^{13}C NMR (500 MHz, $CDCl_3$) δ 165.1, 137.7, 137.5, 133.1, 132.6, 132.3, 129.7, 129.7, 128.9, 128.4, 128.3, 128.2, 128.0, 127.9, 127.9, 127.9, 127.6, 86.1, 83.9, 79.6, 77.4, 75.2, 75.0, 72.4, 61.9. HRMS (ESI): *m/z*: calcd for $C_{33}H_{32}NaO_6S$ [M + Na]: 579.1812; found: 579.1814.

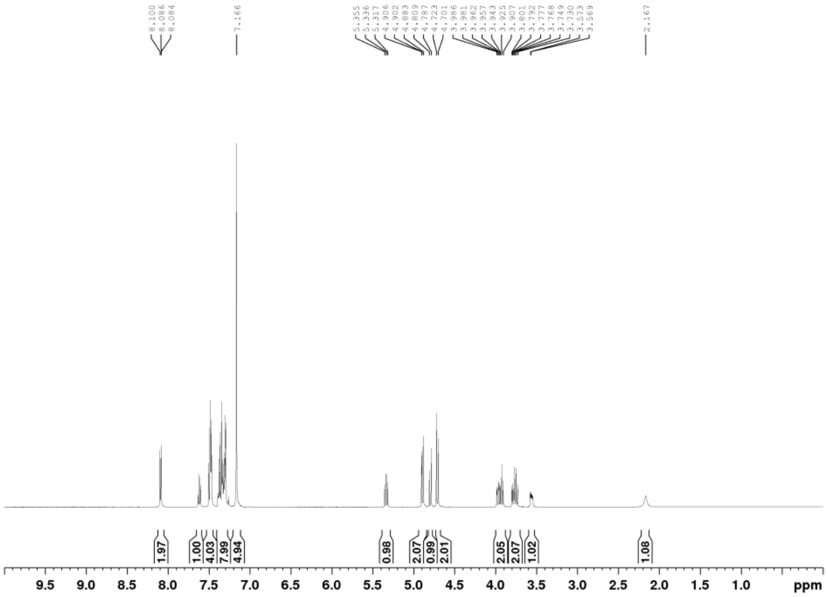

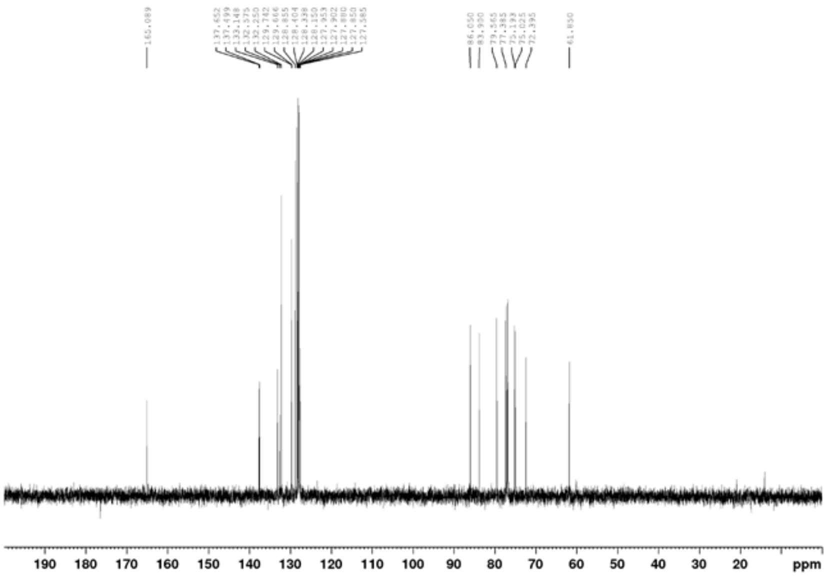

Compound 9

^{1}H NMR (500 MHz, $CDCl_3$) δ 7.95 (dd, *J* = 13.0, 7.9 Hz, 4H), 7.55 – 7.42 (m, 4H), 7.37 (dt, *J* = 12.6, 7.7 Hz, 4H), 7.33 – 7.28 (m, 3H), 5.43 (dd, *J* = 7.4, 4.0 Hz, 2H), 5.01 – 4.91 (m, 1H), 4.02 (dd, *J* = 12.0, 3.1 Hz, 1H), 3.91 (ddd, *J* = 16.9, 13.7, 7.0 Hz, 2H), 3.67 – 3.55 (m, 1H). ^{13}C NMR (500 MHz, $CDCl_3$) δ 167.5, 165.2, 133.6, 133.3, 132.7, 132.0, 130.0, 129.8, 129.2, 129.0, 128.8, 128.4, 128.4, 128.3, 86.1, 80.0, 78.3, 70.2, 69.8, 62.4. HRMS (ESI): *m/z*: calcd for $C_{26}H_{24}NaO_7S$ [M + Na]: 503.1135; found: 503.1130.

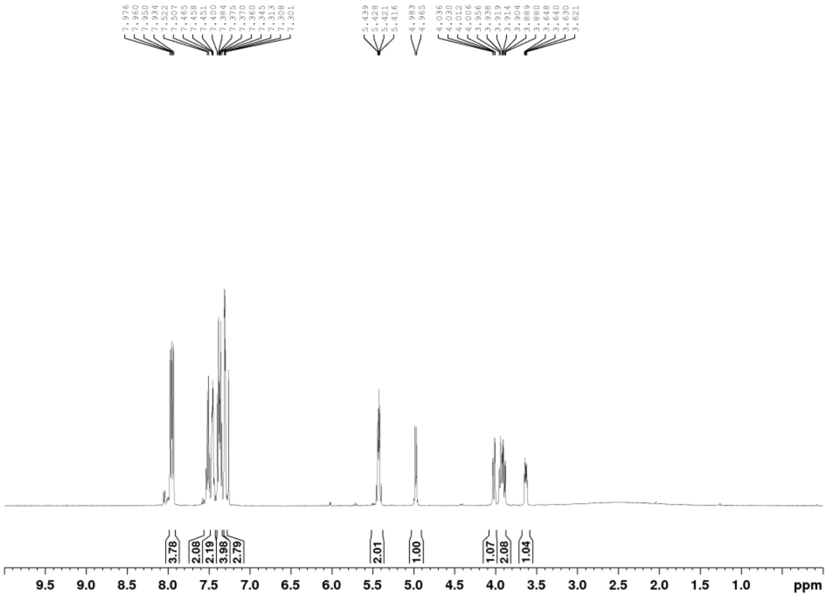

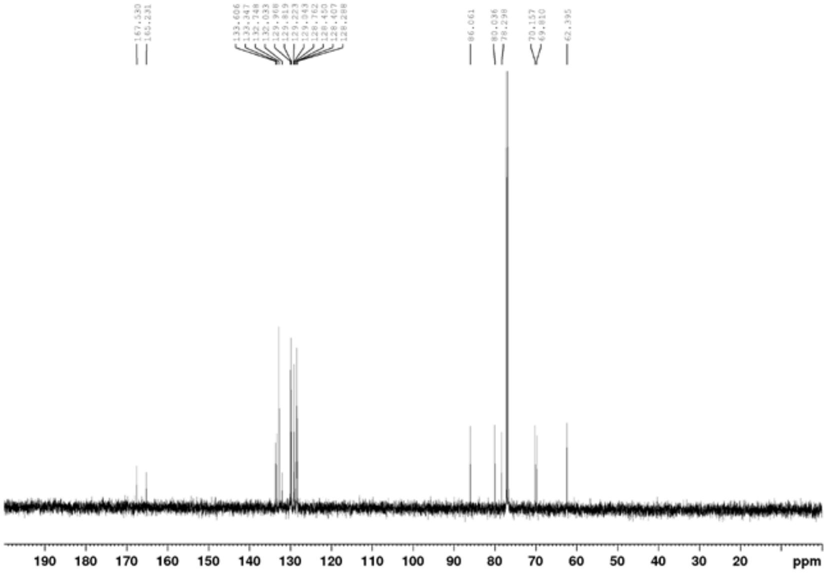

Compound 10

^{1}H NMR (500 MHz, $CDCl_3$) δ 8.08 (d, *J* = 8.0 Hz, 2H), 7.61 (dd, *J* = 10.9, 4.2 Hz, 1H), 7.52 – 7.36 (m, 4H), 7.31 – 7.24 (m, 3H), 7.22 – 7.11 (m, 5H), 5.35 – 5.20 (m, 1H), 4.85 (d, *J* = 10.1 Hz, 1H), 4.67 (dd, *J* = 57.9, 11.4 Hz, 2H), 3.94 (d, *J* = 11.9 Hz, 1H), 3.85 – 3.76 (m, 1H), 3.76 – 3.67 (m, 2H), 3.48 (dt, *J* = 8.4, 4.4 Hz, 1H). ^{13}C NMR (500 MHz, $CDCl_3$) δ 165.2, 137.6, 133.3, 132.7, 132.3, 129.8, 128.9, 128.5, 128.0, 128.0, 86.4, 83.8, 79.4, 74.8, 72.3, 70.3, 62.5. HRMS (ESI): *m/z*: calcd for $C_{26}H_{26}NaO_6S$ [M + Na]: 489.1342; found: 489.1346.

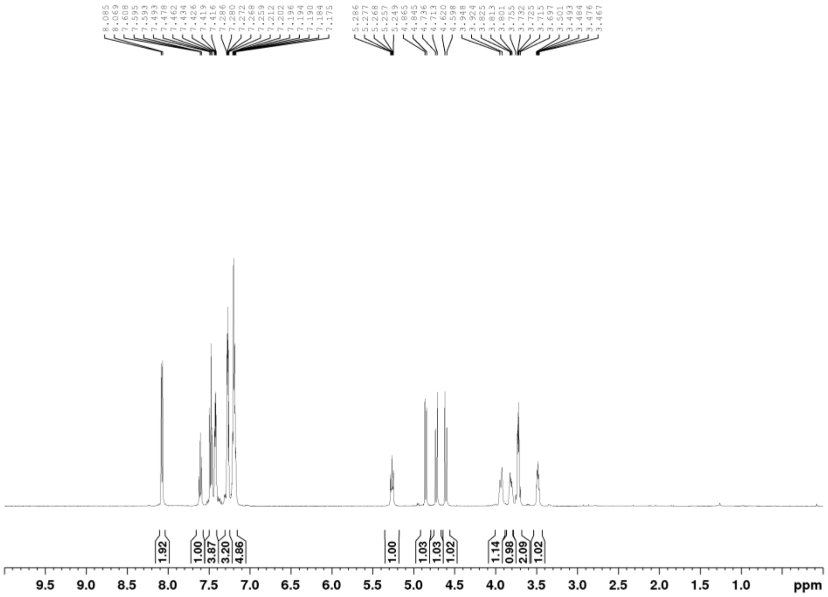

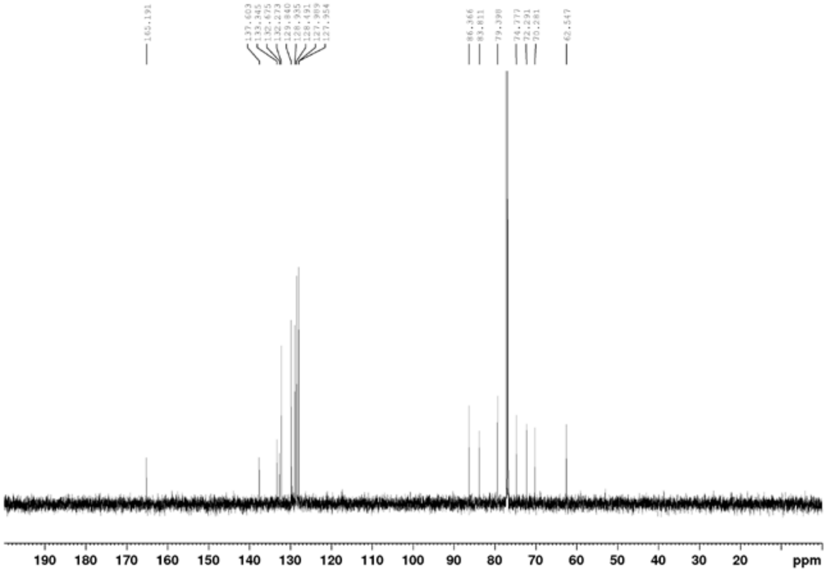

Compound 11

^{1}H NMR (500 MHz, $CDCl_3$) δ 7.99 (dd, *J* = 21.7, 7.9 Hz, 4H), 7.56 – 7.48 (m, 4H), 7.41 – 7.30 (m, 7H), 7.17 (dt, *J* = 7.1, 3.9 Hz, 5H), 5.86 (t, *J* = 8.9 Hz, 1H), 5.50 (t, *J* = 9.6 Hz, 1H), 5.09 (d, *J* = 9.8 Hz, 1H), 4.62 (dd, *J* = 31.8, 11.1 Hz, 2H), 4.27 (dd, *J* = 14.3, 9.1 Hz, 2H), 3.80 (s, 3H). ^{13}C NMR (500 MHz, $CDCl_3$) δ 167.9, 165.3, 164.9, 136.8, 133.1, 132.6, 131.7, 129.6, 129.5, 128.9, 128.8, 128.2, 128.1, 128.1, 127.8, 127.7, 86.6, 77.8, 77.0, 75.3, 74.5, 70.2, 52.5. HRMS (ESI): *m/z*: calcd for $C_{34}H_{30}NaO_8S$ [M + Na]: 621.1554; found: 621.1556.

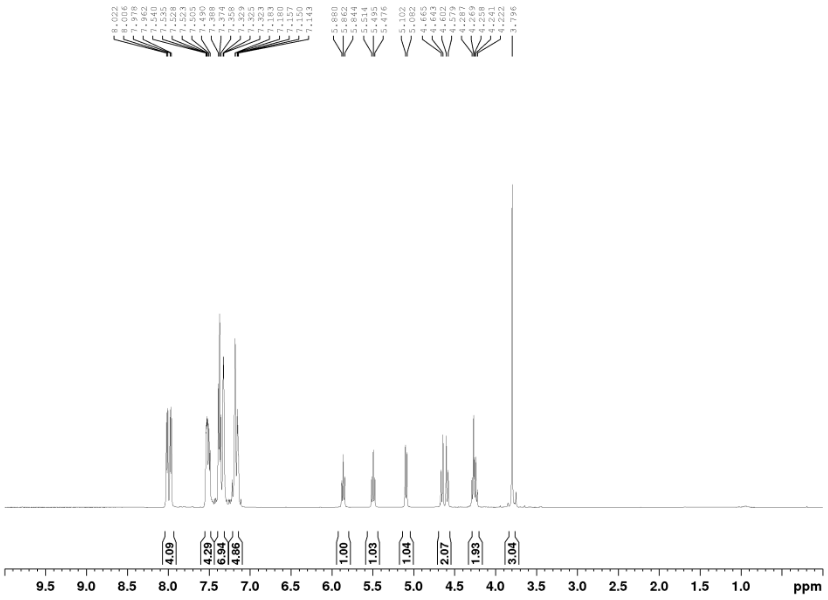

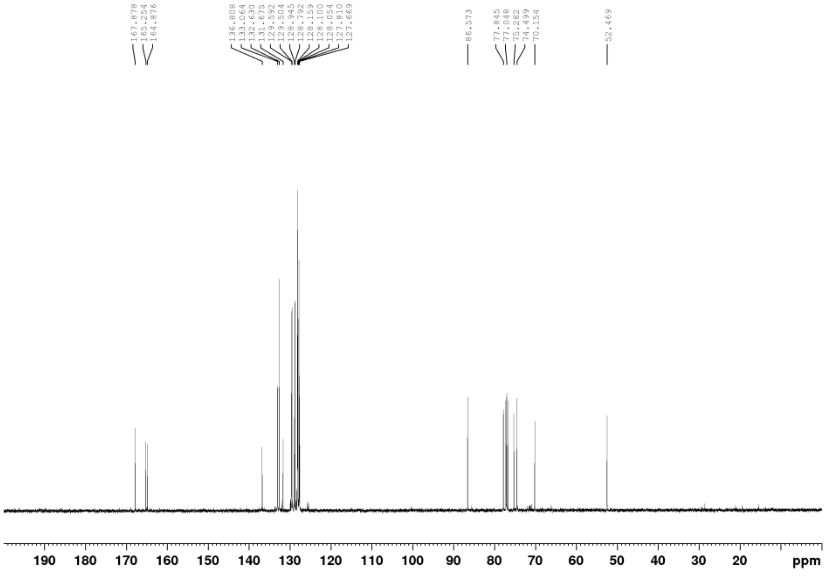

Compound 12

^{1}H NMR (500 MHz, $CDCl_3$) δ 8.06 (dd, *J* = 8.1, 1.0 Hz, 2H), 7.65 – 7.57 (m, 1H), 7.48 (ddd, *J* = 6.2, 5.0, 3.0 Hz, 4H), 7.41 – 7.22 (m, 8H), 7.20 – 7.08 (m, 5H), 5.44 – 5.27 (m, 1H), 4.90 – 4.72 (m, 3H), 4.68 (dd, *J* = 11.0, 4.6 Hz, 2H), 4.08 (d, *J* = 9.5 Hz, 1H), 4.01 (t, *J* = 9.2 Hz, 1H), 3.90 (t, *J* = 8.9 Hz, 1H), 3.78 (s, 3H). ^{13}C NMR (500 MHz, $CDCl_3$) δ 168.2, 164.9, 137.5, 137.3, 133.2, 132.6, 132.2, 129.7, 129.6, 128.8, 128.3, 128.3, 128.1, 128.0, 127.9, 127.9, 127.8, 127.6, 86.9, 83.1, 79.0, 78.0, 75.1, 75.0, 71.7, 52.5. HRMS (ESI): *m/z*: calcd for $C_{34}H_{32}NaO_7S$ [M + Na]: 607.1761; found: 607.1767.

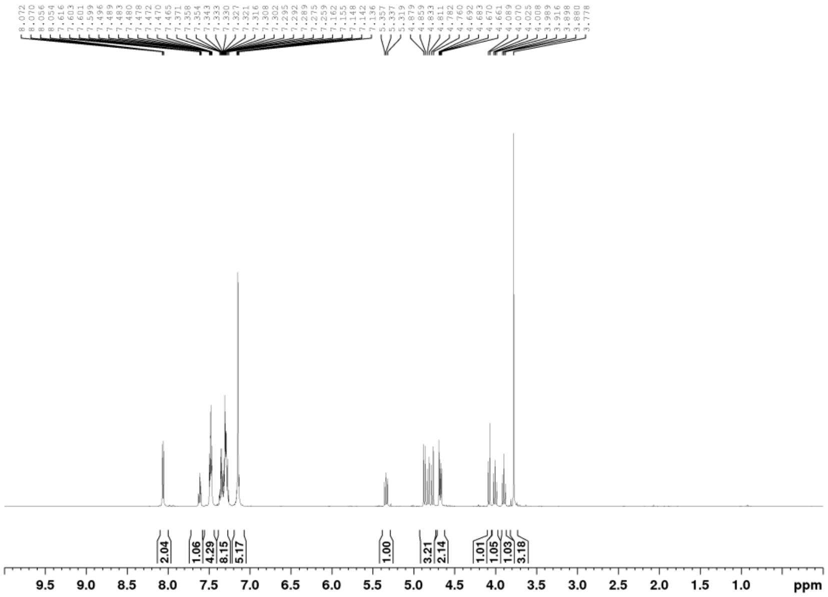

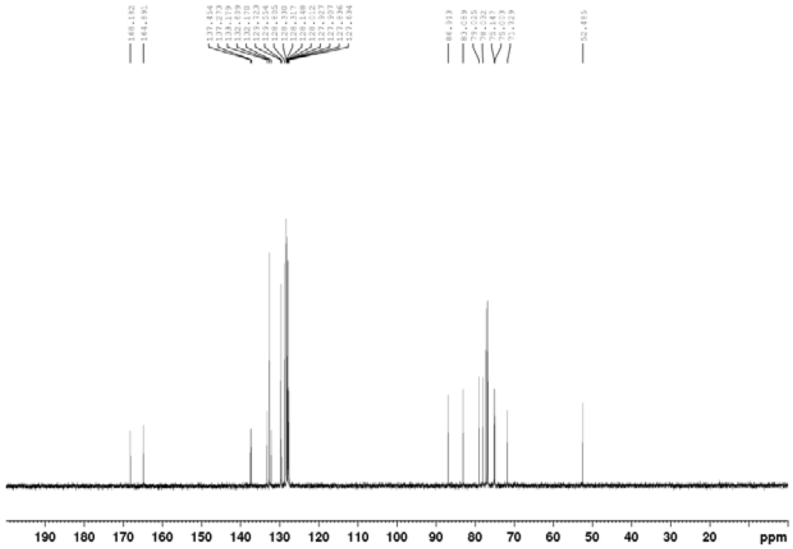

Compound 13

^{1}H NMR (500 MHz, $CDCl_3$) δ 8.00 – 7.88 (m, 4H), 7.58 – 7.45 (m, 4H), 7.42 – 7.33 (m, 4H), 7.33 – 7.28 (m, 3H), 5.55 (t, *J* = 9.2 Hz, 1H), 5.42 (t, *J* = 9.7 Hz, 1H), 4.97 (d, *J* = 10.0 Hz, 1H), 4.18 (td, *J* = 9.4, 2.1 Hz, 1H), 4.11 (d, *J* = 9.6 Hz, 1H), 3.89 (s, 3H), 3.32 (s, 1H). ^{13}C NMR (500 MHz, $CDCl_3$) δ 168.8, 166.6, 165.1, 133.4, 133.3, 133.0, 131.9, 129.9, 129.8, 129.1, 129.0, 128.9, 128.4, 128.4, 87.0, 78.1, 76.2, 70.4, 69.7, 53.0. HRMS (ESI): *m/z*: calcd for $C_{27}H_{24}NaO_8S$ [M + Na]: 531.1084; found: 531.1092.

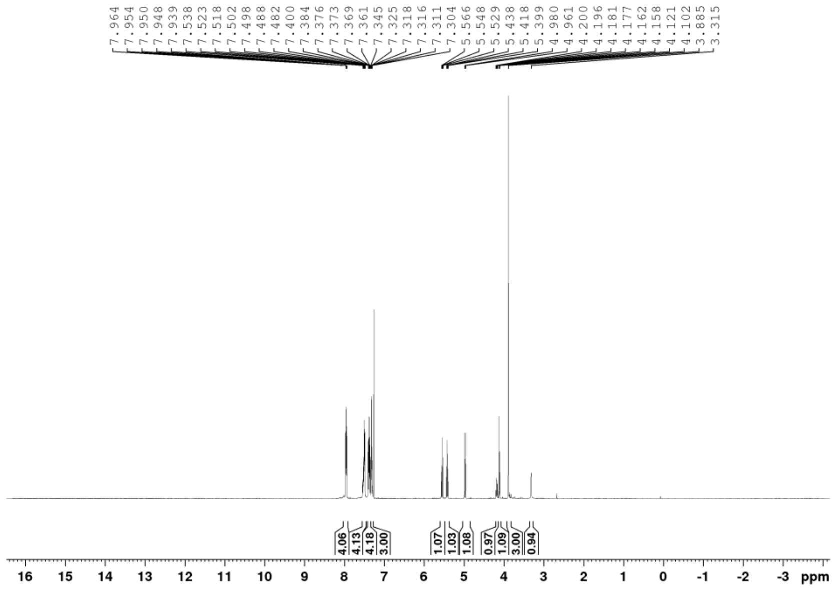

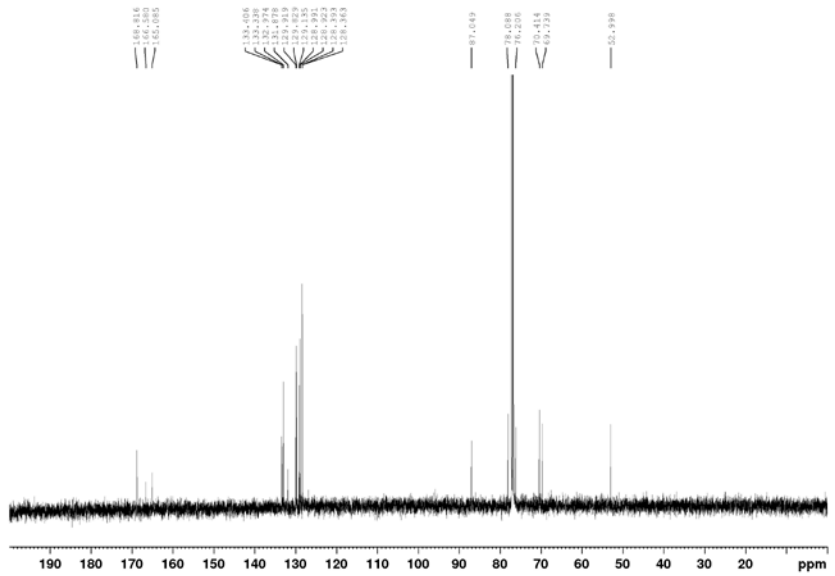

Compound 14

^{1}H NMR (500 MHz, $CDCl_3$) δ 8.05 (d, *J* = 7.2 Hz, 2H), 7.63 (t, *J* = 7.5 Hz, 1H), 7.49 (dd, *J* = 9.2, 5.9 Hz, 4H), 7.33 – 7.24 (m, 3H), 7.21 – 7.12 (m, 5H), 5.33 – 5.23 (m, 1H), 4.85 (d, *J* = 10.1 Hz, 1H), 4.76 (q, *J* = 11.6 Hz, 2H), 4.05 (d, *J* = 9.2 Hz, 1H), 3.97 (d, *J* = 9.7 Hz, 1H), 3.88 (s, 3H), 3.76 (t, *J* = 8.9 Hz, 1H). ^{13}C NMR (500 MHz, $CDCl_3$) δ 169.3, 165.0, 137.6, 133.3, 132.7, 132.4, 129.8, 129.6, 128.9, 128.4, 128.3, 128.1, 128.0, 127.7, 87.2, 82.2, 77.6, 74.7, 71.9, 71.3, 52.9. HRMS (ESI): *m/z*: calcd for $C_{27}H_{26}NaO_7S$ [M + Na]: 517.1291; found: 517.1298.

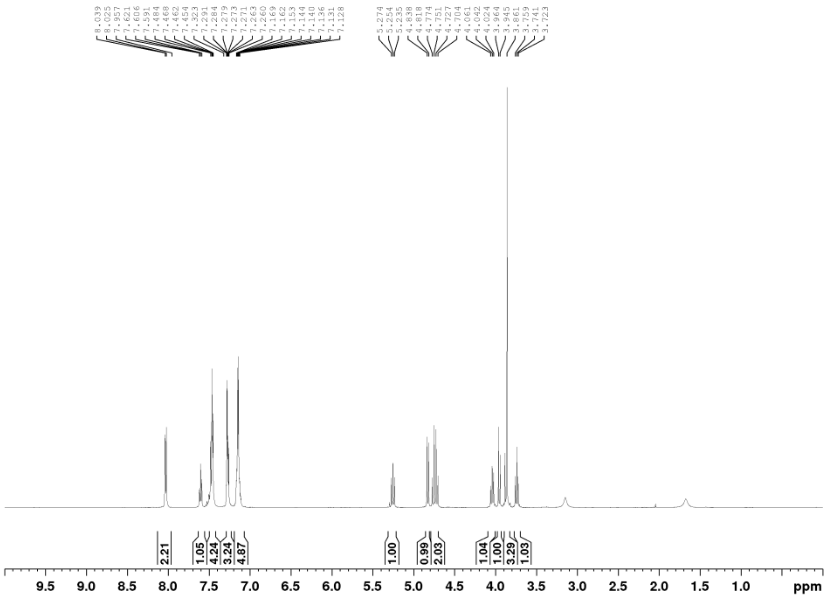

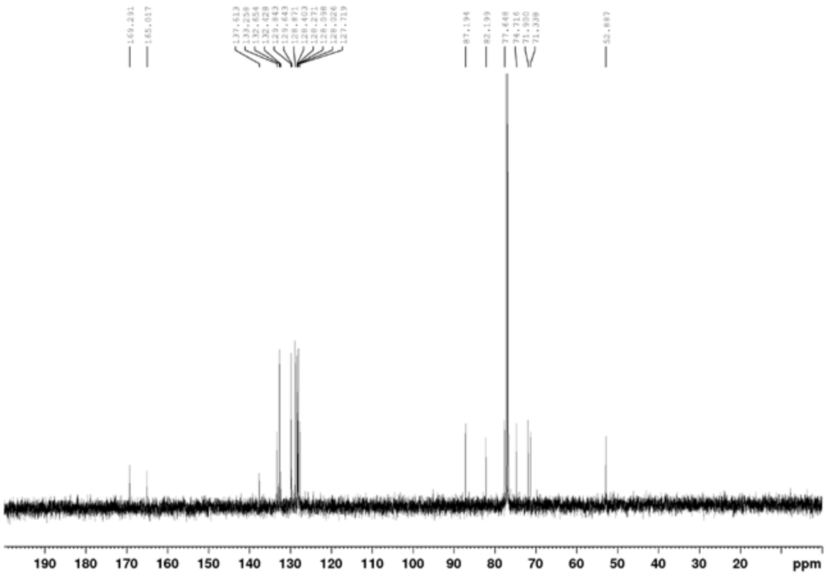

Compound 17

^{1}H NMR (500 MHz, $CDCl_3$) δ 7.97 (ddd, *J* = 14.0, 10.1, 5.4 Hz, 4H), 7.58 – 7.42 (m, 2H), 7.43 – 7.27 (m, 4H), 7.21 – 7.07 (m, 5H), 6.13 (t, *J* = 9.6 Hz, 1H), 5.74 (d, *J* = 3.5 Hz, 1H), 5.24 (dd, *J* = 10.0, 3.6 Hz, 1H), 4.75 (d, *J* = 9.7 Hz, 1H), 4.69 – 4.47 (m, 2H), 4.30 – 4.06 (m, 1H), 3.73 (t, *J* = 4.9 Hz, 3H). ^{13}C NMR (500 MHz, $CDCl_3$) δ 169.7, 168. 8, 166.3, 165.7, 165.5, 137.0, 136.8, 133.3, 133.2, 133.1, 129.7, 129.6, 129.6, 128.3, 128.2, 128.1, 127.9, 95.8, 90.5, 74.6, 74.4, 74.3, 73.7, 73.6, 71.8, 71.5, 69.8, 52.5. HRMS (ESI): *m/z*: calcd for $C_{28}H_{26}NaO_9$ [M + Na]: 529.1469; found: 529.1473.

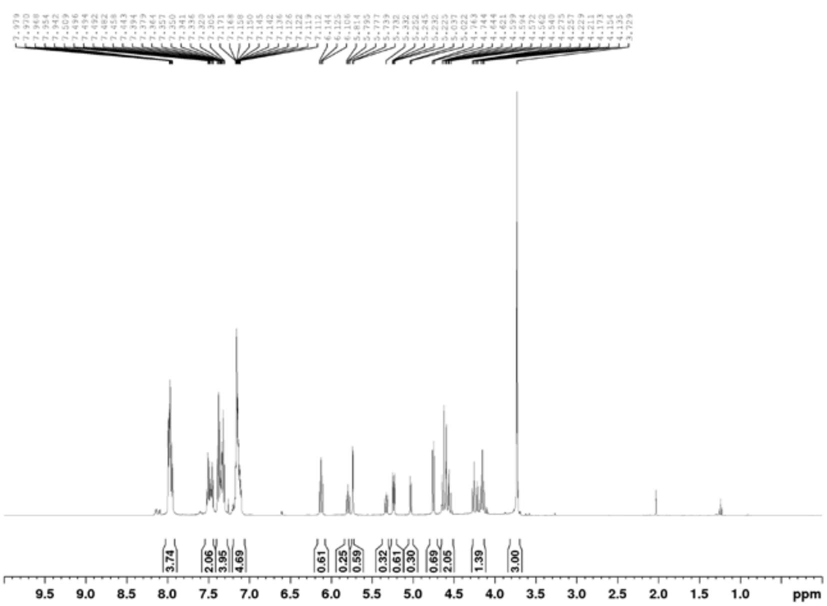

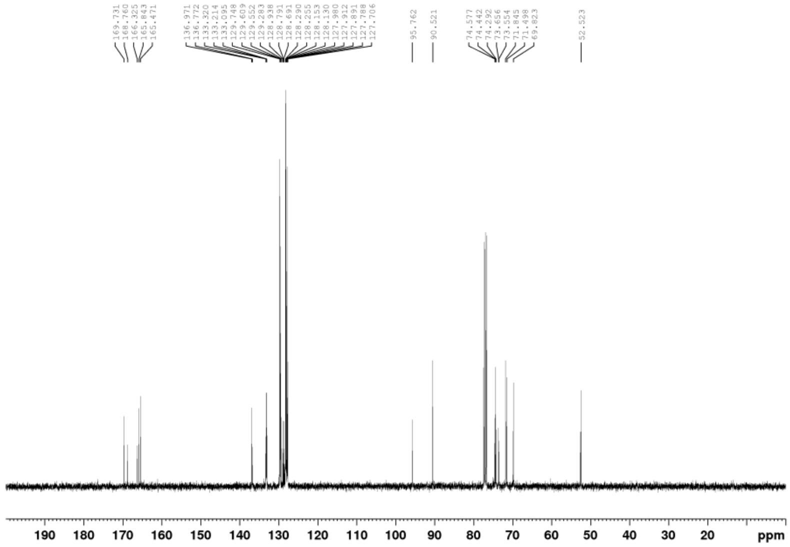

Compound 18

^{1}H NMR (500 MHz, $CDCl_3$) δ 8.08 – 8.02 (m, 2H), 7.57 (t, *J* = 7.4 Hz, 1H), 7.43 (t, *J* = 7.7 Hz, 2H), 7.37 – 7.15 (m, 10H), 5.59 (d, *J* = 3.6 Hz, 1H), 5.15 (dd, *J* = 9.7, 3.5 Hz, 1H), 4.87 – 4.76 (m, 3H), 4.62 (dd, *J* = 32.8, 10.2 Hz, 2H), 4.25 (t, *J* = 9.2 Hz, 1H), 3.96 – 3.87 (m, 1H), 3.73 (s, 4H). ^{13}C NMR (500 MHz, $CDCl_3$) δ 169.8, 165.8, 137.8, 137.7, 133.3, 129.8, 128.4, 128.3, 128.3, 127.9, 127.8, 127.8, 127.7, 90.7, 79.2, 78.7, 75.4, 75.0, 73.4, 70.3, 52.5. HRMS (ESI): *m/z*: calcd for $C_{28}H_{28}NaO_8$ [M + Na]: 515.1676; found: 515.1681.

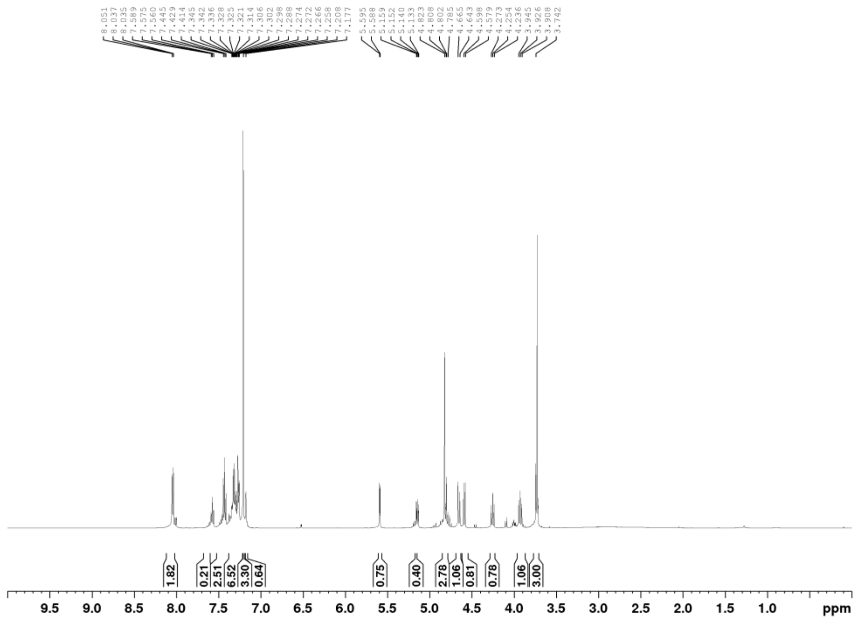

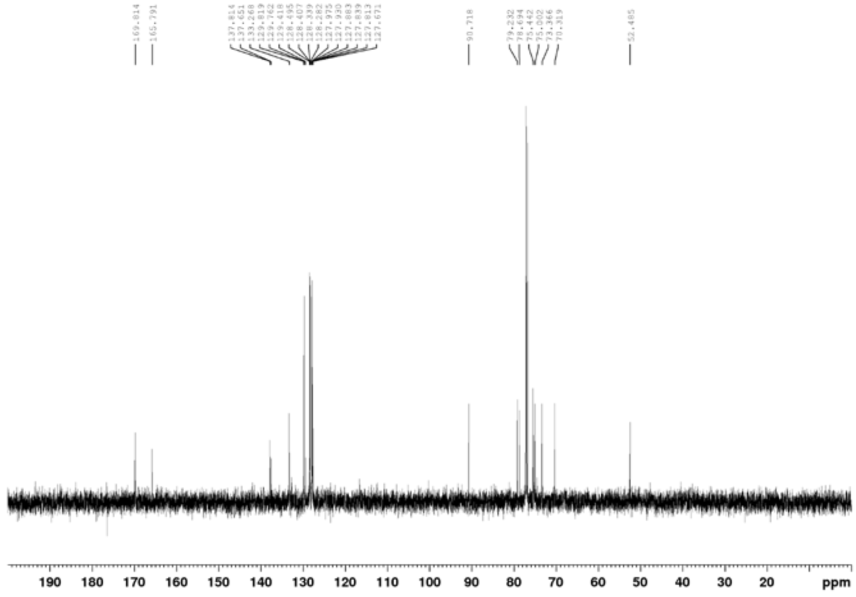

Compound 19

^{1}H NMR (500 MHz, $CDCl_3$) δ 7.95 (dt, *J* = 16.5, 8.1 Hz, 4H), 7.56 – 7.48 (m, 2H), 7.42 – 7.34 (m, 4H), 6.08 (t, *J* = 9.7 Hz, 1H), 5.80 (t, *J* = 4.5 Hz, 1H), 5.57 – 5.46 (m, 1H), 5.30 – 5.20 (m, 1H), 4.78 (d, *J* = 10.0 Hz, 1H), 3.97 (t, *J* = 6.7 Hz, 2H), 3.80 – 3.75 (m, 3H). ^{13}C NMR (500 MHz, $CDCl_3$) δ 168.0, 166.2, 165.6, 133.6, 133.5, 129.9, 129.8, 129.8, 128.8, 128.7, 128.5, 128.5, 90.5, 71.4, 70.8, 69.2, 68.0, 53.1, 40.3. HRMS (ESI): *m/z*: calcd for $C_{23}H_{21}ClNaO_{10}$ [M + Na]: 515.0715; found: 515.0721.

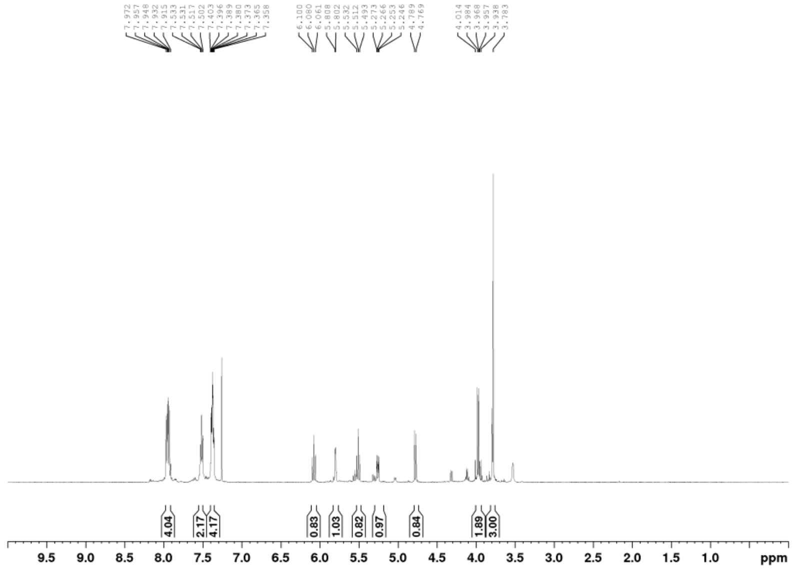

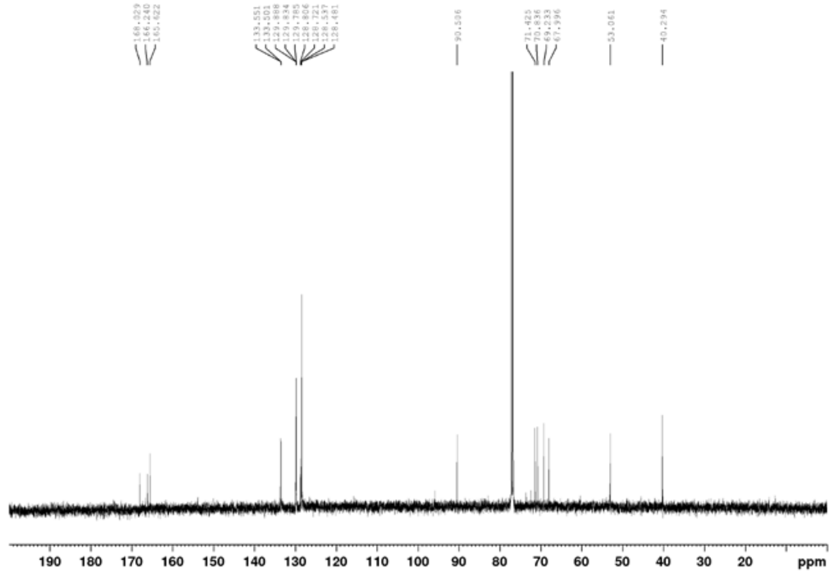

Compound 20

^{1}H NMR (500 MHz, $CDCl_3$) δ 8.12 – 7.95 (m, 2H), 7.59 (dd, *J* = 12.5, 4.3 Hz, 1H), 7.45 (q, *J* = 7.5 Hz, 2H), 7.28 – 7.17 (m, 5H), 5.67 (d, *J* = 3.4 Hz, 1H), 5.25 (d, *J* = 9.2 Hz, 1H), 5.14 (dd, *J* = 9.4, 3.3 Hz, 1H), 4.77 (d, *J* = 11.8 Hz, 1H), 4.65 – 4.56 (m, 2H), 4.27 (q, *J* = 9.0 Hz, 1H), 4.13 – 4.04 (m, 1H), 3.90 – 3.86 (m, 1H), 3.71 (s, 3H). 13C NMR (500 MHz, CDCl3) δ 168.6, 166.2, 165.7, 137.6, 133.7, 133.5, 129.8, 129.8, 128.5, 128.4, 127.9, 127.9, 127.8, 90.2, 75.9, 75.0, 72.9, 72.0, 68.3, 52.9, 40.3. HRMS (ESI): m/z: calcd for C23H23ClNaO9 [M + Na]: 501.0923; found: 501.0922.

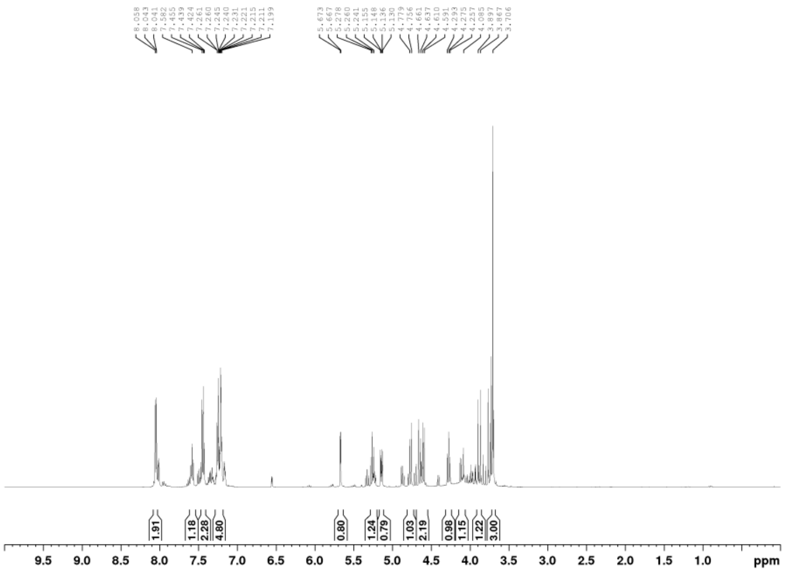

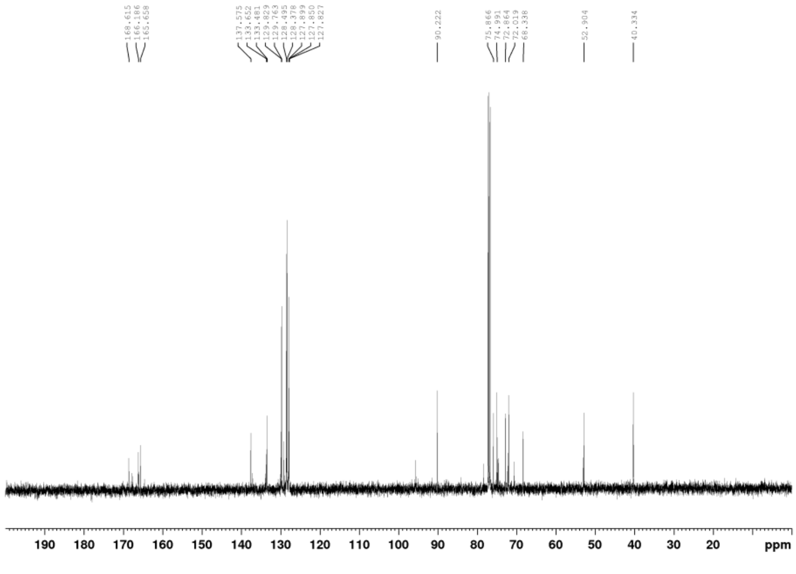

Donor D1

^{1}H NMR (500 MHz, $CDCl_3$) δ 8.62 (s, 1H), 8.00 (d, *J* = 7.6 Hz, 2H), 7.59 (t, *J* = 7.5 Hz, 1H), 7.44 (t, *J* = 7.8 Hz, 2H), 7.40 – 7.29 (m, 5H), 7.29 – 7.17 (m, 5H), 6.71 (d, *J* = 3.6 Hz, 1H), 5.46 (dd, *J* = 9.9, 3.5 Hz, 1H), 4.88 (dt, *J* = 20.0, 8.5 Hz, 3H), 4.70 (d, *J* = 10.7 Hz, 1H), 4.55 (d, *J* = 10.1 Hz, 1H), 4.34 (t, *J* = 9.5 Hz, 1H), 4.07 (t, *J* = 9.7 Hz, 1H), 3.77 (s, 3H). ^{13}C NMR (500 MHz, $CDCl_3$) δ 168.5, 165.2, 160.1, 137.4, 137.2, 133.3, 129.6, 128.9, 128.4, 128.3, 128.2, 128.1, 128.0, 127.7, 93.4, 90.6, 78.9, 78.5, 75.4, 75.4, 72.6, 71.9, 52.5. HRMS (ESI): *m/z*: calcd for $C_{30}H_{28}Cl_3NNaO_8$ [M + Na]: 658.0773; found: 658.0775.

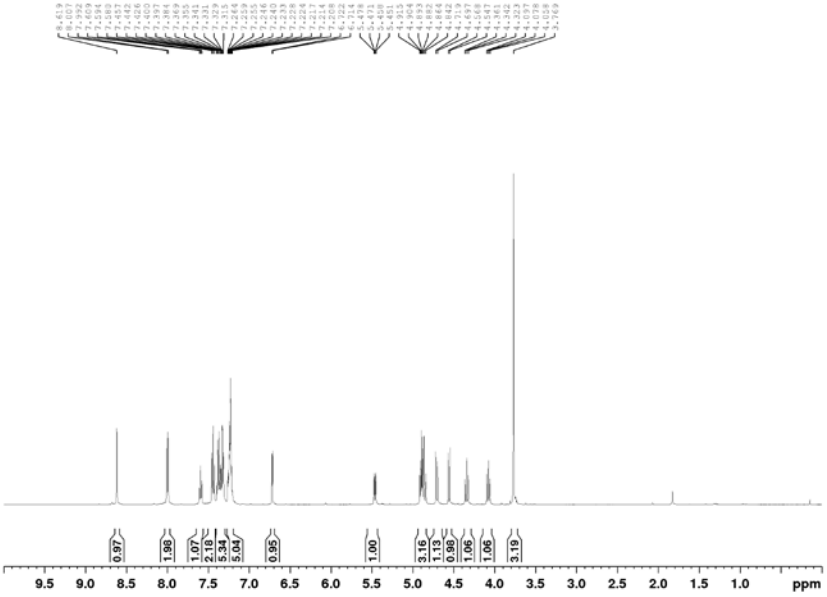

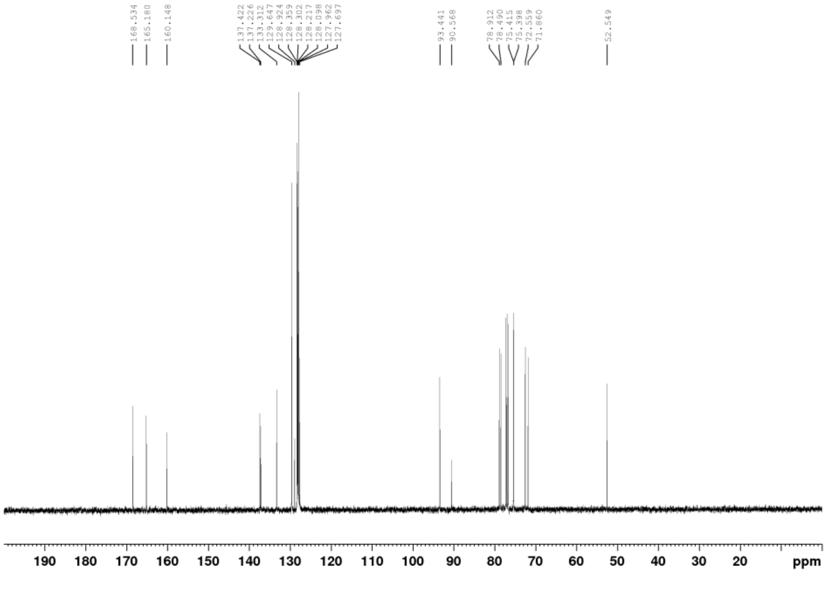

Donor D2

^{1}H NMR (500 MHz, $CDCl_3$) δ 8.63 (s, 1H), 8.00 – 7.89 (m, 4H), 7.57 – 7.47 (m, 2H), 7.40 (t, *J* = 7.8 Hz, 2H), 7.34 (d, *J* = 7.8 Hz, 2H), 7.17 (dd, *J* = 6.5, 3.5 Hz, 3H), 7.12 (dd, *J* = 6.5, 3.0 Hz, 2H), 6.78 (d, *J* = 3.5 Hz, 1H), 6.15 (t, *J* = 9.8 Hz, 1H), 5.49 (dd, *J* = 10.2, 3.7 Hz, 1H), 4.68 – 4.54 (m, 3H), 4.25 (t, *J* = 9.7 Hz, 1H), 3.78 (s, 3H). ^{13}C NMR (500 MHz, $CDCl_3$) δ 168.5, 165.4, 165.0, 160.4, 136.7, 133.5, 133.3, 129.8, 129.7, 129.2, 128.4, 128.4, 128.3, 128.2, 128.0, 93.1, 90.5, 77.1, 75.0, 72.4, 71.4, 70.4, 52.8. HRMS (ESI): *m/z*: calcd for $C_{30}H_{26}Cl_3NNaO_9$ [M + Na]: 672.0565; found: 672.0558.

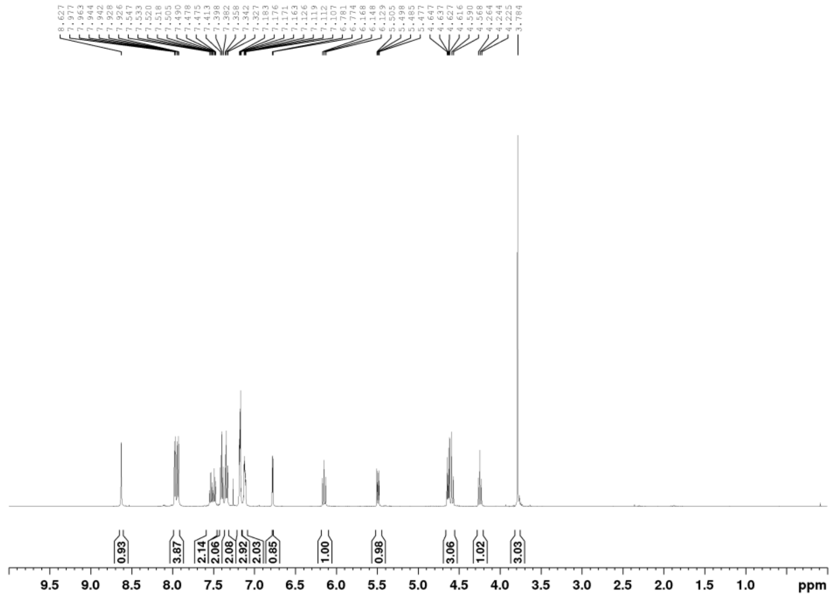

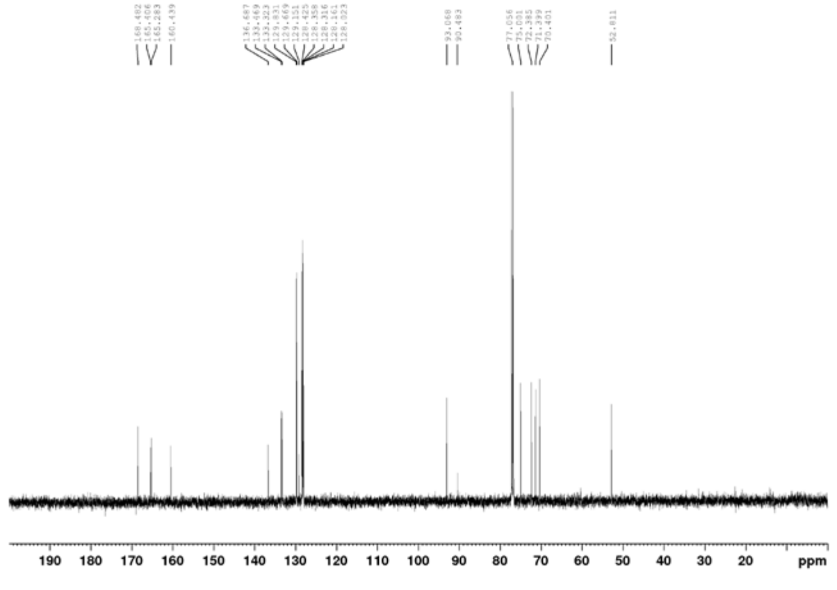

Donor D3

^{1}H NMR (500 MHz, $CDCl_3$) δ 8.61 (s, 1H), 7.95 (dd, *J* = 8.1, 1.0 Hz, 2H), 7.56 (d, *J* = 7.3 Hz, 1H), 7.41 (t, *J* = 7.8 Hz, 2H), 7.25 – 7.21 (m, 3H), 7.18 – 7.14 (m, 2H), 6.71 (d, *J* = 3.6 Hz, 1H), 5.42 (dd, *J* = 9.9, 3.6 Hz, 1H), 5.38 – 5.31 (m, 1H), 4.69 (dd, *J* = 45.7, 11.7 Hz, 2H), 4.46 (d, *J* = 10.2 Hz, 1H), 4.29 (t, *J* = 9.6 Hz, 1H), 3.94 (s, 1H), 3.81 (d, *J* = 14.7 Hz, 1H), 3.72 (s, 3H). ^{13}C NMR (500 MHz, $CDCl_3$) δ 167.2, 166.0, 165.1, 160.0, 137.2, 133.6, 129.8, 128.8, 128.5, 128.4, 128.1, 128.0, 93.0, 90.6, 75.6, 75.1, 71.9, 71.6, 70.6, 53.1, 40.3. HRMS (ESI): *m/z*: calcd for $C_{25}H_{23}Cl_4NNaO_9$ [M + Na]: 644.0019; found: 644.0021.

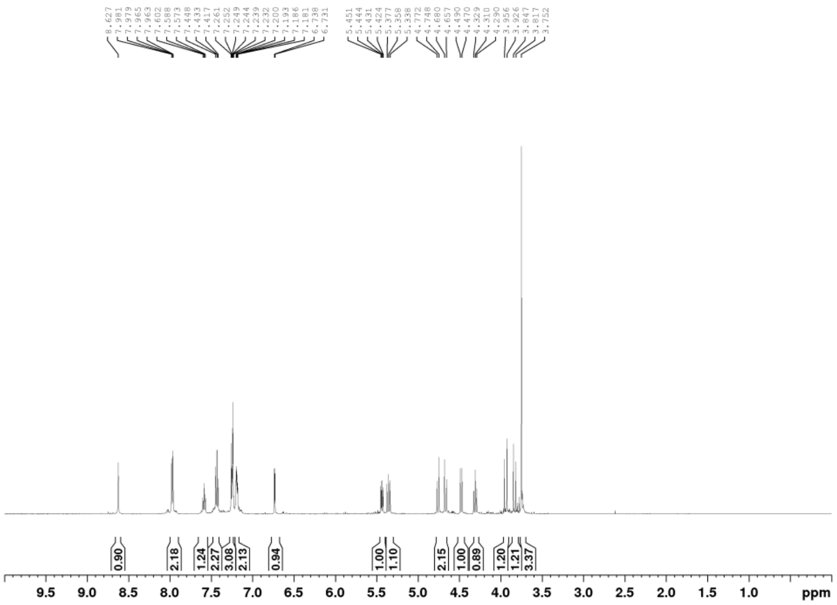

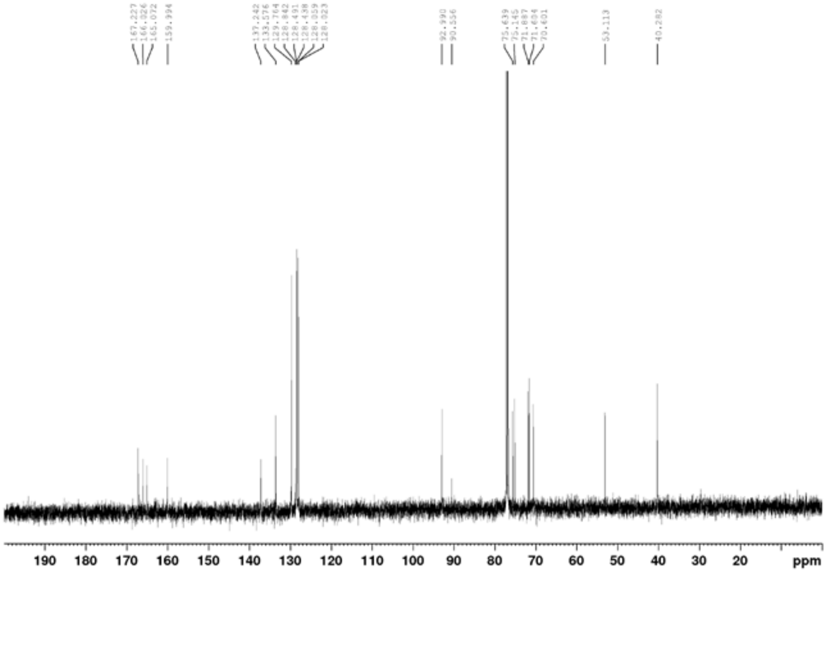

Donor D4

^{1}H NMR (500 MHz, $CDCl_3$) δ 8.69 (s, 1H), 7.96 – 7.89 (m, 4H), 7.55 – 7.48 (m, 2H), 7.37 (dt, *J* = 15.8, 7.7 Hz, 4H), 6.85 (d, *J* = 3.5 Hz, 1H), 6.13 (t, *J* = 10.0 Hz, 1H), 5.64 – 5.49 (m, 2H), 4.68 (d, *J* = 10.2 Hz, 1H), 3.99 (q, *J* = 14.9 Hz, 2H), 3.80 (s, 3H). ^{13}C NMR (500 MHz, $CDCl_3$) δ 166.9, 166.2, 165.5, 165.2, 160.2, 133.7, 129.9, 129.8, 128.5, 128.5, 128.4, 128.3, 92.7, 90.4, 70.4, 70.3, 70.0, 69.2, 53.2, 40.2. HRMS (ESI): *m/z*: calcd for $C_{25}H_{21}Cl_4NNaO_{10}$ [M + Na]: 657.9812; found: 657.9817.

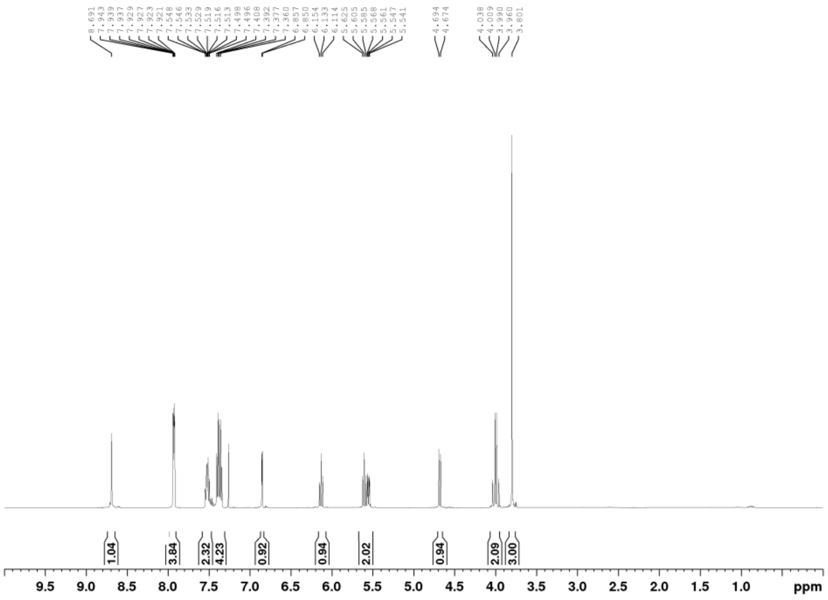

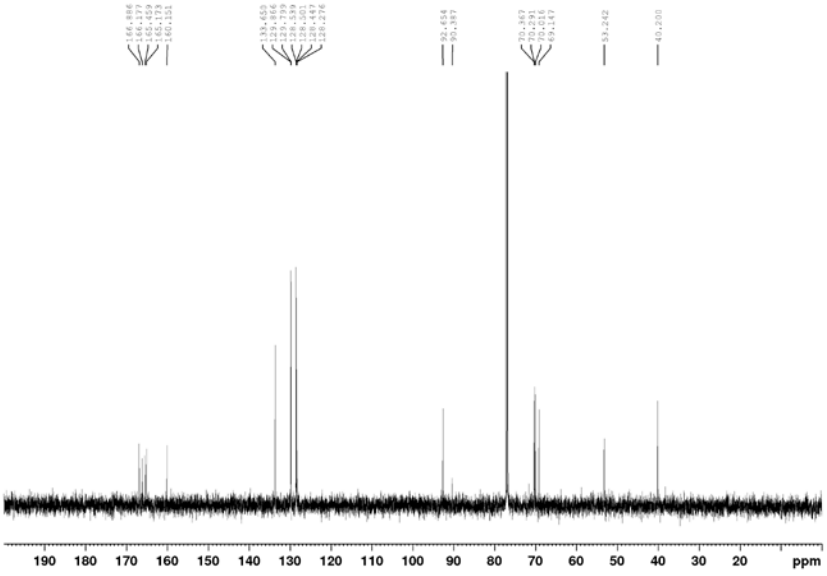

Compound 22

^{1}H NMR (500 MHz, $CDCl_3$) δ 6.40 (dd, *J* = 6.3, 1.7 Hz, 1H), 5.54 – 5.45 (m, 1H), 5.36 (dd, *J* = 4.4, 1.7 Hz, 1H), 4.66 (ddd, *J* = 6.3, 2.4, 1.3 Hz, 1H), 4.26 (d, *J* = 6.2 Hz, 1H), 4.18 (ddd, *J* = 17.0, 11.6, 6.3 Hz, 2H), 2.06 (s, 3H), 2.02 (s, 3H), 1.96 (s, 3H). ^{13}C NMR (500 MHz, $CDCl_3$) δ 170.3, 170.1, 169.9, 145.2, 98.7, 72.6, 63.7, 63.6, 61.7, 20.6, 20.5, 20.4. HRMS (ESI): *m/z*: calcd for $C_{12}H_{16}NaO_7$ [M + Na]: 295.0788; found: 295.0781.

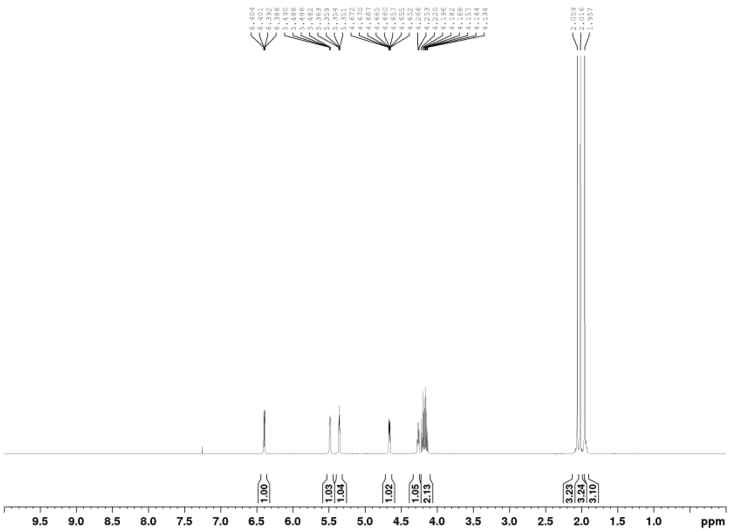

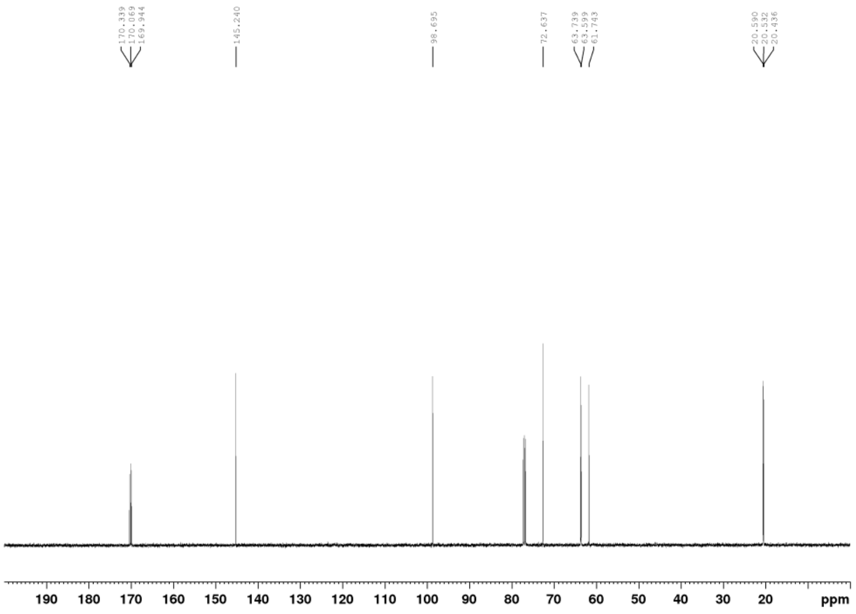

Compound 23

^{1}H NMR (500 MHz, $CDCl_3$) δ 6.28 (d, J = 4.1 Hz, 1H), 5.57 (d, J = 8.8 Hz, 1H), 5.41 (d, J = 2.4 Hz, 1H), 5.30 (d, J = 3.2 Hz, 1H), 5.15 (dd, J = 11.3, 3.2 Hz, 1H), 4.93 (dd, J = 10.6, 3.3 Hz, 1H), 4.30 (t, J = 6.5 Hz, 1H), 4.10 – 3.99 (m, 6H), 3.75 (dd, J = 10.5, 8.9 Hz, 1H), 2.08 (s, 6H), 1.97 (d, J = 1.2 Hz, 6H), 1.94 (d, J = 5.0 Hz, 6H). ^{13}C NMR (500 MHz, $CDCl_3$) δ 170.0, 170.0, 169.7, 169.5, 169.3, 169.2, 97.8, 96.8, 71.6, 71.4, 69.2, 68.3, 66.4, 65.7, 60.7, 60.7, 57.3, 55.7, 20.2, 20.2. HRMS (ESI): m/z: calcd for $C_{12}H_{16}N_4NaO_{10}$ [M + Na]: 399.0759; found: 399.0765.

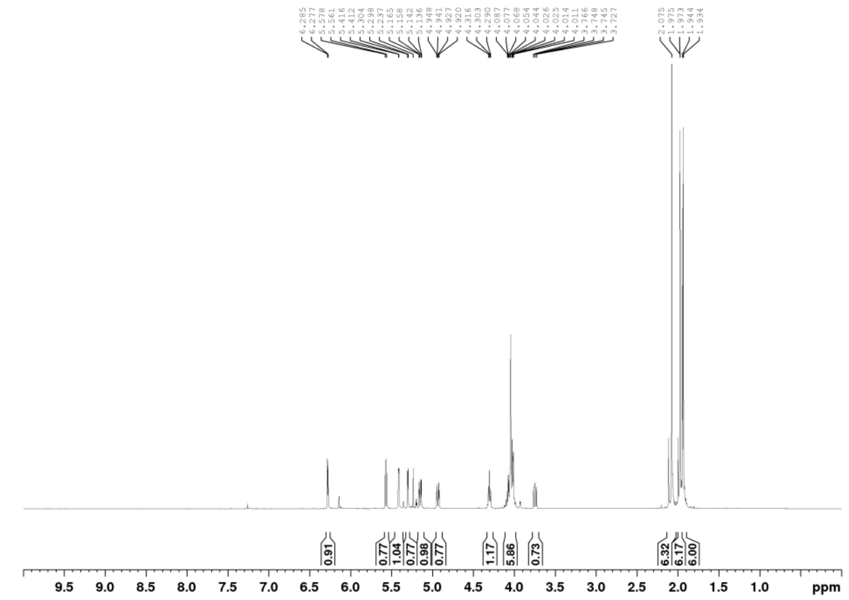

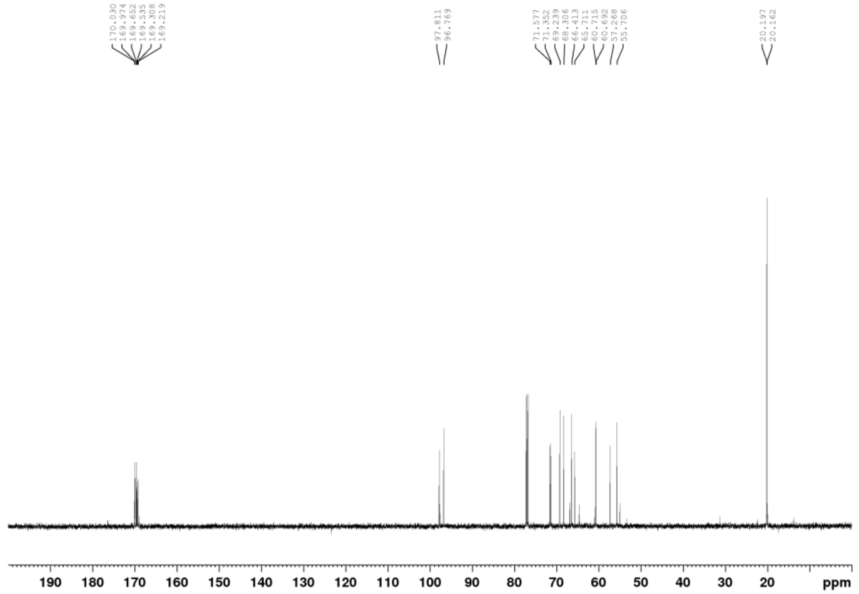

Compound 24

^{1}H NMR (500 MHz, $CDCl_3$) δ 7.59 (dd, *J* = 6.6, 2.9 Hz, 2H), 7.48 (dd, *J* = 7.5, 1.7 Hz, 2H), 7.36 – 7.25 (m, 6H), 5.67 (d, *J* = 5.4 Hz, 1H), 5.45 (d, *J* = 2.8 Hz, 1H), 5.32 (d, *J* = 3.0 Hz, 1H), 5.15 (dd, *J* = 11.1, 3.2 Hz, 1H), 4.85 (dd, *J* = 10.3, 3.2 Hz, 1H), 4.72 (d, *J* = 6.5 Hz, 1H), 4.51 (d, *J* = 10.1 Hz, 1H), 4.29 (dd, *J* = 11.1, 5.5 Hz, 1H), 4.18 – 4.11 (m, 1H), 4.11 – 4.02 (m, 3H), 3.87 (d, *J* = 6.6 Hz, 1H), 3.62 (t, *J* = 10.2 Hz, 1H), 2.12 (s, 3H), 2.04 (d, *J* = 10.5 Hz, 6H), 2.00 (d, *J* = 5.4 Hz, 6H), 1.95 (s, 3H). ^{13}C NMR (500 MHz, $CDCl_3$) δ 170.2, 170.2, 169.8, 169.8, 169.5, 169.4, 133.4, 132.4, 132.3, 130.9, 129.0, 128.8, 128.4, 127.9, 86.7, 86.2, 74.2, 72.8, 70.0, 67.4, 67.3, 66.4, 61.5, 61.4, 59.2, 57.9, 20.5, 20.4, 20.4. HRMS (ESI): *m/z*: calcd for $C_{18}H_{21}N_3NaO_7S$ [M + Na]: 446.0992; found: 446.0999.

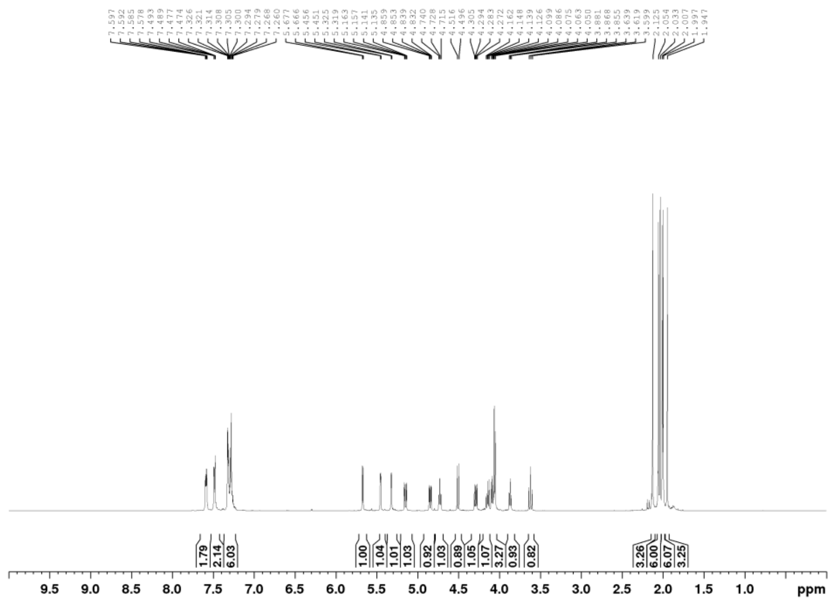

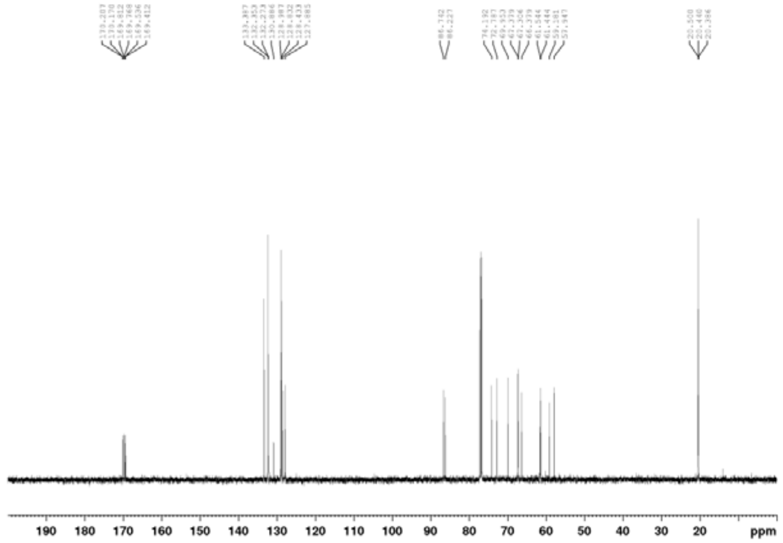

Compound 25

^{1}H NMR (500 MHz, $CDCl_3$) δ 7.53 – 7.45 (m, 4H), 7.42 – 7.38 (m, 3H), 7.32 – 7.24 (m, 3H), 5.75 (d, *J* = 5.2 Hz, 1H), 5.58 (s, 1H), 4.27 (d, *J* = 3.6 Hz, 1H), 4.19 (dd, *J* = 11.2, 5.9 Hz, 3H), 4.08 (dd, *J* = 12.9, 1.8 Hz, 1H), 4.00 (dd, *J* = 10.1, 2.4 Hz, 1H). ^{13}C NMR (500 MHz, $CDCl_3$) δ 137.1, 133.5, 131.0, 129.3, 129.0, 128.2, 127.3, 126.1, 101.2, 87.1, 75.0, 69.4, 69.0, 63.5, 61.1. HRMS (ESI): *m/z*: calcd for $C_{19}H_{19}N_3NaO_4S$ [M + Na]: 408.0988; found: 408.0996.

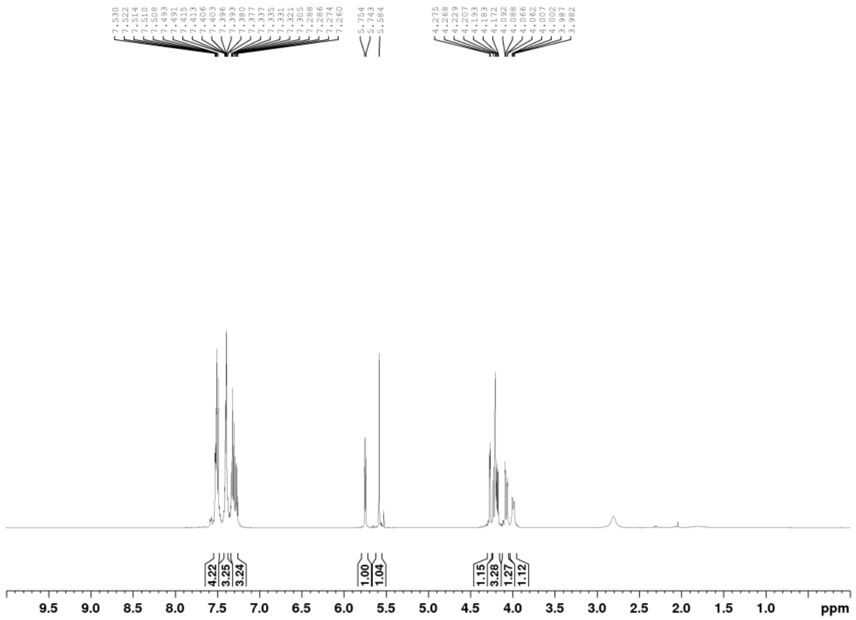

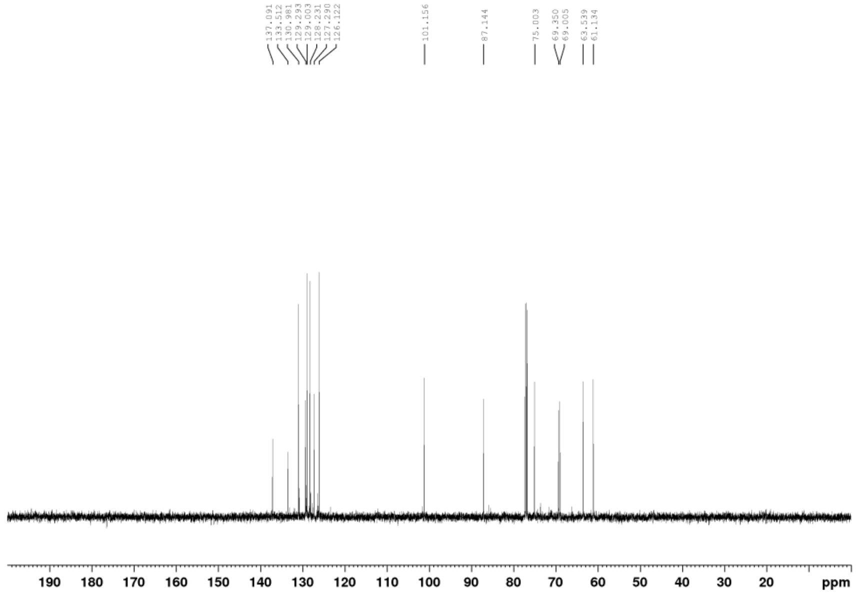

Compound 26

^{1}H NMR (500 MHz, $CDCl_3$) δ 7.85 (ddd, *J* = 10.6, 9.4, 7.8 Hz, 4H), 7.62 – 7.45 (m, 7H), 7.40 (q, *J* = 5.5 Hz, 3H), 7.32 – 7.25 (m, 3H), 5.80 (d, *J* = 5.3 Hz, 1H), 5.50 (s, 1H), 4.95 (dd, *J* = 31.0, 12.2 Hz, 2H), 4.55 (dd, *J* = 10.6, 5.4 Hz, 1H), 4.27 (d, *J* = 3.1 Hz, 1H), 4.19 (dd, *J* = 12.6, 1.0 Hz, 1H), 4.11 (s, 1H), 4.02 (dd, *J* = 12.6, 1.4 Hz, 1H), 3.92 (dd, *J* = 10.6, 3.4 Hz, 1H). ^{13}C NMR (500 MHz, $CDCl_3$) δ 137.5, 135.1, 133.6, 133.2, 133.1, 131.0, 129.0, 128.2, 128.2, 127.9, 127.7, 127.3, 126.4, 126.2, 126.0, 125.6, 100.9, 87.5, 76.5, 72.9, 71.5, 69.2, 63.7, 59.3. HRMS (ESI): *m/z*: calcd for $C_{30}H_{27}N_3NaO_4S$ [M + Na]: 548.1614; found: 548.1615.

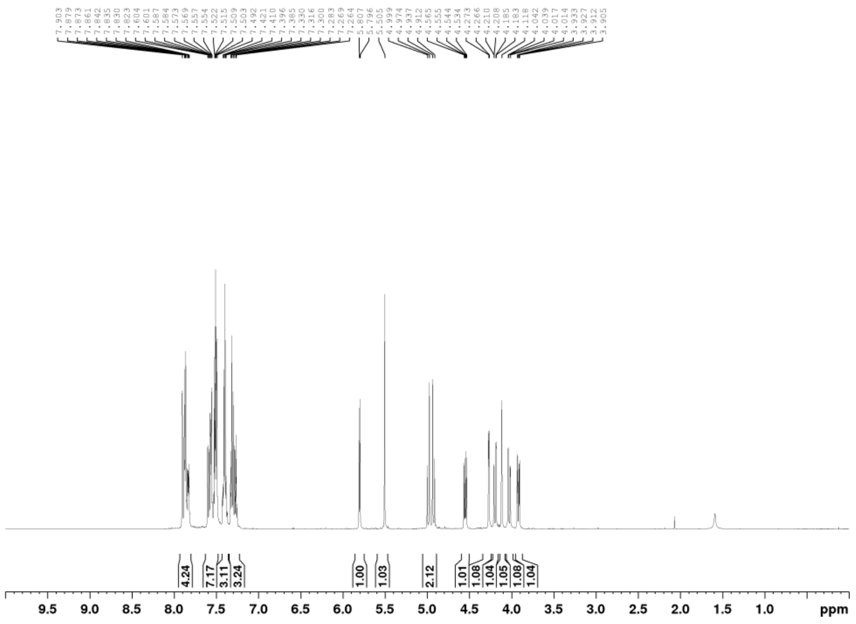

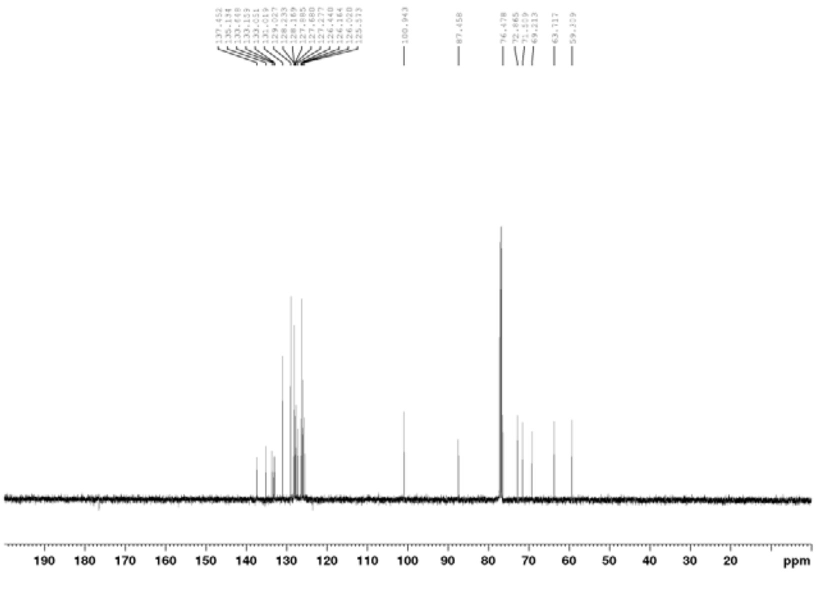

Compound 27

^{1}H NMR (500 MHz, $CDCl_3$) δ 7.88 (dd, *J* = 9.7, 6.7 Hz, 4H), 7.59 – 7.44 (m, 5H), 7.39 – 7.28 (m, 5H), 7.26 (dd, *J* = 5.0, 2.0 Hz, 3H), 5.64 (d, *J* = 5.5 Hz, 1H), 4.91 (d, *J* = 4.1 Hz, 2H), 4.56 – 4.50 (m, 3H), 4.36 (dd, *J* = 10.4, 5.5 Hz, 1H), 4.21 (d, *J* = 2.3 Hz, 1H), 3.84 – 3.76 (m, 2H), 3.73 (dd, *J* = 10.3, 5.9 Hz, 1H). ^{13}C NMR (500 MHz, $CDCl_3$) δ 137.7, 134.4, 133.2, 133.2, 133.2, 133.1, 132.4, 129.0, 128.5, 128.4, 127.9, 127.7, 127.6, 127.6, 126.9, 126.3, 126.2, 125.6, 87.4, 77.8, 73.5, 72.1, 69.7, 69.5, 66.7, 59.6. HRMS (ESI): *m/z*: calcd for $C_{30}H_{29}N_3NaO_4S$ [M + Na]: 550.1771; found: 550.1777.

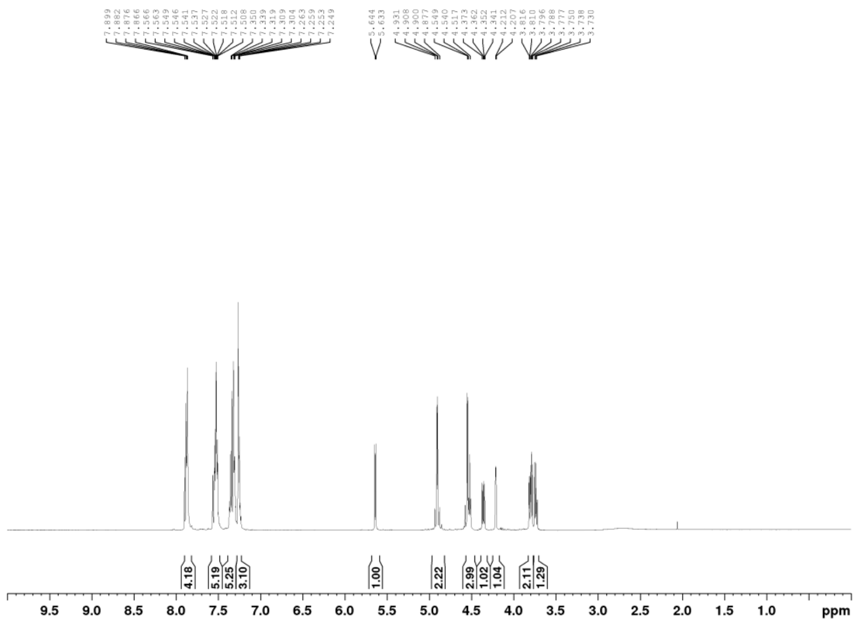

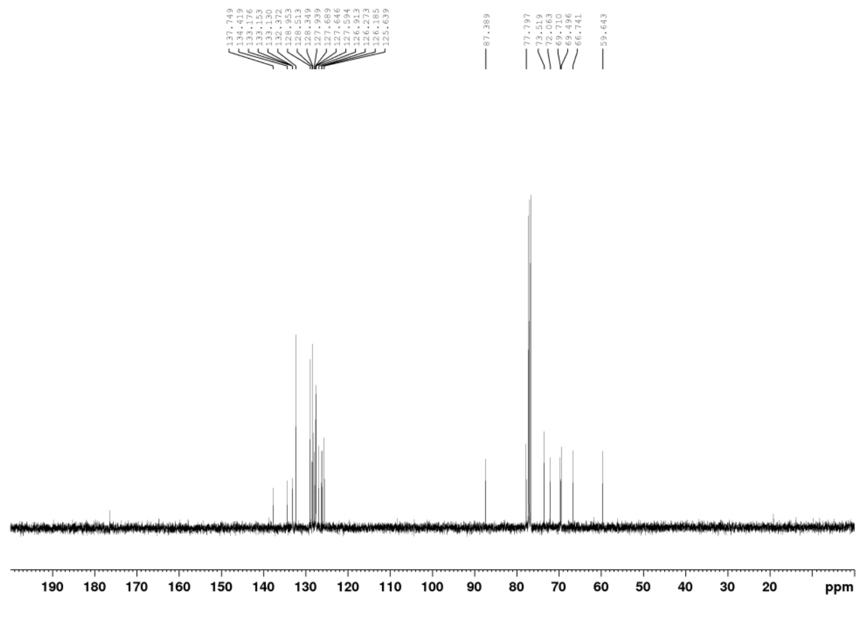

Compound 27

2D COSY

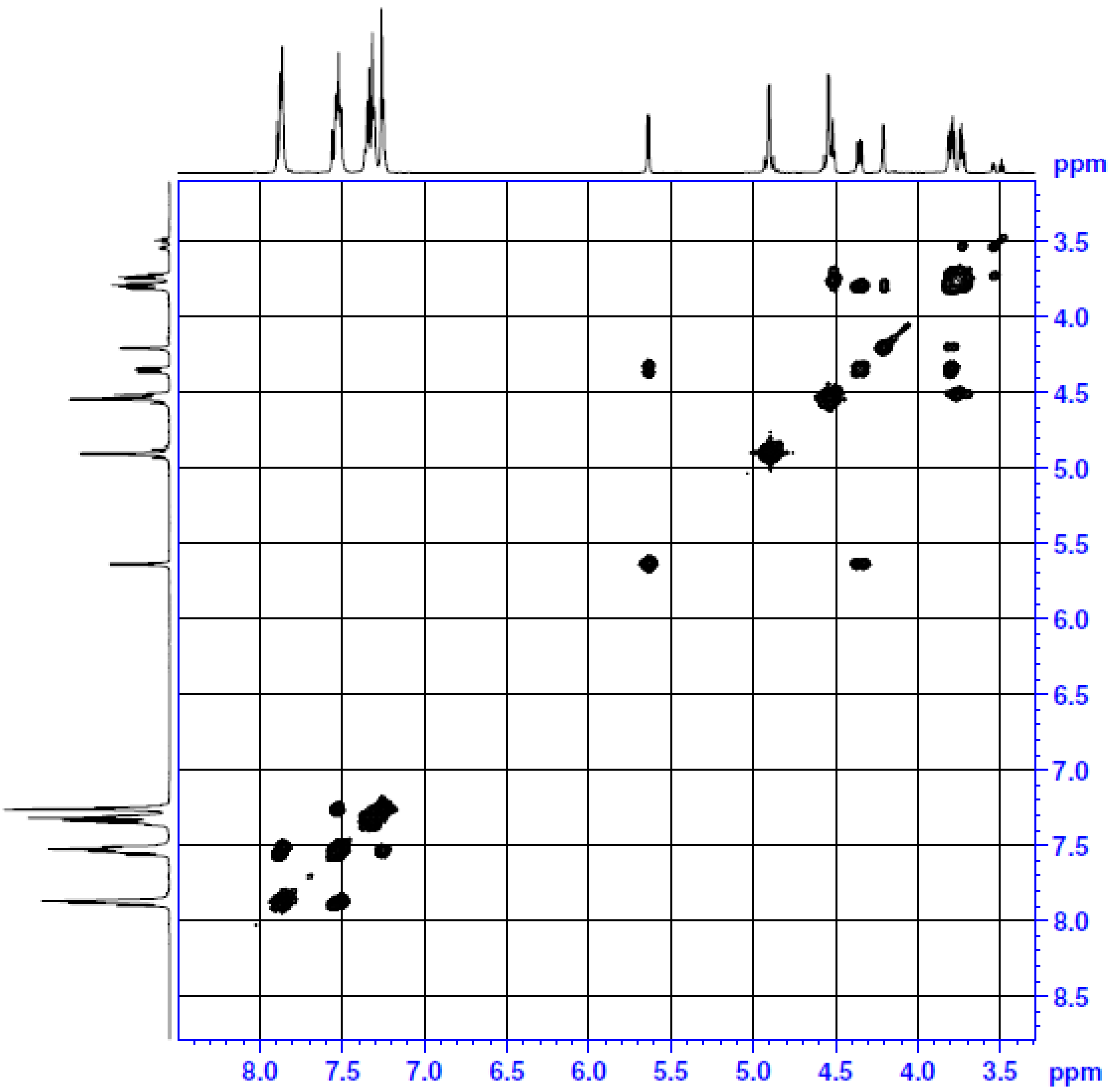

Compound 27

2D HMBC

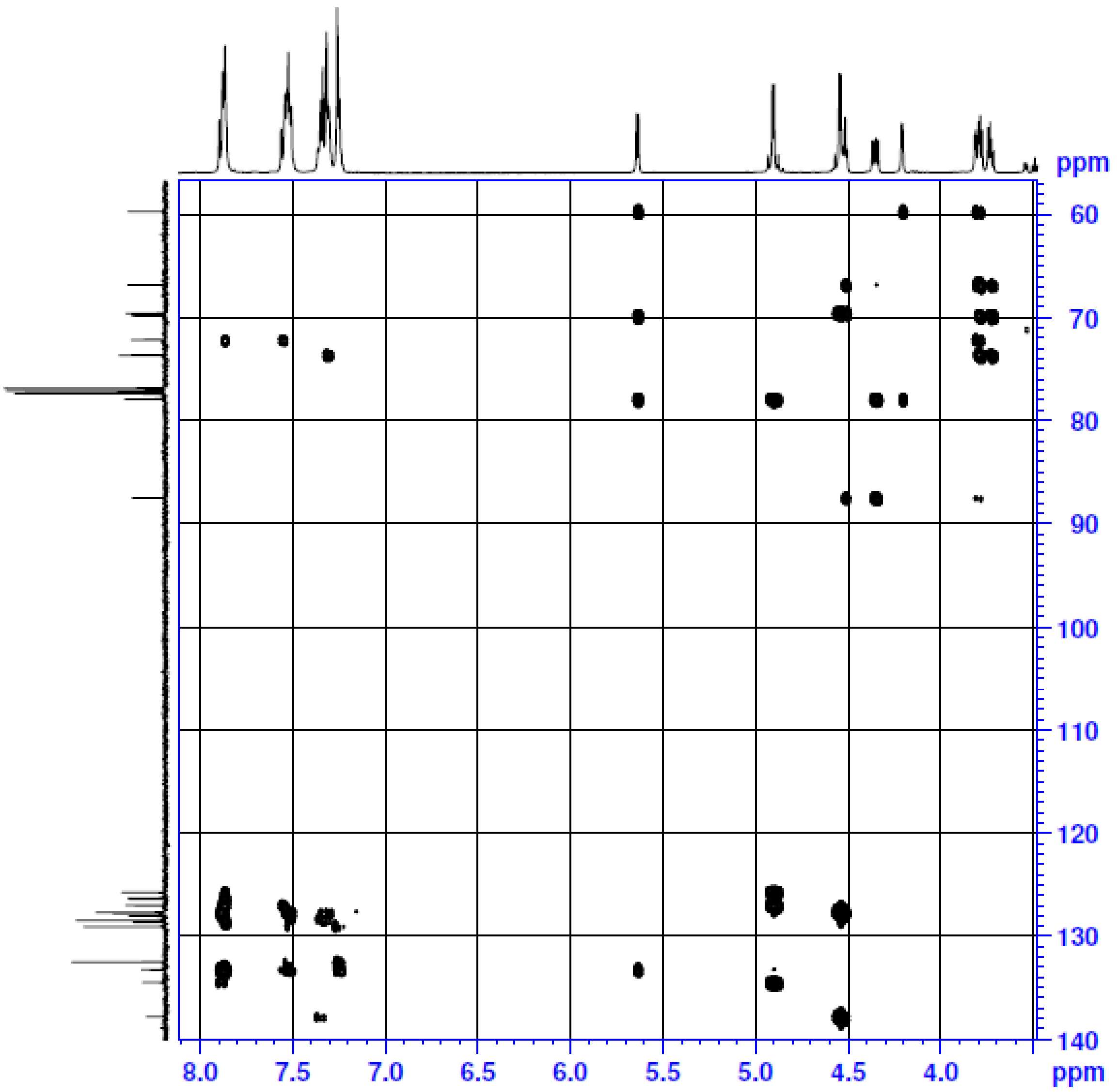

Compound 27

2D HSQC

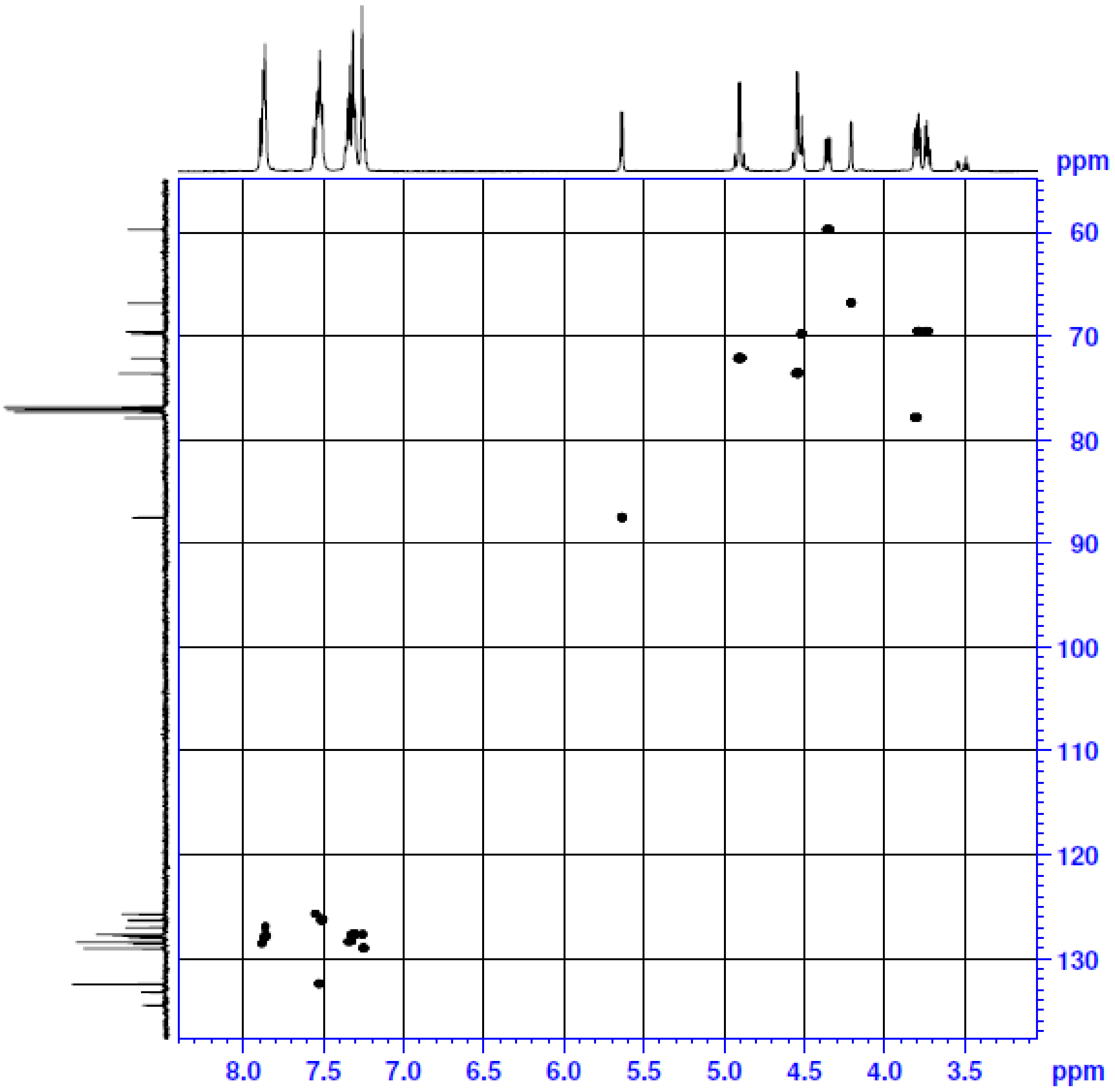

Compound 29

^{1}H NMR (500 MHz, $CDCl_3$) δ 7.94 – 7.83 (m, 4H), 7.58 (dd, *J* = 8.5, 1.3 Hz, 1H), 7.57 – 7.49 (m, 4H), 7.36 – 7.25 (m, 8H), 5.70 (d, *J* = 5.4 Hz, 1H), 5.02 – 4.89 (m, 3H), 4.63 (d, *J* = 11.5 Hz, 1H), 4.51 (dd, *J* = 10.6, 5.5 Hz, 1H), 4.29 (t, *J* = 6.0 Hz, 1H), 4.00 (s, 1H), 3.88 (dd, *J* = 10.5, 2.7 Hz, 1H), 3.75 (dd, *J* = 11.4, 6.8 Hz, 1H), 3.55 (dd, *J* = 11.4, 5.2 Hz, 1H). ^{13}C NMR (500 MHz, $CDCl_3$) δ 137.7, 134.7, 133.2, 133.1, 132.4, 129.0, 128.5, 128.4, 128.2, 128.0, 127.9, 127.7, 127.7, 126.7, 126.2, 126.1, 125.6, 87.3, 79.2, 74.6, 73.3, 72.8, 71.9, 62.0, 60.4. HRMS (ESI): *m/z*: calcd for $C_{30}H_{29}N_3NaO_4S$ [M + Na]: 550.1771; found: 550.1780.

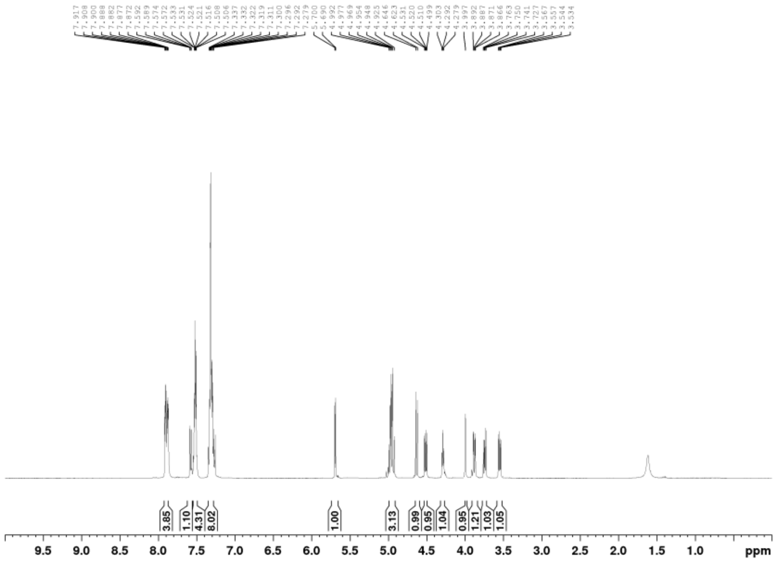

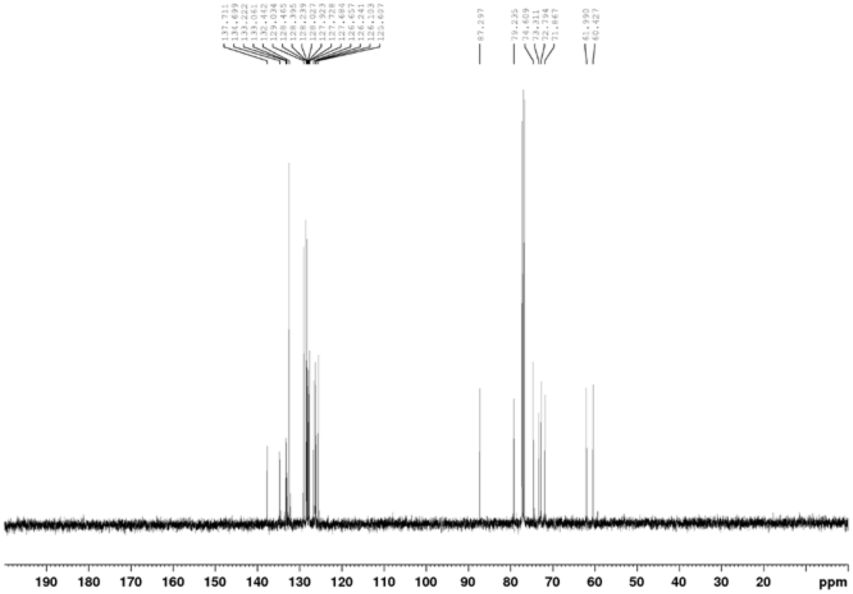

Compound 29

2D COSY

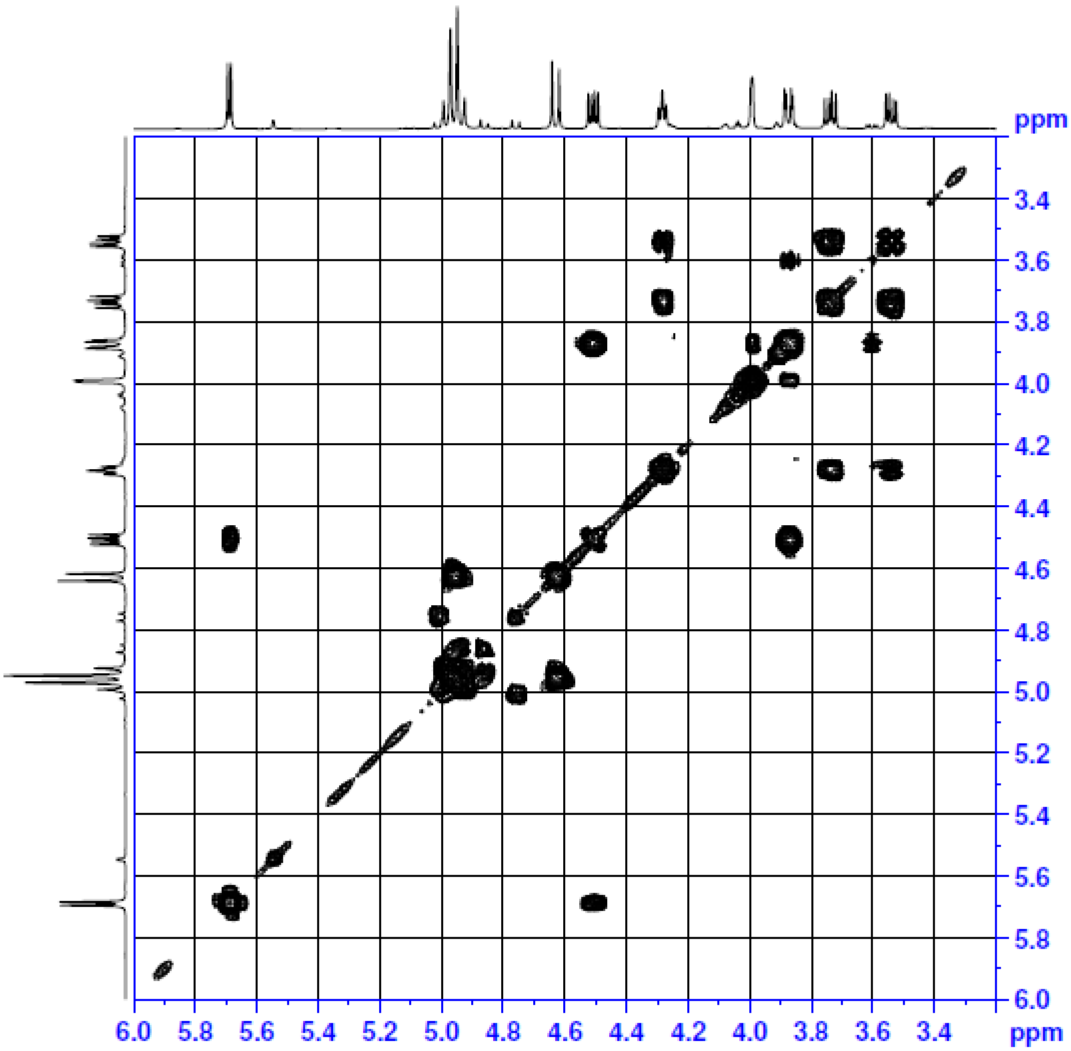

Compound 29

2D HMBC, 2D HSQC

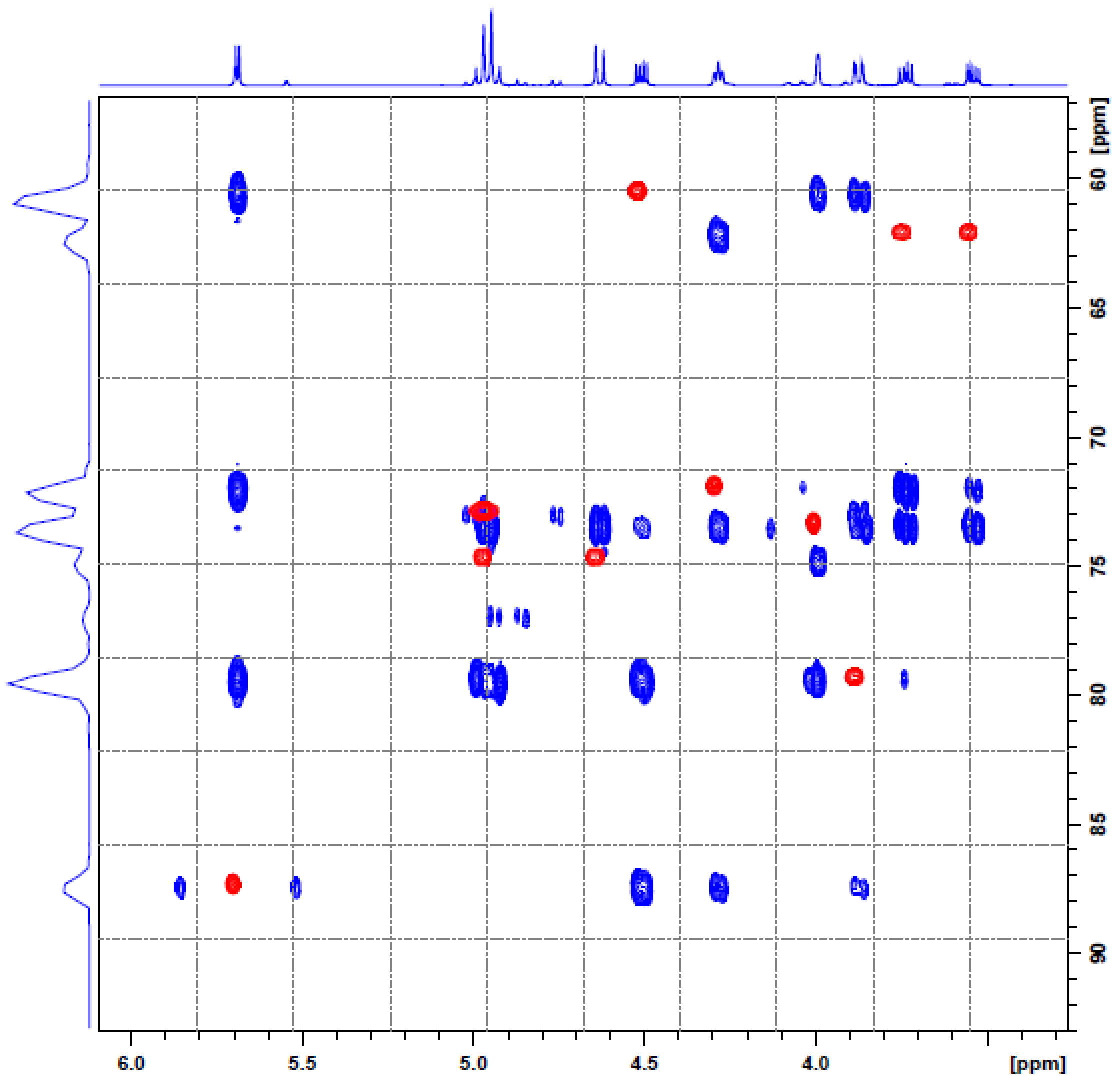

Compound 31

^{1}H NMR (500 MHz, $CDCl_3$) δ 7.96 – 7.77 (m, 4H), 7.62 – 7.41 (m, 5H), 7.38 – 7.22 (m, 3H), 5.64 (d, *J* = 5.4 Hz, 1H), 4.89 (dd, *J* = 31.2, 11.5 Hz, 2H), 4.31 (dd, *J* = 10.0, 5.6 Hz, 2H), 4.16 (s, 1H), 3.89 (dd, *J* = 11.8, 5.6 Hz, 1H), 3.87 – 3.68 (m, 2H). ^{13}C NMR (500 MHz, $CDCl_3$) δ 134.3, 133.2, 132.4, 129.1, 128.6, 127.9, 127.8, 127.7, 127.0, 126.4, 126.3, 125.6, 87.2, 77.6, 72.3, 70.5, 67.6, 62.8, 59.6. HRMS (ESI): *m/z*: calcd for $C_{23}H_{23}N_3NaO_4S$ [M + Na]: 460.1301; found: 460.1300.

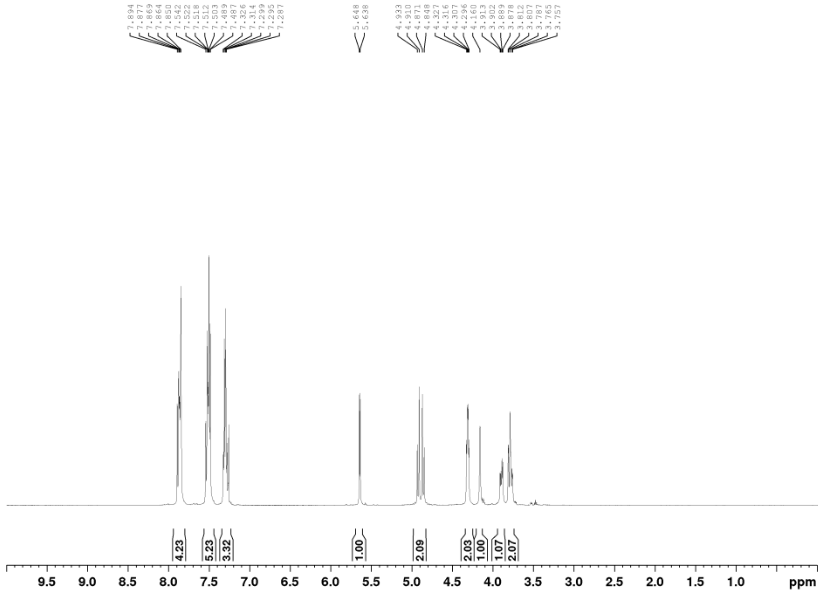

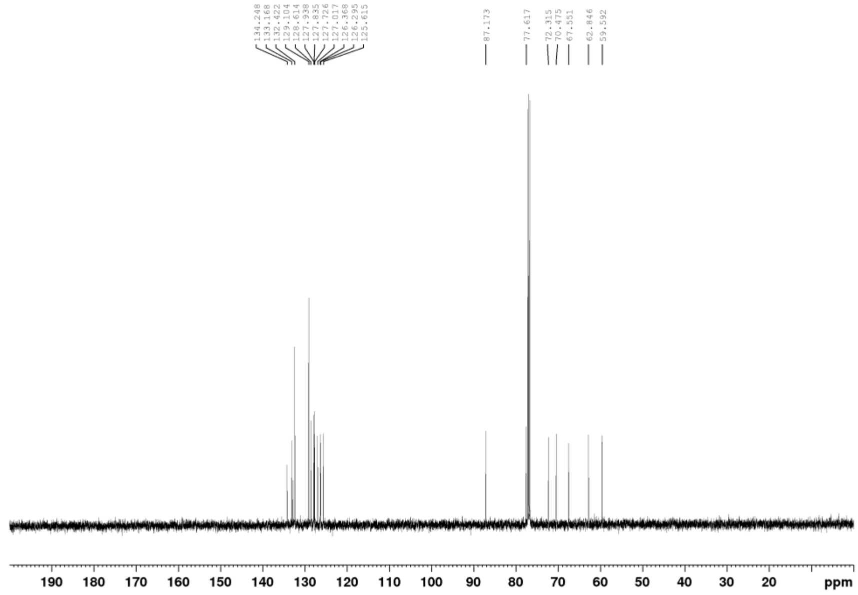

Compound 32

^{1}H NMR (500 MHz, $CDCl_3$) δ 7.93 – 7.85 (m, 4H), 7.59 – 7.48 (m, 5H), 7.39 – 7.24 (m, 13H), 5.67 (d, *J* = 5.5 Hz, 1H), 5.00 – 4.92 (m, 3H), 4.62 (d, *J* = 11.3 Hz, 1H), 4.56 – 4.50 (m, 2H), 4.50 – 4.42 (m, 2H), 4.12 (d, *J* = 1.7 Hz, 1H), 3.88 (dd, *J* = 10.6, 2.7 Hz, 1H), 3.68 (dd, *J* = 9.4, 7.0 Hz, 1H), 3.59 (dd, *J* = 9.4, 6.1 Hz, 1H). ^{13}C NMR (500 MHz, $CDCl_3$) δ 138.2, 137.8, 134.8, 133.4, 133.2, 133.0, 132.2, 128.9, 128.4, 128.3, 128.3, 127.9, 127.7, 127.7, 127.5, 126.5, 126.2, 126.0, 125.6, 87.6, 79.2, 74.9, 73.5, 73.4, 72.5, 70.5, 68.6, 60.4. HRMS (ESI): *m/z*: calcd for $C_{37}H_{35}N_3NaO_4S$ [M + Na]: 640.2240; found: 640.2245.

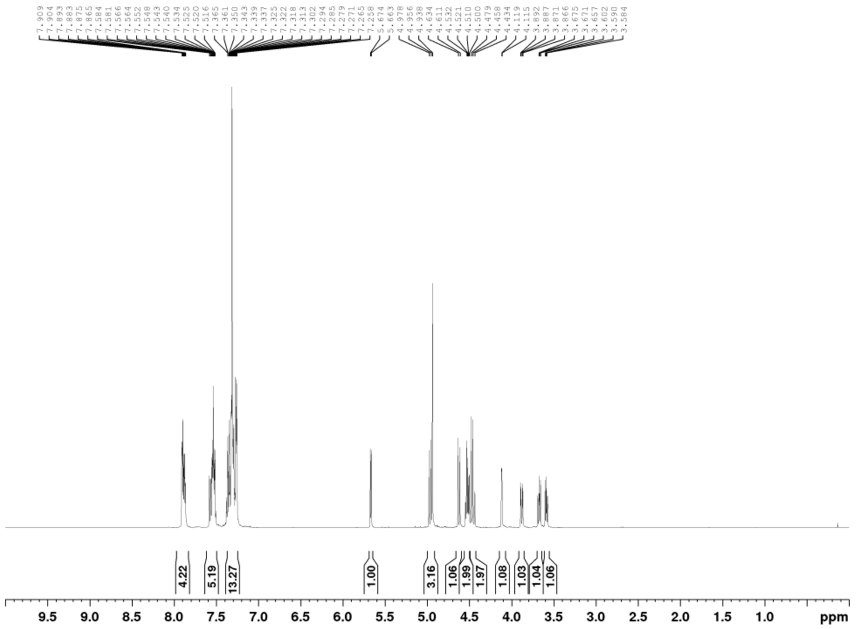

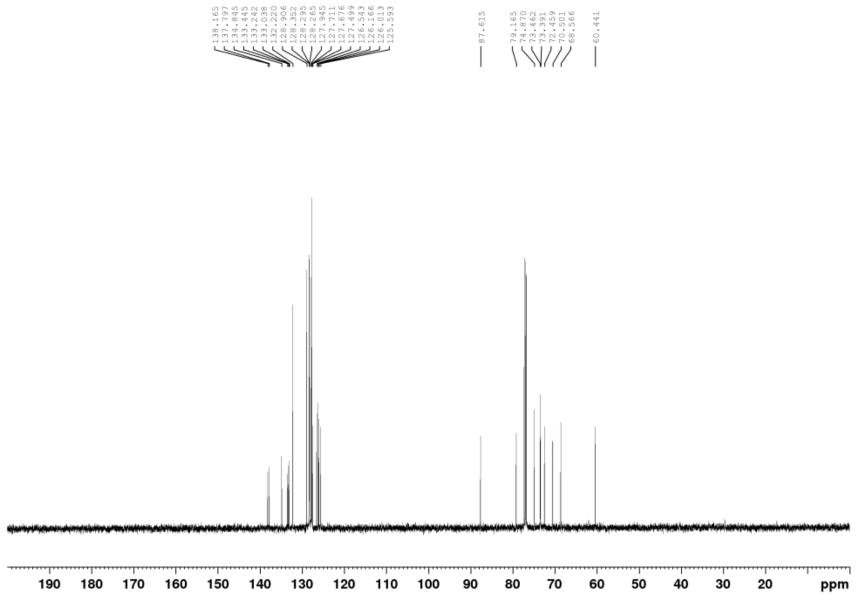

Compound 34

^{1}H NMR (500 MHz, $CDCl_3$) δ 7.84 (dd, *J* = 7.8, 3.9 Hz, 3H), 7.79 (s, 1H), 7.55 – 7.47 (m, 2H), 7.47 – 7.29 (m, 8H), 7.26 – 7.20 (m, 3H), 6.75 (d, *J* = 7.0 Hz, 1H), 5.83 (d, *J* = 5.3 Hz, 1H), 5.79 (d, *J* = 2.1 Hz, 1H), 4.94 (d, *J* = 12.1 Hz, 1H), 4.67 (d, *J* = 6.4 Hz, 1H), 4.64 – 4.55 (m, 3H), 4.49 (d, *J* = 11.7 Hz, 1H), 3.73 (dd, *J* = 11.3, 3.0 Hz, 1H), 3.60 (d, *J* = 6.4 Hz, 2H), 2.13 (s, 3H). ^{13}C NMR (500 MHz, $CDCl_3$) δ 170.3, 161.7, 137.6, 134.3, 133.2, 133.2, 132.8, 132.6, 129.1, 128.7, 128.5, 128.1, 128.0, 127.9, 127.7, 127.2, 126.4, 126.2, 125.7, 92.2, 88.4, 73.7, 73.6, 71.0, 69.3, 68.1, 65.8, 51.6, 20.8. HRMS (ESI): *m/z*: calcd for $C_{34}H_{32}Cl_3NNaO_6S$ [M + Na]: 710.0908; found: 710.0911.

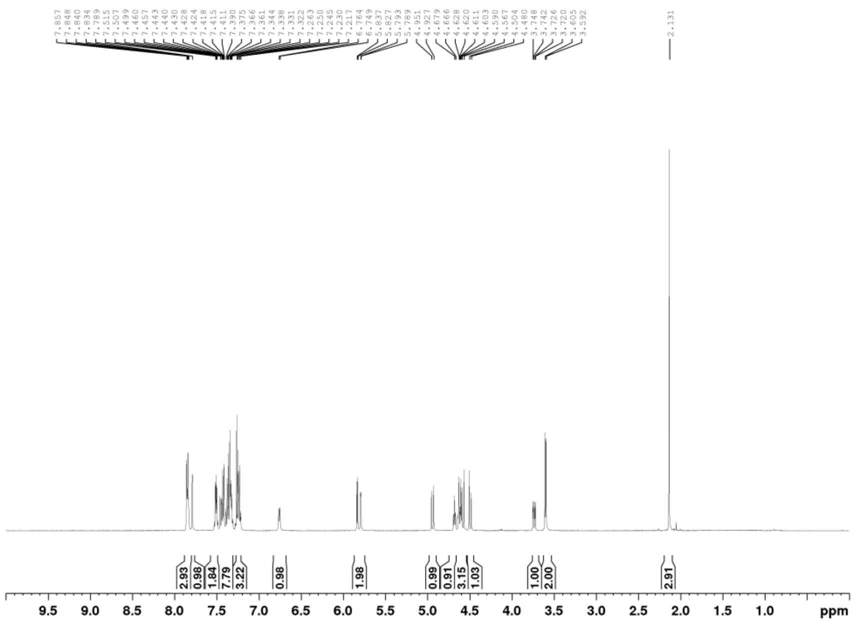

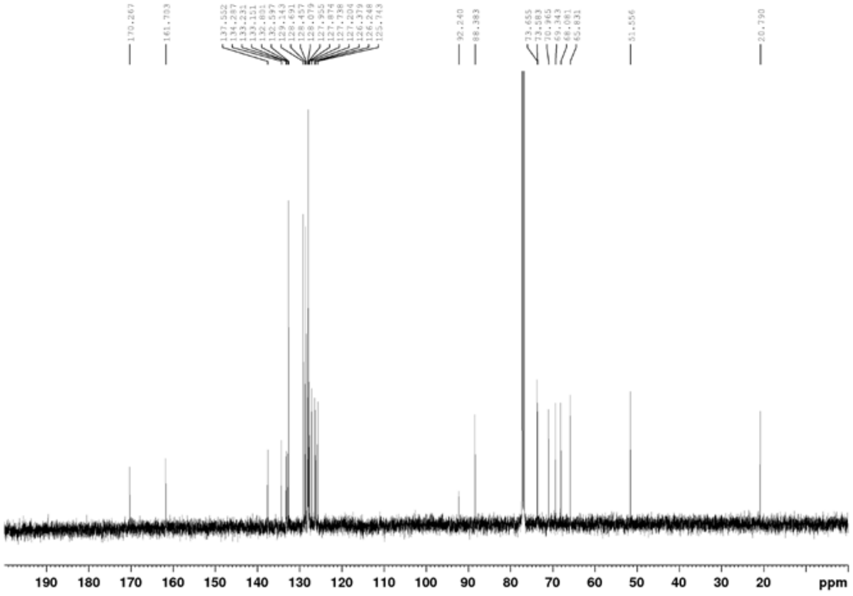

Compound 35

^{1}H NMR (500 MHz, $CDCl_3$) δ 7.94 – 7.82 (m, 4H), 7.58 – 7.48 (m, 3H), 7.45 (dd, *J* = 6.7, 2.9 Hz, 2H), δ 7.38 – 7.26 (m, 8H), 6.86 (d, *J* = 7.4 Hz, 1H), 5.86 (d, *J* = 5.1 Hz, 1H), 5.00 (d, *J* = 11.5 Hz, 1H), 4.96 – 4.88 (m, 2H), 4.75 (d, *J* = 12.0 Hz, 1H), 4.67 (d, *J* = 11.6 Hz, 1H), 4.52 – 4.46 (m, 1H), 4.29 (dd, *J* = 11.3, 7.4 Hz, 1H), 4.22 (dd, *J* = 11.4, 5.0 Hz, 1H), 4.10 (s, 1H), 3.80 (dd, *J* = 11.1, 2.3 Hz, 1H), 2.00 (s, 3H). ^{13}C NMR (500 MHz, $CDCl_3$) δ 170.5, 161.5, 137.6, 134.2, 133.2, 133.1, 132.8, 132.3, 129.0, 128.7, 128.4, 128.2, 127.9, 127.9, 127.7, 126.7, 126.4, 126.3, 125.4, 92.3, 88.2, 77.1, 74.4, 71.8, 71.7, 70.1, 63.2, 51.4, 20.7. HRMS (ESI): *m/z*: calcd for $C_{34}H_{32}Cl_3NNaO_6S$ [M + Na]: 710.0908; found: 710.0906.

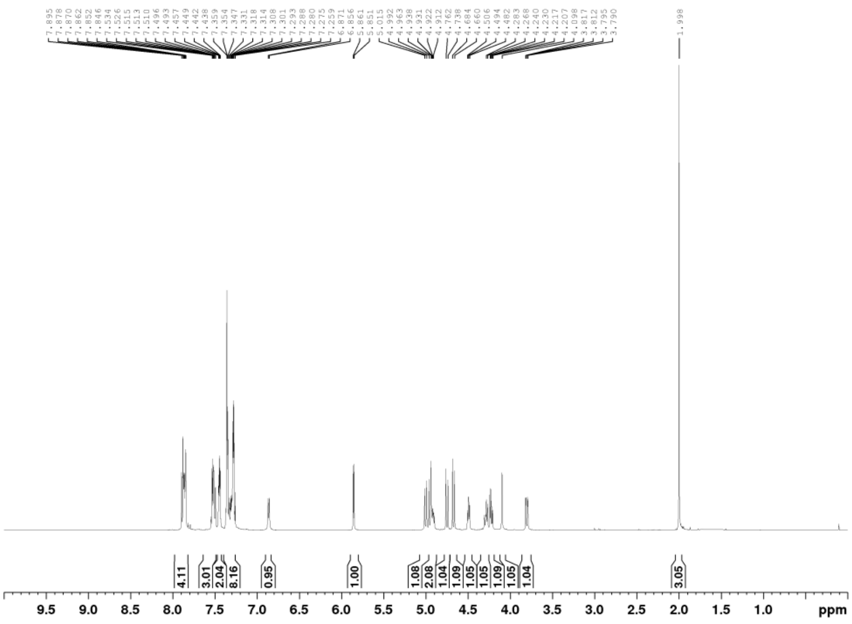

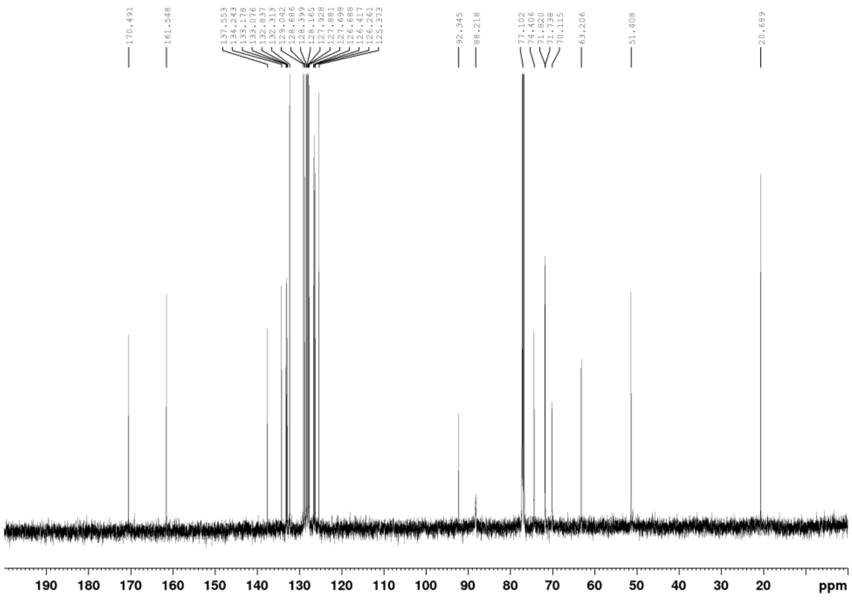

Compound 36

^{1}H NMR (500 MHz, $CDCl_3$) δ 7.90 – 7.80 (m, 4H), 7.54 – 7.44 (m, 5H), 7.40 – 7.21 (m, 13H), 6.87 (d, *J* = 7.3 Hz, 1H), 5.85 (d, *J* = 5.1 Hz, 1H), 4.99 (d, *J* = 11.4 Hz, 1H), 4.96 – 4.85 (m, 2H), 4.68 (t, *J* = 11.3 Hz, 2H), 4.57 – 4.47 (m, 3H), 4.22 (s, 1H), 3.81 – 3.71 (m, 2H), 3.66 (dd, *J* = 9.3, 5.9 Hz, 1H). ^{13}C NMR (500 MHz, $CDCl_3$) δ 161.5, 138.1, 137.8, 134.5, 133.3, 133.2, 133.1, 132.3, 129.1, 128.6, 128.4, 128.3, 128.0, 127.9, 127.8, 127.8, 127.7, 126.6, 126.4, 126.2, 125.40, 92.4, 88.7, 77.1, 74.7, 73.5, 72.1, 71.3, 71.0, 68.5, 51.6. HRMS (ESI): *m/z*: calcd for $C_{39}H_{36}Cl_3NNaO_5S$ [M + Na]: 758.1272; found: 758.1281.

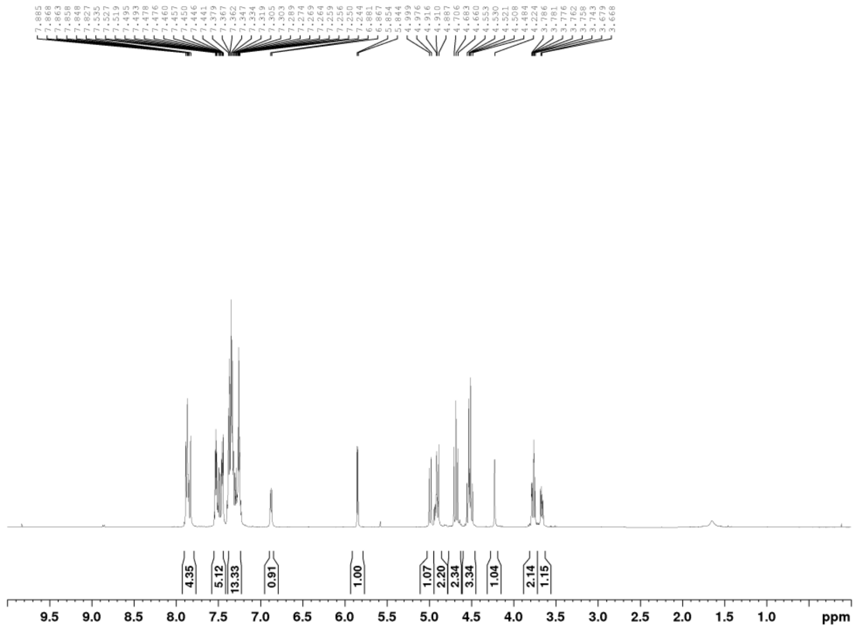

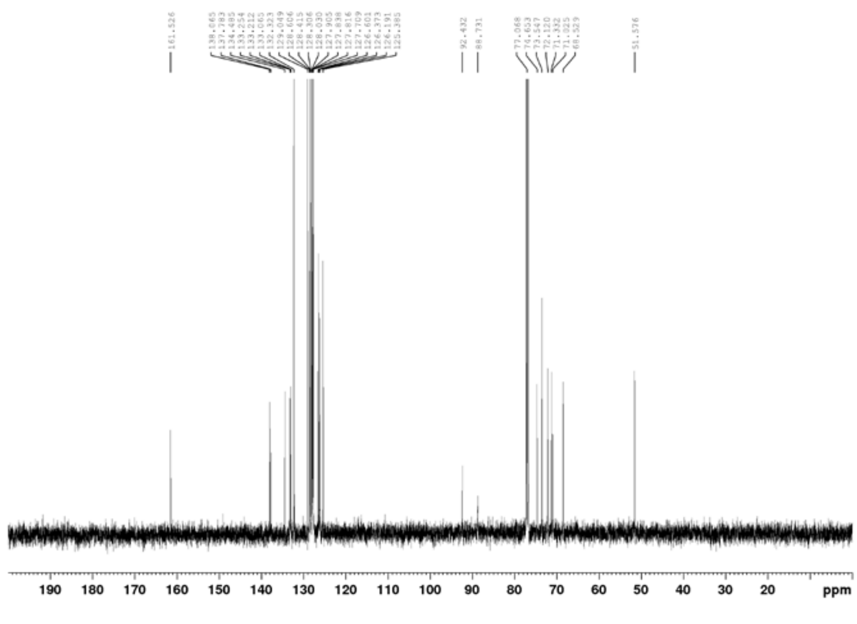

Compound 37

^{1}H NMR (500 MHz, $CDCl_3$) δ 7.78 – 7.71 (m, 4H), 7.44 – 7.34 (m, 5H), 7.21 (dd, *J* = 4.9, 1.6 Hz, 3H), 5.83 (d, *J* = 5.2 Hz, 1H), 5.65 (d, *J* = 2.4 Hz, 1H), 4.83 (d, *J* = 11.4 Hz, 1H), 4.71 – 4.63 (m, 1H), 4.60 – 4.49 (m, 2H), 4.17 (dd, *J* = 11.4, 5.2 Hz, 1H), 4.08 (dd, *J* = 11.4, 7.5 Hz, 1H), 3.87 (dd, *J* = 11.3, 3.0 Hz, 1H), 3.22 (dq, *J* = 14.3, 7.2 Hz, 1H), 2.07 (s, 3H), 1.97 (s, 3H). ^{13}C NMR (500 MHz, $CDCl_3$) δ 170.1, 169.9, 161.5, 134.2, 132.8, 132.7, 132.4, 132.1, 128.8, 128.1, 127.7, 127.5, 127.3, 126.7, 125.9, 125.8, 125.5, 87.5, 84.2, 73.2, 70.8, 67.7, 65.4, 62.0, 51.3, 20.4, 20.4. HRMS (ESI): *m/z*: calcd for $C_{29}H_{27}Cl_3NO_7S$ [M + Na]: 638.0579; found: 638.0584.

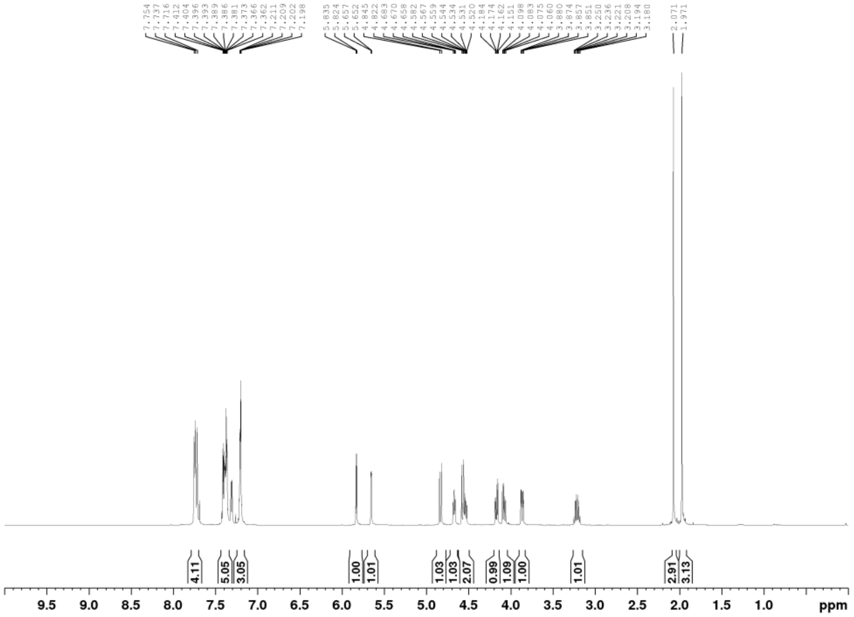

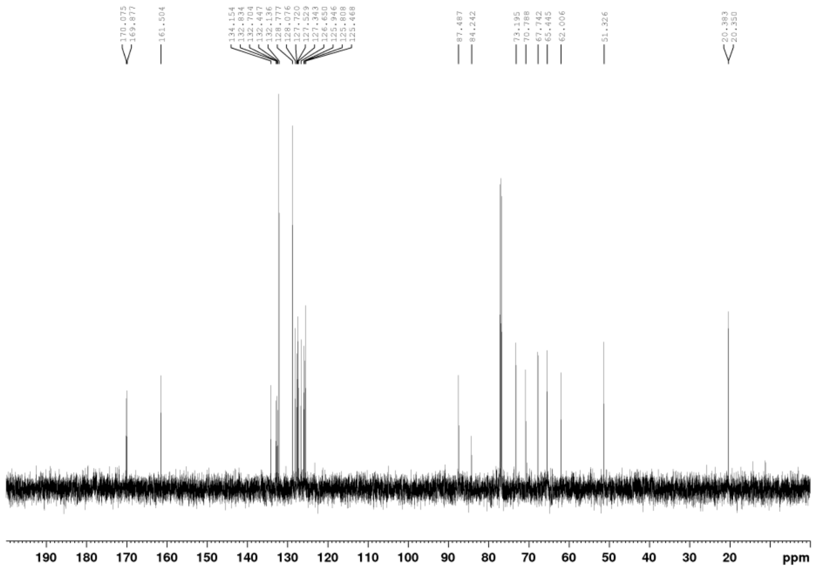

Compound 38

^{1}H NMR (500 MHz, $CDCl_3$) δ 7.80 (dd, *J* = 12.3, 4.3 Hz, 3H), 7.76 (s, 1H), 7.53 – 7.40 (m, 3H), 7.42 – 7.29 (m, 5H), 6.94 (d, *J* = 7.5 Hz, 1H), 5.73 (d, *J* = 3.0 Hz, 1H), 4.91 (dd, *J* = 10.9, 4.8 Hz, 1H), 4.80 (dd, *J* = 8.3, 5.3 Hz, 1H), 4.66 – 4.54 (m, 2H), 4.50 (dd, *J* = 11.9, 5.0 Hz, 1H), 4.24 (dd, *J* = 10.9, 3.2 Hz, 1H), 3.86 (t, *J* = 6.2 Hz, 1H), 3.72 (dd, *J* = 8.0, 2.7 Hz, 1H), 3.64 (dd, *J* = 9.4, 5.6 Hz, 1H), 3.58 (dd, *J* = 9.3, 7.1 Hz, 1H), 3.53 (s, 3H), 2.07 (s, 3H). ^{13}C NMR (500 MHz, $CDCl_3$) δ 170.1, 161.9, 137.4, 134.4, 133.0, 133.0, 128.3, 128.2, 127.9, 127.8, 127.8, 127.5, 127.3, 126.1, 126.0, 125.9, 100.5, 92.3, 74.4, 73.5, 72.1, 71.7, 67.7, 65.7, 57.1, 55.5, 20.6. HRMS (ESI): *m/z*: calcd for $C_{29}H_{30}Cl_3NNaO_7$ [M + Na]: 632.0980; found: 632.0988.

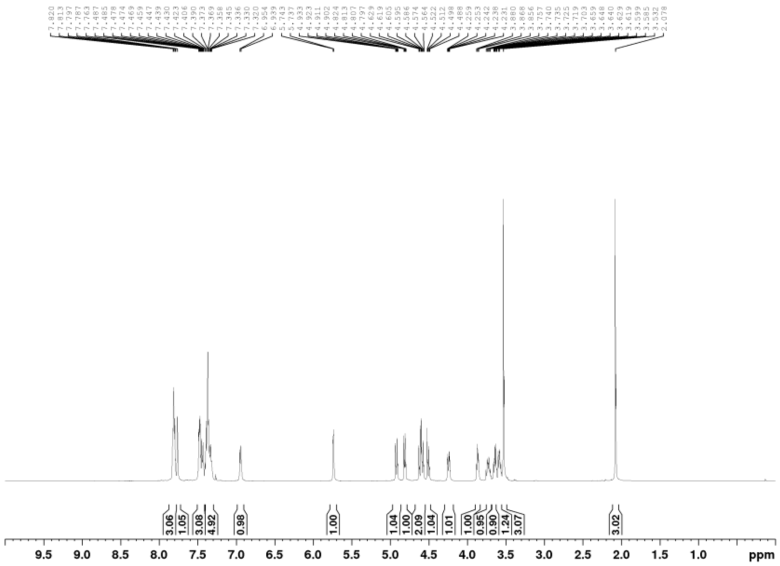

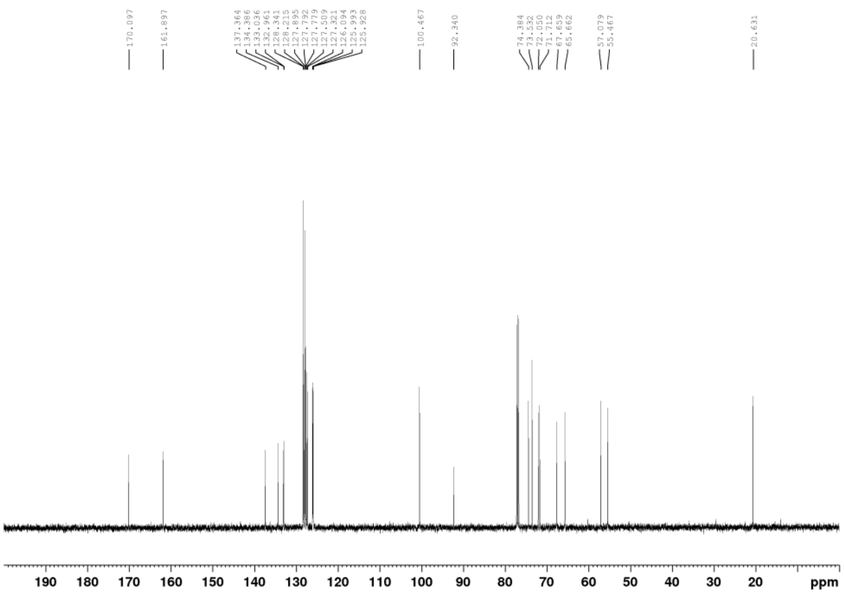

Compound 39

^{1}H NMR (500 MHz, $CDCl_3$) δ 7.96 – 7.73 (m, 4H), 7.56 – 7.40 (m, 3H), 7.34 – 7.19 (m, 5H), 7.03 (d, *J* = 7.1 Hz, 1H), 4.95 (d, *J* = 11.5 Hz, 1H), 4.86 (dd, *J* = 9.9, 3.4 Hz, 2H), 4.77 (d, *J* = 11.4 Hz, 1H), 4.65 (d, *J* = 11.7 Hz, 1H), 4.39 (dd, *J* = 10.9, 2.6 Hz, 1H), 4.27 (dd, *J* = 11.1, 6.6 Hz, 1H), 4.11 (dd, *J* = 11.2, 6.3 Hz, 1H), 3.91 – 3.88 (m, 1H), 3.89 – 3.82 (m, 1H), 3.67 (t, *J* = 6.4 Hz, 1H), 3.51 (s, 3H), 1.97 (s, 3H). ^{13}C NMR (500 MHz, $CDCl_3$) δ 170.4, 162.1, 137.8, 134.7, 133.2, 133.0, 128.4, 128.3, 128.2, 127.8, 127.8, 127.6, 126.8, 126.2, 126.1, 125.8, 100.0, 92.5, 77.3, 74.4, 72.8, 72.2, 71.9, 62.8, 57.0, 56.0, 20.6. HRMS (ESI): *m/z*: calcd for $C_{29}H_{30}Cl_3NNaO_7$ [M + Na]: 632.0980; found: 632.0985.

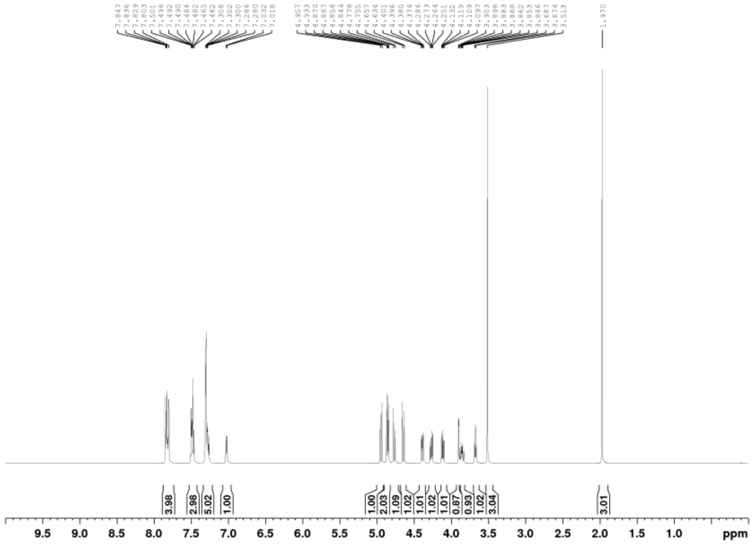

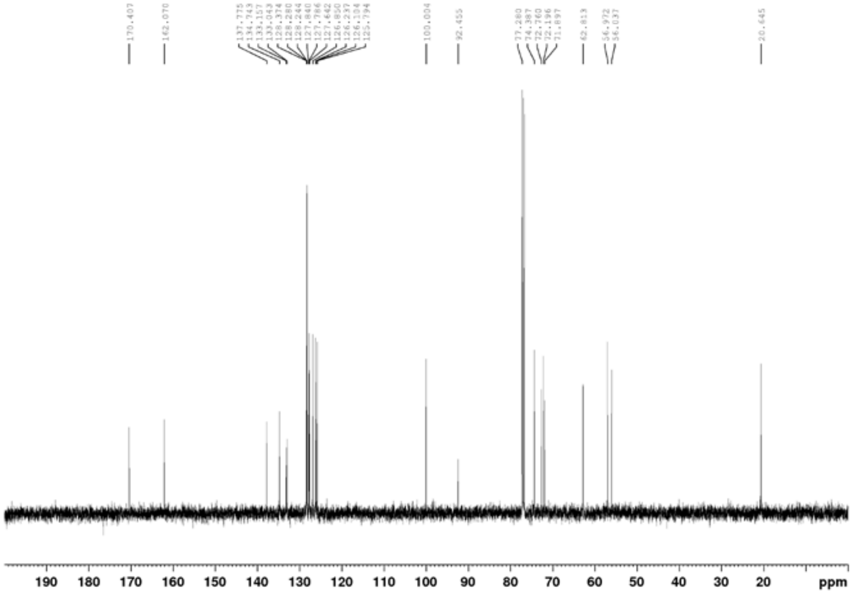

Compound 40

^{1}H NMR (500 MHz, $CDCl_3$) δ 7.93 – 7.75 (m, 4H), 7.59 – 7.43 (m, 3H), 7.42 – 7.24 (m, 10H), 7.02 (d, *J* = 7.3 Hz, 1H), 4.94 (d, *J* = 11.5 Hz, 1H), 4.85 (dd, *J* = 20.0, 9.9 Hz, 2H), 4.69 (dd, *J* = 26.7, 11.5 Hz, 2H), 4.51 (q, *J* = 11.8 Hz, 2H), 4.34 (dd, *J* = 11.0, 2.7 Hz, 1H), 4.09 (d, *J* = 2.6 Hz, 1H), 3.92 (dt, *J* = 10.8, 7.9 Hz, 1H), 3.78 – 3.62 (m, 3H), 3.53 (s, 3H). ^{13}C NMR (500 MHz, $CDCl_3$) δ 162.0, 138.2, 137.7, 134.8, 133.1, 133.0, 128.4, 128.3, 128.1, 128.1, 127.8, 127.8, 127.6, 127.5, 126.7, 126.1, 126.0, 125.8, 100.2, 92.5, 77.3, 74.5, 73.5, 73.4, 72.3, 72.2, 68.4, 56.9, 56.0. HRMS (ESI): *m/z*: calcd for $C_{34}H_{34}Cl_3NNaO_6$ [M + Na]: 680.1344; found: 680.1346.

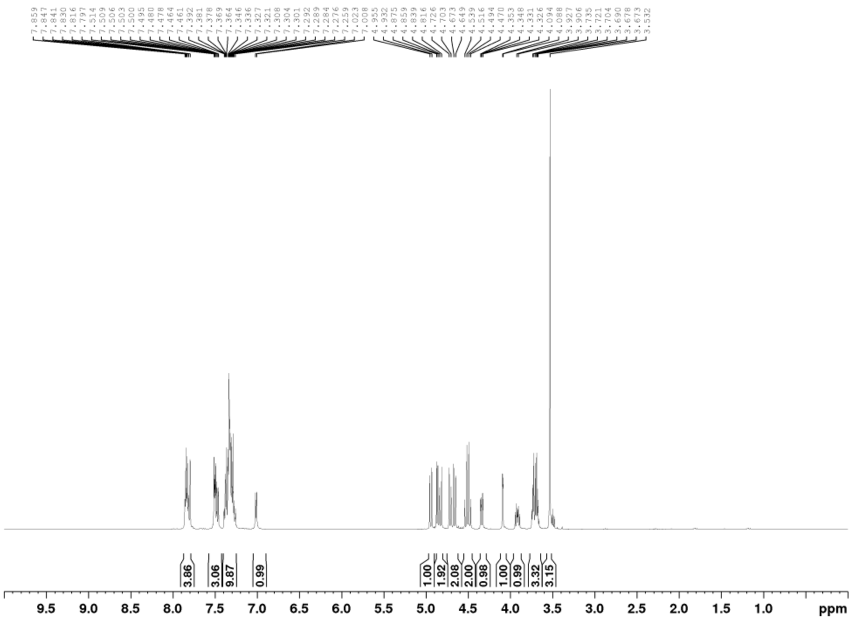

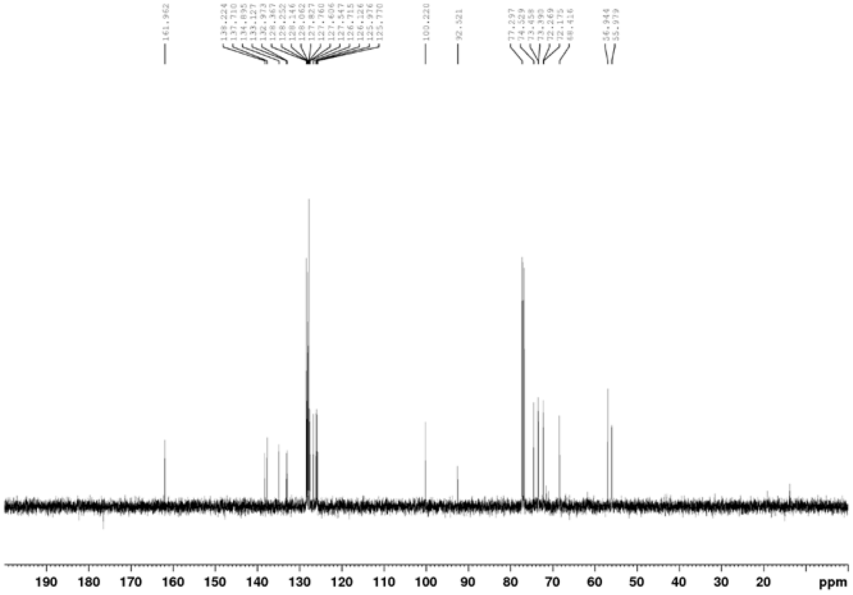

Compound 41

^{1}H NMR (500 MHz, $CDCl_3$) δ 7.83 – 7.76 (m, 3H), 7.73 (s, 1H), 7.50 – 7.43 (m, 2H), 7.40 (dd, *J* = 8.5, 1.2 Hz, 1H), 6.93 (d, *J* = 7.2 Hz, 1H), 5.62 (d, *J* = 2.9 Hz, 1H), 4.88 (d, *J* = 8.9 Hz, 2H), 4.56 (d, *J* = 10.8 Hz, 1H), 4.34 (dd, *J* = 10.9, 3.3 Hz, 1H), 4.20 (d, *J* = 6.6 Hz, 2H), 3.90 (t, *J* = 6.6 Hz, 1H), 3.62 (dt, *J* = 10.9, 7.9 Hz, 1H), 3.52 (s, 3H), 2.15 (s, 3H), 2.09 (s, 3H). ^{13}C NMR (500 MHz, $CDCl_3$) δ 170.5, 170.3, 162.0, 134.3, 133.2, 133.1, 128.4, 127.9, 127.6, 127.6, 126.2, 126.1, 126.1, 100.2, 92.4, 74.0, 72.1, 70.9, 65.6, 61.9, 57.2, 55.8, 20.8, 20.7. HRMS (ESI): *m/z*: calcd for $C_{24}H_{25}Cl_3NO_8$ [M – H]: 560.0651; found: 560.0655.

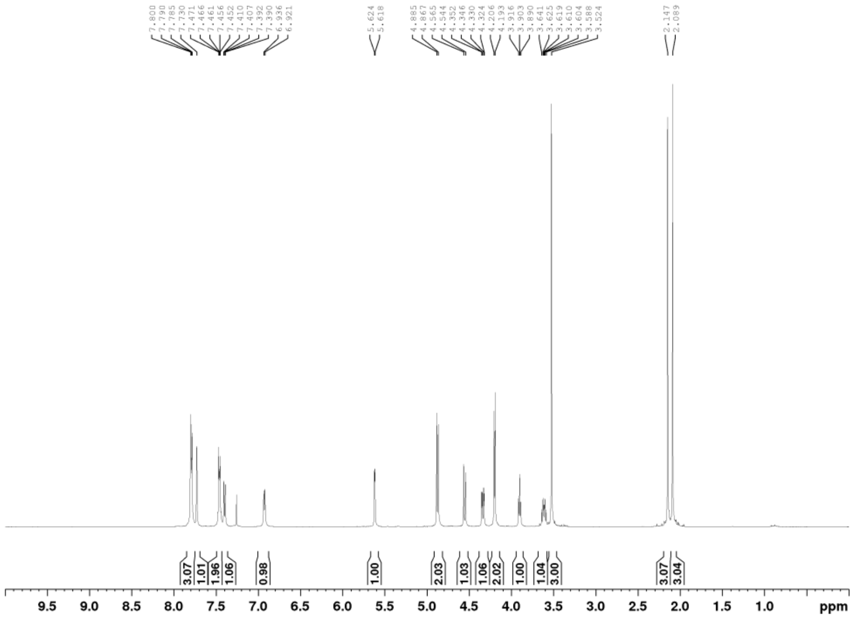

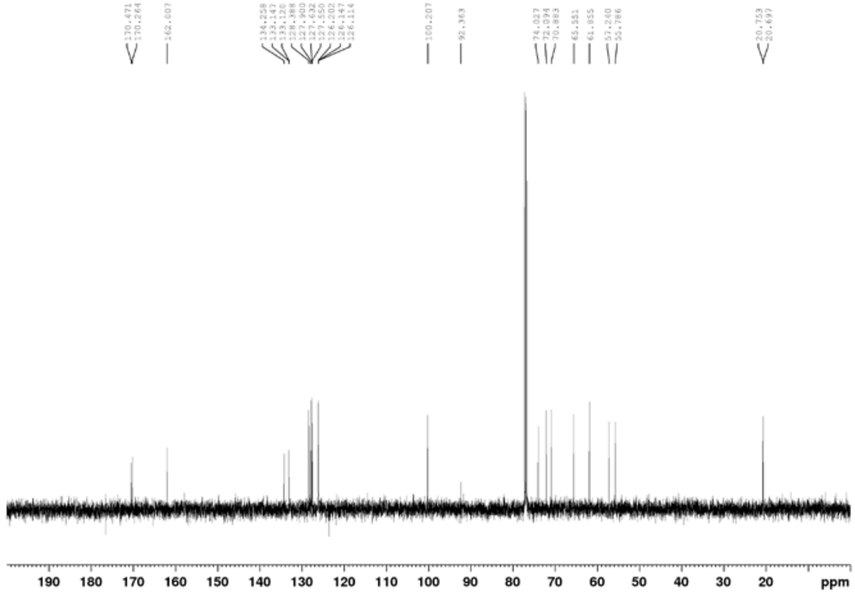

Acceptor A1

^{1}H NMR (500 MHz, $CDCl_3$) δ 7.39 – 7.25 (m, 10H), 6.90 (d, *J* = 7.3 Hz, 1H), 4.71 (d, *J* = 1.4 Hz, 2H), 4.61 – 4.43 (m, 3H), 4.02 (dd, *J* = 10.8, 3.3 Hz, 1H), 3.91 (d, *J* = 3.2 Hz, 1H), 3.76 (dd, *J* = 7.8, 2.8 Hz, 1H), 3.72 – 3.66 (m, 3H), 3.49 (s, 3H). ^{13}C NMR (500 MHz, $CDCl_3$) δ 162.6, 137.9, 137.6, 128.5, 128.4, 128.0, 127.9, 127.8, 127.8, 100.9, 92.5, 75.7, 75.3, 73.5, 73.5, 70.9, 68.1, 57.1, 56.8. HRMS (ESI): *m/z*: calcd for $C_{23}H_{26}Cl_3NNaO_6$ [M + Na]: 540.0718; found: 540.0710.

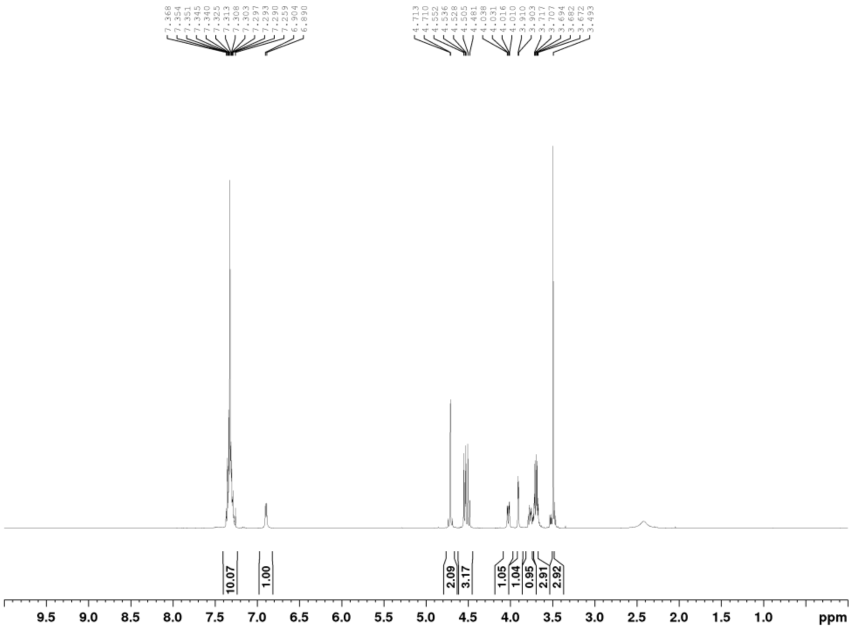

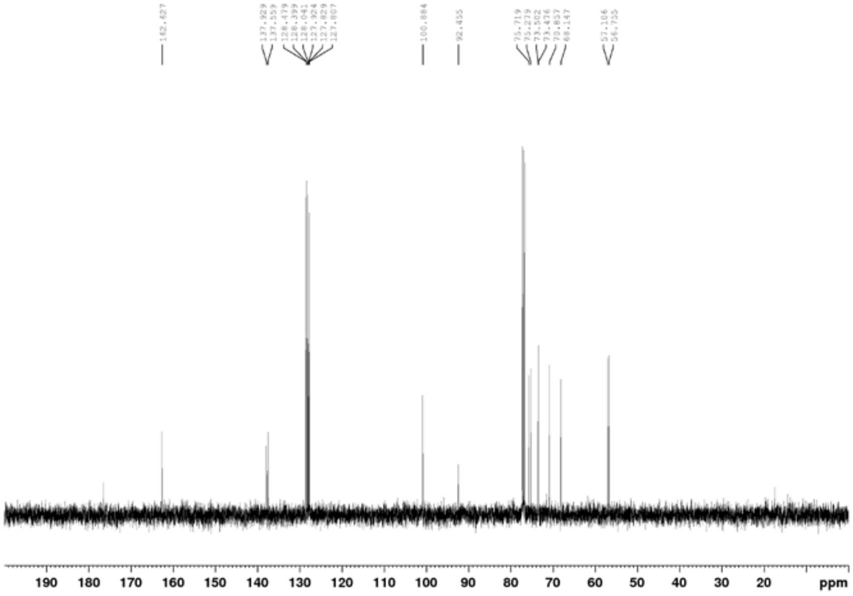

Acceptor A2

^{1}H NMR (500 MHz, MeOD) δ 7.33 (d, *J* = 4.2 Hz, 4H), 7.29 – 7.25 (m, 1H), 5.35 (d, *J* = 2.8 Hz, 1H), 4.54 (d, *J* = 11.8 Hz, 1H), 4.48 (dd, *J* = 9.7, 7.9 Hz, 2H), 4.00 (dd, *J* = 10.8, 3.2 Hz, 1H), 3.95 – 3.88 (m, 1H), 3.85 (t, *J* = 6.0 Hz, 1H), 3.58 – 3.52 (m, 2H), 3.49 (d, *J* = 14.5 Hz, 3H), 2.09 (s, 3H). ^{13}C NMR (500 MHz, MeOD) δ 172.3, 164.5, 139.2, 129.3, 129.0, 128.8, 103.5, 94.1, 74.5, 73.6, 71.4, 70.3, 69.6, 57.4, 56.4, 20.9. HRMS (ESI): *m/z*: calcd for $C_{18}H_{22}Cl_{3}NNaO_{7}$ [M + Na]: 492.0354; found: 492.0361.

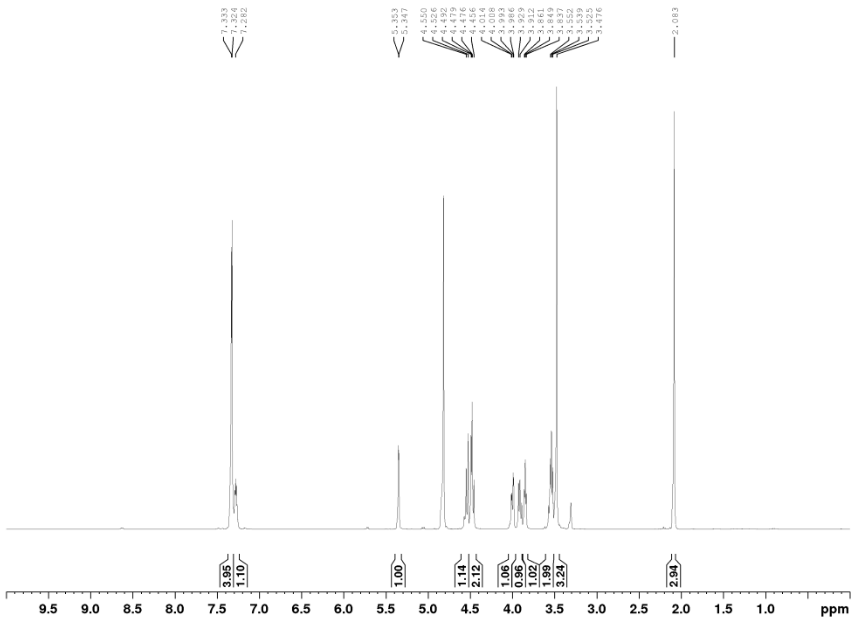

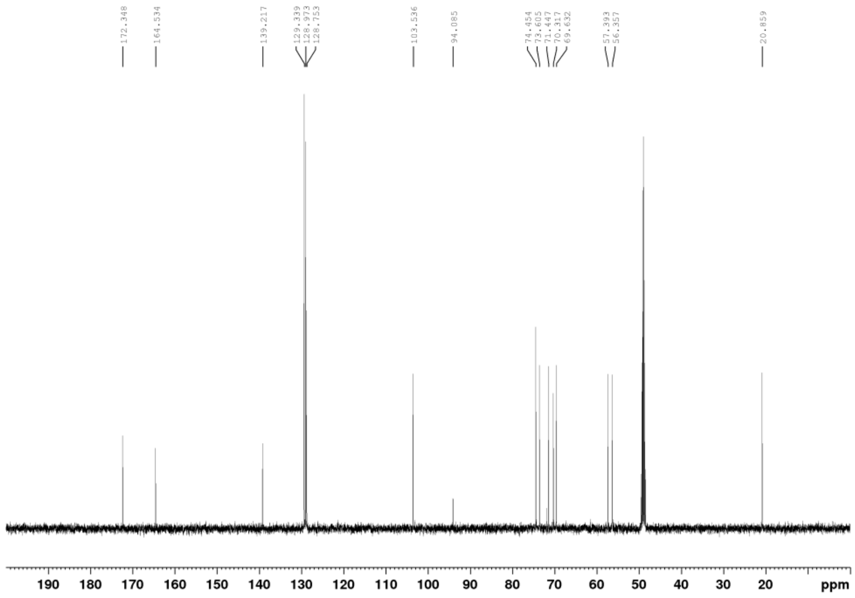

Acceptor A3

^{1}H NMR (500 MHz, $CDCl_3$) δ 7.41 – 7.21 (m, 5H), 6.98 (d, *J* = 7.2 Hz, 1H), 4.73 (dd, *J* = 26.3, 11.6 Hz, 2H), 4.51 (d, *J* = 8.3 Hz, 1H), 4.30 (dd, *J* = 11.2, 6.6 Hz, 1H), 4.07 (ddd, *J* = 10.9, 7.9, 4.8 Hz, 2H), 3.85 – 3.74 (m, 2H), 3.67 (t, *J* = 6.5 Hz, 1H), 3.46 (s, 3H), 2.00 (s, 3H). ^{13}C NMR (500 MHz, $CDCl_3$) δ 170.5, 162.8, 137.5, 128.5, 128.2, 128.0, 100.8, 92.4, 75.4, 72.3, 71.1, 62.6, 56.9, 56.8, 20.7. HRMS (ESI): *m/z*: calcd for $C_{18}H_{22}Cl_3NNaO_7$ [M + Na]: 492.0354; found: 492.0362.

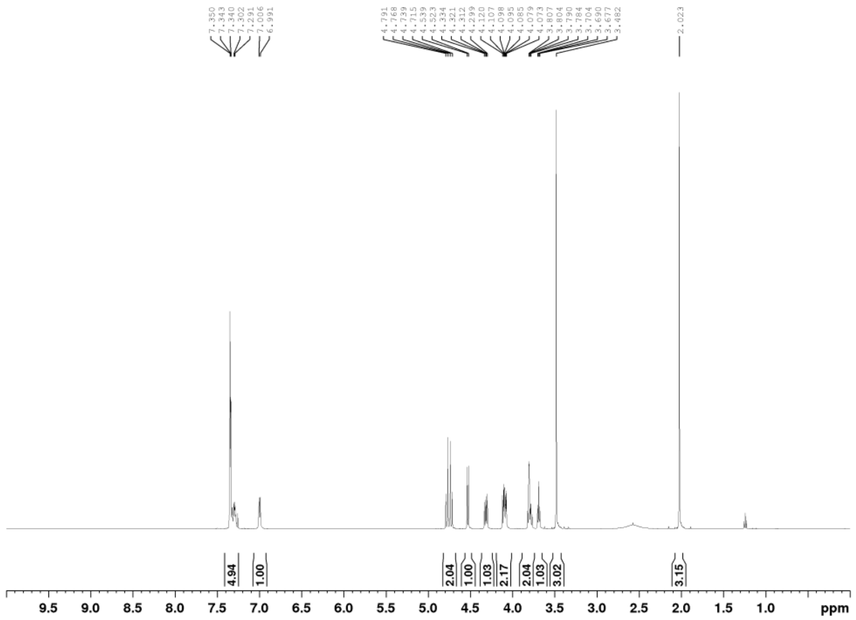

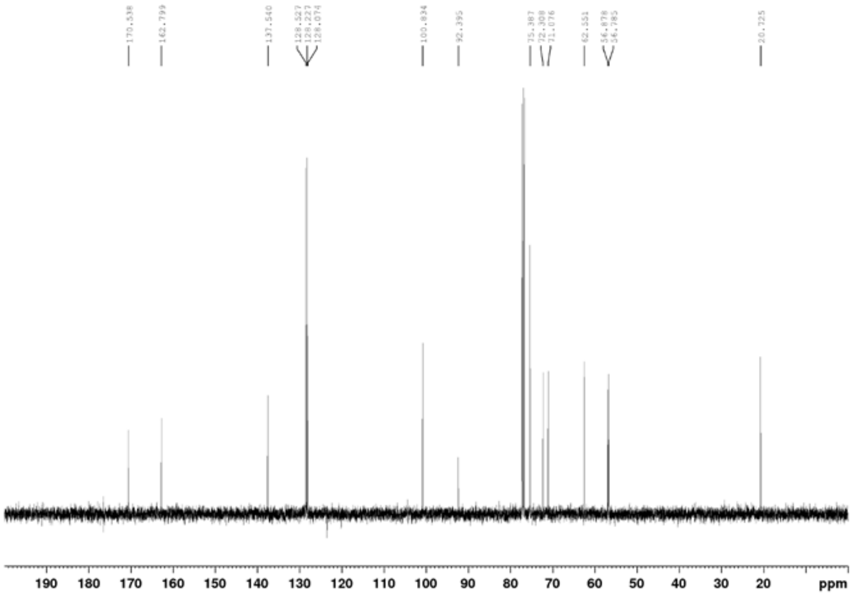

Acceptor A4

^{1}H NMR (500 MHz, $CDCl_3$) δ 7.22 (d, J = 7.8 Hz, 1H), 5.31 (d, J = 3.1 Hz, 1H), 4.62 (d, J = 8.3 Hz, 1H), 4.37 – 4.19 (m, 1H), 4.13 (d, J = 6.4 Hz, 2H), 3.87 (t, J = 6.3 Hz, 1H), 3.83 – 3.72 (m, 1H), 3.70 (d, J = 6.2 Hz, 1H), 3.49 (s, 3H), 2.14 (s, 3H), 2.04 (s, 3H). ^{13}C NMR (500 MHz, $CDCl_3$) δ 171.3, 170.6, 162.8, 101.0, 92.3, 70.9, 69.4, 68.7, 62.0, 57.1, 56.1, 20.7, 20.7. HRMS (ESI): *m/z*: calcd for $C_{13}H_{17}Cl_3NO_8$ [M – H]: 420.0025; found: 420.0022.

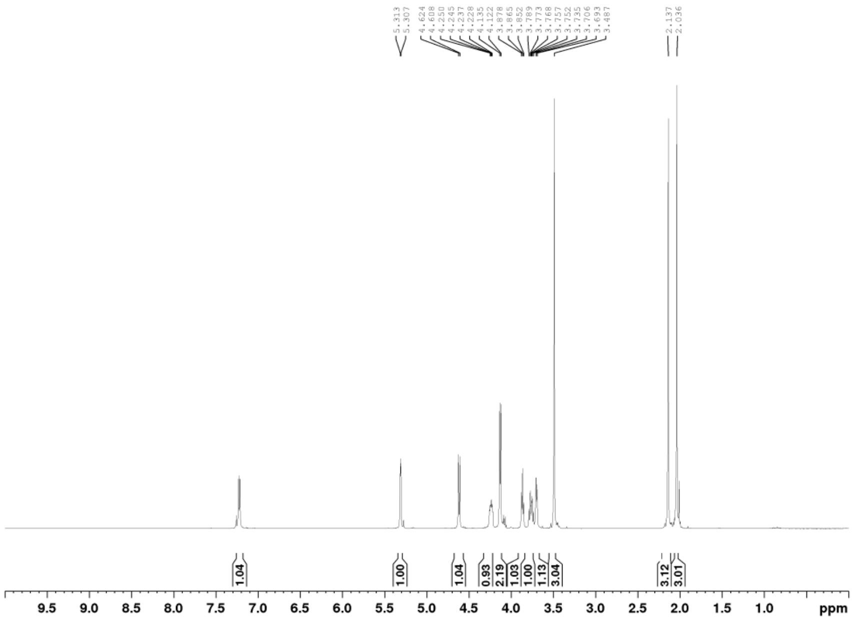

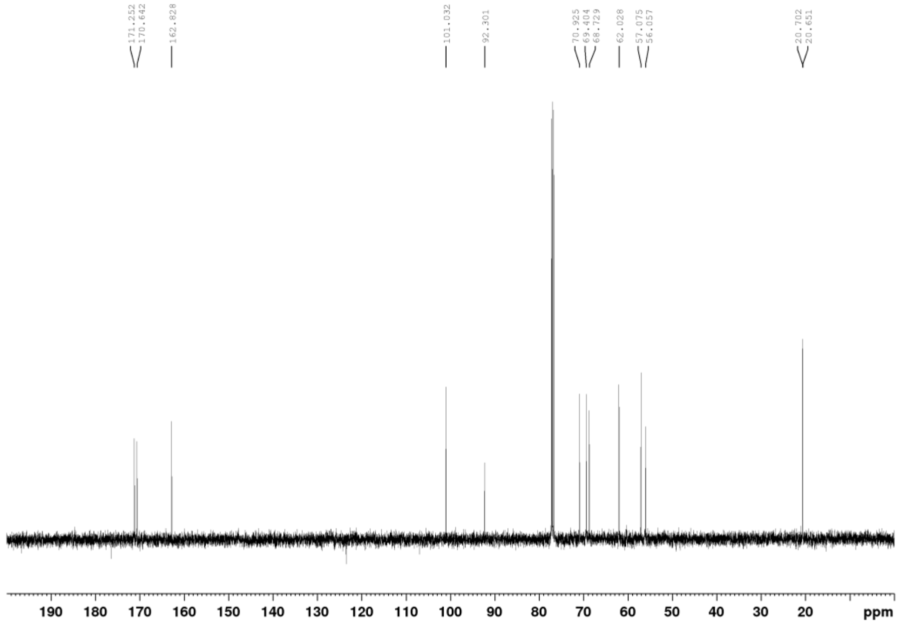

Protected dimer D1-A2

^{1}H NMR (500 MHz, $CDCl_3$) δ 7.58 (dd, *J* = 5.9, 1.9 Hz, 2H), 7.35 – 7.26 (m, 16H), 7.16 – 7.12 (m, 2H), 6.71 (d, *J* = 7.4 Hz, 1H), 6.05 (d, *J* = 4.8 Hz, 1H), 5.63 (d, *J* = 3.1 Hz, 1H), 4.86 (d, *J* = 8.3 Hz, 1H), 4.69 (d, *J* = 11.7 Hz, 1H), 4.53 (dt, *J* = 20.2, 11.4 Hz, 5H), 4.44 (d, *J* = 11.4 Hz, 1H), 4.38 (dd, *J* = 10.9, 3.3 Hz, 1H), 3.95 (d, *J* = 7.9 Hz, 1H), 3.83 – 3.71 (m, 3H), 3.65 (s, 3H), 3.59 – 3.53 (m, 3H), 3.51 (d, *J* = 5.2 Hz, 3H), 2.05 (s, 3H). ^{13}C NMR (500 MHz, $CDCl_3$) δ 169.9, 169.8, 162.2, 137.6, 137.6, 137.5, 137.4, 129.8, 129.4, 128.4, 128.3, 128.2, 127.9, 127.9, 127.8, 127.8, 127.7, 126.0, 122.4, 100.2, 97.7, 92.3, 78.0, 77.7, 75.7, 73.7, 73.2, 72.7, 72.5, 71.8, 69.5, 68.9, 68.4, 57.3, 55.2, 52.3, 20.8. HRMS (ESI): *m/z*: calcd for $C_{46}H_{48}Cl_3NNaO_{14}$ [M + Na]: 966.2033; found: 966.2038.

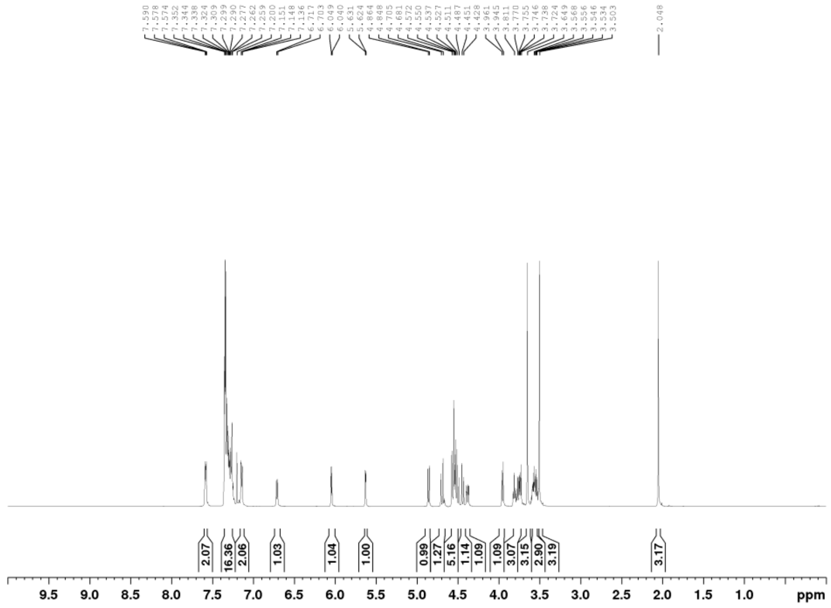

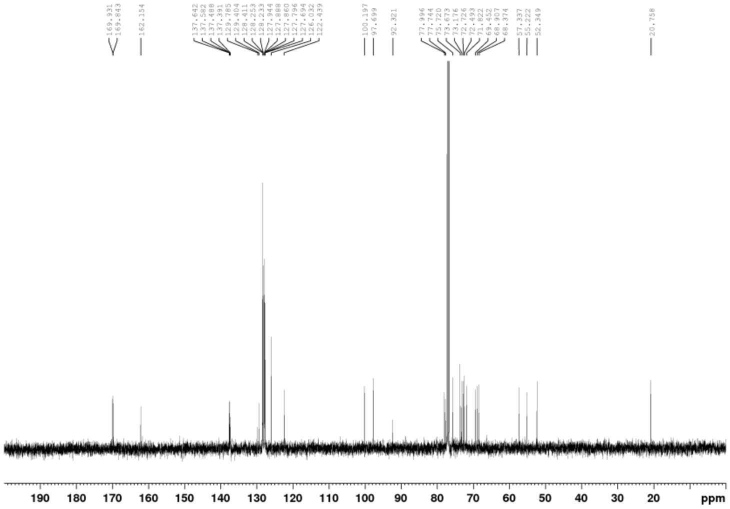

Compound 42b

^{1}H NMR (500 MHz, $CDCl_3$) δ 7.60 (dd, *J* = 7.4, 1.9 Hz, 2H), 7.41 – 7.21 (m, 16H), 7.19 – 7.09 (m, 2H), 6.10 (d, *J* = 4.8 Hz, 1H), 5.59 (d, *J* = 3.0 Hz, 1H), 4.95 (d, *J* = 7.9 Hz, 1H), 4.68 (dd, *J* = 23.7, 10.1 Hz, 2H), 4.52 (tdd, *J* = 14.4, 10.2, 4.5 Hz, 5H), 4.42 (d, *J* = 11.5 Hz, 1H), 4.19 (dd, *J* = 10.9, 3.2 Hz, 1H), 3.93 (d, *J* = 8.4 Hz, 1H), 3.85 – 3.72 (m, 3H), 3.67 (s, 3H), 3.58 – 3.47 (m, 3H), 3.45 (d, *J* = 4.8 Hz, 3H), 2.05 (s, 3H), 1.78 (s, 3H). ^{13}C NMR (500 MHz, $CDCl_3$) δ 170.6, 170.3, 169.7, 137.7, 137.6, 137.4, 137.2, 129.4, 128.4, 128.4, 128.2, 128.0, 127.9, 127.9, 127.8, 127.7, 127.7, 126.1, 121.8, 101.3, 98.0, 76.6, 76.0, 73.6, 72.9, 72.4, 72.2, 71.1, 69.8, 68.4, 56.9, 53.3, 52.4, 23.8, 20.9. HRMS (ESI): *m/z*: calcd for $C_{46}H_{51}NNaO_{14}$ [M + Na]: 864.3202; found: 864.3205.

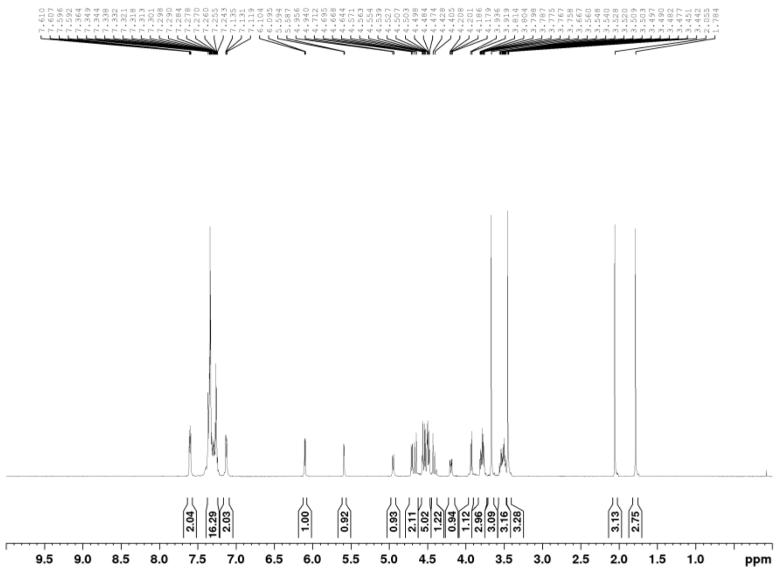

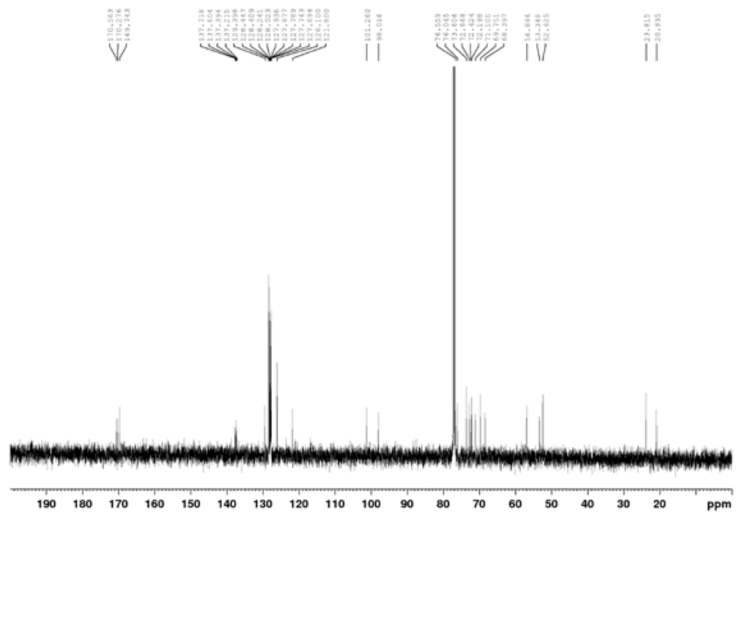

Compound 43b

^{1}H NMR (500 MHz, DMSO) δ 7.69 (d, *J* = 8.9 Hz, 1H), 7.38 – 7.18 (m, 14H), 4.87 (d, *J* = 11.5 Hz, 1H), 4.82 (s, 1H), 4.68 (dd, *J* = 18.3, 11.2 Hz, 2H), 4.58 (d, *J* = 10.9 Hz, 1H), 4.51 (s, 2H), 4.46 (d, *J* = 7.6 Hz, 1H), 4.30 (d, *J* = 8.5 Hz, 1H), 3.86 (dd, *J* = 20.1, 10.9 Hz, 2H), 3.77 (d, *J* = 9.2 Hz, 1H), 3.69 – 3.58 (m, 4H), 3.54 (dd, *J* = 11.3, 8.4 Hz, 1H), 3.48 – 3.43 (m, 1H), 3.40 – 3.33 (m, 2H), 3.32 (s, 3H), 1.82 (s, 3H). ^{13}C NMR (500 MHz, $CDCl_3$) δ 170.5, 169.9, 138.9, 138.5, 138.3, 128.2, 128.0, 127.6, 127.6, 127.4, 127.4, 127.3, 127.2, 104.2, 101.6, 83.7, 79.7, 79.3, 73.7, 73.6, 73.3, 73.2, 72.2, 69.4, 67.6, 59.9, 55.3, 50.5, 23.1. HRMS (ESI): *m/z*: calcd for $C_{36}H_{43}NNaO_{12}$ [M + Na]: 704.2677; found: 704.2678.

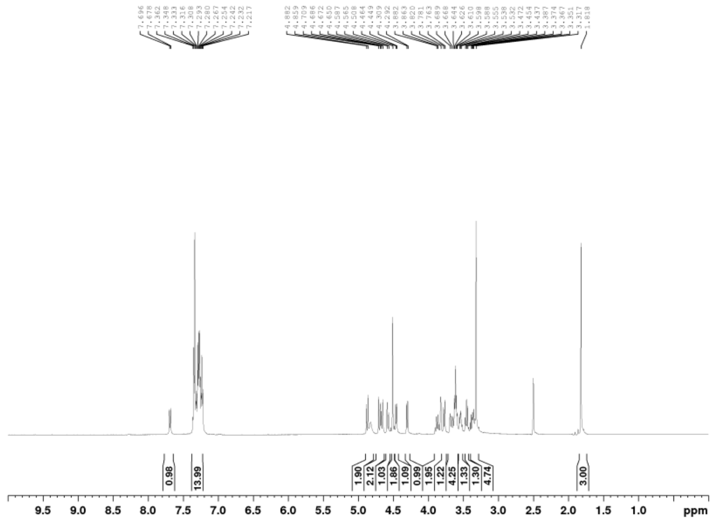

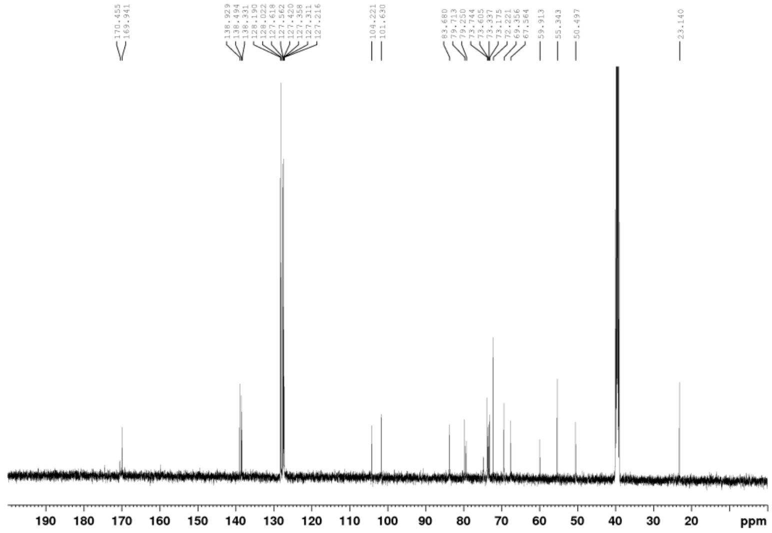

Compound 44b

^{1}H NMR (500 MHz, DMSO) δ 7.95 (d, *J* = 7.2 Hz, 1H), 7.37 – 7.20 (m, 14H), 4.91 (d, *J* = 11.4 Hz, 1H), 4.79 (d, *J* = 8.2 Hz, 1H), 4.63 – 4.37 (m, 9H), 3.95 (d, *J* = 10.9 Hz, 1H), 3.74 (d, *J* = 11.4 Hz, 3H), 3.68 (d, *J* = 7.9 Hz, 1H), 3.61 (t, *J* = 8.5 Hz, 2H), 3.38 (s, 3H), 1.89 (s, 3H). ^{13}C NMR (500 MHz, DMSO) δ 172.1, 170.9, 138.9, 138.8, 138.8, 128.1, 127.9, 127.8, 127.8, 127.6, 127.3, 127.0, 102.8, 100.7, 80.2, 78.6, 77.8, 74.2, 73.3, 72.2, 72.0, 71.9, 70.3, 55.5, 52.8, 48.6, 23.2. HRMS (ESI): *m/z*: calcd for $C_{36}H_{40}NNa_2O_{18}S$ [M - Na]: 884.1488; found: 884.1480.

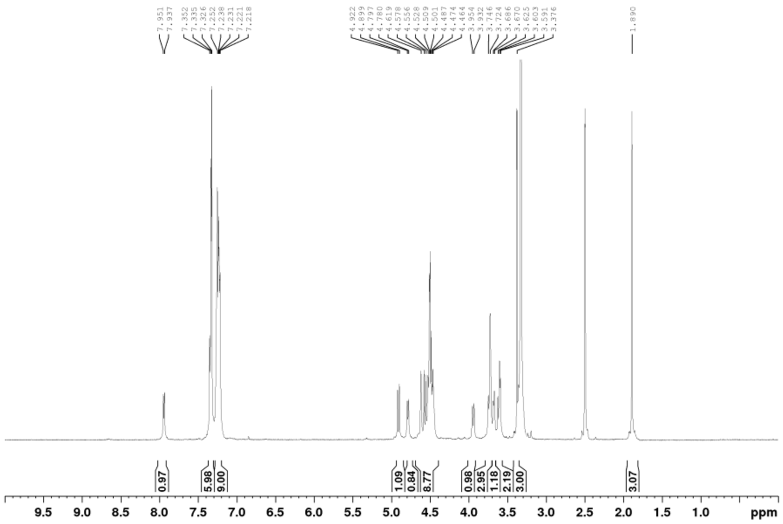

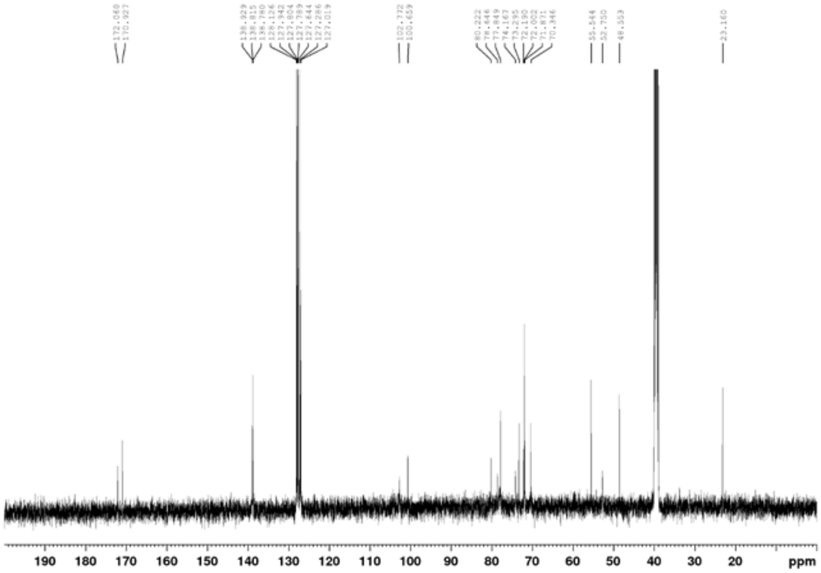

Compound 45b

^{1}H NMR (500 MHz, D_2O) δ 4.83 (d, *J* = 2.8 Hz, 1H), 4.62 (dd, *J* = 8.0, 4.1 Hz, 2H), 4.13 (dt, *J* = 15.1, 5.2 Hz, 2H), 3.76 (dt, *J* = 8.2, 5.8 Hz, 4H), 3.72 – 3.61 (m, 4H), 3.47 (s, 3H), 2.00 (s, 3H). ^{13}C NMR (500 MHz, D_2O) δ 175.7, 174.9, 101.9, 101.8, 80.2, 76.4, 76.3, 76.2, 75.1, 74.7, 71.3, 61.1, 57.1, 52.5, 22.4. HRMS (ESI): *m/z*: calcd for $C_{15}H_{22}NNa_2O_{18}S_2$ [M – Na]: 614.0079; found: 614.0076.

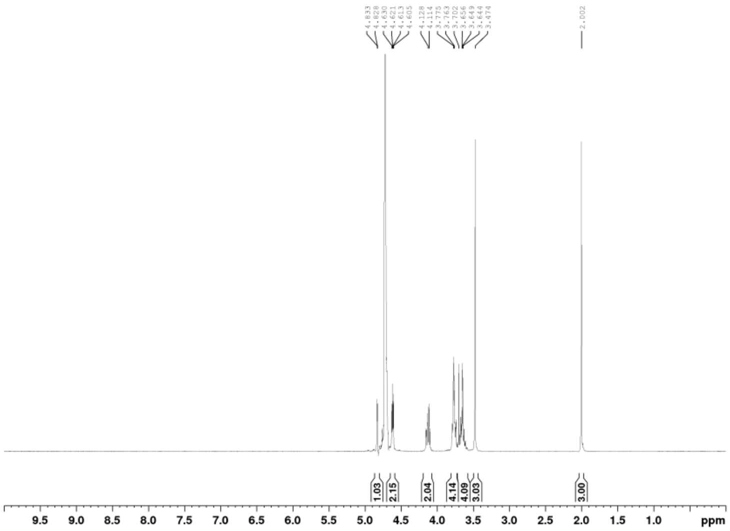

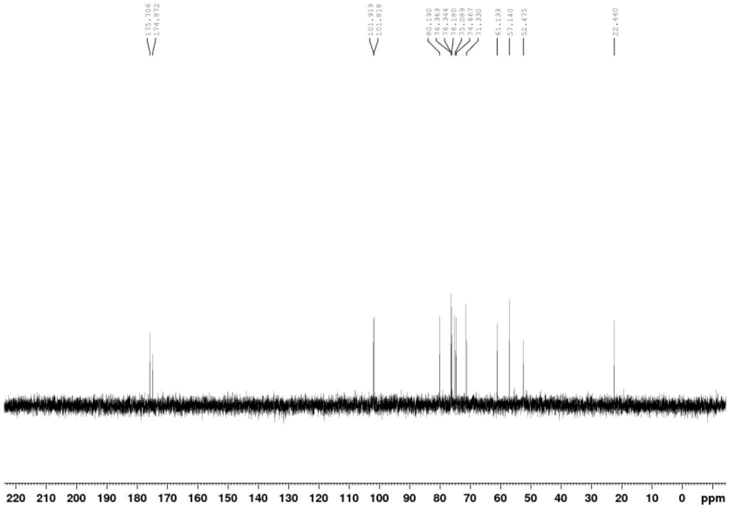

Protected dimer D1-A3

^{1}H NMR (500 MHz, $CDCl_3$) δ 8.03 – 7.94 (m, 2H), 7.61 – 7.54 (m, 1H), 7.47 – 7.38 (m, 4H), 7.36 – 7.23 (m, 8H), 7.18 – 7.06 (m, 5H), 6.83 (d, *J* = 6.7 Hz, 1H), 5.41 (dd, *J* = 9.0, 7.9 Hz, 1H), 5.00 (d, *J* = 11.4 Hz, 1H), 4.83 – 4.72 (m, 4H), 4.67 (ddd, *J* = 14.4, 11.9, 5.3 Hz, 4H), 4.19 (dd, *J* = 11.2, 6.7 Hz, 1H), 4.10 – 3.94 (m, 4H), 3.87 – 3.80 (m, 1H), 3.77 (s, 3H), 3.68 (t, *J* = 6.4 Hz, 1H), 3.54 (ddd, *J* = 10.9, 8.2, 6.8 Hz, 1H), 3.43 (s, 3H), 1.98 (s, 3H). ^{13}C NMR (500 MHz, $CDCl_3$) δ 170.4, 168.5, 164.9, 162.2, 138.0, 137.4, 137.2, 133.3, 129.9, 129.3, 128.9, 128.4, 128.4, 128.2, 128.1, 127.9, 127.9, 127.7, 127.6, 101.3, 99.1, 92.0, 81.4, 79.4, 75.8, 75.1, 75.0, 74.55, 74.5, 74.2, 73.2, 71.9, 62.7, 57.0, 56.3, 52.5, 20.6. HRMS (ESI): *m/z*: calcd for $C_{46}H_{48}Cl_3NNaO_{14}$ [M + Na]: 966.2033; found: 966.2039.

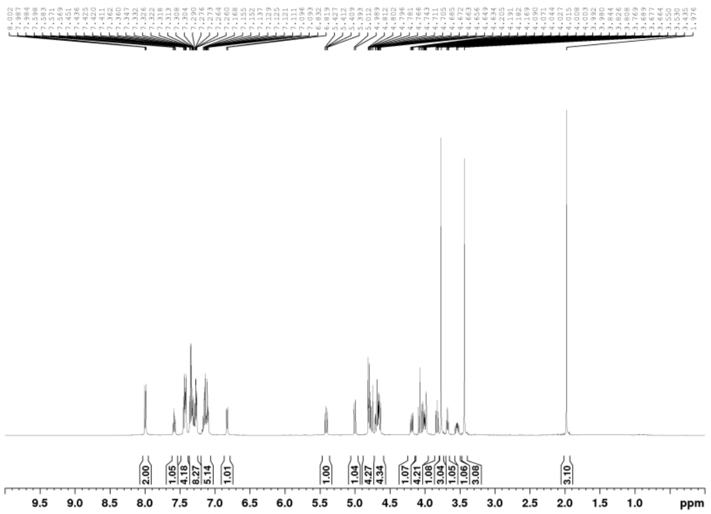

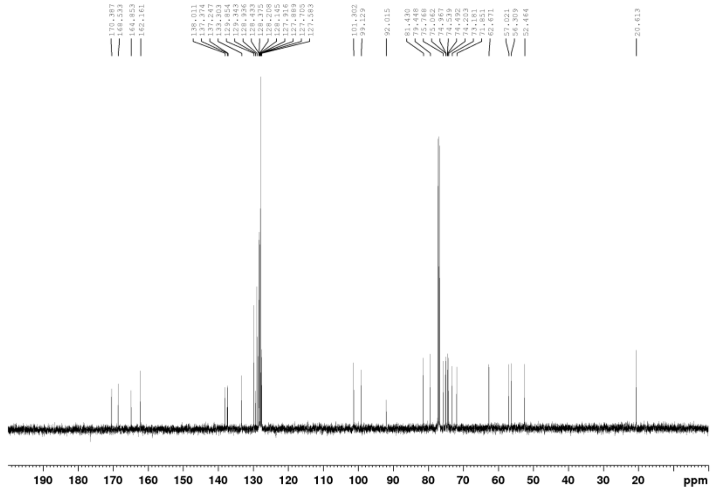

Compound 42c

^{1}H NMR (500 MHz, $CDCl_3$) δ 7.99 (d, *J* = 7.5 Hz, 2H), 7.60 (t, *J* = 7.5 Hz, 1H), 7.48 – 7.38 (m, 4H), 7.38 – 7.21 (m, 8H), 7.19 – 7.06 (m, 5H), 5.36 (dd, *J* = 9.0, 8.2 Hz, 1H), 5.30 (d, *J* = 6.6 Hz, 1H), 4.96 (d, *J* = 11.6 Hz, 1H), 4.86 – 4.60 (m, 8H), 4.14 (dd, *J* = 11.2, 6.8 Hz, 1H), 4.07 – 3.98 (m, 2H), 3.94 (dd, *J* = 11.1, 6.0 Hz, 2H), 3.87 (d, *J* = 8.6 Hz, 1H), 3.75 (s, 3H), 3.64 (t, *J* = 6.3 Hz, 1H), 3.40 (s, 3H), 3.18 (dd, *J* = 7.1, 3.0 Hz, 1H), 1.93 (d, *J* = 6.5 Hz, 3H), 1.50 (s, 3H). ^{13}C NMR (500 MHz, $CDCl_3$) δ 171.2, 170.4, 168.6, 164.7, 138.4, 137.6, 137.5, 133.4, 129.6, 129.0, 128.6, 128.4, 128.3, 128.2, 127.9, 127.9, 127.7, 127.6, 102.4, 99.5, 81.6, 79.5, 77.9, 75.1, 75.1, 74.6, 74.3, 74.1, 73.6, 71.7, 63.0, 56.7, 55.6, 52.5, 23.4, 20.7. HRMS (ESI): *m/z*: calcd for $C_{46}H_{51}NNaO_{14}$ [M + Na]: 864.3202; found: 864.3207.

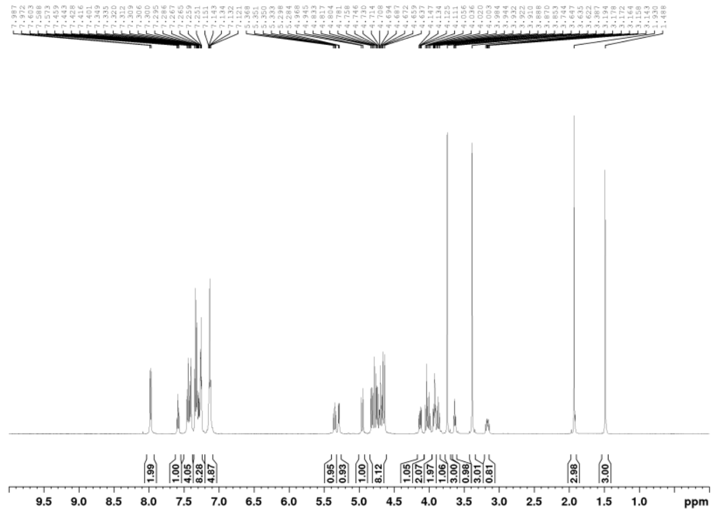

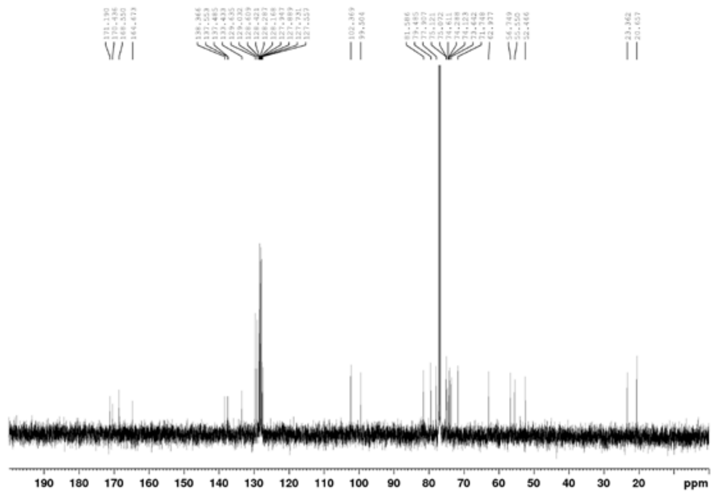

Compound 43c

^{1}H NMR (500 MHz, DMSO) δ 7.71 (d, *J* = 7.5 Hz, 1H), 7.41 – 7.19 (m, 14H), 4.93 – 4.82 (m, 2H), 4.71 (d, *J* = 11.5 Hz, 1H), 4.68 – 4.56 (m, 2H), 4.54 (d, *J* = 11.5 Hz, 1H), 4.48 (d, *J* = 7.5 Hz, 1H), 4.31 (d, *J* = 7.4 Hz, 1H), 3.90 (s, 1H), 3.83 (dd, *J* = 12.5, 8.8 Hz, 3H), 3.64 (t, *J* = 9.3 Hz, 1H), 3.53 – 3.46 (m, 2H), 3.44 – 3.32 (m, 3H), 3.31 (s, 3H), 1.83 (s, 3H). ^{13}C NMR (500 MHz, DMSO) δ 170.5, 170.0, 139.2, 139.0, 138.4, 128.0, 127.9, 127.7, 127.5, 127.4, 127.2, 104.7, 101.7, 83.8, 80.5, 79.2, 75.3, 74.7, 73.9, 73.9, 73.8, 73.8, 60.0, 55.5, 51.3, 23.2. HRMS (ESI): *m/z*: calcd for $C_{36}H_{43}NNaO_{12}$ [M + Na]: 704.2677; found: 704.2670.

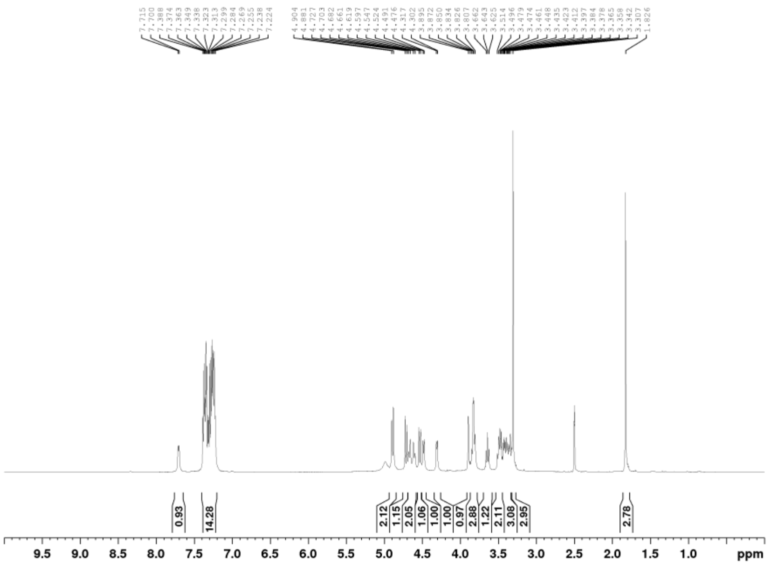

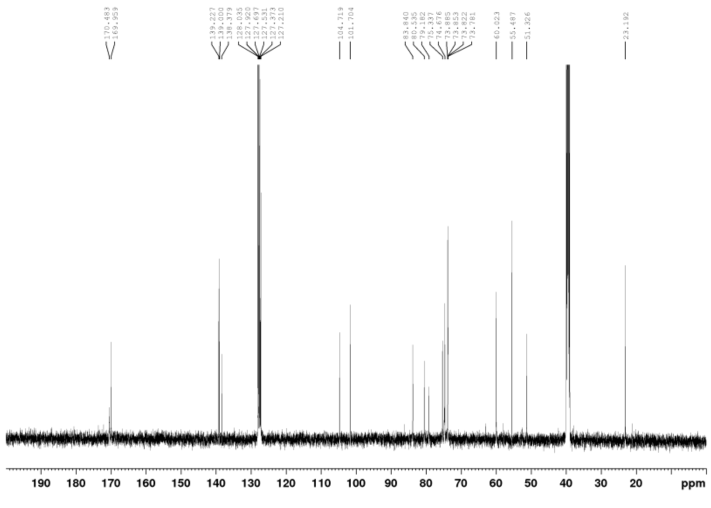

Compound 44c

^{1}H NMR (500 MHz, DMSO) δ 7.93 (d, *J* = 8.0 Hz, 1H), 7.35 (dd, *J* = 6.5, 2.7 Hz, 2H), 7.32 – 7.28 (m, 2H), 7.24 – 7.17 (m, 10H), 5.09 (d, *J* = 11.1 Hz, 1H), 4.93 (d, *J* = 11.3 Hz, 1H), 4.77 (d, *J* = 4.9 Hz, 1H), 4.66 (d, *J* = 11.3 Hz, 1H), 4.57 – 4.51 (m, 3H), 4.48 (d, *J* = 11.1 Hz, 1H), 4.29 (t, *J* = 5.0 Hz, 1H), 4.18 (s, 1H), 4.04 – 3.93 (m, 2H), 3.91 (dd, *J* = 10.3, 6.5 Hz, 1H), 3.82 (dd, *J* = 10.3, 5.9 Hz, 1H), 3.70 – 3.57 (m, 4H), 3.29 (s, 3H), 1.87 (s, 3H). ^{13}C NMR (500 MHz, DMSO) δ 173.0, 170.2, 139.5, 139.3, 139.1, 128.0, 127. 9, 127.8, 127.8, 127.5, 127.0, 126.9, 101.6, 100.9, 82.9, 79.0, 77.8, 77.4, 76.7, 75.4, 73.9, 72.9, 72.7, 71.9, 64.7, 55.6, 52.1, 23.1. HRMS (ESI): *m/z*: calcd for $C_{36}H_{40}NNa_2O_{18}S_2$ [M – Na]: 884.1488; found: 884.1489.

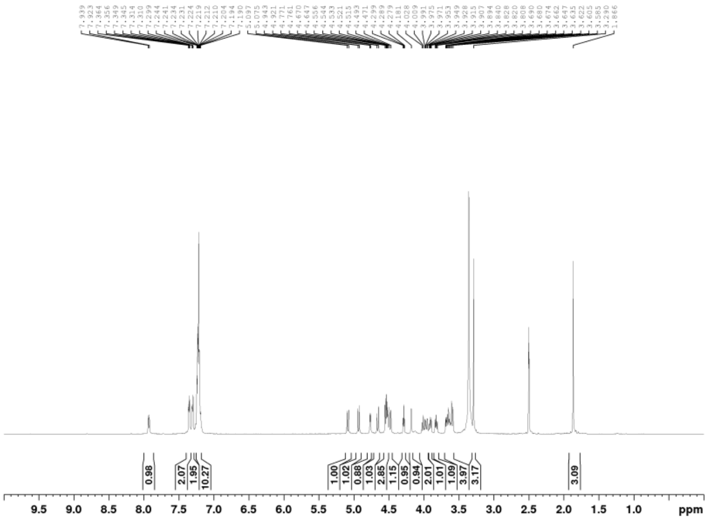

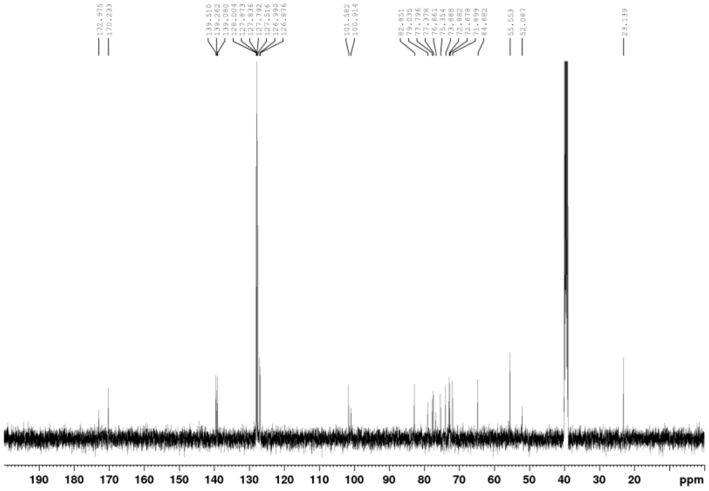

Compound 45c

^{1}H NMR (500 MHz, D_2O) δ 4.48 (d, *J* = 8.2 Hz, 1H), 4.25 – 4.14 (m, 3H), 4.09 – 4.03 (m, 1H), 3.91 (tdd, *J* = 16.7, 11.2, 5.6 Hz, 3H), 3.73 – 3.66 (m, 3H), 3.56 (t, *J* = 9.5 Hz, 1H), 3.47 (s, 3H), 2.01 (s, 3H). ^{13}C NMR (500 MHz, D_2O) δ 175.7, 174.9, 102.4, 101.2, 79.9, 79.7, 76.0, 74.6, 72.6, 71.5, 67.8, 67.2, 57.1, 51.1, 22.5. HRMS (ESI): *m/z*: calcd for $C_{15}H_{22}NNa_2O_{18}S_2$ [M – Na]: 614.0079; found: 614.0085.

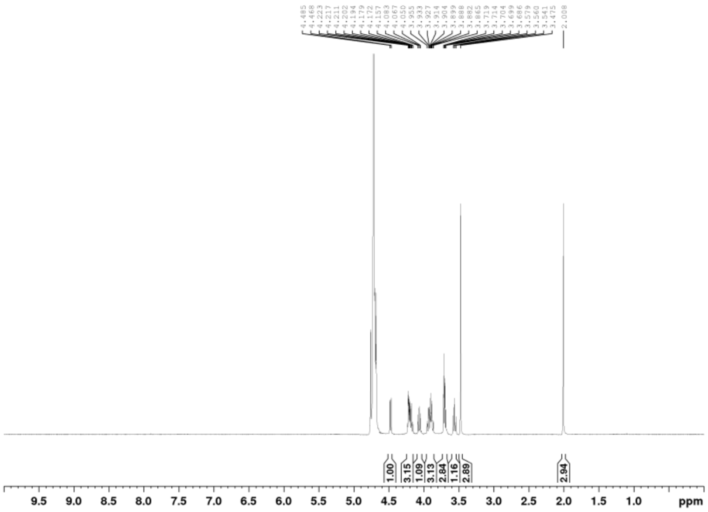

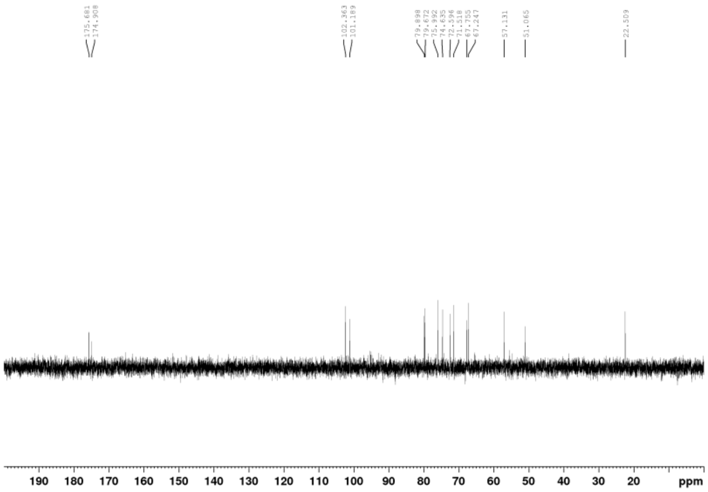

Protected dimer D1-A4

^{1}H NMR (500 MHz, $CDCl_3$) δ 7.94 (dd, *J* = 8.1, 0.9 Hz, 2H), 7.56 (t, *J* = 7.5 Hz, 1H), 7.42 (t, *J* = 7.8 Hz, 2H), 7.33 – 7.21 (m, 5H), 7.14 – 7.05 (m, 5H), 6.84 (d, *J* = 7.0 Hz, 1H), 5.47 (d, *J* = 3.3 Hz, 1H), 5.22 (dd, *J* = 8.3, 7.4 Hz, 1H), 4.84 (d, *J* = 8.4 Hz, 1H), 4.78 – 4.59 (m, 6H), 4.16 (dd, *J* = 11.6, 5.4 Hz, 1H), 4.07 (dd, *J* = 11.6, 7.2 Hz, 1H), 4.02 – 3.98 (m, 2H), 3.87 (t, *J* = 6.3 Hz, 1H), 3.76 (d, *J* = 6.0 Hz, 3H), 3.74 – 3.70 (m, 1H), 3.52 – 3.40 (m, 4H), 2.08 (s, 3H), 2.06 (s, 3H). ^{13}C NMR (500 MHz, $CDCl_3$) δ 170.5, 169.8, 168.4, 164.8, 162.0, 137.5, 137.3, 133.3, 129.8, 129.3, 128.4, 128.3, 128.2, 127.9, 127.9, 127.7, 100.4, 99.4, 91.9, 81.4, 78.8, 74.9, 74.6, 74.2, 73.1, 72.6, 71.2, 68.7, 62.3, 57.2, 56.2, 52.5, 20.6, 20.5. HRMS (ESI): *m/z*: calcd for $C_{41}H_{44}Cl_3NNaO_{15}$ [M + Na]: 918.1669; found: 918.1673.

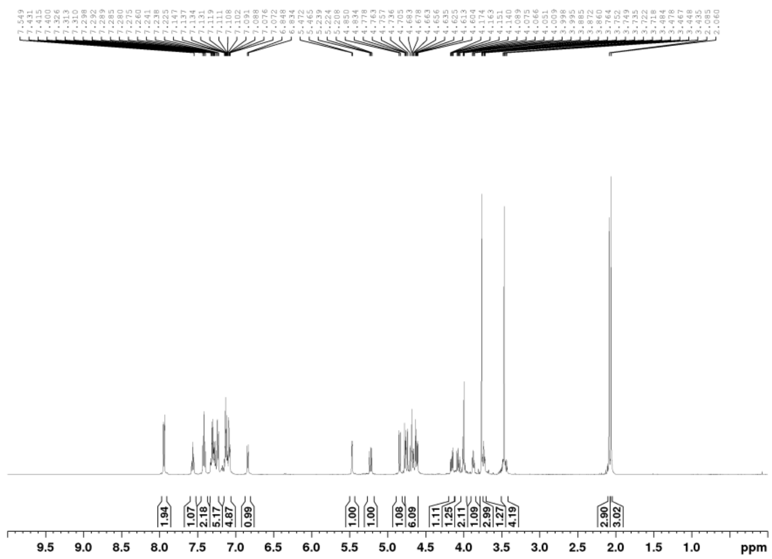

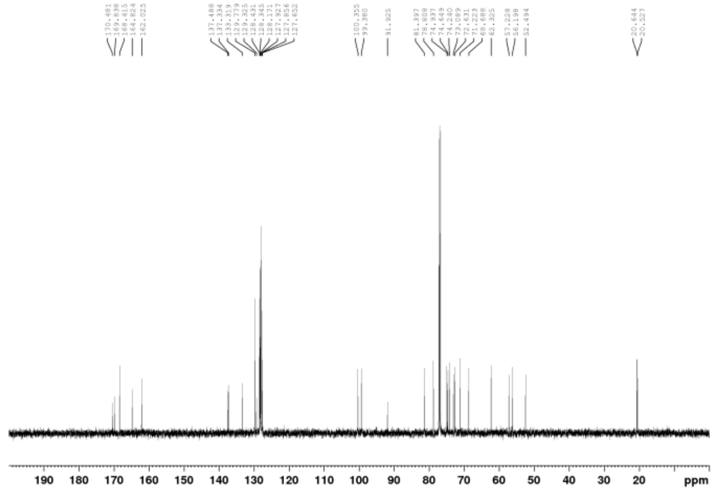

Compound 42d

^{1}H NMR (500 MHz, $CDCl_3$) δ 8.03 – 7.93 (m, 2H), 7.60 (t, *J* = 7.5 Hz, 1H), 7.46 (t, *J* = 7.8 Hz, 2H), 7.35 – 7.21 (m, 5H), 7.17 – 7.06 (m, 5H), 5.58 (d, *J* = 6.8 Hz, 1H), 5.40 (d, *J* = 3.4 Hz, 1H), 5.26 (dd, *J* = 8.7, 7.7 Hz, 1H), 4.97 (d, *J* = 8.3 Hz, 1H), 4.73 (ddd, *J* = 14.7, 12.3, 7.3 Hz, 4H), 4.64 (dd, *J* = 11.1, 3.8 Hz, 2H), 4.19 (dd, *J* = 11.6, 4.8 Hz, 1H), 4.06 – 3.95 (m, 3H), 3.86 (dd, *J* = 7.2, 5.1 Hz, 1H), 3.82 – 3.74 (m, 4H), 3.45 (s, 3H), 3.15 (dt, *J* = 10.6, 7.9 Hz, 1H), 2.07 (s, 3H), 2.06 (s, 3H), 1.36 (s, 3H). ^{13}C NMR (500 MHz, $CDCl_3$) δ 171.0, 170.5, 170.0, 168.3, 164.8, 137.5, 137.4, 133.4, 129.7, 129.4, 128.5, 128.3, 128.2, 127.9, 127.9, 127.6, 101.0, 99.6, 81.4, 79.0, 74.9, 74.8, 74.2, 73.2, 71.2, 68.6, 62.8, 56.9, 55.3, 52.5, 22.8, 20.7, 20.6. HRMS (ESI): *m/z*: calcd for $C_{41}H_{47}NNaO_{15}$ [M + Na]: 816.2838; found: 816.2835.

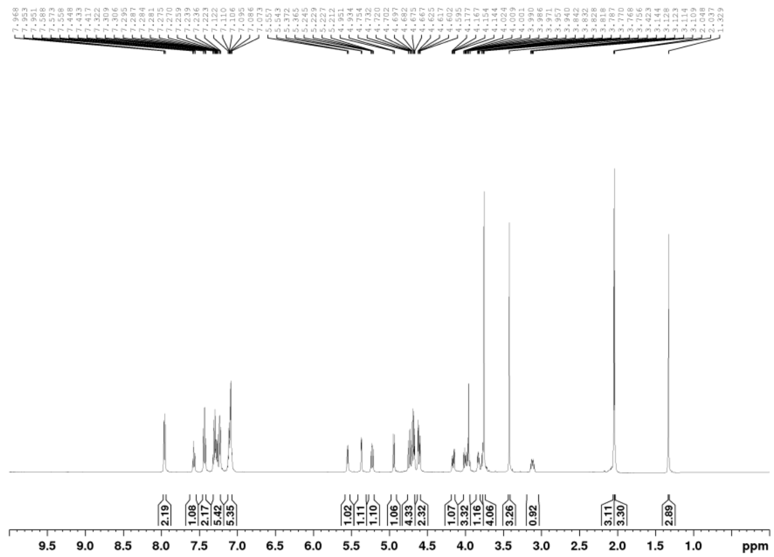

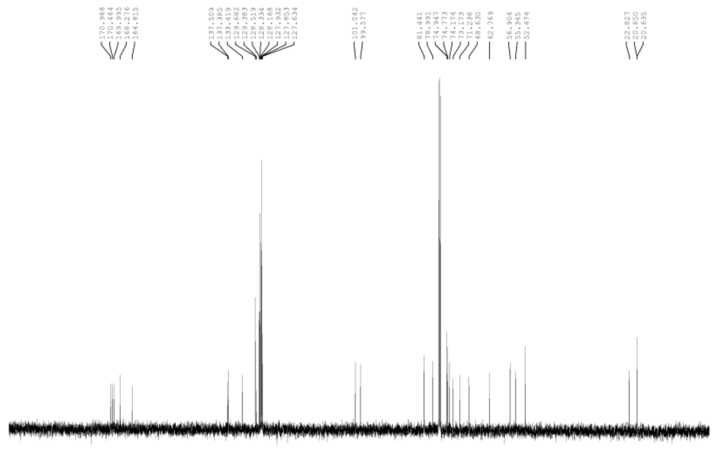

Compound 43d

^{1}H NMR (500 MHz, DMSO) δ 7.75 (d, *J* = 8.7 Hz, 1H), 7.34 – 7.21 (m, 9H), 5.45 (s, 1H), 5.13 (s, 1H), 4.81 (d, *J* = 11.7 Hz, 1H), 4.69 (dd, *J* = 11.1, 6.3 Hz, 2H), 4.61 (s, 1H), 4.53 (d, *J* = 10.9 Hz, 1H), 4.38 (d, *J* = 6.3 Hz, 1H), 4.28 (d, *J* = 8.4 Hz, 1H), 3.96 (s, 1H), 3.81 (d, *J* = 9.7 Hz, 1H), 3.74 (t, *J* = 7.4 Hz, 1H), 3.60 (dd, *J* = 18.8, 10.8 Hz, 3H), 3.46 – 3.42 (m, 2H), 3.30 (s, 3H), 1.80 (s, 3H). ^{13}C NMR (500 MHz, DMSO) δ 172.9, 170.0, 139.2, 138.9, 127.9, 127.9, 127.4, 127.1, 127.1, 104.5, 101.8, 83.7, 80.7, 80.4, 77.6, 74.9, 73.5, 73.0, 72.8, 66.5, 59.9, 55.3, 50.7, 23.1. HRMS (ESI): *m/z*: calcd for $C_{29}H_{36}NO_{12}$ [M – Na]: 590.2243; found: 590.2238.

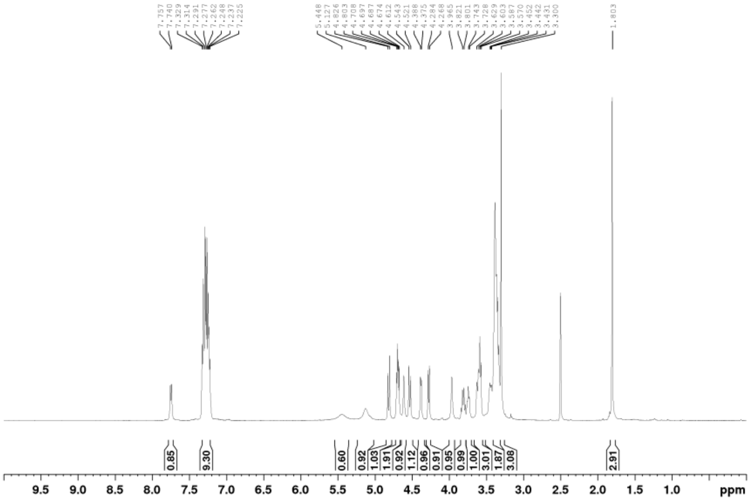

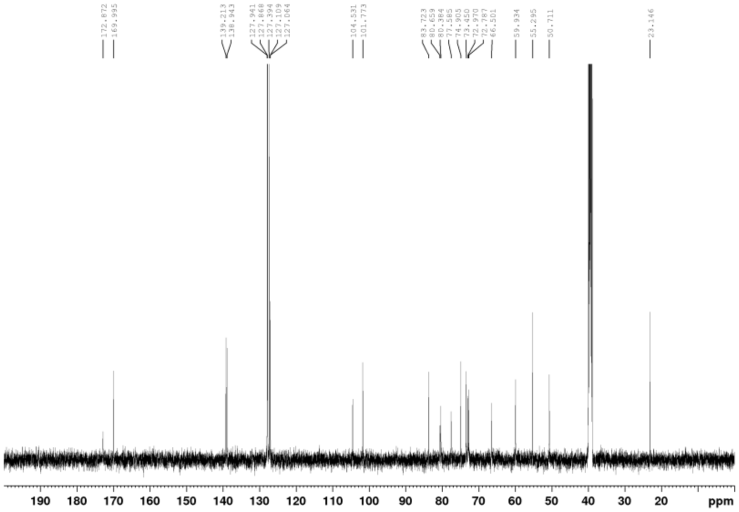

Compound 44d

^{1}H NMR (500 MHz, DMSO) δ 7.91 (d, *J* = 7.4 Hz, 1H), 7.35 (d, *J* = 6.8 Hz, 2H), 7.28 – 7.16 (m, 8H), 4.91 (d, *J* = 11.4 Hz, 1H), 4.74 (d, *J* = 8.2 Hz, 1H), 4.55 (dd, *J* = 18.5, 9.2 Hz, 3H), 4.48 (s, 2H), 4.44 (t, *J* = 6.2 Hz, 1H), 4.04 (d, *J* = 9.9 Hz, 1H), 3.88 (d, *J* = 10.8 Hz, 1H), 3.81 – 3.66 (m, 5H), 3.33 (s, 3H), 1.90 (s, 3H). ^{13}C NMR (500 MHz, DMSO) δ 172.6, 171.1, 138.9, 138.7, 128.0, 127.8, 127.7, 127.5, 127.1, 103.2, 100.5, 79.9, 78.6, 78.3, 78.2, 77.7, 74.8, 72.9, 72.2, 71.7, 66.6, 55.5, 52.3, 23.1. HRMS (ESI): *m/z*: calcd for $C_{29}H_{33}NNa_3O_{21}S_3$ [M + Na]: 896.0406; found: 896.0402.

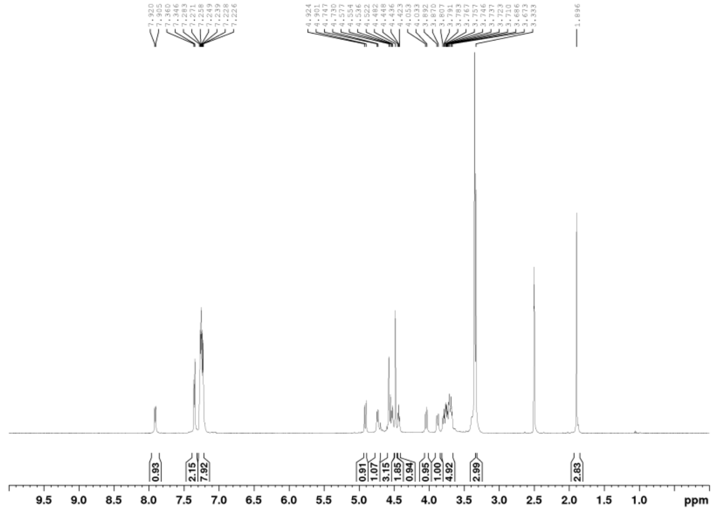

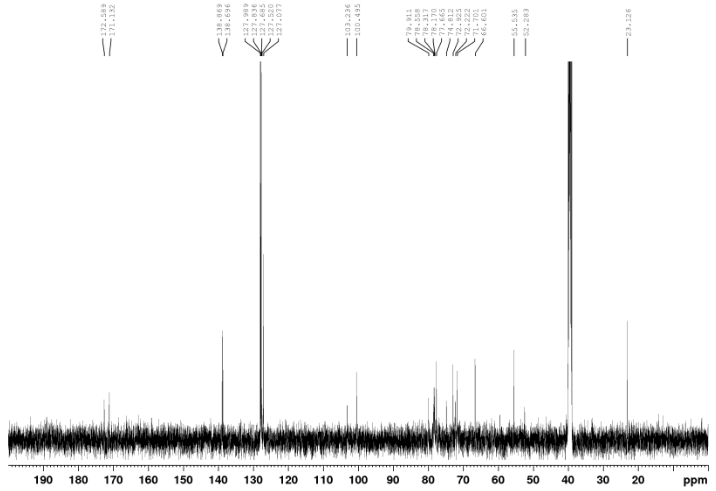

Compound 45d

^{1}H NMR (500 MHz, D_2O) δ 4.87 (d, *J* = 2.6 Hz, 1H), 4.62 (d, *J* = 8.0 Hz, 2H), 4.28 (dd, *J* = 11.4, 2.7 Hz, 1H), 4.20 – 4.09 (m, 3H), 4.03 (dd, *J* = 8.9, 2.7 Hz, 1H), 3.80 (dd, *J* = 10.8, 8.9 Hz, 1H), 3.70 – 3.57 (m, 3H), 3.49 (s, 3H), 2.00 (s, 3H). ^{13}C NMR (500 MHz, D_2O) δ 175.7, 174.9, 101.9, 101.8, 80.1, 76.3, 76.2, 76.1, 75.1, 72.5, 71.4, 68.1, 57.2, 52.3, 22.4. HRMS (ESI): *m/z*: calcd for $C_{15}H_{21}NNa_5O_{21}S_3$ [M + Na]: 761.9251; found: 761.9257.

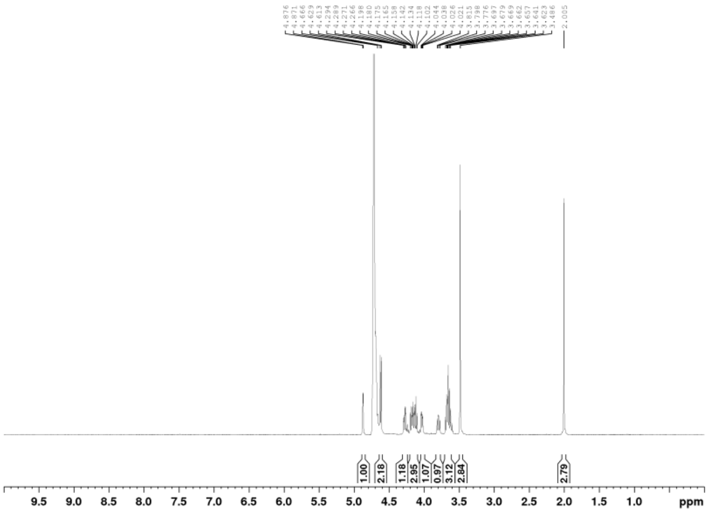

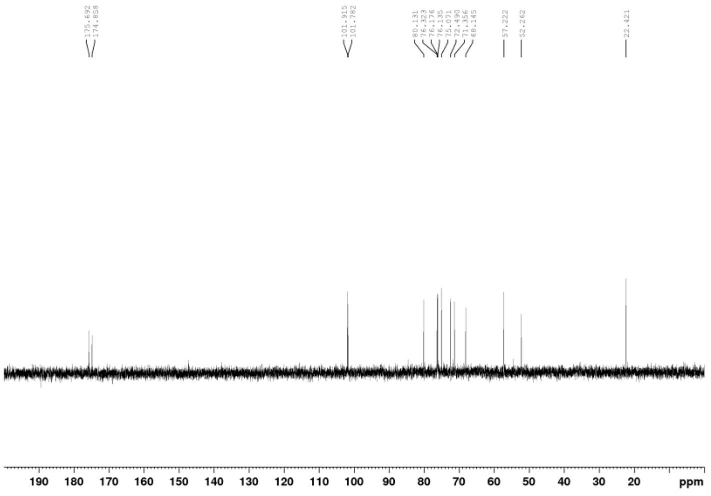

Protected dimer D2-A1

^{1}H NMR (500 MHz, $CDCl_3$) δ 7.94 – 7.84 (m, 4H), 7.54 – 7.43 (m, 2H), 7.44 – 7.37 (m, 2H), 7.40 – 7.23 (m, 12H), 7.18 – 7.13 (m, 3H), 7.09 (dd, *J* = 6.5, 2.9 Hz, 2H), 6.73 (d, *J* = 6.6 Hz, 1H), 5.72 – 5.60 (m, 1H), 5.44 (dd, *J* = 9.6, 7.8 Hz, 1H), 5.00 (d, *J* = 11.4 Hz, 1H), 4.91 (d, *J* = 7.7 Hz, 1H), 4.82 (d, *J* = 8.3 Hz, 1H), 4.71 (dd, *J* = 11.0, 2.8 Hz, 1H), 4.66 – 4.47 (m, 3H), 4.45 – 4.34 (m, 2H), 4.21 – 4.13 (m, 2H), 4.07 (d, *J* = 2.6 Hz, 1H), 3.78 – 3.67 (m, 4H), 3.59 – 3.44 (m, 3H), 3.43 (s, 3H). ^{13}C NMR (500 MHz, $CDCl_3$) δ 168.3, 165.4, 165.1, 162.3, 138.5, 136.9, 133.3, 129.9, 129.7, 129.0, 128.9, 128.4, 128.4, 128.3, 128.2, 128.0, 127.9, 127.8, 127.7, 127.6, 101.5, 99.2, 92.0, 77.4, 76.5, 75.3, 74.8, 74.7, 74.3, 73.9, 73.4, 73.3, 71.8, 68.7, 57.2, 56.6, 52.6. HRMS (ESI): *m/z*: calcd for $C_{51}H_{49}Cl_3NO_{14}$ [M – H]: 1004.2224; found: 1004.2217.

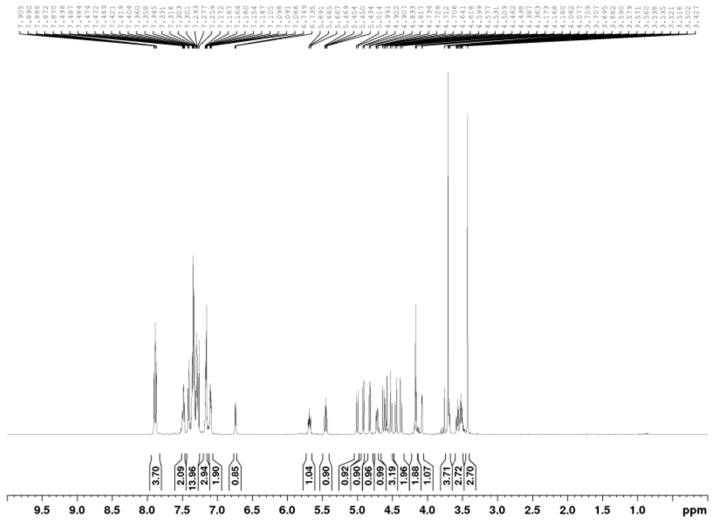

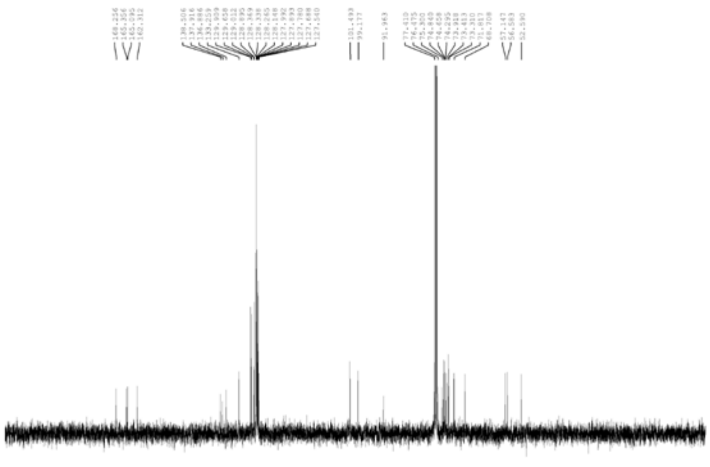

Compound 42e

^{1}H NMR (500 MHz, $CDCl_3$) δ 8.04 – 7.85 (m, 4H), 7.59 – 7.45 (m, 2H), 7.43 – 7.24 (m, 14H), 7.20 – 7.14 (m, 3H), 7.15 – 7.07 (m, 2H), 5.74 (dt, *J* = 9.2, 4.4 Hz, 1H), 5.43 (dd, *J* = 9.7, 7.9 Hz, 1H), 5.26 (d, *J* = 6.7 Hz, 1H), 4.97 (d, *J* = 11.5 Hz, 1H), 4.87 (dd, *J* = 8.0, 2.3 Hz, 2H), 4.76 (dd, *J* = 10.9, 2.8 Hz, 1H), 4.67 – 4.49 (m, 3H), 4.39 (dd, *J* = 40.2, 11.7 Hz, 2H), 4.17 (t, *J* = 5.2 Hz, 2H), 4.02 (d, *J* = 2.5 Hz, 1H), 3.77 – 3.62 (m, 4H), 3.55 (dd, *J* = 9.5, 5.7 Hz, 1H), 3.46 (dd, *J* = 9.5, 6.5 Hz, 1H), 3.40 (s, 3H), 3.21 – 3.14 (m, 1H), 1.47 (s, 3H). ^{13}C NMR (500 MHz, $CDCl_3$) δ 171.3, 168.3, 165.4, 164.9, 138.8, 138.1, 137.0, 133.4, 133.3, 129.7, 129.6, 129.4, 129.1, 128.9, 128.6, 128.4, 128.3, 128.3, 128.1, 128.0, 127.9, 127.7, 127.6, 127.5, 102.4, 99.6, 78.4, 77.4, 75.3, 74.7, 74.5, 74.2, 74.00, 73.3, 73.2, 72.1, 69.0, 56.8, 55.8, 52.6, 23.3. HRMS (ESI): *m/z*: calcd for $C_{51}H_{53}NNaO_{14}$ [M + Na]: 926.3358; found: 926.3355.

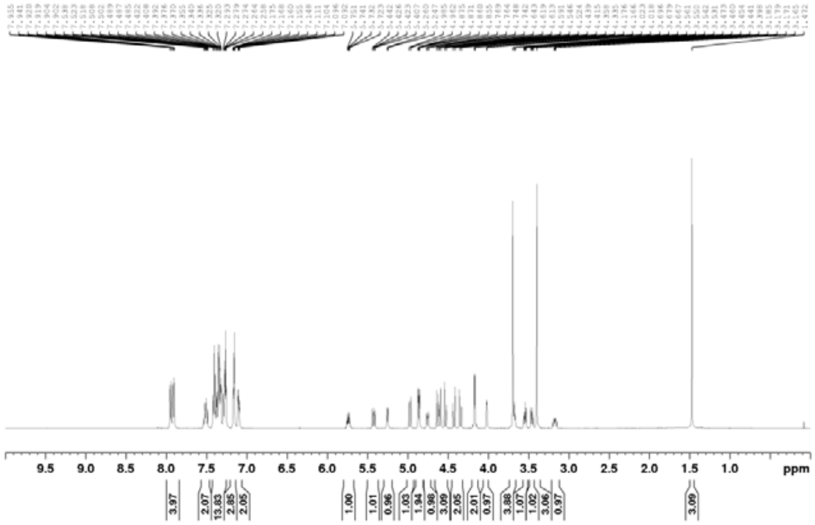

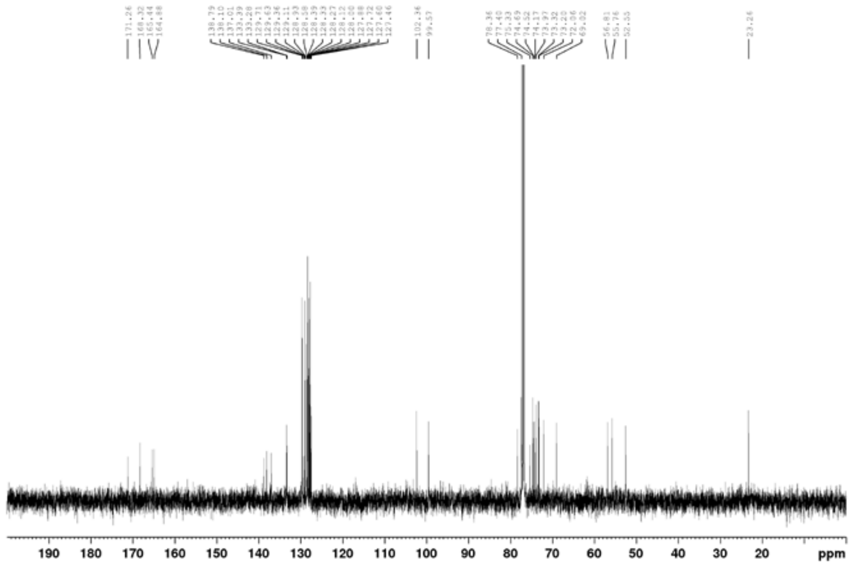

Compound 43e

^{1}H NMR (500 MHz, DMSO) δ 7.72 (d, *J* = 7.1 Hz, 1H), 7.37 – 7.21 (m, 14H), 4.82 (dd, *J* = 23.6, 11.4 Hz, 2H), 4.60 (d, *J* = 11.2 Hz, 1H), 4.47 (d, *J* = 11.6 Hz, 1H), 4.42 – 4.33 (m, 3H), 4.30 (d, *J* = 7.2 Hz, 1H), 3.87 – 3.76 (m, 3H), 3.71 (d, *J* = 9.5 Hz, 1H), 3.61 (t, *J* = 5.9 Hz, 1H), 3.51 (t, *J* = 9.3 Hz, 1H), 3.45 – 3.34 (m, 3H), 3.33 – 3.30 (m, 1H), 3.28 (s, 3H), 3.12 (t, *J* = 8.3 Hz, 1H), 1.80 (s, 3H). ^{13}C NMR (500 MHz, DMSO) δ 170.8, 170.0, 139.0, 138.9, 138.2, 128.3, 128.2, 128.1, 127.9, 127.6, 127.4, 127.3, 127.1, 104.9, 101.6, 80.3, 80.1, 75.8, 75.3, 73.9, 73.7, 73.5, 72.6, 72.2, 68.8, 60.00, 55.5, 51.3, 23.1. HRMS (ESI): *m/z*: calcd for $C_{36}H_{42}NO_{12}$ [M – H]: 680.2712; found: 680.2716.

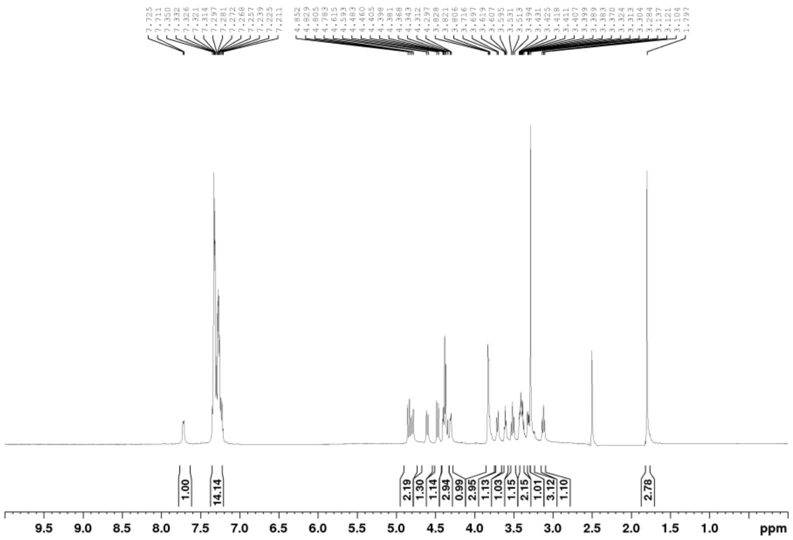

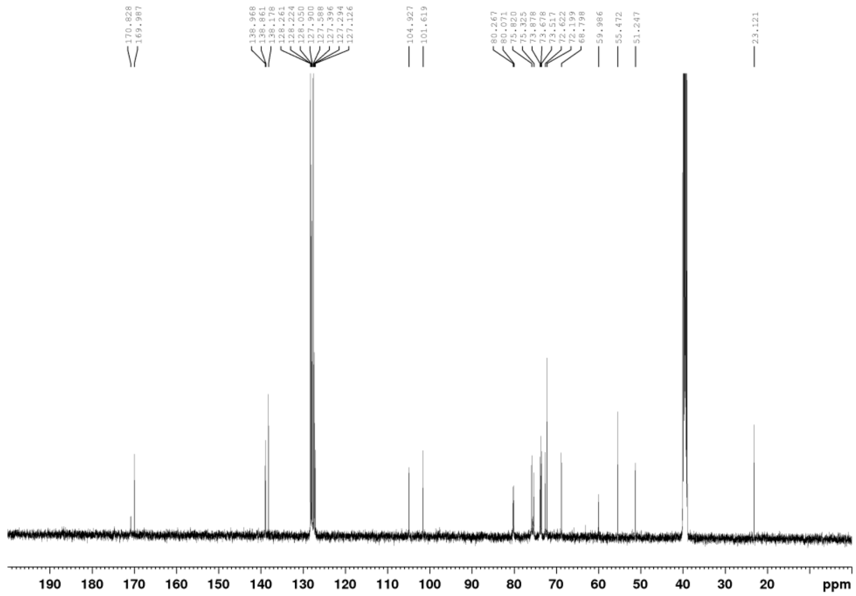

Compound 44e

^{1}H NMR (500 MHz, DMSO) δ 7.78 (d, *J* = 8.3 Hz, 1H), 7.44 – 7.14 (m, 15H), 5.11 (d, *J* = 11.8 Hz, 1H), 5.00 (d, *J* = 2.9 Hz, 1H), 4.79 (d, *J* = 11.1 Hz, 1H), 4.61 – 4.53 (m, 2H), 4.52 – 4.42 (m, 4H), 4.34 (d, *J* = 8.3 Hz, 1H), 4.29 – 4.22 (m, 1H), 4.14 (s, 1H), 4.03 (d, *J* = 11.5 Hz, 1H), 3.78 – 3.70 (m, 2H), 3.63 (t, *J* = 5.5 Hz, 1H), 3.54 (t, *J* = 8.4 Hz, 1H), 3.48 (dd, *J* = 10.0, 4.4 Hz, 1H), 3.27 (s, 3H), 1.83 (s, 3H). ^{13}C NMR (500 MHz, DMSO) δ 173.2, 170.1, 139.7, 139.3, 138.3, 128.2, 128.0, 127.8, 127.8, 127.7, 127.6, 127.4, 126.8, 102.0, 100.4, 77.8, 77.6, 76.8, 76.7, 76.5, 76.0, 74.0, 73.6, 72.2, 71.4, 69.6, 55.4, 51.4, 23.1. HRMS (ESI): *m/z*: calcd for $C_{36}H_{41}NNa_3O_{18}S_2$ [M + H]: 908.1453; found: 908.1450.

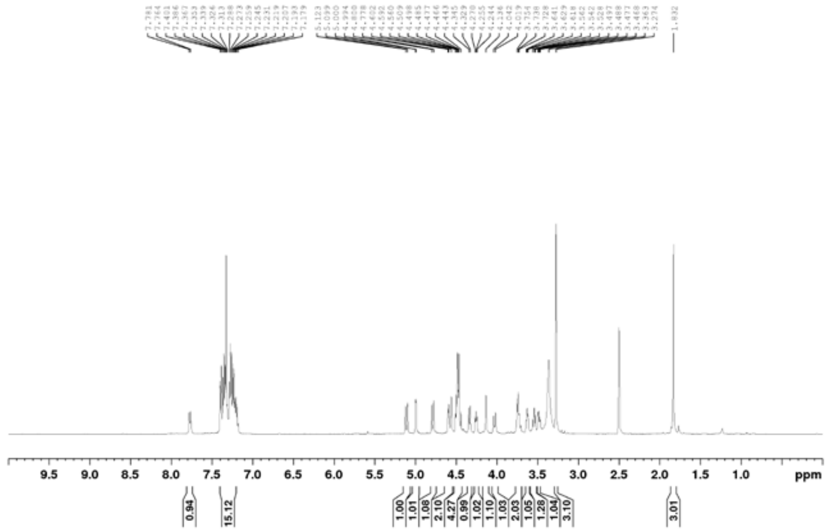

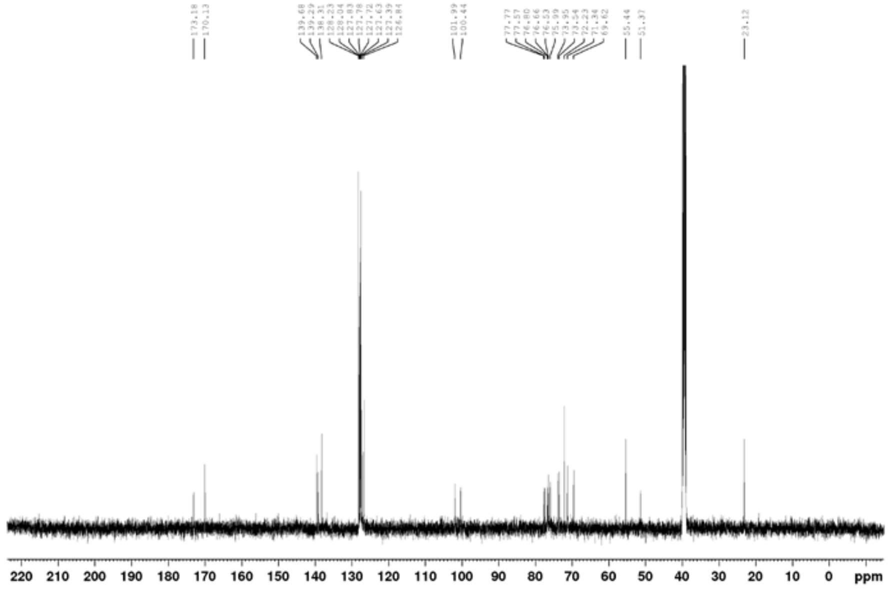

Compound 45e

^{1}H NMR (500 MHz, D_2O) δ 4.83 (d, *J* = 6.5 Hz, 1H), 4.44 (d, *J* = 8.4 Hz, 1H), 4.39 – 4.32 (m, 1H), 4.24 (t, *J* = 6.8 Hz, 1H), 4.14 (d, *J* = 2.8 Hz, 1H), 3.81 (dddd, *J* = 24.4, 9.7, 7.6, 2.6 Hz, 6H), 3.63 (dd, *J* = 7.4, 4.7 Hz, 1H), 3.43 (s, 3H), 1.98 (s, 3H). ^{13}C NMR (500 MHz, D_2O) δ 175.6, 174.9, 102.4, 100.7, 81.1, 79.7, 77.2, 76.1, 74.8, 70.4, 67.2, 61.1, 57.0, 51.2, 22.5. HRMS (ESI): *m/z*: calcd for $C_{15}H_{22}NNa_2O_{18}S_2$ [M – Na]: 614.0079; found: 614.0077.

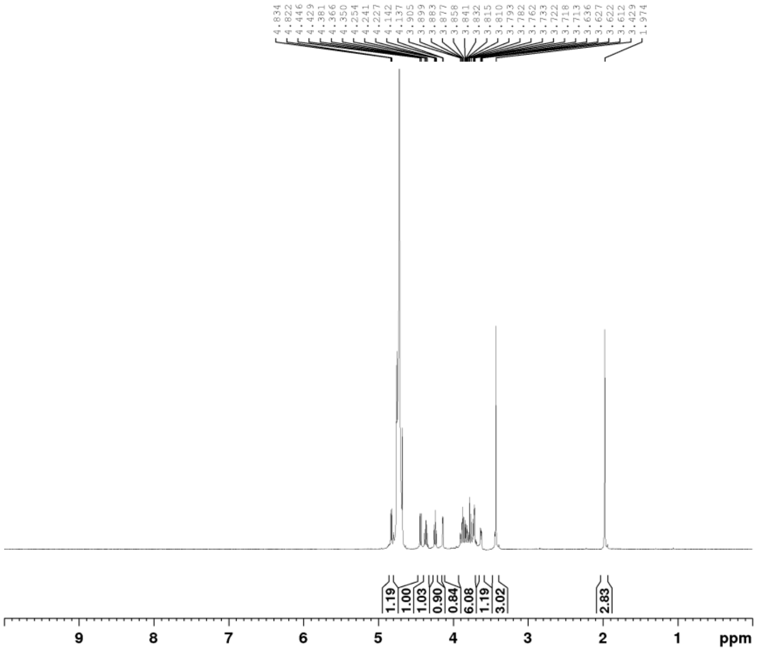

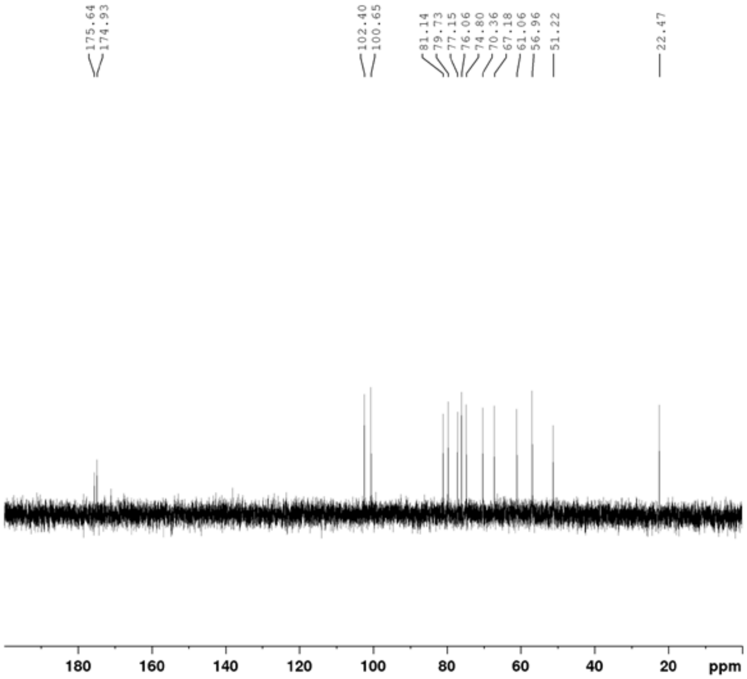

Protected dimer D2-A2

^{1}H NMR (500 MHz, $CDCl_3$) δ 7.95 – 7.81 (m, 4H), 7.48 (ddd, *J* = 7.3, 3.5, 1.4 Hz, 2H), 7.37 – 7.31 (m, 8H), 7.30 – 7.25 (m, 1H), 7.16 – 7.12 (m, 3H), 7.09 (dd, *J* = 6.7, 2.9 Hz, 2H), 6.77 (d, *J* = 7.0 Hz, 1H), 5.63 (t, *J* = 9.1 Hz, 1H), 5.57 (d, *J* = 3.4 Hz, 1H), 5.33 (dd, *J* = 9.1, 7.4 Hz, 1H), 4.89 (dd, *J* = 30.2, 7.8 Hz, 2H), 4.71 (dd, *J* = 10.8, 3.5 Hz, 1H), 4.55 (dt, *J* = 23.4, 11.4 Hz, 4H), 4.22 (t, *J* = 9.3 Hz, 1H), 4.13 (d, *J* = 9.5 Hz, 1H), 3.86 (t, *J* = 6.0 Hz, 1H), 3.75 (s, 3H), 3.58 (dd, *J* = 5.9, 2.3 Hz, 2H), 3.53 – 3.44 (m, 4H), 2.12 (s, 3H). ^{13}C NMR (500 MHz, $CDCl_3$) δ 169.8, 168.0, 165.3, 164.9, 162.0, 137.8, 137.0, 133.2, 133.1, 129.8, 129.6, 129.1, 129.0, 128.3, 128.3, 128.2, 127.9, 127.7, 127.7, 127.6, 100.5, 99.5, 91.9, 77.0, 74.6, 74.4, 74.1, 73.5, 73.3, 72.8, 72.0, 69.3, 68.7, 57.2, 56.3, 52.5, 20.6. HRMS (ESI): *m/z*: calcd for $C_{46}H_{46}Cl_3NNaO_{15}$ [M + Na]: 980.1825; found: 980.1820.

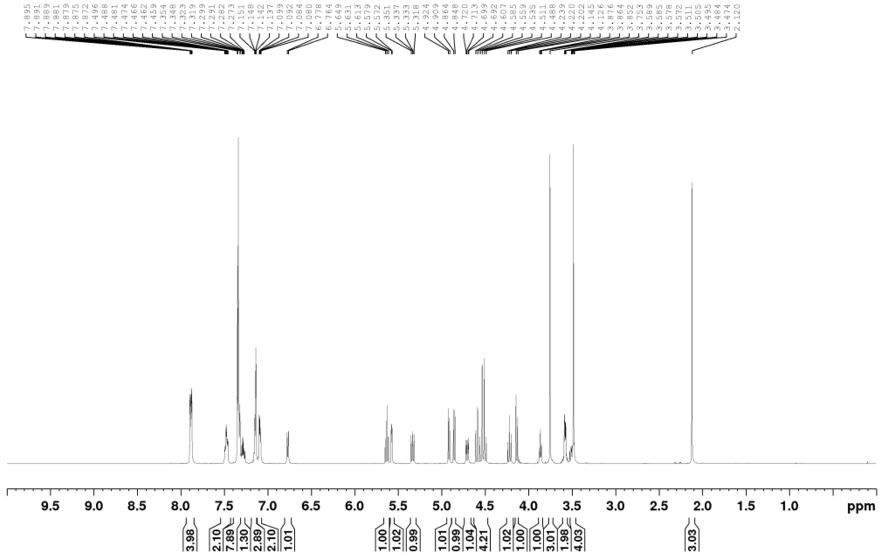

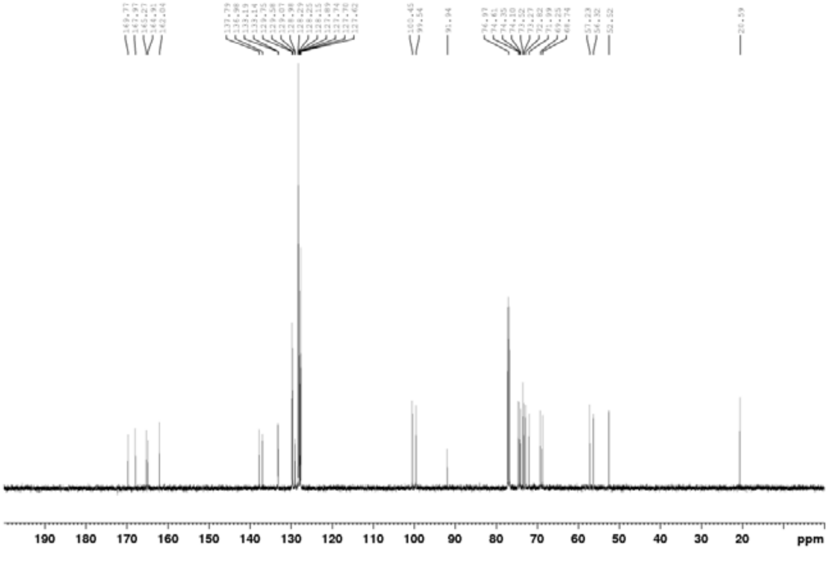

Compound 42f

^{1}H NMR (500 MHz, $CDCl_3$) δ 8.02 – 7.83 (m, 4H), 7.51 (dd, *J* = 13.7, 7.1 Hz, 2H), 7.41 – 7.22 (m, 9H), 7.18 – 7.12 (m, 3H), 7.08 (dt, *J* = 5.9, 3.1 Hz, 2H), 5.65 (t, *J* = 9.2 Hz, 1H), 5.46 (d, *J* = 3.4 Hz, 1H), 5.40 – 5.21 (m, 2H), 4.95 (d, *J* = 8.3 Hz, 1H), 4.80 (d, *J* = 7.5 Hz, 1H), 4.73 (dd, *J* = 10.7, 3.4 Hz, 1H), 4.60 – 4.45 (m, 4H), 4.18 (t, *J* = 9.3 Hz, 1H), 4.09 (d, *J* = 9.6 Hz, 1H), 3.90 – 3.80 (m, 1H), 3.77 (d, *J* = 7.8 Hz, 3H), 3.55 (ddd, *J* = 17.0, 10.3, 5.9 Hz, 2H), 3.46 (d, *J* = 7.8 Hz, 3H), 3.11 (dt, *J* = 10.6, 7.8 Hz, 1H), 2.09 (s, 3H), 1.41 (s, 3H). ^{13}C NMR (500 MHz, $CDCl_3$) δ 171.0, 170.1, 168.1, 165.4, 165.0, 138.0, 137.1, 133.5, 133.3, 129.7, 129.3, 128.6, 128.4, 128.3, 128.0, 127.9, 127.7, 127.7, 101.2, 99.8, 77.1, 75.2, 74.7, 74.3, 74.2, 73.6, 72.9, 72.0, 69.4, 69.3, 57.2, 55.7, 52.7, 23.0, 20.9. HRMS (ESI): *m/z*: calcd for $C_{46}H_{49}NNaO_{15}$ [M + Na]: 878.2994; found: 878.2998.

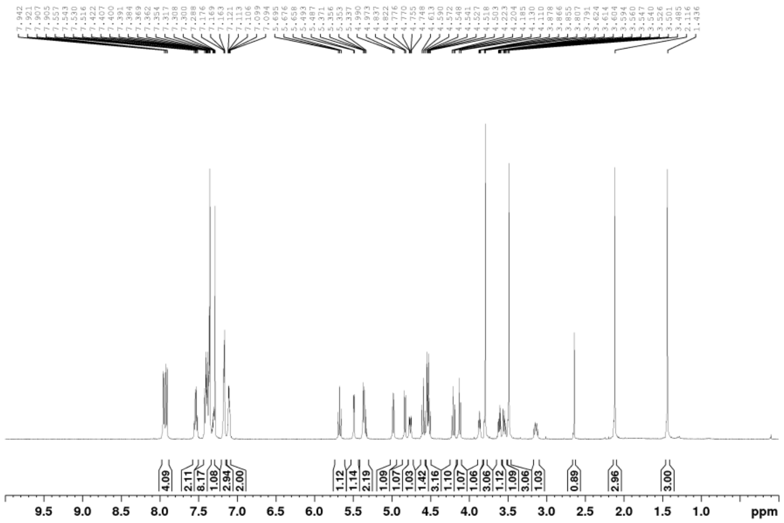

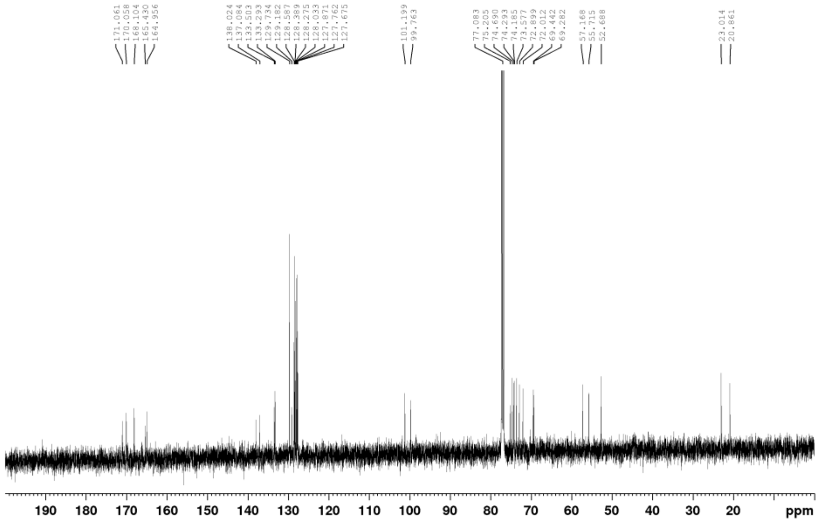

Compound 43f

^{1}H NMR (500 MHz, DMSO) δ 7.75 (d, *J* = 8.7 Hz, 1H), 7.38 – 7.15 (m, 10H), 4.65 (s, 2H), 4.49 (d, *J* = 13.5 Hz, 3H), 4.34 (d, *J* = 7.3 Hz, 1H), 4.28 (d, *J* = 8.4 Hz, 1H), 3.89 – 3.78 (m, 2H), 3.67 – 3.43 (m, 6H), 3.30 (s, 3H), 3.14 (t, *J* = 7.1 Hz, 1H), 1.79 (s, 3H). ^{13}C NMR (500 MHz, DMSO) δ 173.7, 170.1, 139.5, 138.5, 128.2, 128.2, 127.8, 127.7, 127.7, 127.5, 127.4, 126.9, 104.3, 101.6, 80.9, 79.6, 75.4, 73.3, 73.1, 72.7, 72.3, 69.5, 67.6, 56.0, 55.4, 50.6, 23.1. HRMS (ESI): *m/z*: calcd for $C_{29}H_{36}NO_{12}$ [M – H]: 590.2243; found: 590.2248.

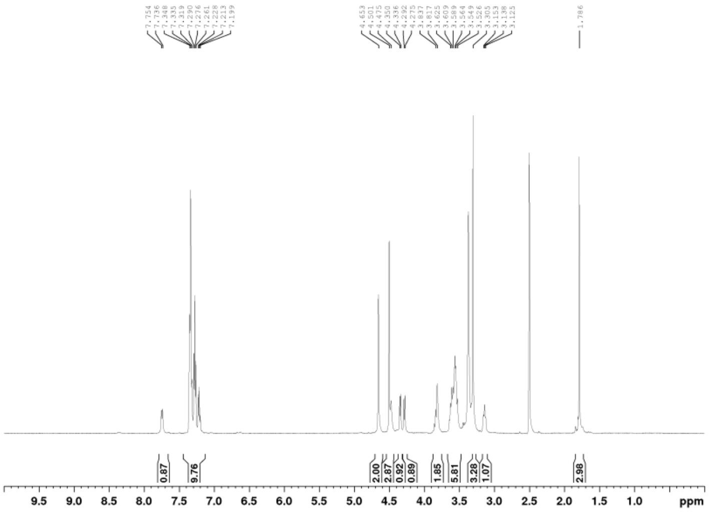

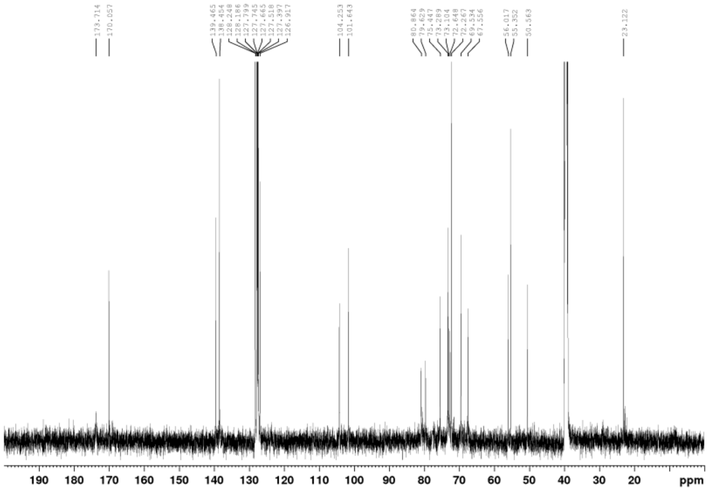

Compound 44f

^{1}H NMR (500 MHz, DMSO) δ 7.69 (d, *J* = 7.9 Hz, 1H), 7.41 (d, *J* = 7.2 Hz, 2H), 7.36 – 7.19 (m, 8H), 4.86 (s, 2H), 4.74 (d, *J* = 11.2 Hz, 1H), 4.59 (s, 1H), 4.53 (d, *J* = 4.7 Hz, 1H), 4.49 (s, 2H), 4.44 (d, *J* = 11.2 Hz, 1H), 4.38 (d, *J* = 8.0 Hz, 1H), 4.11 (s, 1H), 3.92 (d, *J* = 3.4 Hz, 1H), 3.81 (d, *J* = 10.6 Hz, 1H), 3.77 – 3.61 (m, 3H), 3.58 (dd, *J* = 10.5, 7.8 Hz, 1H), 3.32 (s, 3H), 1.89 (s, 3H). ^{13}C NMR (500 MHz, DMSO) δ 173.5, 170.9, 138.8, 138.6, 128.2, 127.9, 127.9, 127.4, 127.3, 127.2, 101.8, 101.6, 79.0, 77.4, 76.7, 75.9, 73.4, 73.2, 72.1, 70.5, 70.3, 55.5, 51.1, 23.1. HRMS (ESI): *m/z*: calcd for $C_{29}H_{33}NNa_5O_{21}S_3$ [M + Na]: 942.0190; found: 942.0183.

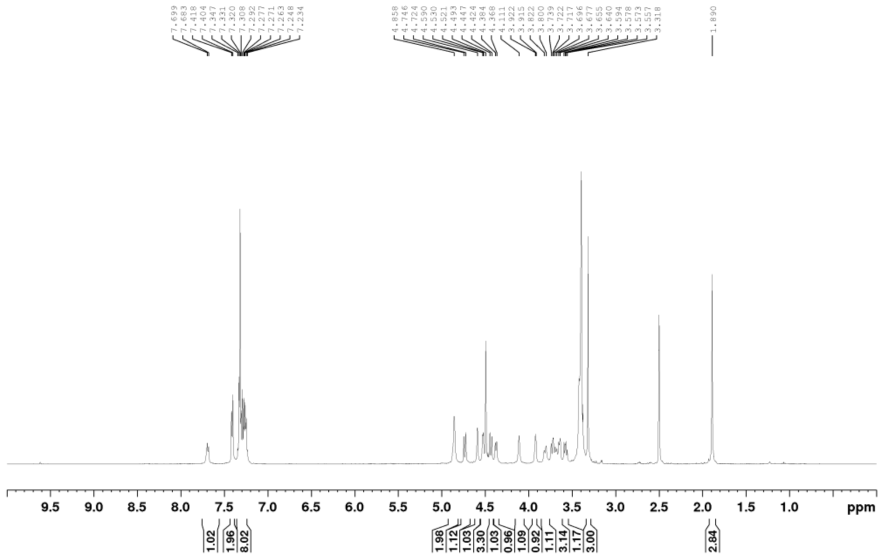

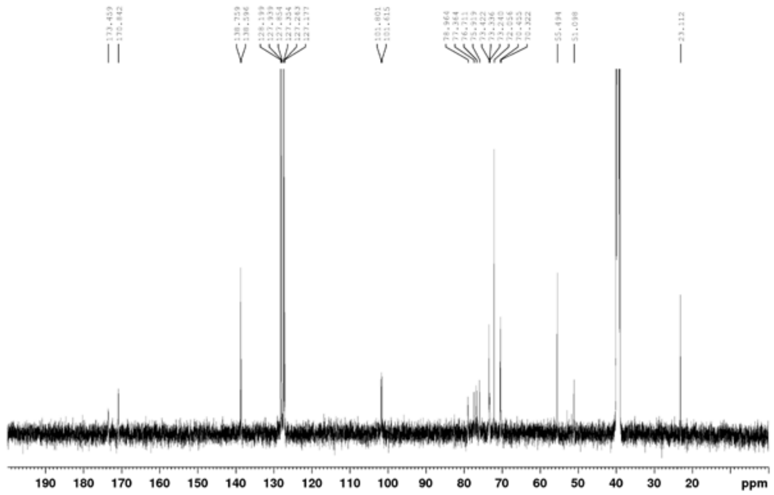

Compound 45f

^{1}H NMR (500 MHz, D_2O) δ 4.84 (d, *J* = 2.7 Hz, 1H), 4.38 (dd, *J* = 13.3, 5.5 Hz, 1H), 4.27 (t, *J* = 7.2 Hz, 1H), 4.20 – 4.12 (m, 1H), 3.92 (t, *J* = 9.0 Hz, 1H), 3.85 – 3.73 (m, 5H), 3.69 (dd, *J* = 11.1, 8.8 Hz, 1H), 3.46 (s, 3H), 2.00 (s, 3H). ^{13}C NMR (500 MHz, D_2O) δ 175.4, 175.0, 101.9, 101.8, 81.7, 77.4, 76.5, 76.4, 76.1, 74.7, 70.3, 61.1, 57.1, 52.6, 22.6. HRMS (ESI): *m/z*: calcd for $C_{15}H_{21}NNa_5O_{21}S_3$ [M + Na]: 761.9251; found: 761.9256.

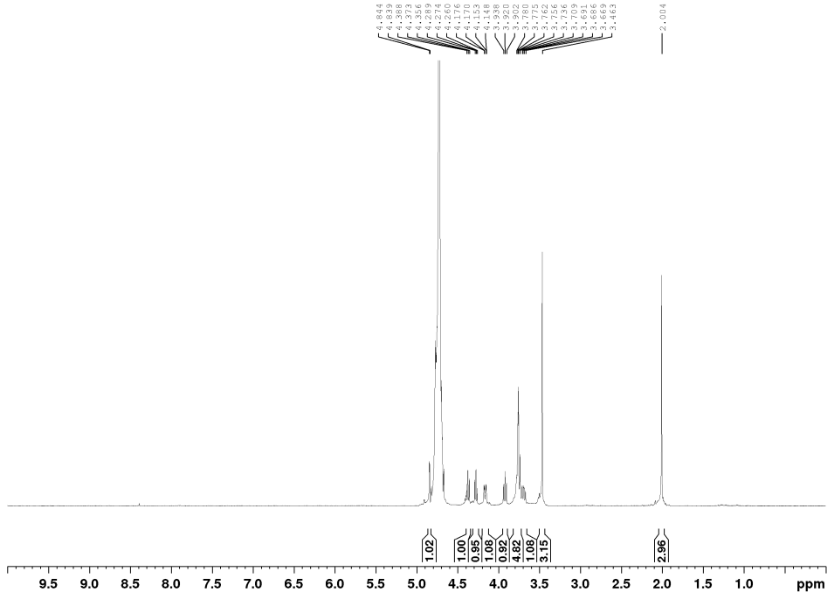

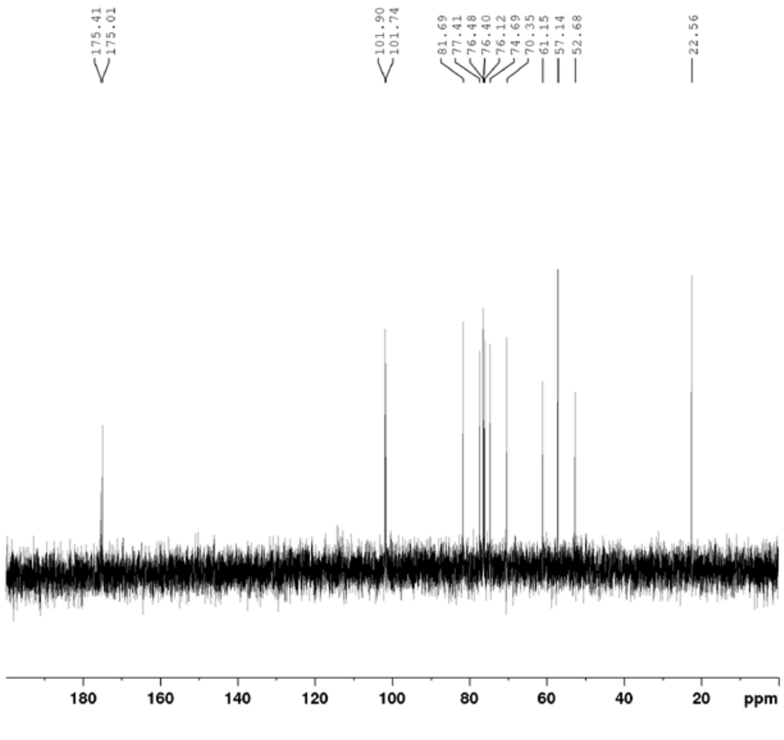

Protected dimer D2-A3

^{1}H NMR (500 MHz, $CDCl_3$) δ 7.90 (dd, *J* = 12.5, 4.4 Hz, 4H), 7.55 – 7.42 (m, 4H), 7.40 – 7.28 (m, 7H), 7.20 – 7.14 (m, 3H), 7.10 (dd, *J* = 6.5, 2.9 Hz, 2H), 6.81 (d, *J* = 6.5 Hz, 1H), 5.70 (dd, *J* = 9.3, 8.6 Hz, 1H), 5.48 (dd, *J* = 9.6, 8.0 Hz, 1H), 5.02 (d, *J* = 11.4 Hz, 1H), 4.92 (d, *J* = 7.8 Hz, 1H), 4.82 (d, *J* = 8.3 Hz, 1H), 4.74 (dd, *J* = 10.9, 2.9 Hz, 1H), 4.69 (d, *J* = 11.4 Hz, 1H), 4.56 (dd, *J* = 29.3, 11.1 Hz, 2H), 4.19 (dt, *J* = 11.3, 8.5 Hz, 3H), 4.00 (dd, *J* = 11.3, 5.9 Hz, 2H), 3.79 (s, 3H), 3.69 (t, *J* = 6.4 Hz, 1H), 3.56 – 3.47 (m, 1H), 3.43 (s, 3H), 1.98 (s, 3H). ^{13}C NMR (500 MHz, $CDCl_3$) δ 170.4, 168.3, 165.3, 165.1, 162.4, 138.0, 136.8, 133.3, 133.3, 129.9, 129.6, 129.0, 128.9, 128.4, 128.3, 128.3, 128.2, 128.0, 127.9, 127.7, 101.5, 99.1, 91.9, 77.4, 76.3, 74.7, 74.6, 74.3, 73.8, 71.9, 71.8, 62.7, 57.1, 56.4, 52.6, 20.6. HRMS (ESI): *m/z*: calcd for $C_{46}H_{45}Cl_3NO_{15}$ [M – H]: 956.1860; found: 956.1867.

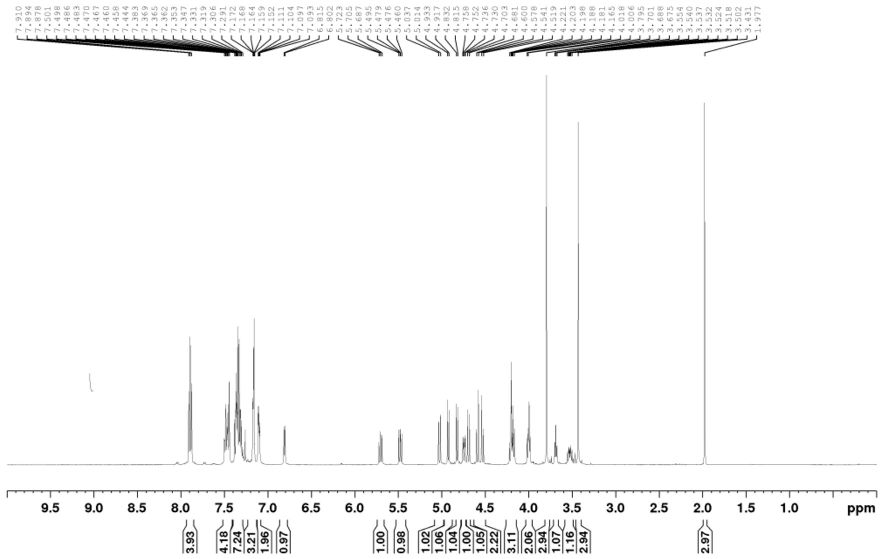

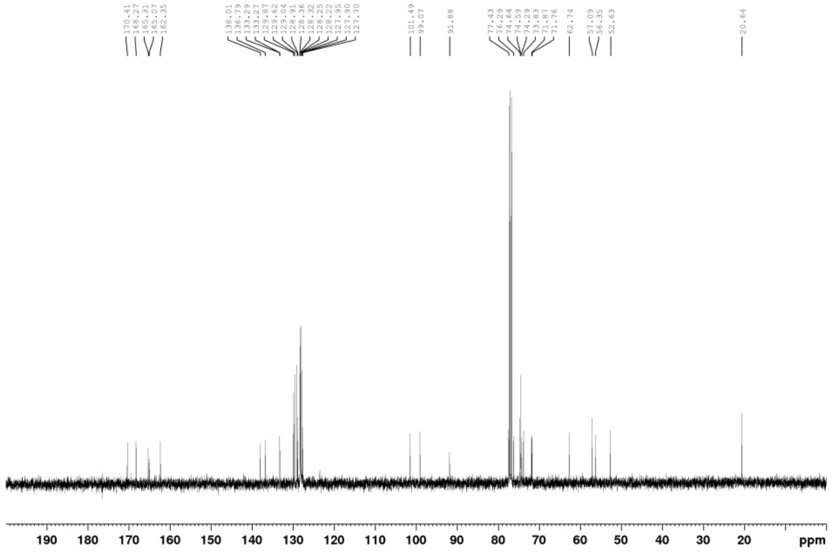

Compound 42g

^{1}H NMR (500 MHz, CDCl3) δ 7.93 (dd, J = 16.6, 7.8 Hz, 4H), 7.51 (dt, J = 11.0, 7.5 Hz, 2H), 7.37 (tdd, J = 25.2, 21.1, 7.2 Hz, 9H), 7.16 (dd, J = 4.9, 1.7 Hz, 3H), 7.12 – 7.08 (m, 2H), 5.81 – 5.69 (m, 1H), 5.44 (dd, J = 9.4, 8.2 Hz, 1H), 5.27 (d, J = 6.6 Hz, 1H), 4.98 (d, J = 11.6 Hz, 1H), 4.86 (dd, J = 14.3, 8.0 Hz, 2H), 4.77 (dd, J = 11.0, 2.6 Hz, 1H), 4.68 (d, J = 11.6 Hz, 1H), 4.57 (dd, J = 28.7, 11.1 Hz, 2H), 4.19 (d, J = 3.9 Hz, 2H), 4.15 – 4.10 (m, 1H), 3.94 (dd, J = 9.9, 4.3 Hz, 2H), 3.78 (s, 3H), 3.65 (t, J = 6.2 Hz, 1H), 3.39 (s, 3H), 3.18 (dt, J = 10.7, 7.7 Hz, 1H), 1.94 (s, 3H), 1.48 (s, 3H). ^{13}C NMR (500 MHz, $CDCl_3$) δ 171.4, 170.5, 168.4, 165.5, 164.9, 138.4, 137.0, 133.5, 133.4, 129.8, 129.7, 129.4, 129.2, 128.6, 128.4, 128.3, 128.3, 128.0, 128.0, 127.7, 102.5, 99.6, 78.3, 77.5, 74.8, 74.7, 74.4, 74.3, 74.00, 72.1, 71.8, 63.1, 56.8, 55.7, 52.7, 23.3, 20.7. HRMS (ESI): *m/z*: calcd for $C_{46}H_{49}NNaO_{15}$ [M + Na]: 878.2994; found: 878.2996.

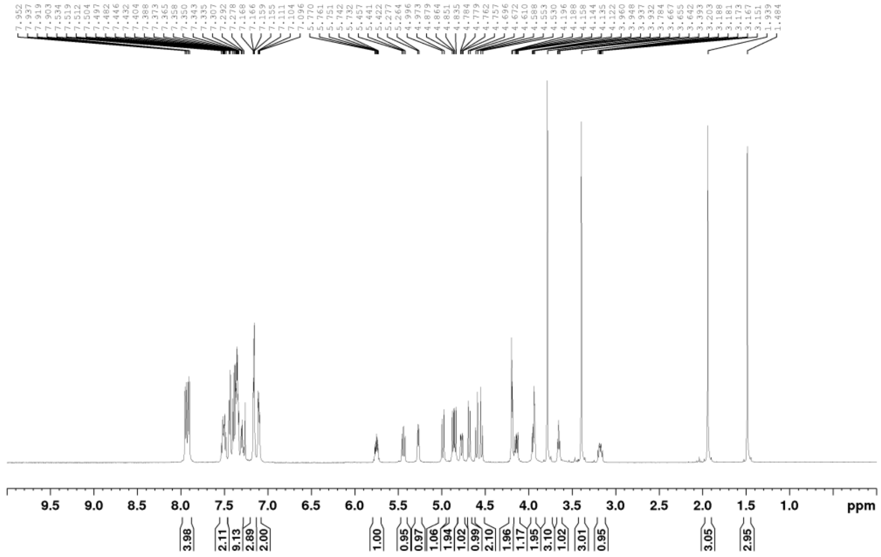

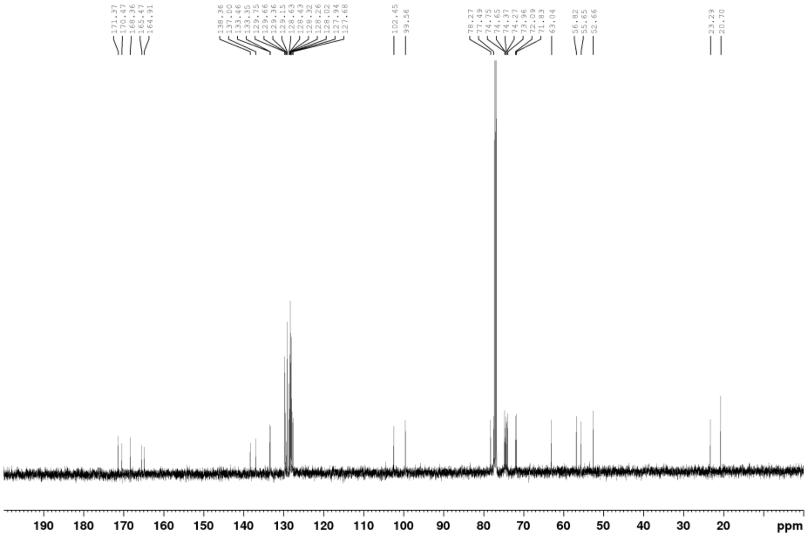

Compound 43g

^{1}H NMR (500 MHz, DMSO) δ 7.71 (d, *J* = 7.8 Hz, 1H), 7.42 – 7.12 (m, 10H), 4.86 (d, *J* = 11.5 Hz, 1H), 4.79 (d, *J* = 11.2 Hz, 1H), 4.58 (d, *J* = 11.2 Hz, 1H), 4.52 (d, *J* = 11.5 Hz, 1H), 4.38 (d, *J* = 7.7 Hz, 1H), 4.28 (d, *J* = 4.2 Hz, 1H), 3.86 (s, 1H), 3.81 (d, *J* = 7.4 Hz, 2H), 3.70 (d, *J* = 9.5 Hz, 2H), 3.49 – 3.34 (m, 4H), 3.29 (s, 3H), 3.11 (t, *J* = 8.3 Hz, 1H), 1.79 (s, 3H). ^{13}C NMR (500 MHz, DMSO) δ 170.9, 170.1, 139.2, 139.0, 128.1, 128.0, 127.6, 127.3, 127.2, 105.0, 101.7, 80.5, 80.0, 75.8, 75.4, 75.3, 74.7, 73.9, 73.6, 60.1, 55.5, 51.4, 23.2. HRMS (ESI): *m/z*: calcd for $C_{29}H_{37}NNaO_{12}$ [M – H]: 614.2208; found: 614.2201.

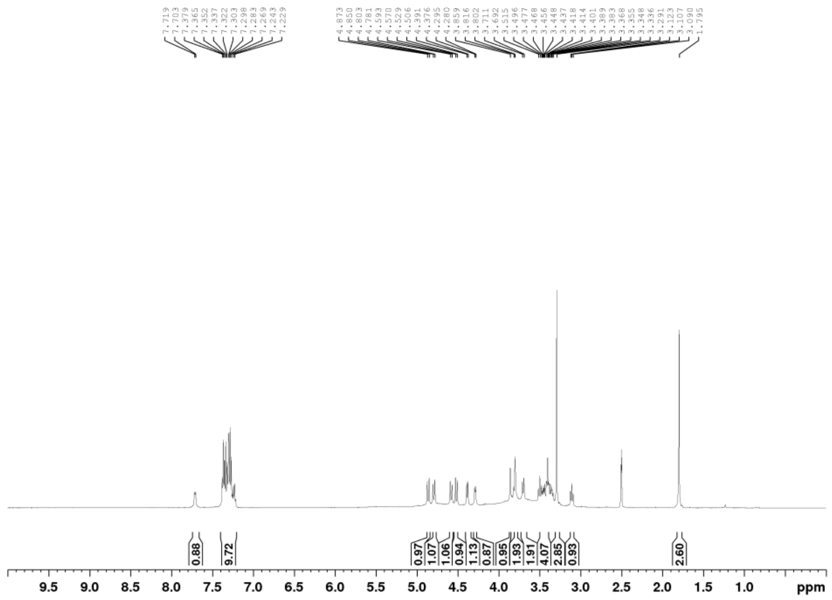

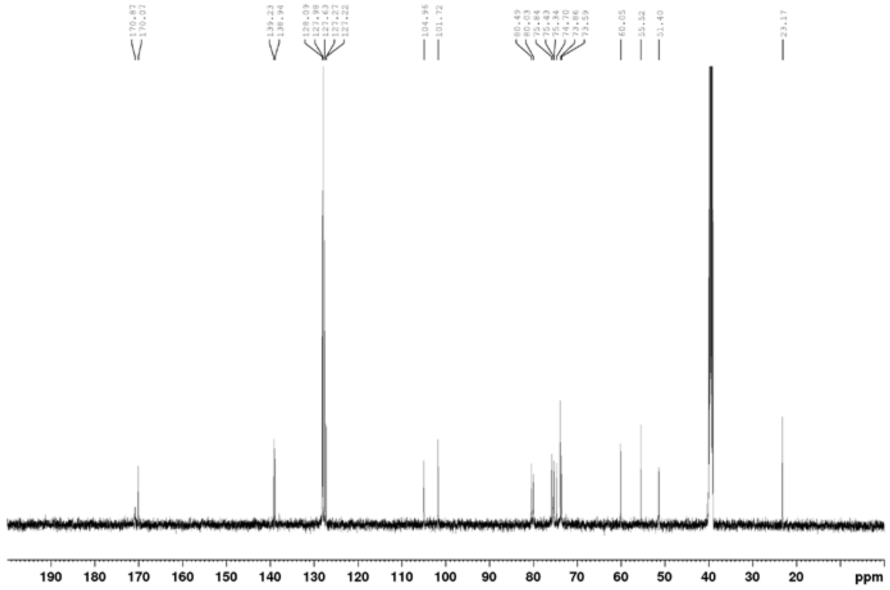

Compound 44g

^{1}H NMR (500 MHz, DMSO) δ 7.77 (d, *J* = 8.6 Hz, 1H), 7.46 (d, *J* = 7.4 Hz, 2H), 7.34 – 7.25 (m, 4H), 7.24 – 7.14 (m, 4H), 5.16 (d, *J* = 11.5 Hz, 1H), 5.05 (d, *J* = 2.2 Hz, 1H), 4.82 (d, *J* = 11.1 Hz, 1H), 4.63 – 4.51 (m, 2H), 4.47 (d, *J* = 11.2 Hz, 2H), 4.35 (d, *J* = 8.3 Hz, 1H), 4.30 (dd, *J* = 8.9, 5.9 Hz, 1H), 4.17 (s, 1H), 4.04 – 3.97 (m, 1H), 3.93 (dd, *J* = 10.4, 5.7 Hz, 1H), 3.87 (dd, *J* = 10.4, 6.3 Hz, 1H), 3.78 (d, *J* = 8.9 Hz, 1H), 3.73 – 3.67 (m, 1H), 3.63 (t, *J* = 6.0 Hz, 1H), 3.28 (s, 3H), 1.83 (s, 3H). ^{13}C NMR (500 MHz, DMSO) δ 172.9, 170.2, 139.8, 139.1, 127.9, 127.9, 127.9, 127.8, 127.0, 126.9, 101.8, 100.1, 78.3, 77.2, 76.7, 76.3, 76.1, 74.1, 73.0, 71.6, 65.2, 55.6, 51.5, 23.1. HRMS (ESI): *m/z*: calcd for $C_{29}H_{34}NNa_4O_{21}S_3$ [M + H]: 920.0371; found: 920.0375.

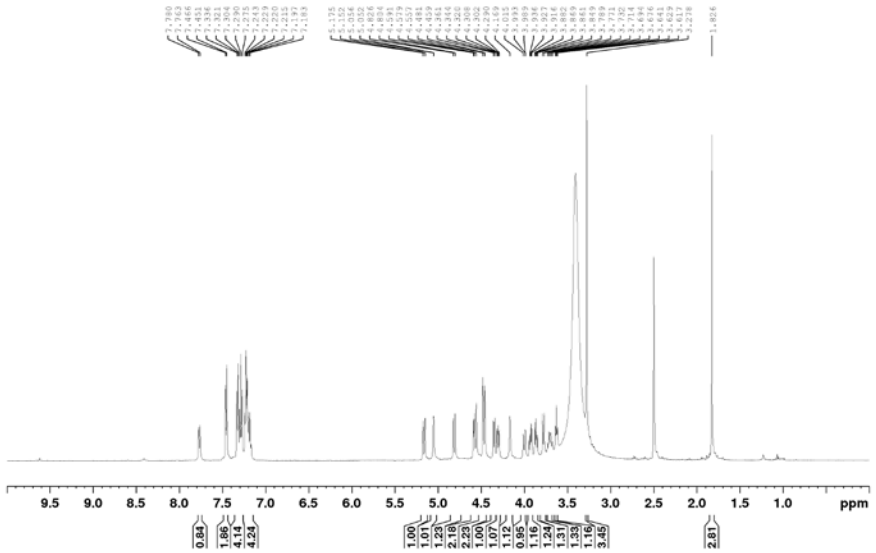

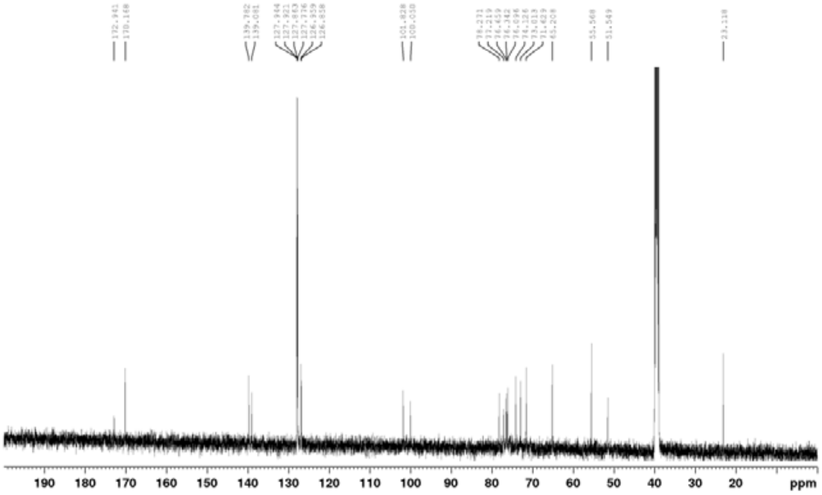

Compound 45g

^{1}H NMR (500 MHz, D_2O) δ 4.84 (d, *J* = 6.4 Hz, 1H), 4.45 (d, *J* = 8.4 Hz, 1H), 4.39 – 4.34 (m, 1H), 4.25 (t, *J* = 6.8 Hz, 1H), 4.21 – 4.14 (m, 3H), 3.93 – 3.76 (m, 5H), 3.44 (s, 3H), 1.98 (s, 3H). ^{13}C NMR (500 MHz, D_2O) δ 175.6, 175.0, 102.4, 100.7, 81.2, 79.6, 77.2, 76.1, 72.6, 70.4, 67.8, 67.1, 57.1, 51.1, 22.5. HRMS (ESI): *m/z*: calcd for $C_{15}H_{21}NNa_5O_{21}S_3$ [M + Na]: 761.9251; found: 761.9246.

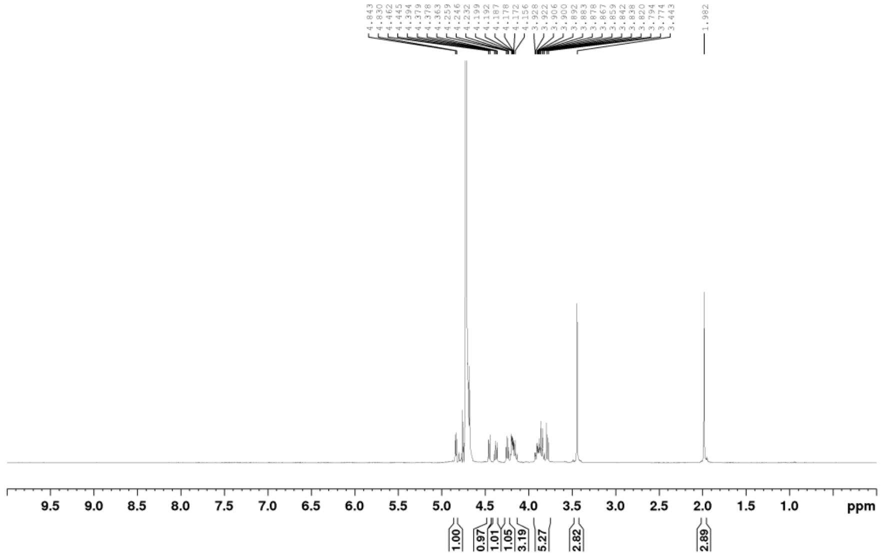

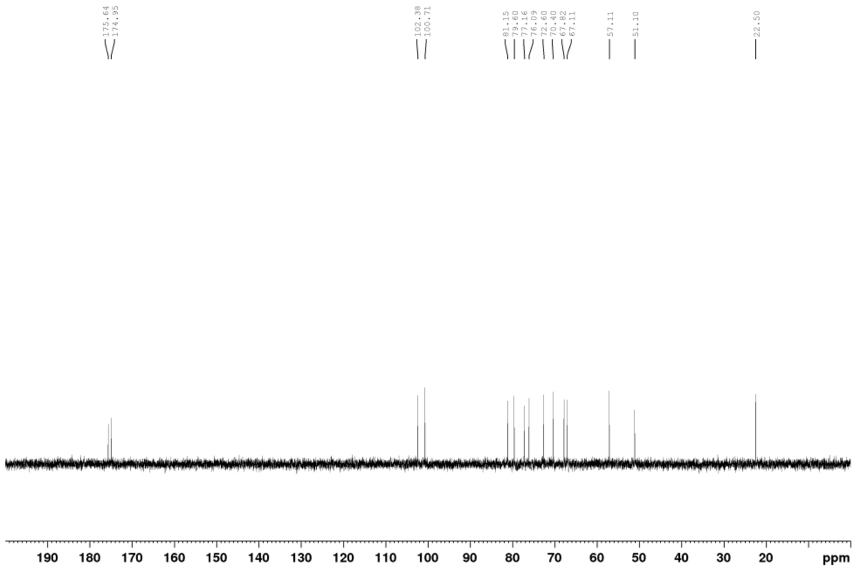

Protected dimer D2-A4

^{1}H NMR (500 MHz, $CDCl_3$) δ 7.94 – 7.82 (m, 4H), 7.48 (td, *J* = 7.2, 1.2 Hz, 2H), 7.34 (t, *J* = 7.6 Hz, 4H), 7.18 – 7.10 (m, 3H), 7.10 – 7.02 (m, 2H), 6.77 (d, *J* = 6.8 Hz, 1H), 5.61 (t, *J* = 9.1 Hz, 1H), 5.50 (d, *J* = 3.4 Hz, 1H), 5.31 (dd, *J* = 9.3, 7.5 Hz, 1H), 4.87 (d, *J* = 7.9 Hz, 2H), 4.73 (dd, *J* = 10.8, 3.5 Hz, 1H), 4.53 (dd, *J* = 33.5, 11.1 Hz, 2H), 4.13 (ddt, *J* = 18.7, 11.6, 8.2 Hz, 4H), 3.90 (t, *J* = 6.3 Hz, 1H), 3.79 (s, 3H), 3.49 – 3.40 (m, 4H), 2.14 (s, 3H), 2.07 (s, 3H). ^{13}C NMR (500 MHz, $CDCl_3$) δ 170.5, 169.8, 168.0, 165.3, 165.0, 162.2, 136.9, 133.3, 133.3, 129.8, 129.6, 129.0, 128.9, 128.4, 128.3, 128.2, 128.0, 127.9, 100.6, 99.3, 91.8, 74.7, 74.4, 74.0, 73.0, 71.8, 71.2, 68.8, 62.3, 57.3, 56.3, 52.7, 20.7, 20.6. HRMS (ESI): *m/z*: calcd for $C_{41}H_{42}Cl_3NNaO_{16}$ [M + Na]: 932.1461; found: 932.1458.

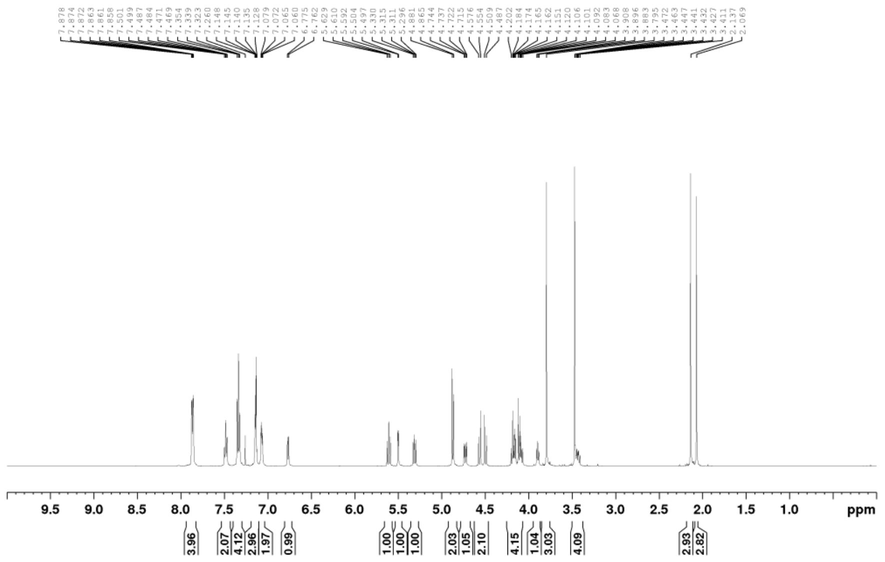

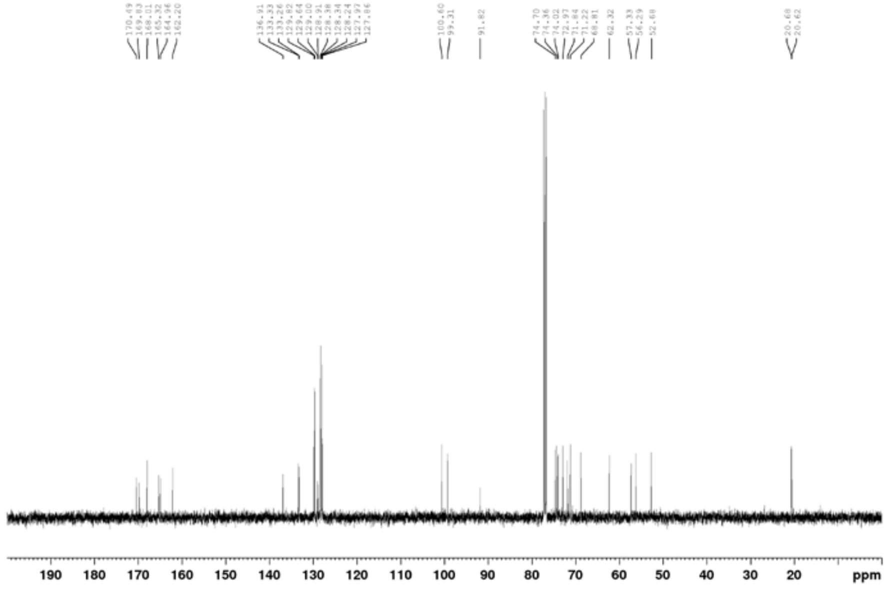

Compound 42h

^{1}H NMR (500 MHz, $CDCl_3$) δ 7.90 (dd, *J* = 19.5, 7.9 Hz, 4H), 7.55 – 7.45 (m, 2H), 7.43 – 7.31 (m, 4H), 7.18 – 7.10 (m, 3H), 7.10 – 7.04 (m, 2H), 5.65 (t, *J* = 9.2 Hz, 1H), 5.41 (dd, *J* = 20.3, 5.1 Hz, 2H), 5.33 (dd, *J* = 9.3, 7.7 Hz, 1H), 4.95 (d, *J* = 8.2 Hz, 1H), 4.81 (d, *J* = 7.5 Hz, 1H), 4.80 – 4.72 (m, 1H), 4.53 (dd, *J* = 33.4, 11.1 Hz, 2H), 4.21 – 4.13 (m, 2H), 4.10 (d, *J* = 9.5 Hz, 1H), 4.04 (dd, *J* = 11.5, 7.4 Hz, 1H), 3.90 – 3.83 (m, 1H), 3.79 (s, 3H), 3.44 (s, 3H), 3.10 (dt, *J* = 10.5, 7.7 Hz, 1H), 2.10 (s, 3H), 2.05 (s, 3H), 1.39 (s, 3H). ^{13}C NMR (500 MHz, $CDCl_3$) δ 171.1, 170.5, 169.9, 168.0, 165.4, 164.9, 137.0, 133.5, 133.3, 129.7, 129.1, 128.6, 128.4, 128.2, 128.0, 127.9, 101.2, 99.6, 75.0, 74.7, 74.3, 74.1, 71.9, 71.2, 68.9, 62.7, 57.0, 55.5, 52.7, 22.9, 20.7, 20.7. HRMS (ESI): *m/z*: calcd for $C_{41}H_{45}NNaO_{16}$ [M + Na]: 830.2631; found: 830.2635.

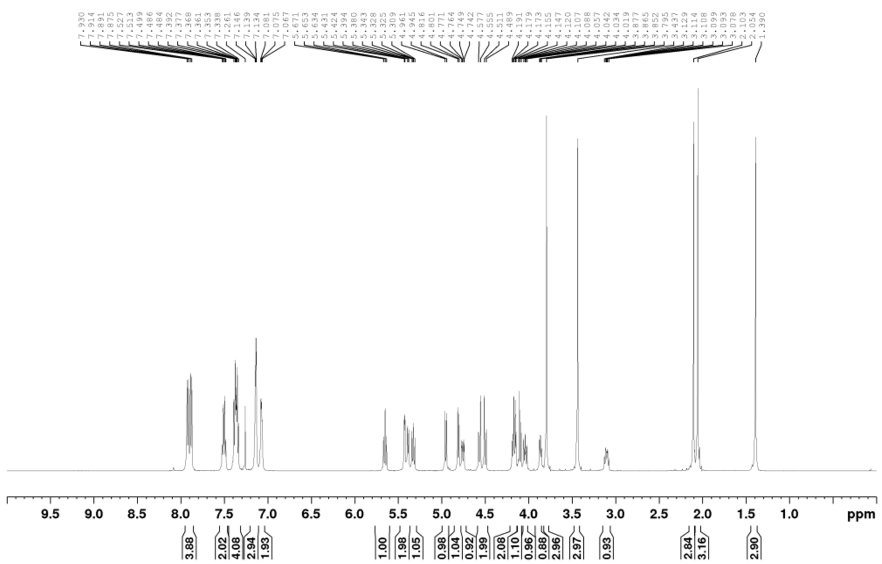

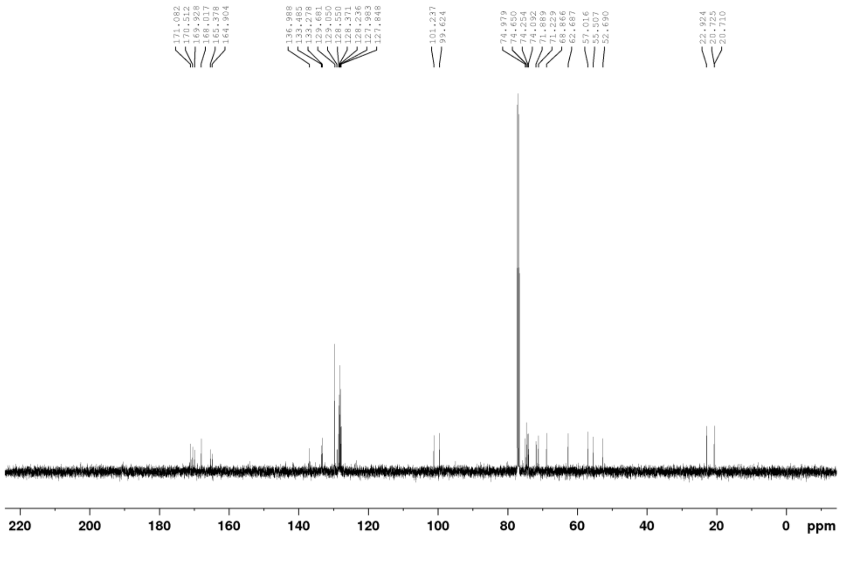

Compound 43h

^{1}H NMR (500 MHz, DMSO) δ 7.69 (d, *J* = 8.9 Hz, 1H), 7.36 – 7.22 (m, 5H), 4.72 (d, *J* = 11.2 Hz, 1H), 4.57 (d, *J* = 11.2 Hz, 1H), 4.34 (d, *J* = 7.6 Hz, 1H), 4.25 (d, *J* = 8.4 Hz, 1H), 3.90 – 3.78 (m, 2H), 3.62 – 3.52 (m, 3H), 3.52 – 3.39 (m, 2H), 3.33 (dd, *J* = 9.9, 4.0 Hz, 2H), 3.31 (d, *J* = 6.1 Hz, 3H), 3.14 (dd, *J* = 16.2, 8.1 Hz, 1H), 1.78 (s, 3H). ^{13}C NMR (500 MHz, DMSO) δ 171.7, 170.0, 139.1, 127.9, 127.6, 127.6, 127.1, 104.5, 101.8, 80.5, 80.5, 76.3, 75.5, 75.0, 73.3, 73.2, 66.7, 60.1, 55.3, 50.6, 23.1. HRMS (ESI): *m/z*: calcd for $C_{22}H_{31}NNaO_{12}$ [M + Na]: 524.1738; found: 524.1745.

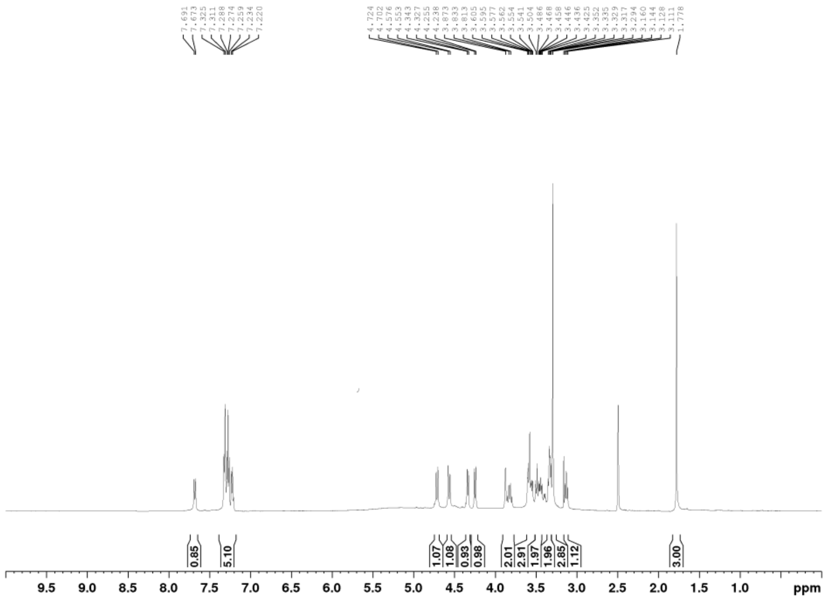

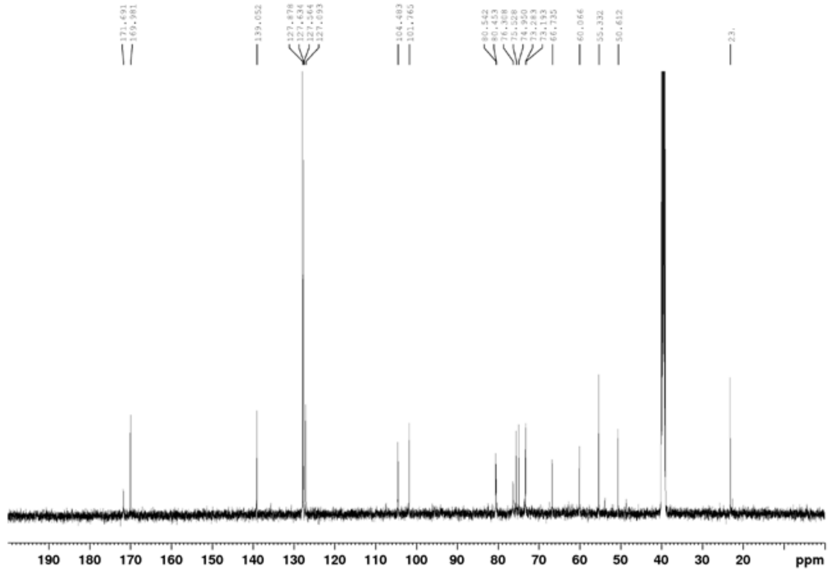

Compound 43h’

^{1}H NMR (500 MHz, MeOD) δ 7.42 – 7.16 (m, 10H), 5.15 (d, *J* = 2.1 Hz, 1H), 4.60 (s, 1H), 4.46 (dd, *J* = 21.7, 9.4 Hz, 2H), 4.36 (d, *J* = 8.3 Hz, 1H), 4.03 (dd, *J* = 10.7, 8.6 Hz, 1H), 3.99 – 3.93 (m, 2H), 3.82 – 3.67 (m, 3H), 3.64 – 3.53 (m, 3H), 3.51 – 3.48 (m, 1H), 3.47 (s, 3H), 3.28 – 3.21 (m, 2H), 1.94 (s, 3H). ^{13}C NMR (500 MHz, MeOD) δ 174.4, 170.4, 139.6, 136.8, 129.6, 129.5, 129.2, 129.0, 128.6, 106.4, 103.6, 82.0, 80.8, 77.4, 76.4, 75.7, 74.7, 69.7, 68.3, 62.5, 59.5, 56.9, 52.8, 23.2. HRMS (ESI): *m/z*: calcd for $C_{29}H_{37}NNaO_{12}$ [M + Na]: 614.2208; found: 614.2214.

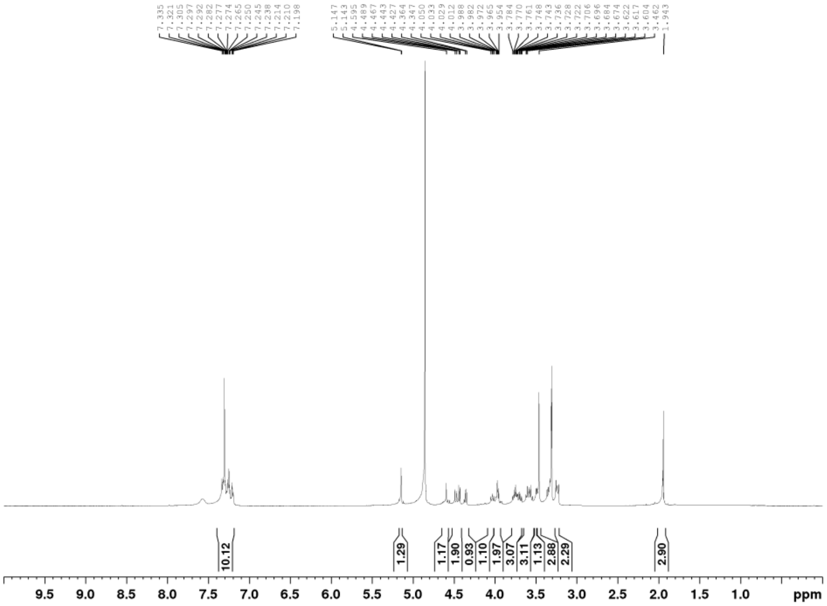

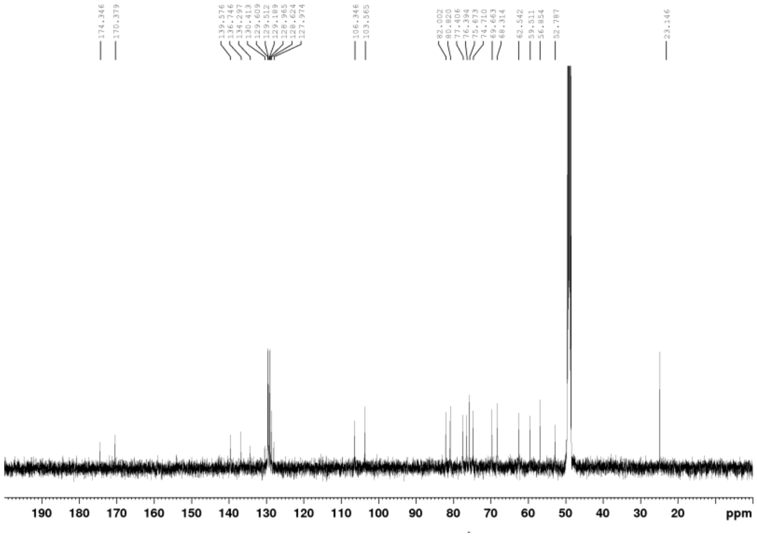

Compound 44h

^{1}H NMR (500 MHz, MeOD) δ 7.45 – 7.27 (m, 7H), 7.23 (dd, *J* = 8.1, 4.2 Hz, 3H), 5.22 (dd, *J* = 35.0, 12.1 Hz, 2H), 5.12 (t, *J* = 4.9 Hz, 2H), 4.95 (s, 1H), 4.70 (d, *J* = 5.6 Hz, 1H), 4.62 – 4.56 (m, 2H), 4.54 – 4.46 (m, 2H), 4.39 (dd, *J* = 11.7, 3.7 Hz, 1H), 4.25 (dd, *J* = 11.6, 7.8 Hz, 1H), 3.97 (d, *J* = 3.3 Hz, 2H), 3.93 (dd, *J* = 7.6, 3.7 Hz, 1H), 3.46 (s, 3H), 2.01 (s, 3H). ^{13}C NMR (500 MHz, MeOD) δ 174.9, 172.2, 139.0, 136.6, 129.7, 129.6, 129.5, 129.2, 128.7, 103.0, 102.3, 79.4, 78.5, 77.9, 76.6, 76.5, 76.5, 74.1, 73.4, 69.0, 68.9, 57.1, 53.1, 23.3. HRMS (ESI): *m/z*: calcd for $C_{29}H_{33}NNa_3O_{24}S_4$ [M – Na]: 975.9974; found: 975.9978.

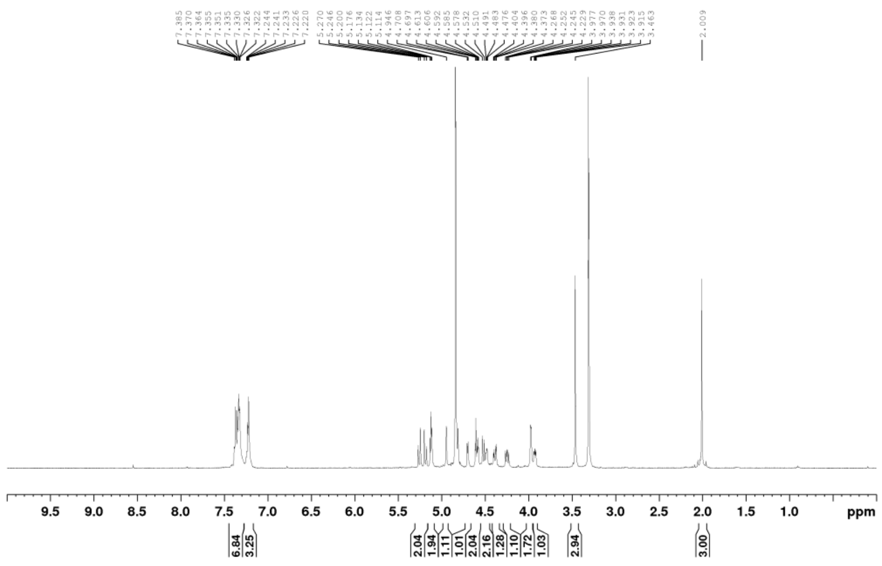

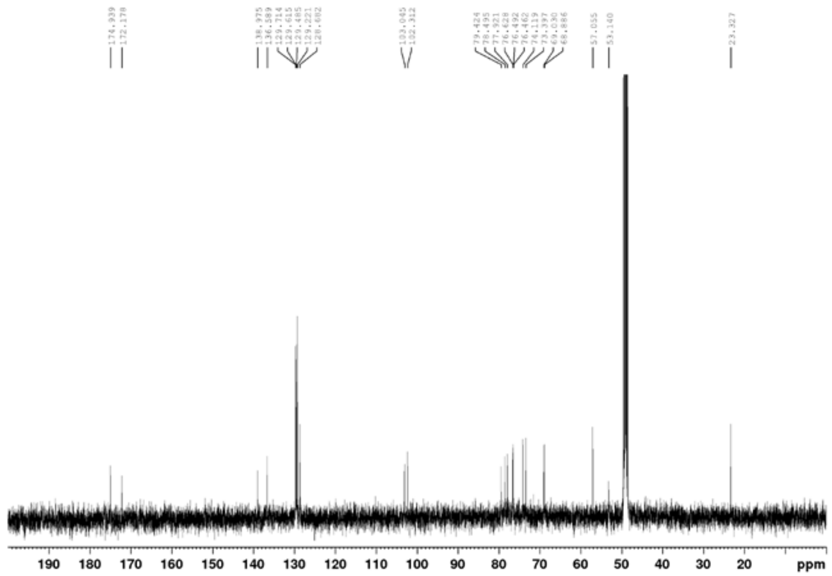

Compound 45h

^{1}H NMR (500 MHz, D_2O) δ 4.88 (d, *J* = 2.6 Hz, 1H), 4.66 (d, *J* = 8.6 Hz, 1H), 4.38 (t, *J* = 7.9 Hz, 1H), 4.30 – 4.24 (m, 2H), 4.20 – 4.11 (m, 2H), 4.02 (dd, *J* = 8.8, 2.5 Hz, 1H), 3.92 (t, *J* = 8.9 Hz, 1H), 3.75 (dt, *J* = 11.0, 6.1 Hz, 2H), 3.47 (s, 3H), 2.01 (s, 3H). ^{13}C NMR (500 MHz, D_2O) δ 175.4, 175.0, 101.9, 101.7, 81.6, 77.3, 76.5, 76.4, 75.9, 72.5, 70.4, 68.1, 57.2, 52.4, 22.5. HRMS (ESI): *m/z*: calcd for $C_{15}H_{20}NNa_4O_{24}S_4$ [M – Na]: 817.8854; found: 817.8857.

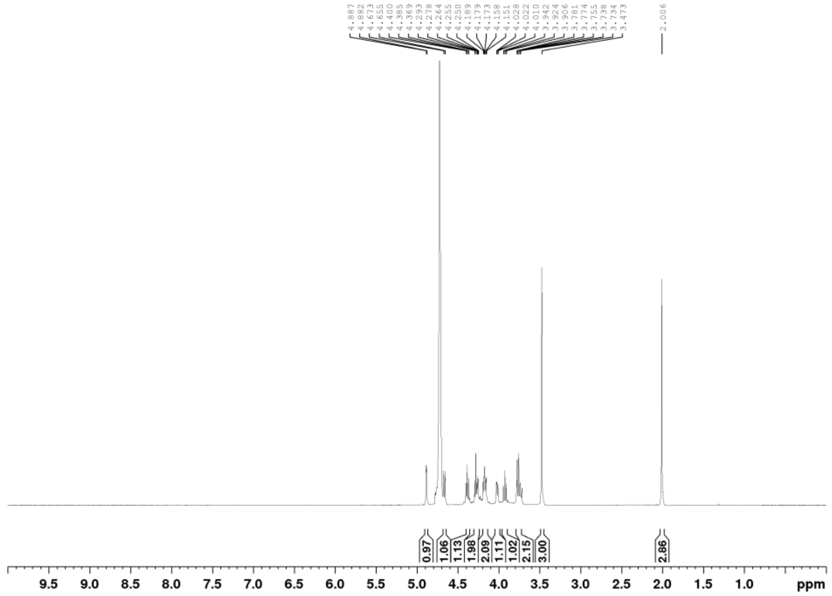

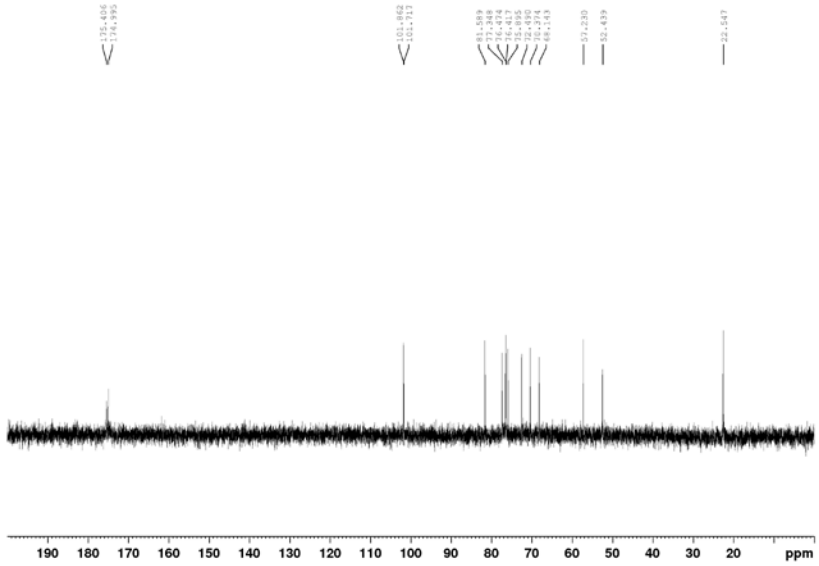

Protected dimer D1-A1

^{1}H NMR (500 MHz, $CDCl_3$) δ 8.03 (d, *J* = 7.3 Hz, 2H), 7.60 (t, *J* = 7.5 Hz, 1H), 7.51 – 7.40 (m, 4H), 7.40 – 7.26 (m, 13H), 7.21 – 7.10 (m, 5H), 6.90 (d, *J* = 6.7 Hz, 1H), 5.48 – 5.37 (m, 1H), 5.02 (d, *J* = 11.4 Hz, 1H), 4.90 – 4.75 (m, 4H), 4.69 (ddd, *J* = 23.2, 12.9, 7.2 Hz, 4H), 4.43 (dd, *J* = 39.1, 11.7 Hz, 2H), 4.14 – 4.07 (m, 2H), 4.09 – 4.02 (m, 1H), 3.85 (t, *J* = 8.8 Hz, 1H), 3.74 – 3.67 (m, 4H), 3.64 – 3.52 (m, 3H), 3.45 (s, 3H). ^{13}C NMR (500 MHz, $CDCl_3$) δ 168.4, 164.9, 162.1, 138.4, 137.8, 137.4, 137.2, 133.2, 129.8, 129.3, 128.7, 128.4, 128.3, 128.2, 128.1, 128.0, 127.9, 127.8, 127.7, 127.6, 127.6, 127.4, 101.2, 99.2, 92.0, 81.4, 79.3, 76.0, 75.2, 75.0, 74.9, 74.7, 74.1, 73.3, 73.2, 68.6, 57.0, 56.3, 52.4. HRMS (ESI): *m/z*: calcd for $C_{51}H_{52}Cl_3NNaO_{13}$ [M + Na]: 1014.2396; found: 1014.2401.

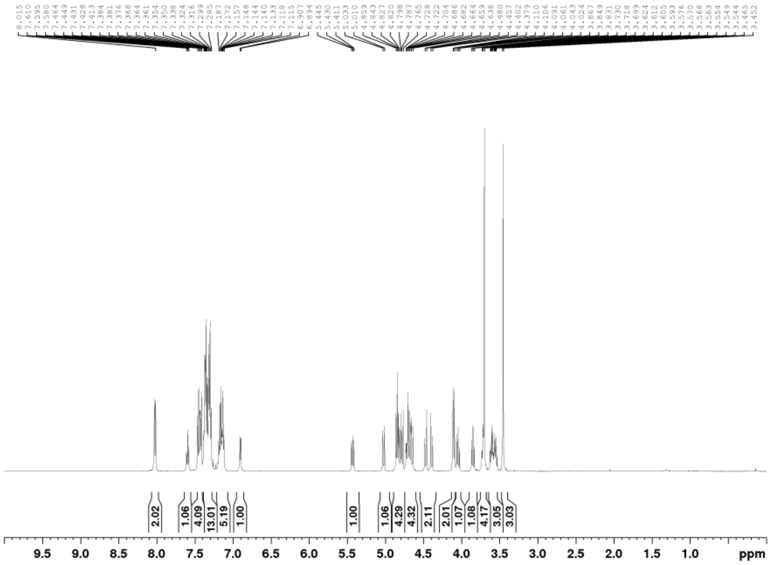

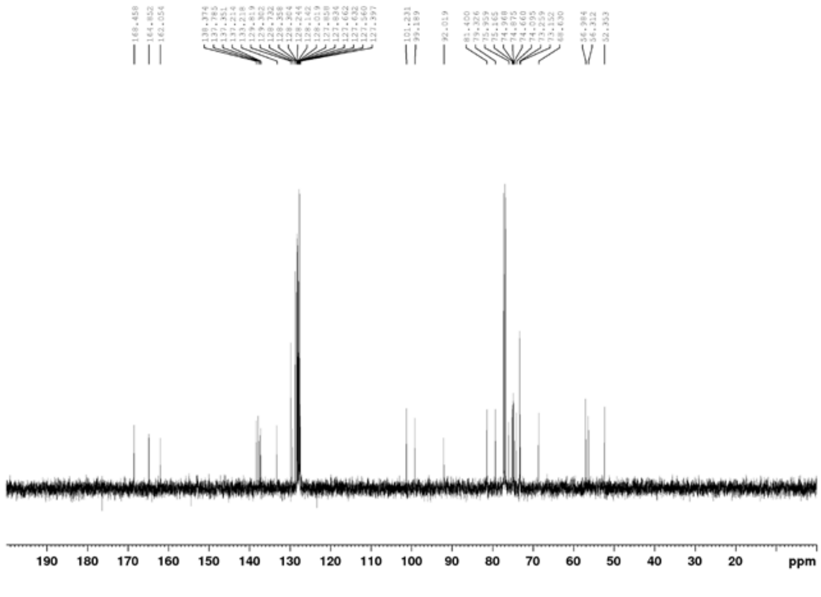

Compound 42a

^{1}H NMR (500 MHz, $CDCl_3$) δ 8.00 (d, *J* = 7.2 Hz, 2H), 7.59 (t, *J* = 7.3 Hz, 1H), 7.53 – 7.19 (m, 17H), 7.21 – 7.08 (m, 5H), 5.36 (dd, *J* = 14.8, 6.6 Hz, 2H), 4.97 (d, *J* = 11.7 Hz, 1H), 4.86 (d, *J* = 8.3 Hz, 1H), 4.83 – 4.74 (m, 3H), 4.74 – 4.58 (m, 4H), 4.38 (dd, *J* = 41.6, 11.7 Hz, 2H), 4.15 – 3.95 (m, 3H), 3.88 (t, *J* = 8.9 Hz, 1H), 3.74 – 3.60 (m, 4H), 3.55 (dd, *J* = 9.5, 5.8 Hz, 1H), 3.46 (dd, *J* = 9.6, 6.6 Hz, 1H), 3.40 (d, *J* = 4.6 Hz, 3H), 3.19 (dt, *J* = 10.8, 7.8 Hz, 1H), 1.48 (s, 3H). ^{13}C NMR (500 MHz, $CDCl_3$) δ 171.1, 168.5, 164.7, 138.8, 138.0, 137.5, 137.5, 133.5, 129.6, 128.8, 128.5, 128.4, 128.3, 128. 2, 128.0, 127.9, 127.84, 127.6, 127.5, 127.3, 102.38, 99.5, 81.6, 79.4, 78.1, 75.3, 75.0, 75.0, 74.4, 74.0, 73.6, 73.2, 73.2, 69.0, 56.7, 55.6, 52.3, 23.3. HRMS (ESI): *m/z*: calcd for $C_{51}H_{55}NNaO_{13}$ [M + Na]: 912.3566; found: 912.3572.

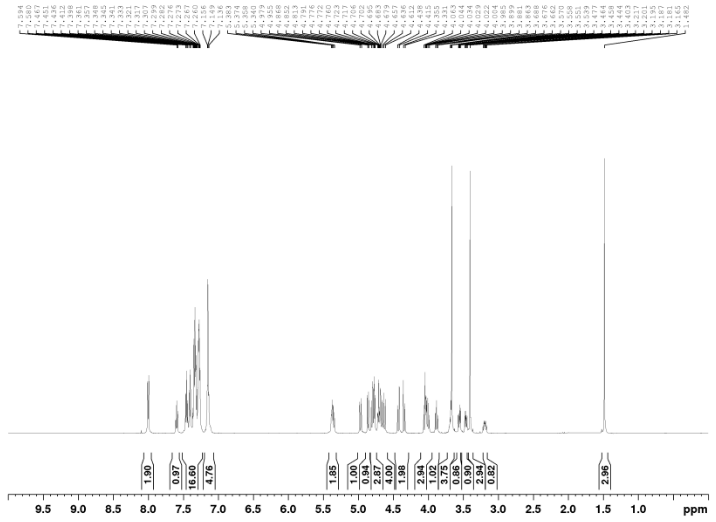

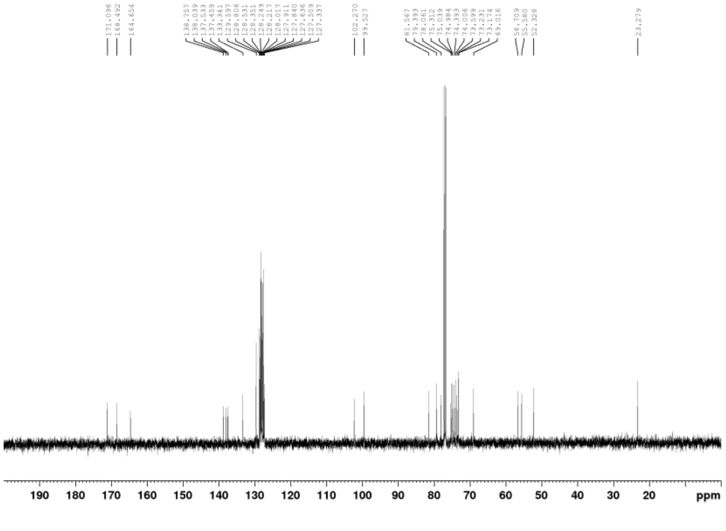

Compound 43a

^{1}H NMR (500 MHz, DMSO) δ 7.74 (d, *J* = 7.3 Hz, 1H), 7.43 – 7.19 (m, 19H), 4.90 (dd, *J* = 21.2, 11.5 Hz, 2H), 4.77 – 4.67 (m, 2H), 4.61 (d, *J* = 10.8 Hz, 1H), 4.52 (dd, *J* = 16.7, 9.6 Hz, 2H), 4.38 (dt, *J* = 17.4, 8.7 Hz, 3H), 3.97 – 3.79 (m, 4H), 3.66 (dt, *J* = 12.2, 7.6 Hz, 2H), 3.55 (t, *J* = 8.9 Hz, 1H), 3.45 (dd, *J* = 9.6, 6.3 Hz, 1H), 3.40 – 3.33 (m, 2H), 3.31 (s, 3H), 1.85 (s, 3H). ^{13}C NMR (500 MHz, DMSO) δ 170.4, 170.1, 139.0, 138.9, 138.3, 138.2, 128.3, 128.1, 128.1, 127.7, 127.6, 127.5, 127.4, 127.3, 127.3, 104.8, 101.7, 83.8, 80.6, 79.1, 75.4, 74.4, 73.9, 73.9, 73.8, 72.6, 72.3, 68.8, 55.5, 51.3, 48.6, 23.2. HRMS (ESI): *m/z*: calcd for $C_{43}H_{49}NNaO_{12}$ [M + Na]: 794.3147; found: 794.3140.

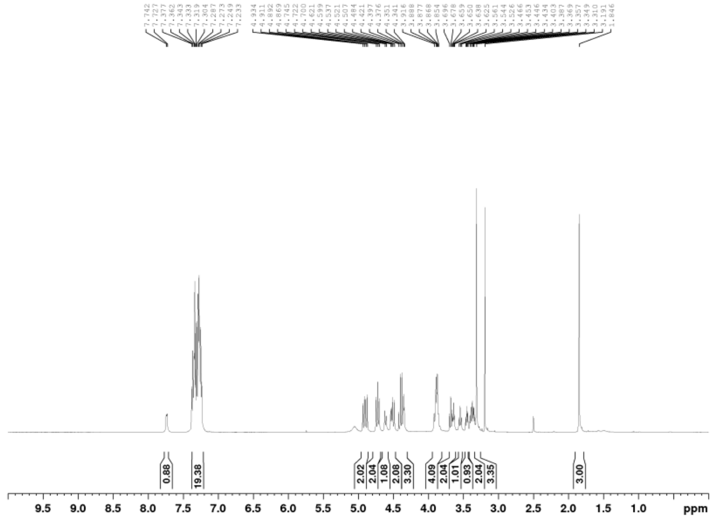

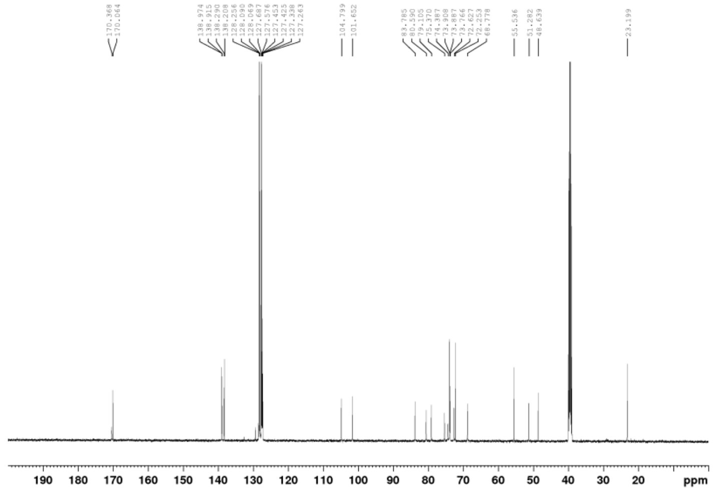

Compound 44a

^{1}H NMR (500 MHz, DMSO) δ 7.94 (d, *J* = 8.0 Hz, 1H), 7.47 – 7.17 (m, 19H), 4.99 (d, *J* = 11.3 Hz, 1H), 4.95 – 4.80 (m, 2H), 4.61 – 4.51 (m, 3H), 4.44 (dt, *J* = 18.0, 11.7 Hz, 4H), 4.37 – 4.29 (m, 1H), 4.11 – 3.94 (m, 3H), 3.89 – 3.74 (m, 3H), 3.62 (s, 1H), 3.43 – 3.58 (m, 2H), 3.27 (s, 3H), 1.87 (s, 3H). ^{13}C NMR (500 MHz, DMSO) δ 172.2, 170.3, 139.2, 138.8, 138.8, 138.3, 128.2, 128.2, 128.0, 127.9, 127.6, 127.5, 127.4, 127.1, 127.0, 101.7, 100.5, 82.4, 78.5, 77.7, 77.4, 75.3, 73.9, 73.1, 73.0, 72.3, 71.8, 69.1, 55.6, 51.8, 23.1. HRMS (ESI): *m/z*: calcd for $C_{43}H_{48}NNa_2O_{15}S$ [M + Na]: 896.2535; found: 896.2539.

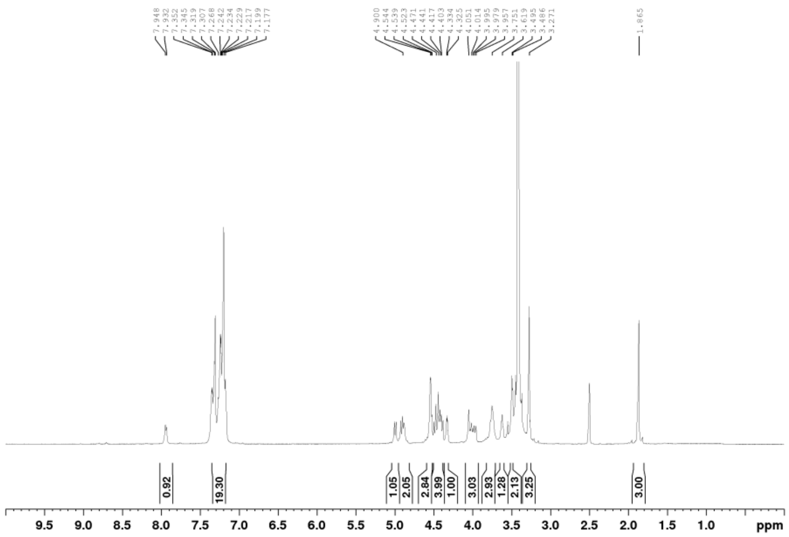

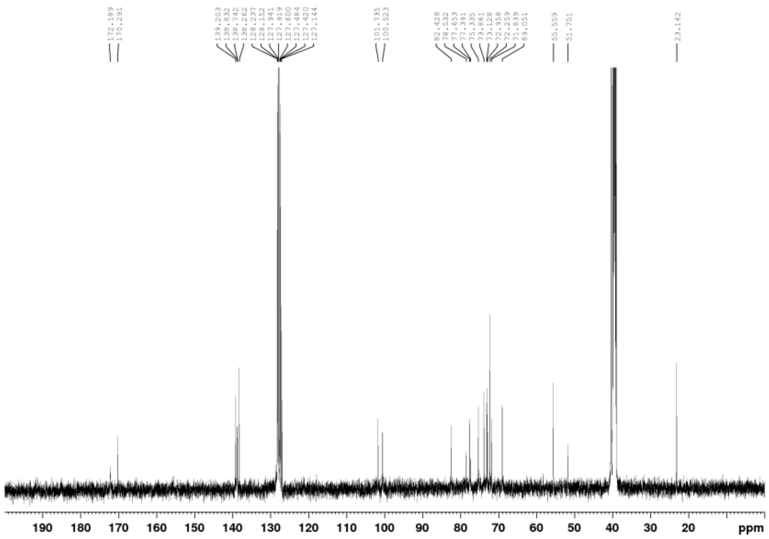

Compound 45a

^{1}H NMR (500 MHz, D_2O) δ 4.44 (d, *J* = 8.3 Hz, 1H), 4.14 (d, *J* = 2.7 Hz, 1H), 4.03 (dd, *J* = 8.8, 7.8 Hz, 1H), 3.87 (ddd, *J* = 19.2, 11.0, 5.7 Hz, 2H), 3.76 – 3.70 (m, 2H), 3.70 – 3.65 (m, 2H), 3.63 (dd, *J* = 7.3, 4.6 Hz, 1H), 3.53 (t, *J* = 9.5 Hz, 1H), 3.44 (s, 3H), 1.98 (s, 3H). ^{13}C NMR (500 MHz, D_2O) δ 175.7, 174.9, 102.4, 101.2, 79.9, 79.8, 76.0, 74.8, 74.6, 71.5, 67.4, 61.1, 57.0, 51.2, 22.5. HRMS (ESI): *m/z*: calcd for $C_{15}H_{23}NNa_3O_{15}S$ [M + Na]: 558.0476; found: 558.0483.

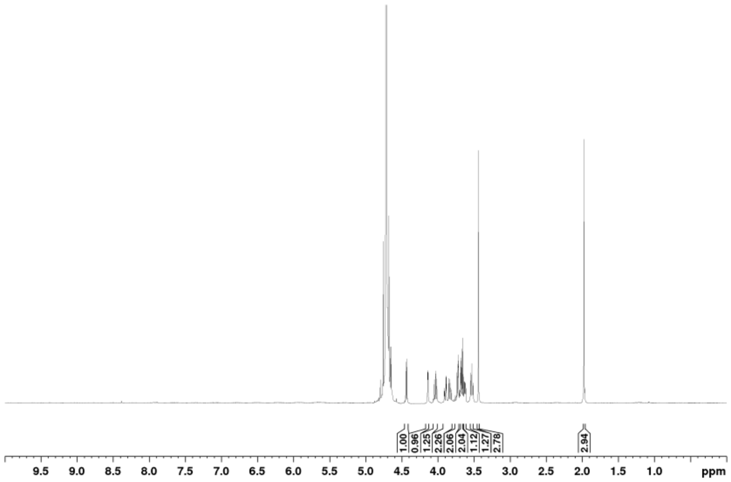

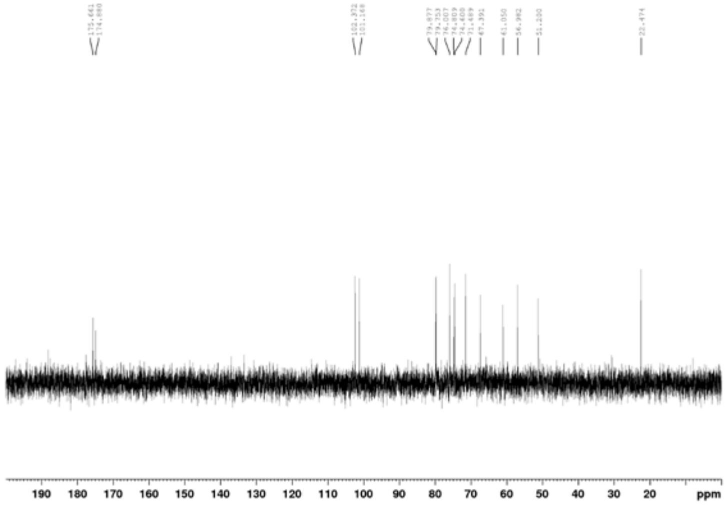

Protected dimer D3-A1

^1H NMR (500 MHz, $CDCl_3$) δ 8.02 – 7.95 (m, 2H), 7.63 – 7.56 (m, 1H), 7.48 – 7.39 (m, 4H), 7.37 – 7.26 (m, 8H), 7.23 – 7.14 (m, 3H), 7.10 – 7.04 (m, 2H), 6.79 (d, *J* = 6.5 Hz, 1H), 5.39 (dd, *J* = 9.2, 7.9 Hz, 1H), 5.33 – 5.23 (m, 1H), 4.99 (d, *J* = 11.5 Hz, 1H), 4.79 (dd, *J* = 14.1, 8.0 Hz, 2H), 4.71 (dd, *J* = 11.0, 2.9 Hz, 1H), 4.67 – 4.60 (m, 2H), 4.55 (d, *J* = 11.6 Hz, 1H), 4.41 (dd, *J* = 34.8, 11.7 Hz, 2H), 4.16 (d, *J* = 2.6 Hz, 1H), 4.04 (d, *J* = 10.0 Hz, 1H), 3.96 – 3.74 (m, 3H), 3.68 (t, *J* = 6.3 Hz, 1H), 3.65 (s, 3H), 3.54 (dddd, *J* = 14.5, 10.7, 8.7, 6.0 Hz, 3H), 3.43 (s, 3H). ^{13}C NMR (500 MHz, $CDCl_3$) δ 167.1, 166.0, 164.6, 162.3, 138.5, 137.9, 137.2, 133.5, 130.0, 129.2, 128.8, 128.5, 128.47, 128.1, 127.9, 127.8, 127.7, 127.5, 101.1, 99.2, 92.1, 79.1, 76.4, 75.5, 74.9, 74.5, 73.5, 73.5, 72.9, 72.4, 71.9, 68.7, 57.2, 56.6, 52.8, 40.3. HRMS (ESI): *m/z*: calcd for $C_{48}H_{46}Cl_4NO_{14}$ [M + Na]: 1000.1667; found: 1000.1660.

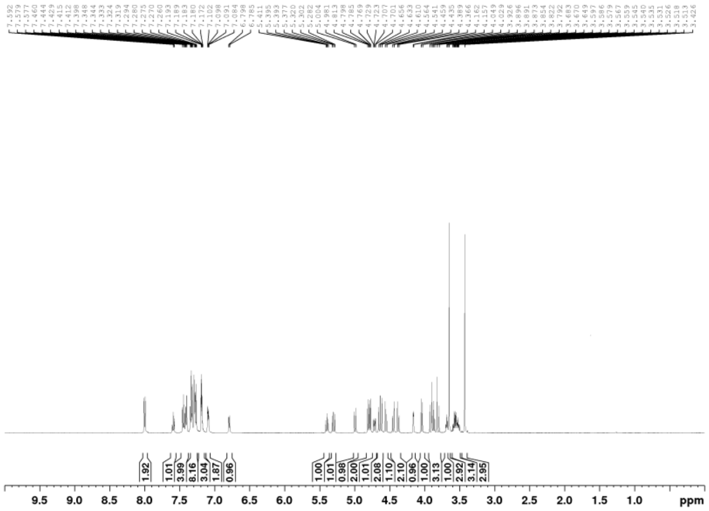

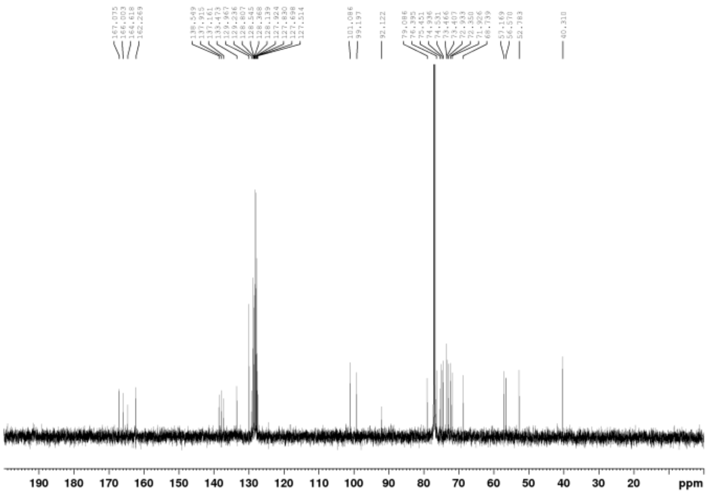

Compound 46a

^{1}H NMR (500 MHz, $CDCl_3$) δ 8.00 (d, *J* = 7.3 Hz, 2H), 7.60 (t, *J* = 7.4 Hz, 1H), 7.50 – 7.36 (m, 4H), 7.33 (dd, *J* = 11.0, 5.2 Hz, 4H), 7.26 (dd, *J* = 7.0, 5.2 Hz, 4H), 7.20 – 7.06 (m, 5H), 5.35 (dd, *J* = 16.2, 7.9 Hz, 2H), 5.27 (t, *J* = 9.6 Hz, 1H), 4.99 (d, *J* = 11.7 Hz, 1H), 4.82 (d, *J* = 8.3 Hz, 1H), 4.77 (d, *J* = 7.8 Hz, 1H), 4.71 (dd, *J* = 11.0, 2.6 Hz, 1H), 4.68 – 4.55 (m, 3H), 4.38 (dd, *J* = 36.7, 11.7 Hz, 2H), 4.10 (d, *J* = 2.1 Hz, 1H), 4.01 (d, *J* = 9.9 Hz, 1H), 3.92 (t, *J* = 9.2 Hz, 1H), 3.72 – 3.58 (m, 4H), 3.55 (dd, *J* = 9.4, 5.7 Hz, 1H), 3.50 – 3.45 (m, 1H), 3.39 (s, 3H), 3.19 (dt, *J* = 10.8, 7.8 Hz, 1H), 2.01 (s, 3H), 1.46 (s, 3H). ^{13}C NMR (500 MHz, $CDCl_3$) δ 171.3, 169.5, 167.4, 164.5, 138.9, 138.1, 137.3, 133.5, 129.7, 129.6, 128.8, 128.6, 128.3, 128.1, 127.8, 127.8, 127.7, 127.6, 127.4, 101.9, 99.6, 79.0, 78.2, 75.6, 74.7, 74.2, 73.3, 73.3, 73.2, 72.3, 71.0, 69.0, 56.8, 55.6, 52.6, 23.3, 20.6. HRMS (ESI): *m/z*: calcd for $C_{46}H_{51}NNaO_{14}$ [M + Na]: 864.3202; found: 864.3206.

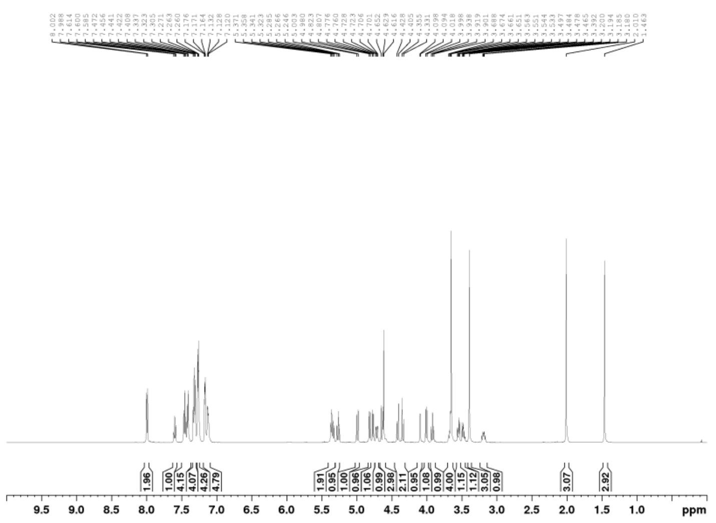

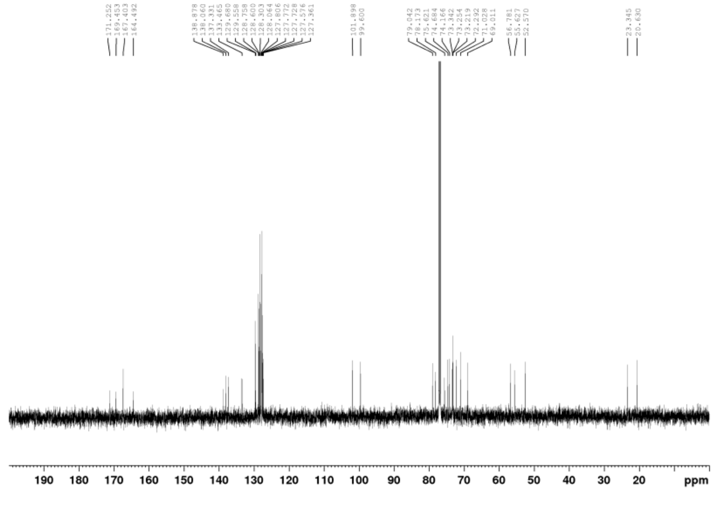

Compound 48a

^{1}H NMR (500 MHz, DMSO) δ 7.95 (d, *J* = 7.6 Hz, 2H), 7.56 (s, 1H), 7.43 (t, *J* = 7.5 Hz, 2H), 5.15 – 5.01 (m, 2H), 4.92 (d, *J* = 8.0 Hz, 1H), 4.59 (t, *J* = 9.2 Hz, 1H), 4.53 (s, 1H), 4.28 (d, *J* = 10.1 Hz, 1H), 4.11 – 4.02 (m, 2H), 3.88 – 3.80 (m, 1H), 3.78 – 3.67 (m, 5H), 3.62 (d, *J* = 8.3 Hz, 1H), 3.23 (s, 3H), 1.94 (s, 3H), 1.16 (s, 3H). ^{13}C NMR (500 MHz, DMSO) δ 169.2, 168.8, 168.7, 164.9, 132.3, 130.6, 130.0, 127.7, 101.8, 101.7, 78.6, 76.1, 74.6, 73.4, 71.8, 71.6, 69.7, 66.8, 55.0, 52.6, 49.9, 22.1, 20.7. HRMS (ESI): *m/z*: calcd for $C_{25}H_{30}NNa_4O_{23}S_3$ [M + Na]: 899.9956; found: 899.9953.

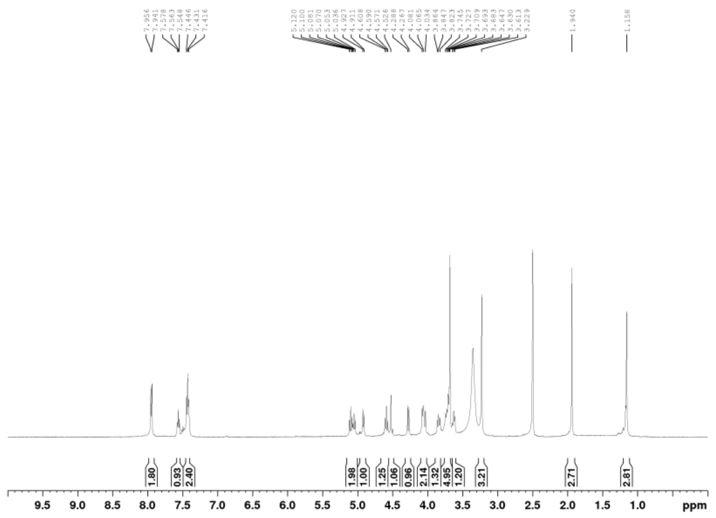

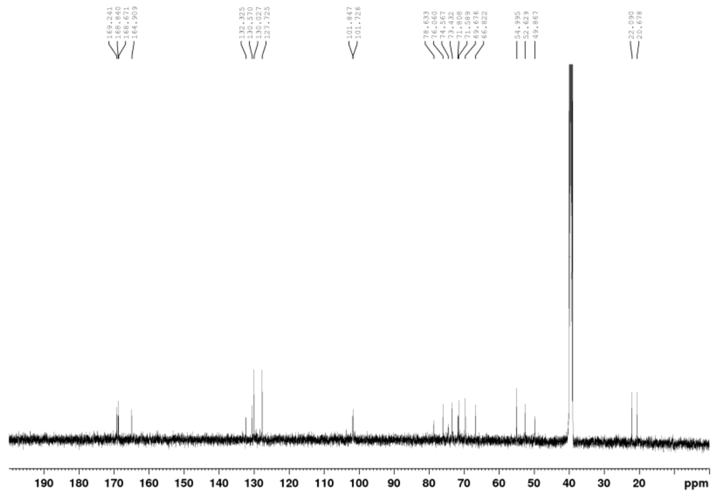

Compound 49a

^{1}H NMR (500 MHz, D_2O) δ 4.50 (d, *J* = 7.9 Hz, 1H), 4.43 (d, *J* = 7.7 Hz, 1H), 4.29 – 4.20 (m, 2H), 4.18 – 4.11 (m, 1H), 4.06 – 3.96 (m, 3H), 3.67 (q, *J* = 10.1 Hz, 2H), 3.53 – 3.42 (m, 4H), 1.96 (s, 3H). ^{13}C NMR (500 MHz, D_2O) δ 175.4, 175.0, 103.1, 102.0, 83.8, 76.4, 76.2, 75.2, 72.4, 71.3, 70.5, 68.0, 57.2, 51.5, 22.3. HRMS (ESI): *m/z*: calcd for $C_{15}H_{21}NNa_5O_{21}S_3$ [M + Na]: 761.9251; found: 761.9258.

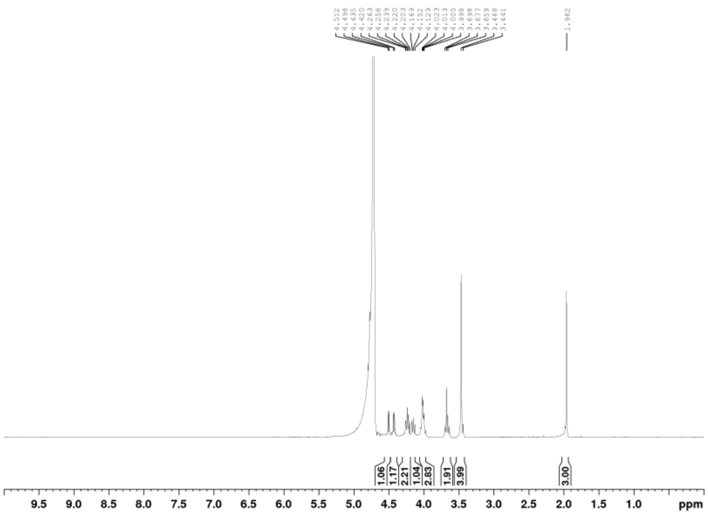

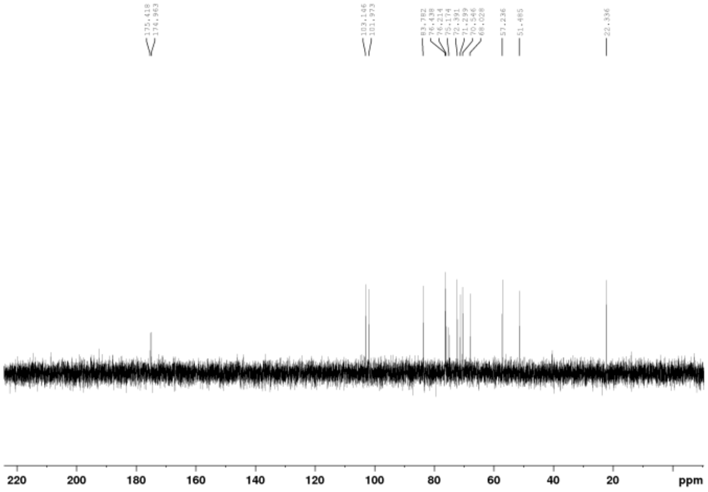

Protected dimer D3-A2

^{1}H NMR (500 MHz, $CDCl_3$) δ 7.97 (dd, *J* = 8.0, 0.9 Hz, 2H), 7.58 (dd, *J* = 10.8, 4.2 Hz, 1H), 7.44 (t, *J* = 7.8 Hz, 2H), 7.36 – 7.25 (m, 5H), 7.21 – 7.14 (m, 3H), 7.08 (dd, *J* = 6.3, 3.0 Hz, 2H), 6.81 (d, *J* = 6.8 Hz, 1H), 5.54 (d, *J* = 3.3 Hz, 1H), 5.38 – 5.24 (m, 2H), 4.80 (dd, *J* = 20.7, 7.9 Hz, 2H), 4.73 – 4.66 (m, 1H), 4.59 – 4.42 (m, 4H), 4.00 (d, *J* = 9.9 Hz, 1H), 3.89 (d, *J* = 14.8 Hz, 1H), 3.85 – 3.77 (m, 3H), 3.71 (s, 3H), 3.55 (dd, *J* = 5.8, 3.1 Hz, 2H), 3.47 (d, *J* = 4.0 Hz, 4H), 2.10 (s, 3H). ^{13}C NMR (500 MHz, $CDCl_3$) δ 169.8, 167.0, 165.7, 164.6, 162.1, 137.8, 137.1, 133.5, 129.8, 129.2, 128.5, 128.3, 128.3, 127.9, 127.8, 127.8, 127.7, 100.2, 99.4, 92.0, 78.9, 74.1, 73.6, 73.2, 72.9, 72.8, 72.2, 72.0, 69.1, 68.8, 57.3, 56.4, 52.8, 40.3, 20.6. HRMS (ESI): *m/z*: calcd for $C_{41}H_{43}Cl_4NNaO_{15}$ [M + Na]: 952.1279; found: 952.1285.

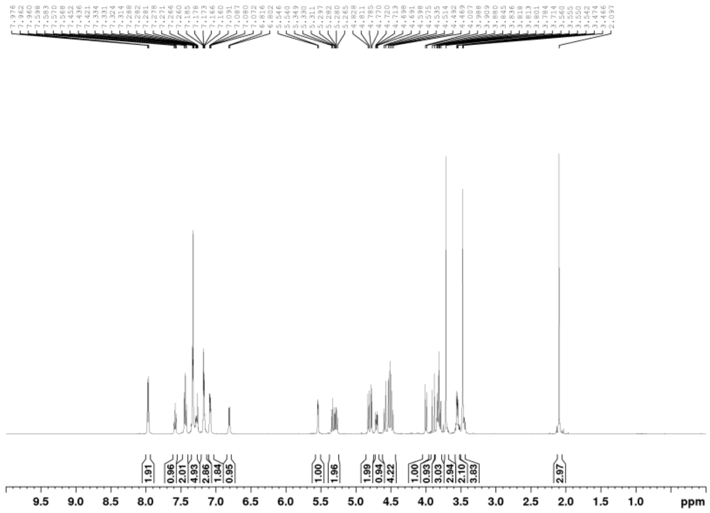

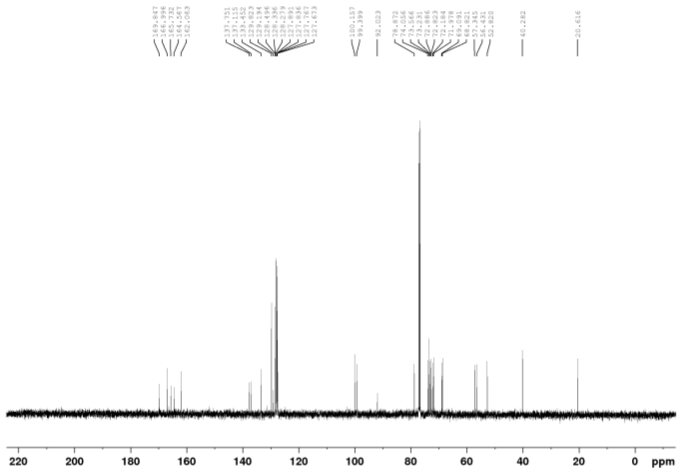

Compound 46b

^{1}H NMR (500 MHz, $CDCl_3$) δ 7.98 (d, *J* = 7.9 Hz, 2H), 7.59 (t, *J* = 7.5 Hz, 1H), 7.45 (t, *J* = 7.7 Hz, 2H), 7.32 (dd, *J* = 7.2, 6.3 Hz, 4H), 7.26 (dd, *J* = 8.4, 4.9 Hz, 1H), 7.18 – 7.03 (m, 5H), 5.52 (d, *J* = 6.7 Hz, 1H), 5.41 (d, *J* = 3.2 Hz, 1H), 5.28 (dd, *J* = 18.3, 9.0 Hz, 2H), 4.93 (d, *J* = 8.2 Hz, 1H), 4.78 – 4.65 (m, 2H), 4.57 (d, *J* = 12.2 Hz, 2H), 4.54 – 4.44 (m, 2H), 3.95 (d, *J* = 9.7 Hz, 1H), 3.88 – 3.79 (m, 2H), 3.73 (s, 3H), 3.57 (dd, *J* = 10.2, 4.8 Hz, 1H), 3.50 (dd, *J* = 10.3, 6.9 Hz, 1H), 3.44 (s, 3H), 3.15 (dt, *J* = 10.5, 7.7 Hz, 1H), 2.07 (s, 3H), 1.98 (s, 3H), 1.38 (s, 3H). ^{13}C NMR (500 MHz, $CDCl_3$) δ 171.0, 170.1, 169.2, 167.2, 164.6, 137.9, 137.2, 133.5, 129.7, 129.3, 128.5, 128.3, 128.2, 127.8, 127.7, 127.7, 127.6, 100.6, 99.7, 79.0, 75.4, 73.8, 73.5, 72.9, 72.8, 72.7, 70.7, 69.3, 68.8, 57.1, 55.4, 52.6, 22.9, 20.7, 20.6. HRMS (ESI): *m/z*: calcd for $C_{41}H_{47}NNaO_{15}$ [M + Na]: 816.2838; found: 816.2833.

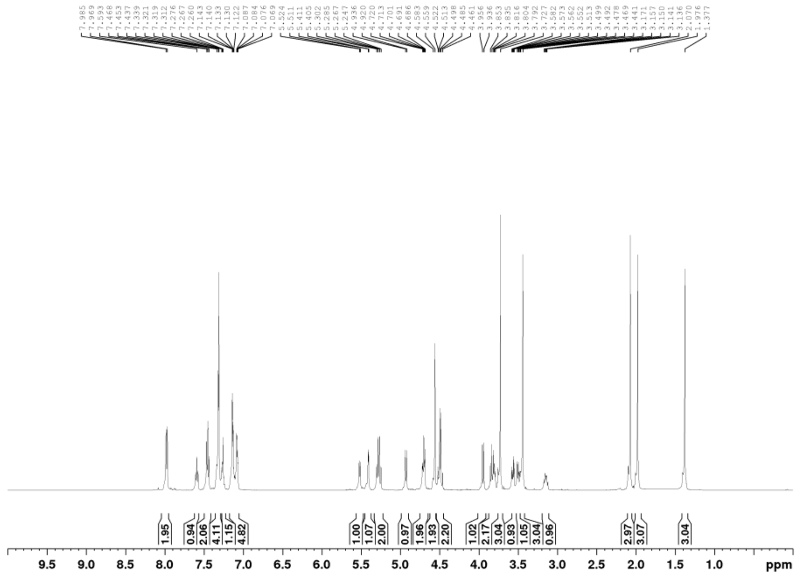

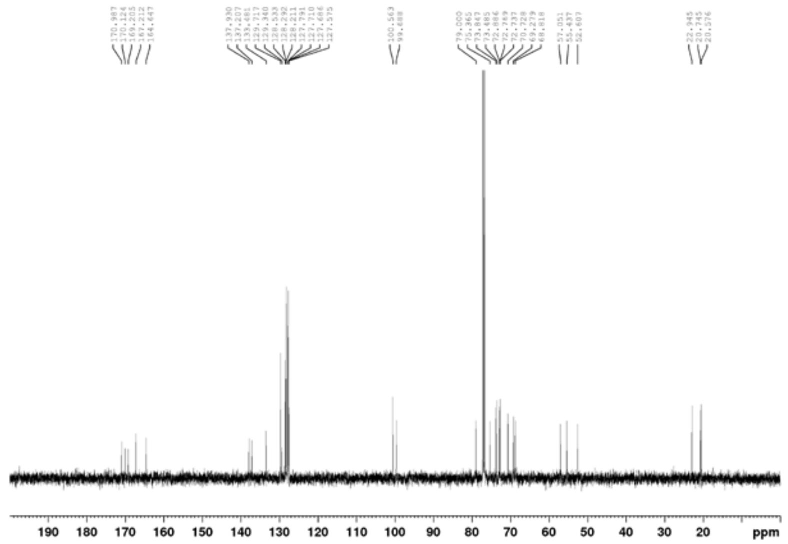

Compound 48b

^{1}H NMR (500 MHz, MeOD) δ 8.09 (d, *J* = 7.2 Hz, 2H), 7.54 (dd, *J* = 10.5, 4.1 Hz, 1H), 7.41 (t, *J* = 7.8 Hz, 2H), 5.30 (dd, *J* = 18.1, 9.0 Hz, 2H), 5.00 – 4.89 (m, 2H), 4.38 (dd, *J* = 11.8, 4.4 Hz, 1H), 4.35 – 4.20 (m, 3H), 3.96 (dd, *J* = 10.5, 8.2 Hz, 1H), 3.84 (d, *J* = 10.7 Hz, 1H), 3.78 (dd, *J* = 8.0, 5.1 Hz, 1H), 3.73 (s, 3H), 3.27 (dd, *J* = 3.1, 1.7 Hz, 3H), 2.03 (d, *J* = 4.7 Hz, 3H), 2.01 (s, 3H), 1.32 (s, 3H). ^{13}C NMR (500 MHz, MeOD) δ 173.7, 172.6, 171.8, 170.0, 167.8, 134.3, 131.8, 131.2, 129.2, 103.3, 103.1, 79.8, 78.9, 76.6, 73.7, 73.5, 73.3, 71.0, 65.3, 56.7, 53.4, 52.2, 22.4, 21.0, 20.8. HRMS (ESI): *m/z*: calcd for $C_{27}H_{33}NNa_3O_{21}S_2$ [M + Na]: 840.0674; found: 840.0681.

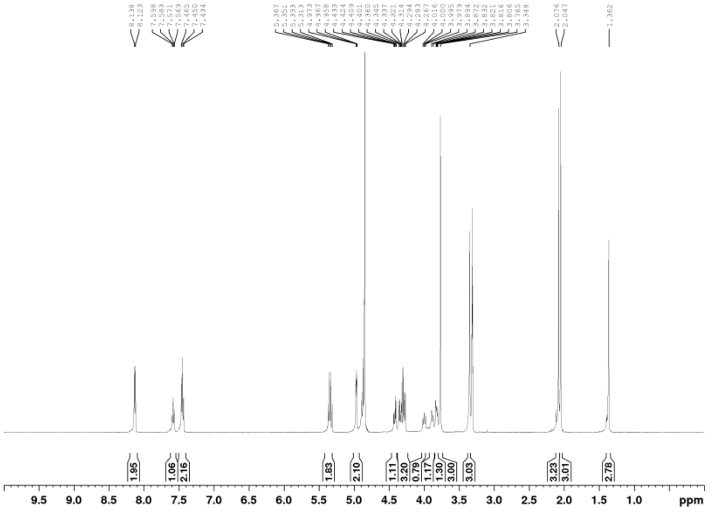

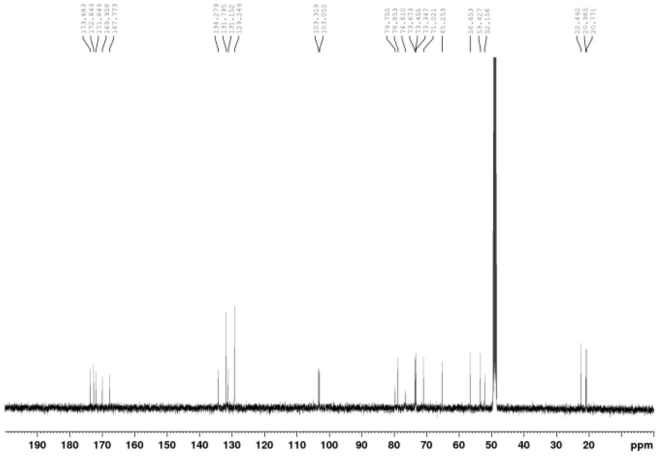

Compound 49b

^{1}H NMR (500 MHz, D_2O) δ 4.51 (d, *J* = 7.9 Hz, 1H), 4.42 (d, *J* = 7.8 Hz, 1H), 4.22 (t, *J* = 9.0 Hz, 1H), 4.03 – 3.95 (m, 2H), 3.80 – 3.72 (m, 4H), 3.68 (t, *J* = 9.4 Hz, 1H), 3.50 – 3.41 (m, 4H), 1.96 (s, 3H). ^{13}C NMR (500 MHz, D_2O) δ 175.0, 174.6, 103.3, 102.0, 83.6, 76.4, 75.9, 75.7, 74.5, 71.3, 70.4, 61.0, 57.2, 51.6, 22.3. HRMS (ESI): *m/z*: calcd for $C_{15}H_{22}NNa_4O_{18}S_2$ [M + Na]: 659.9864; found: 659.9861.

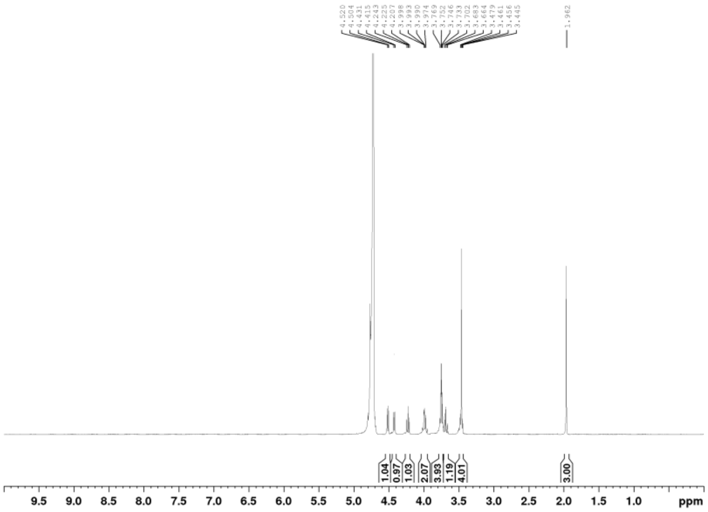

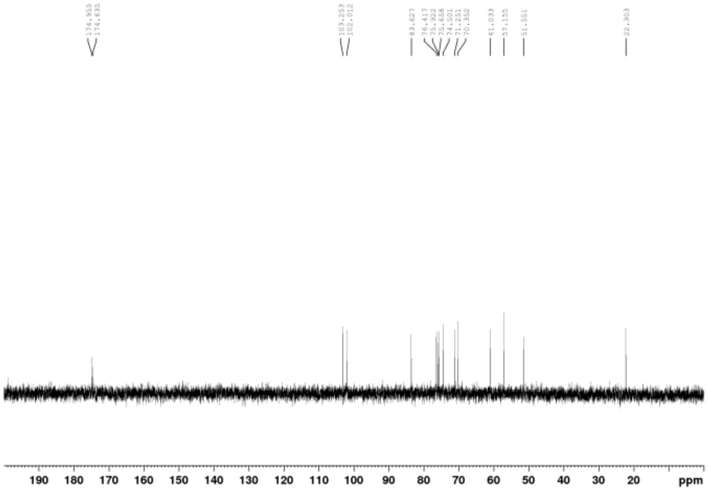

Protected dimer D3-A3

^{1}H NMR (500 MHz, $CDCl_3$) δ 8.00 (d, *J* = 7.6 Hz, 2H), 7.59 (t, *J* = 7.4 Hz, 1H), 7.44 (t, *J* = 7.1 Hz, 4H), 7.35 (t, *J* = 7.4 Hz, 2H), 7.28 (dd, *J* = 13.5, 6.4 Hz, 1H), 7.21 – 7.15 (m, 3H), 7.09 (dd, *J* = 6.2, 2.4 Hz, 2H), 6.87 (d, *J* = 6.5 Hz, 1H), 5.41 (dd, *J* = 8.9, 8.2 Hz, 1H), 5.30 (t, *J* = 9.5 Hz, 1H), 5.01 (d, *J* = 11.5 Hz, 1H), 4.82 – 4.68 (m, 4H), 4.59 (dd, *J* = 32.3, 11.7 Hz, 2H), 4.20 (dd, *J* = 11.1, 6.6 Hz, 1H), 4.08 (d, *J* = 10.1 Hz, 2H), 4.02 (dd, *J* = 11.1, 6.4 Hz, 1H), 3.95 – 3.79 (m, 3H), 3.75 (s, 3H), 3.69 (dd, *J* = 11.5, 5.0 Hz, 1H), 3.58 – 3.48 (m, 1H), 3.42 (d, *J* = 5.5 Hz, 3H), 1.99 (s, 3H). ^{13}C NMR (500 MHz, $CDCl_3$) δ 170.5, 167.0, 166.0, 164.6, 162.3, 138.1, 137.1, 133.5, 129.9, 129.1, 128.9, 128.5, 128.3, 128.2, 127.9, 127.9, 127.6, 101.1, 99.2, 92.0, 79.1, 76.2, 74.9, 74.8, 74.6, 72.9, 72.4, 71.9, 62.6, 57.1, 56.3, 52.8, 40.3, 20.6. HRMS (ESI): *m/z*: calcd for $C_{41}H_{43}Cl_4NNaO_{15}$ [M + Na]: 952.1279; found: 952.1276.

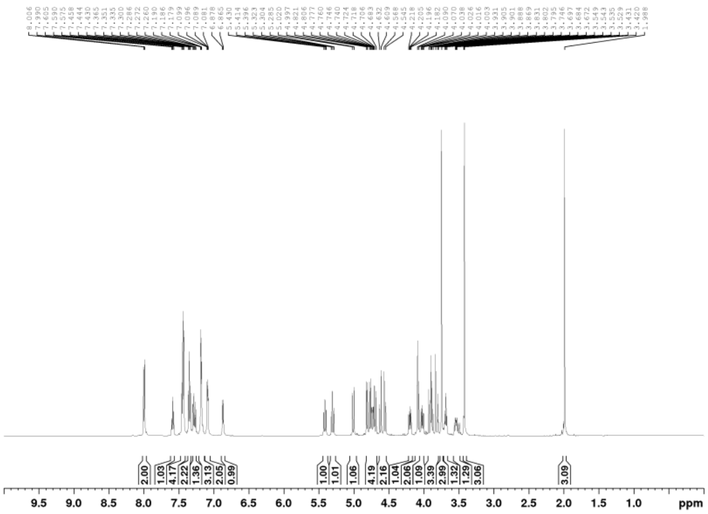

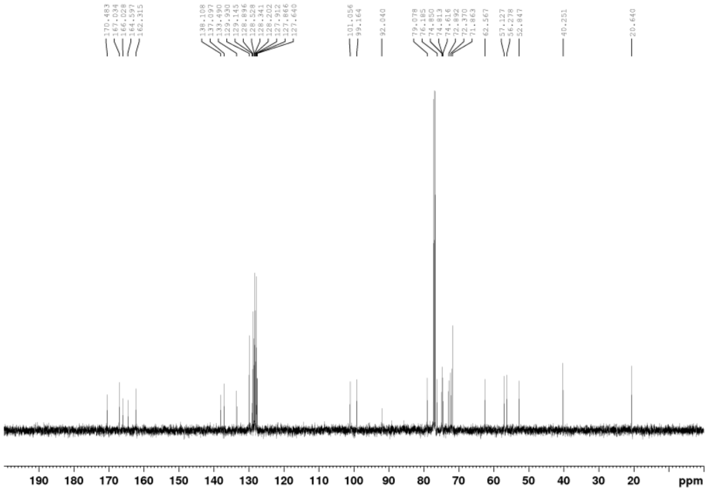

Compound 46c

^{1}H NMR (500 MHz, $CDCl_3$) δ 8.02 – 7.92 (m, 2H), 7.60 (t, *J* = 7.5 Hz, 1H), 7.48 – 7.38 (m, 4H), 7.34 (t, *J* = 7.4 Hz, 2H), 7.30 – 7.24 (m, 1H), 7.19 – 7.09 (m, 5H), 5.42 – 5.31 (m, 2H), 5.27 (t, *J* = 9.5 Hz, 1H), 4.99 (d, *J* = 11.6 Hz, 1H), 4.78 (dd, *J* = 13.2, 8.1 Hz, 2H), 4.74 – 4.65 (m, 2H), 4.62 (s, 2H), 4.15 (dd, *J* = 11.2, 6.7 Hz, 1H), 4.01 (t, *J* = 13.3 Hz, 2H), 3.95 (dt, *J* = 18.5, 7.7 Hz, 2H), 3.73 (d, *J* = 8.0 Hz, 3H), 3.65 (t, *J* = 6.3 Hz, 1H), 3.39 (d, *J* = 5.6 Hz, 3H), 3.19 (dt, *J* = 10.9, 7.9 Hz, 1H), 2.01 (s, 3H), 1.95 (s, 3H), 1.48 (s, 3H). ^{13}C NMR (500 MHz, $CDCl_3$) δ 171.3, 170.5, 169.5, 167.4, 164.5, 138.4, 137.3, 133.5, 129.7, 129.5, 128.9, 128.6, 128.3, 128.2, 127.8, 127.5, 101.9, 99.6, 79.0, 77.9, 75.0, 74.6, 74.2, 73.2, 72.3, 71.7, 71.1, 62.9, 56.7, 55.4, 52.7, 23.4, 20.7, 20.6. HRMS (ESI): *m/z*: calcd for $C_{41}H_{47}NNaO_{15}$ [M + Na]: 816.2838; found: 816.2846.

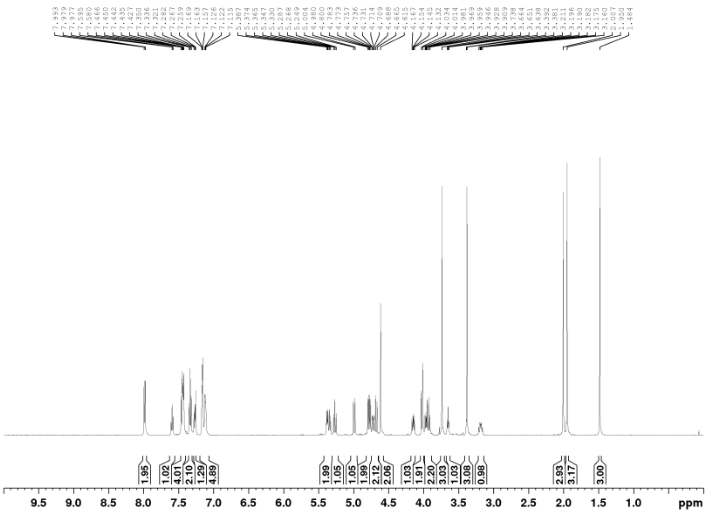

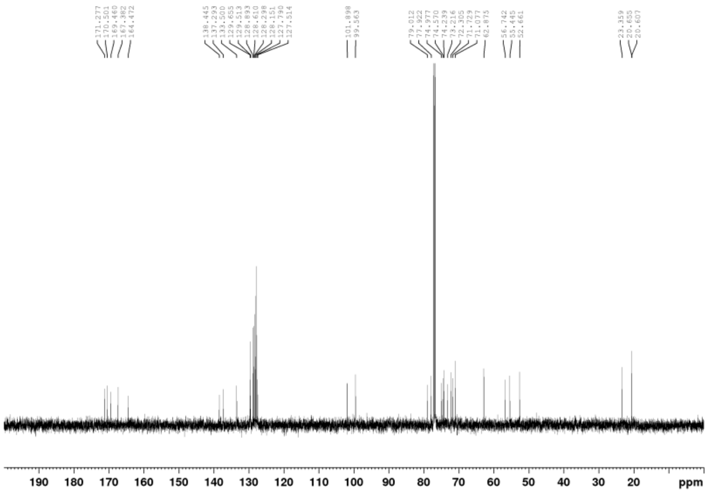

Compound 48c

^{1}H NMR (500 MHz, MeOD) δ 8.09 (d, *J* = 8.1 Hz, 2H), 7.55 (t, *J* = 7.4 Hz, 1H), 7.41 (t, *J* = 7.6 Hz, 2H), 5.29 (t, *J* = 8.9 Hz, 2H), 4.99 – 4.92 (m, 2H), 4.88 – 4.81 (m, 1H), 4.38 (dd, *J* = 11.8, 4.1 Hz, 1H), 4.28 (ddd, *J* = 17.6, 12.6, 7.9 Hz, 3H), 3.95 (d, *J* = 8.2 Hz, 1H), 3.84 (d, *J* = 10.9 Hz, 1H), 3.78 (dd, *J* = 7.2, 4.5 Hz, 1H), 3.73 (s, 3H), 3.27 (s, 3H), 2.04 (s, 3H), 2.01 (s, 3H), 1.32 (s, 3H). ^{13}C NMR (500 MHz, MeOD) δ 173.7, 172.7, 171.9, 170.0, 167.8, 134.3, 131.8, 131.1, 129.2, 103.4, 103.1, 79.8, 78.9, 76.7, 73.7, 73.4, 73.3, 71.1, 65.2, 56.7, 53.5, 52.2, 22.4, 21.0, 20.8. HRMS (ESI): *m/z*: calcd for $C_{27}H_{33}NNa_3O_{21}S_2$ [M + Na]: 840.0674; found: 840.0682.

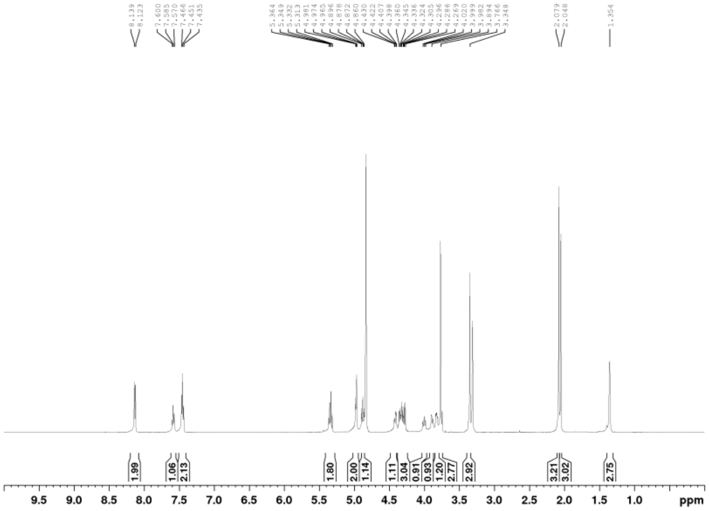

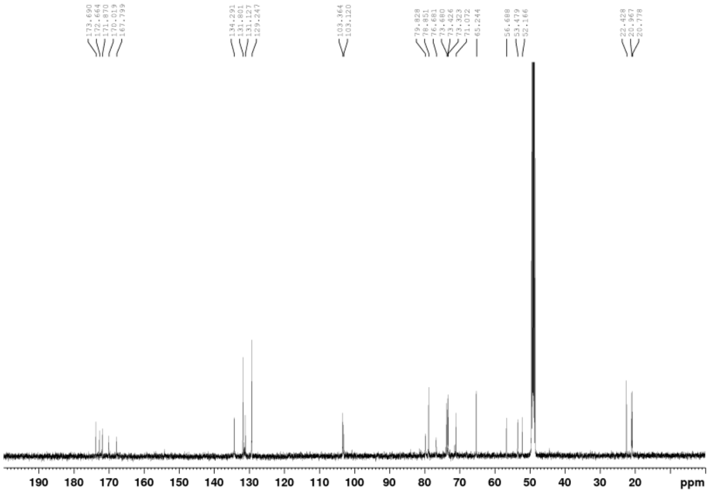

Compound 49c

^{1}H NMR (500 MHz, D_2O) δ 4.52 (d, *J* = 7.8 Hz, 1H), 4.44 (d, *J* = 7.9 Hz, 1H), 4.24 (t, *J* = 8.6 Hz, 1H), 4.06 – 3.95 (m, 2H), 3.81 – 3.74 (m, 3H), 3.69 (q, *J* = 10.1 Hz, 2H), 3.50 – 3.47 (m, 4H), 1.98 (s, 3H). ^{13}C NMR (500 MHz, D_2O) δ 175.2, 175.0, 103.2, 102.1, 83.7, 76.5, 76.3, 75.5, 74.5, 71.3, 70.5, 61.1, 57.2, 51.6, 22.3. HRMS (ESI): *m/z*: calcd for $C_{15}H_{22}NNa_4O_{18}S_2$ [M + Na]: 659.9864; found: 659.9872.

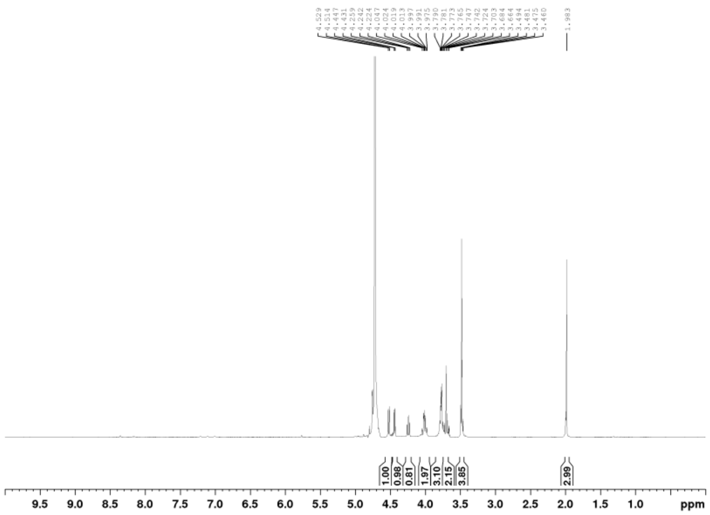

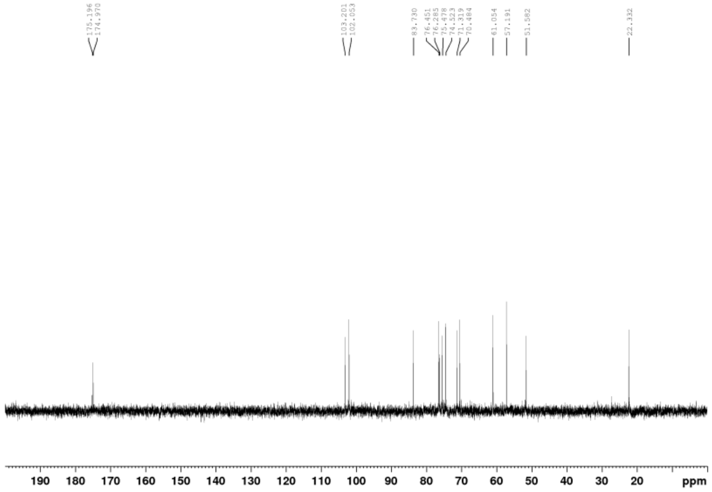

Protected dimer D3-A4

^{1}H NMR (500 MHz, $CDCl_3$) δ 7.99 – 7.88 (m, 2H), 7.59 (t, *J* = 7.5 Hz, 1H), 7.44 (t, *J* = 7.8 Hz, 2H), 7.17 (dd, *J* = 5.0, 1.7 Hz, 3H), 7.08 (dd, *J* = 6.4, 3.0 Hz, 2H), 6.80 (s, 1H), 5.50 (d, *J* = 3.4 Hz, 1H), 5.36 – 5.19 (m, 2H), 4.83 (d, *J* = 8.4 Hz, 1H), 4.80 – 4.71 (m, 2H), 4.55 (dd, *J* = 32.2, 11.7 Hz, 2H), 4.20 – 4.06 (m, 2H), 4.00 (d, *J* = 9.9 Hz, 1H), 3.85 (ddd, *J* = 31.9, 11.8, 6.3 Hz, 4H), 3.76 (s, 3H), 3.48 – 3.41 (m, 4H), 2.12 (s, 3H), 2.06 (s, 3H). ^{13}C NMR (500 MHz, $CDCl_3$) δ 170.5, 169.8, 167.0, 165.8, 164.6, 162.2, 137.1, 133.5, 129.8, 129.2, 128.5, 128.3, 127.9, 127.9, 100.3, 99.3, 92.0, 78.9, 74.1, 72.9, 72.8, 72.2, 72.0, 71.3, 68.7, 62.3, 57.3, 56.3, 52.9, 40.3, 20.7, 20.6. HRMS (ESI): *m/z*: calcd for $C_{36}H_{38}Cl_4NO_{16}$ [M – H]: 880.0950; found: 880.0946.

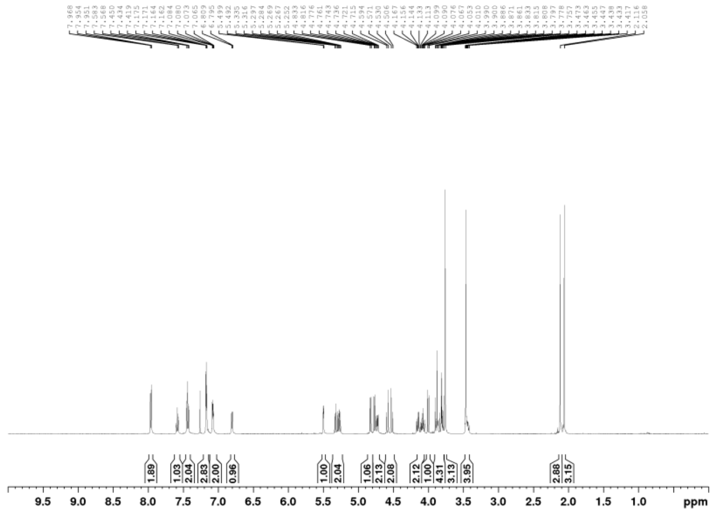

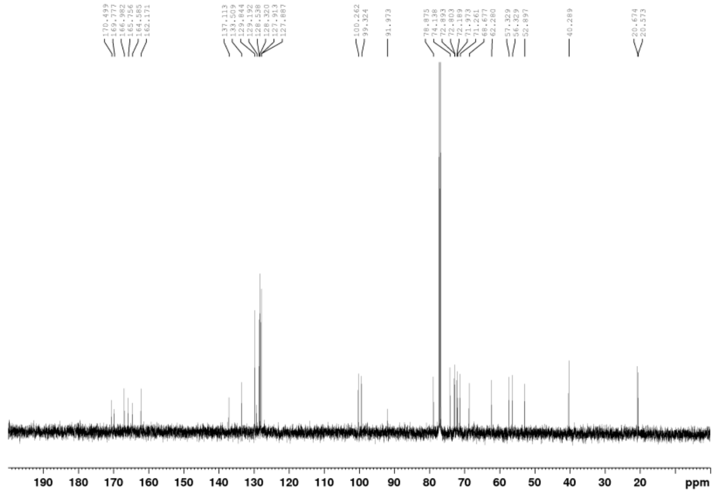

Compound 46d

^{1}H NMR (500 MHz, $CDCl_3$) δ 7.97 (d, *J* = 7.6 Hz, 2H), 7.59 (t, *J* = 7.4 Hz, 1H), 7.45 (t, *J* = 7.7 Hz, 2H), 7.19 – 7.11 (m, 3H), 7.08 (dd, *J* = 6.4, 2.5 Hz, 2H), 5.53 (d, *J* = 6.7 Hz, 1H), 5.38 (d, *J* = 3.2 Hz, 1H), 5.31 – 5.21 (m, 2H), 4.91 (d, *J* = 8.2 Hz, 1H), 4.71 (dd, *J* = 14.3, 5.5 Hz, 2H), 4.56 (s, 2H), 4.15 (dd, *J* = 11.6, 4.9 Hz, 1H), 4.01 (dd, *J* = 11.6, 7.4 Hz, 1H), 3.95 (d, *J* = 9.8 Hz, 1H), 3.84 (dd, *J* = 11.4, 6.9 Hz, 2H), 3.75 (s, 3H), 3.42 (s, 3H), 3.14 (dt, *J* = 10.4, 7.8 Hz, 1H), 2.08 (s, 3H), 2.04 (s, 3H), 1.97 (s, 3H), 1.38 (s, 3H). ^{13}C NMR (500 MHz, $CDCl_3$) δ 171.1, 170.5, 170.0, 169.2, 167.2, 164.7, 137.2, 133.5, 129.7, 128.6, 128.3, 127.8, 127.8, 100.7, 99.7, 79.0, 75.1, 73.9, 72.8, 71.3, 70.7, 68.4, 62.7, 57.0, 55.3, 52.7, 23.0, 20.7, 20.6. HRMS (ESI): *m/z*: calcd for $C_{36}H_{42}NO_{16}$ [M – H]: 744.2509; found: 744.2503.

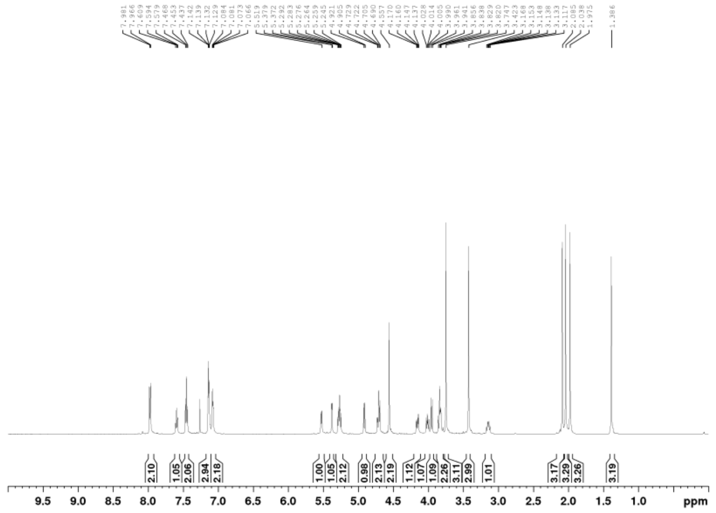

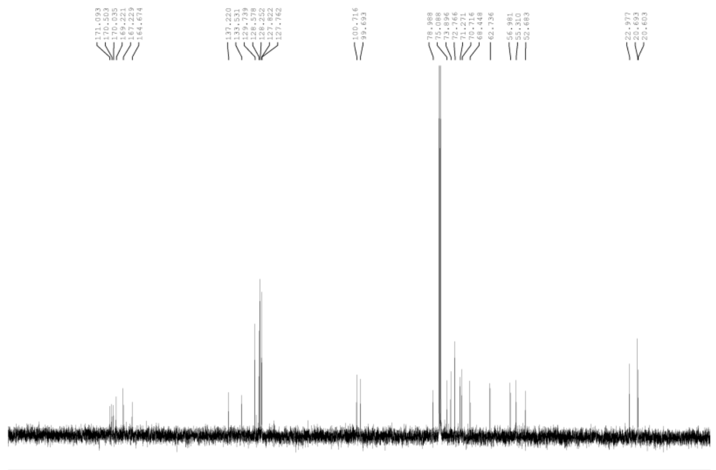

Compound 48d

^{1}H NMR (500 MHz, DMSO) δ 7.95 (d, *J* = 7.3 Hz, 2H), 7.59 (t, *J* = 7.4 Hz, 1H), 7.46 (t, *J* = 7.6 Hz, 2H), 5.22 (d, *J* = 3.2 Hz, 1H), 4.99 (d, *J* = 8.1 Hz, 1H), 4.89 – 4.78 (m, 2H), 4.58 (t, *J* = 9.4 Hz, 1H), 4.29 – 4.19 (m, 2H), 4.05 (dd, *J* = 11.4, 3.4 Hz, 1H), 3.95 – 3.87 (m, 1H), 3.80 (ddd, *J* = 11.3, 9.6, 5.7 Hz, 2H), 3.71 (d, *J* = 9.5 Hz, 1H), 3.64 (s, 3H), 3.24 (s, 3H), 1.99 (d, *J* = 2.5 Hz, 6H), 1.92 (s, 3H), 1.18 (s, 3H). ^{13}C NMR (500 MHz, DMSO) δ 170.1, 169.9, 169.2, 168.8, 167.5, 164.5, 132.7, 130.1, 130.0, 128.0, 101.6, 99.7, 77.4, 75.6, 71.8, 71.7, 70.8, 70.0, 68.4, 62.9, 55.7, 52.2, 22.2, 20.7, 20.6. HRMS (ESI): *m/z*: calcd for $C_{29}H_{36}NNa_2O_{19}S$ [M + Na]: 780.1392; found: 780.1397.

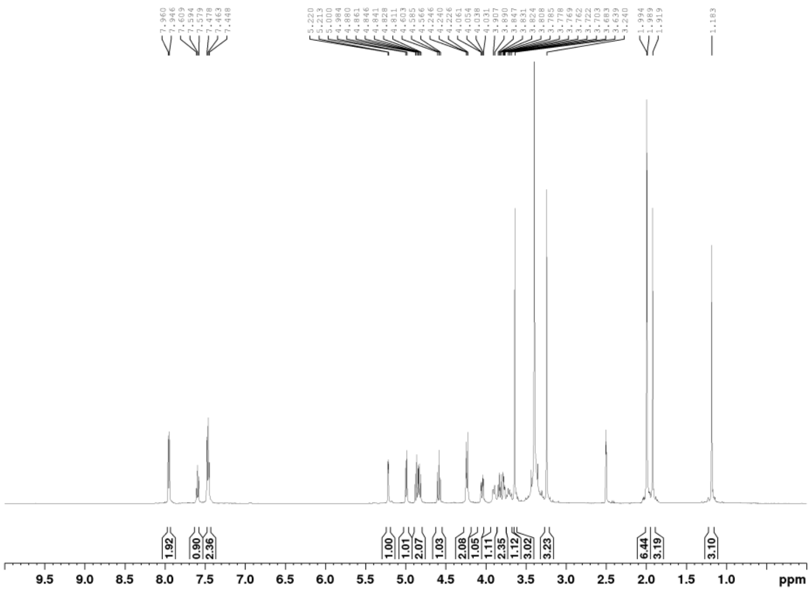

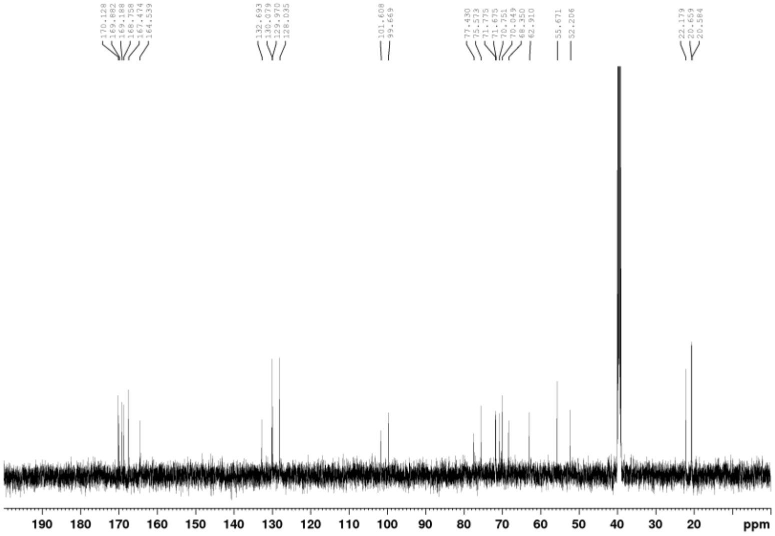

Compound 49d

^{1}H NMR (500 MHz, D_2O) δ 4.52 (d, *J* = 7.9 Hz, 1H), 4.36 (d, *J* = 8.6 Hz, 1H), 4.21 (t, *J* = 9.0 Hz, 1H), 4.11 (d, *J* = 3.0 Hz, 1H), 3.95 (dd, *J* = 10.8, 8.6 Hz, 1H), 3.78 – 3.67 (m, 5H), 3.64 – 3.57 (m, 2H), 3.46 – 3.40 (m, 4H), 1.94 (s, 3H). ^{13}C NMR (500 MHz, D_2O) δ 175.5, 174.9, 103.7, 102.2, 83.9, 80.7, 76.1, 74.9, 71.6, 70.5, 67.6, 61.1, 57.0, 50.9, 22.3. HRMS (ESI): *m/z*: calcd for $C_{15}H_{23}NNa_3O_{15}S$ [M + Na]: 558.0476; found: 558.0471.

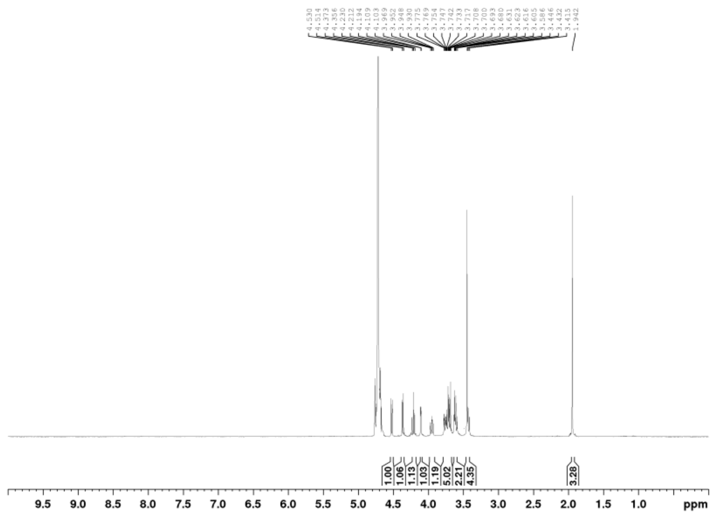

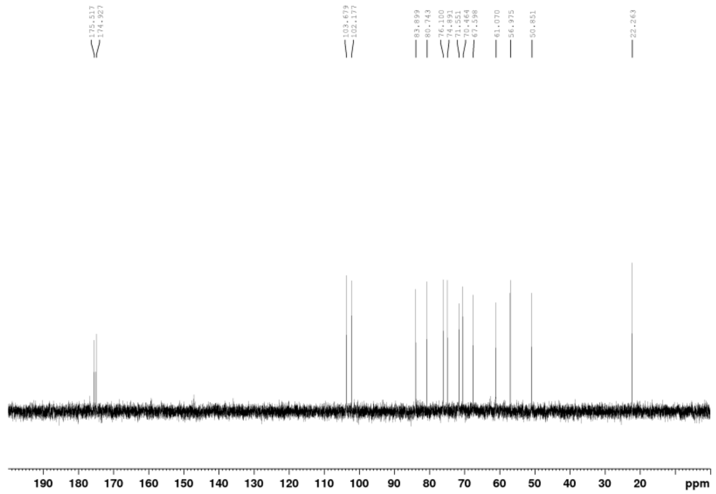

Protected dimer D4-A1

^{1}H NMR (500 MHz, $CDCl_3$) δ 7.88 (dd, *J* = 20.3, 7.6 Hz, 4H), 7.46 (dd, *J* = 29.2, 7.3 Hz, 4H), 7.39 – 7.27 (m, 12H), 6.80 (d, *J* = 6.5 Hz, 1H), 5.68 (d, *J* = 9.7 Hz, 1H), 5.49 (dd, *J* = 5.7, 4.1 Hz, 2H), 5.01 (d, *J* = 11.5 Hz, 1H), 4.95 (d, *J* = 7.9 Hz, 1H), 4.81 (d, *J* = 8.3 Hz, 2H), 4.68 (d, *J* = 11.4 Hz, 1H), 4.56 – 4.50 (m, 1H), 4.43 (dd, *J* = 34.7, 11.6 Hz, 2H), 4.25 (d, *J* = 10.0 Hz, 1H), 4.19 (d, *J* = 1.9 Hz, 1H), 4.00 – 3.91 (m, 2H), 3.74 – 3.66 (m, 4H), 3.62 – 3.53 (m, 2H), 3.43 (s, 3H). ^{13}C NMR (500 MHz, $CDCl_3$) δ 166.6, 166.1, 165.5, 164.8, 162.4, 138.5, 137.9, 133.6, 133.4, 129.9, 129.7, 128.8, 128.5, 128.4, 128.4, 128.4, 128.2, 128.1, 128.0, 127.9, 127.8, 127.7, 127.6, 101.2, 99.2, 92.0, 75.7, 75.5, 75.0, 73.5, 73.3, 71.9, 71.8, 71.4, 70.8, 68.6, 57.2, 56.5, 52.9, 40.2. HRMS (ESI): *m/z*: calcd for $C_{46}H_{45}Cl_4NNaO_{15}$ [M + Na]: 1014.1436; found: 1014.1441.

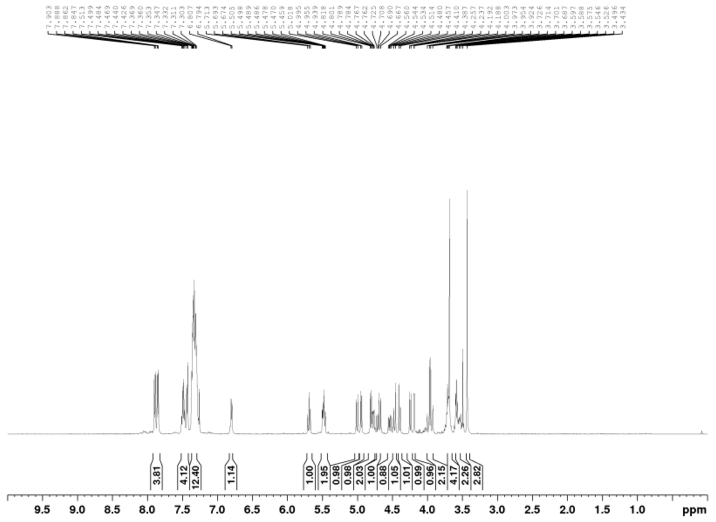

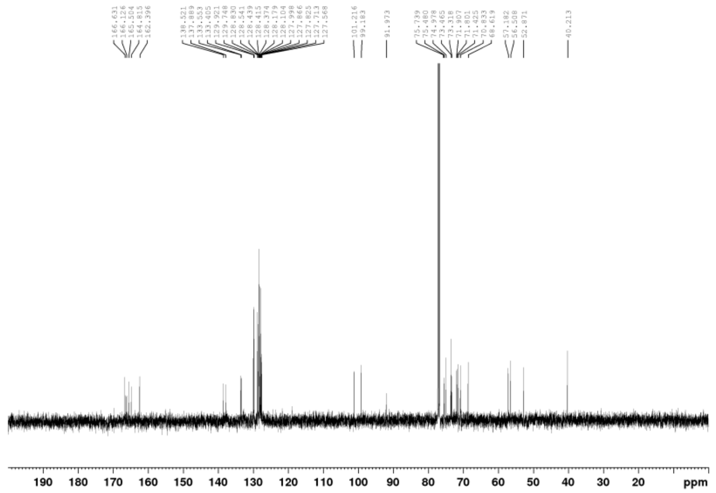

Compound 46e

^{1}H NMR (500 MHz, $CDCl_3$) δ 7.92 (dd, *J* = 24.8, 7.5 Hz, 4H), 7.55 – 7.46 (m, 2H), 7.43 – 7.25 (m, 14H), 5.72 (t, *J* = 9.7 Hz, 1H), 5.46 (dd, *J* = 13.6, 6.1 Hz, 2H), 5.28 (d, *J* = 6.5 Hz, 1H), 5.00 (d, *J* = 11.7 Hz, 1H), 4.90 (d, *J* = 7.8 Hz, 1H), 4.84 (d, *J* = 8.2 Hz, 1H), 4.78 (d, *J* = 2.7 Hz, 1H), 4.66 (d, *J* = 11.7 Hz, 1H), 4.39 (dd, *J* = 36.8, 11.7 Hz, 2H), 4.20 (d, *J* = 10.0 Hz, 1H), 4.10 (s, 1H), 3.68 (d, *J* = 5.8 Hz, 4H), 3.56 (dd, *J* = 9.5, 5.7 Hz, 1H), 3.49 (dd, *J* = 9.4, 6.8 Hz, 1H), 3.40 (s, 3H), 3.19 (dd, *J* = 7.2, 2.8 Hz, 1H), 1.96 (s, 3H), 1.47 (s, 3H). ^{13}C NMR (500 MHz, $CDCl_3$) δ 171.3, 169.4, 167.0, 165.6, 164.7, 138.8, 138.1, 133.5, 129.8, 129.7, 129.3, 128.8, 128.7, 128.6, 128.5, 128.3, 128.1, 127.8, 127.6, 127.4, 102.0, 99.6, 78.4, 75.6, 74.7, 73.4, 73.3, 72.3, 72.1, 71.8, 69.4, 69.0, 56.8, 55.7, 52.7, 23.3, 20.4. HRMS (ESI): *m/z*: calcd for $C_{46}H_{49}NNaO_{15}$ [M + Na]: 878.2994; found: 878.3002.

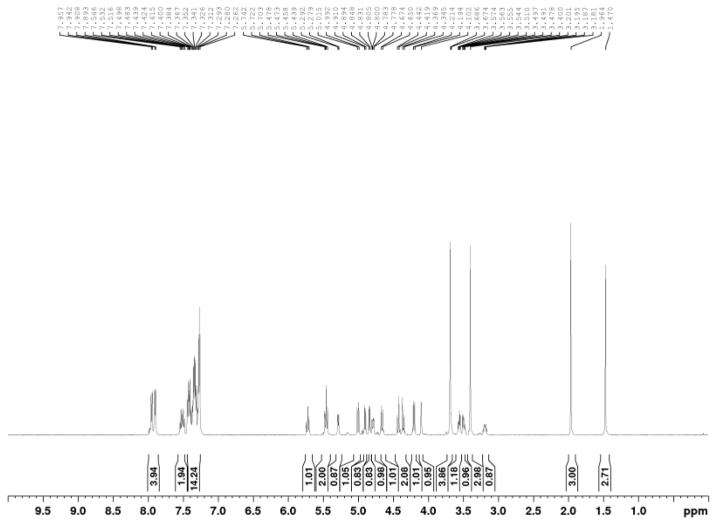

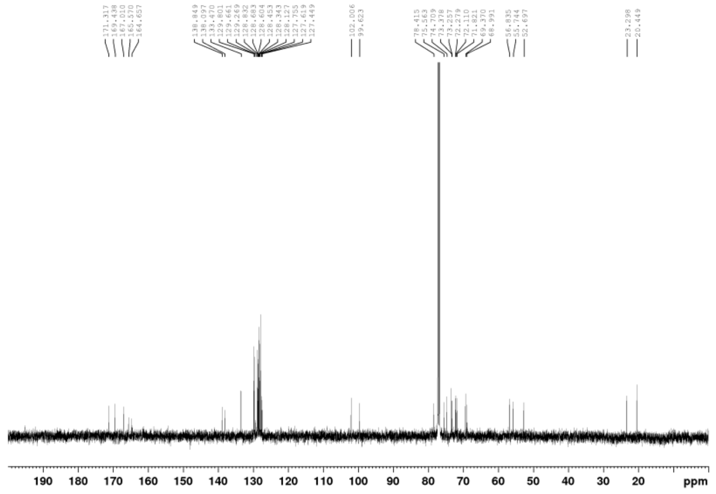

Compound 48e

^{1}H NMR (500 MHz, $CDCl_3$) δ 7.81 – 7.71 (m, 4H), 7.58 (ddd, *J* = 20.9, 13.8, 8.1 Hz, 2H), 7.49 – 7.36 (m, 4H), 5.70 (t, *J* = 9.4 Hz, 1H), 5.36 – 5.24 (m, 2H), 5.20 (d, *J* = 7.8 Hz, 1H), 4.58 – 4.50 (m, 2H), 4.16 (d, *J* = 7.6 Hz, 1H), 4.09 (dd, *J* = 12.0, 2.0 Hz, 1H), 3.90 – 3.75 (m, 3H), 3.71 (s, 3H), 3.66 (d, *J* = 8.0 Hz, 1H), 3.25 (s, 3H), 1.90 (s, 3H), 1.44 (s, 3H). ^{13}C NMR (500 MHz, DMSO) δ 169.1, 168.7, 167.8, 165.0, 164.5, 133.7, 133.2, 129.3, 129.2, 129.1, 128.7, 128.5, 128.3, 101.6, 100.6, 77.4, 74.1, 73.5, 73.2, 71.8, 71.0, 69.0, 66.9, 55.1, 52.8, 52.7, 22.5, 20.1. HRMS (ESI): *m/z*: calcd for $C_{32}H_{36}NO_{21}S_2$ [M – H]: 834.1227; found: 834.1221.

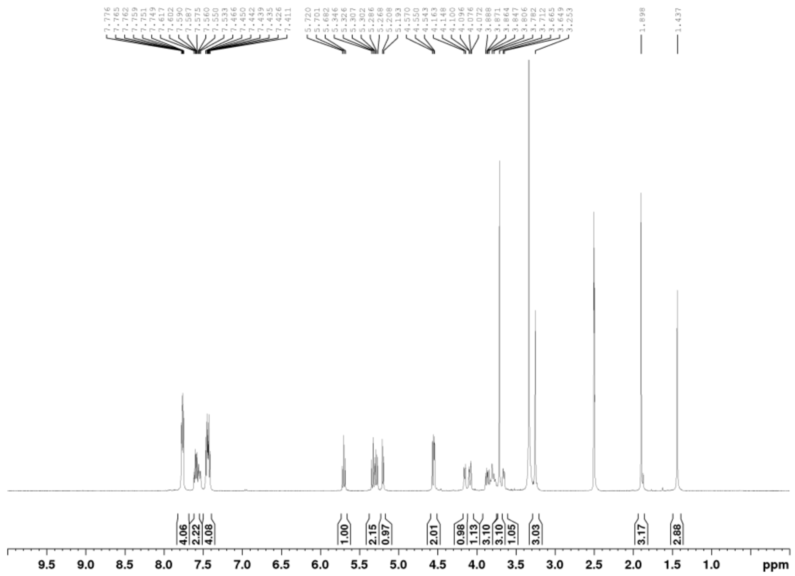

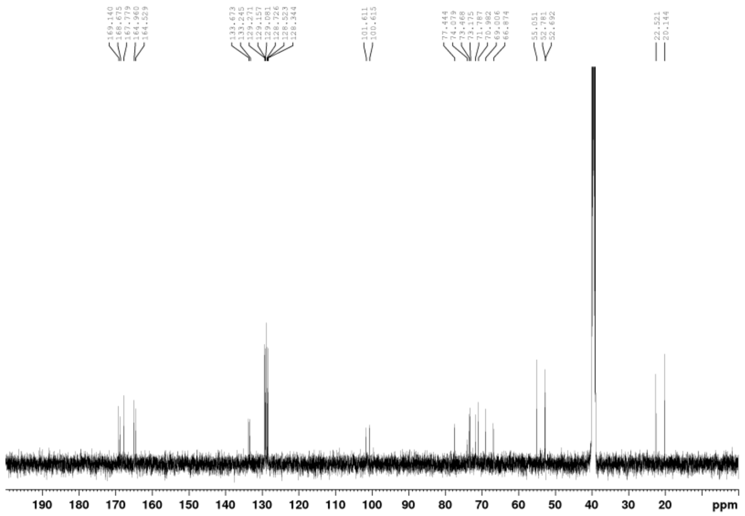

Compound 49e

^{1}H NMR (500 MHz, D_2O) δ 4.45 (dd, *J* = 7.8, 1.5 Hz, 2H), 4.29 (dd, *J* = 11.4, 3.0 Hz, 1H), 4.19 (dd, *J* = 11.4, 8.7 Hz, 1H), 4.05 (qd, *J* = 10.6, 8.1 Hz, 3H), 3.64 (d, *J* = 9.8 Hz, 1H), 3.54 – 3.48 (m, 4H), 3.43 (d, *J* = 9.0 Hz, 1H), 3.36 – 3.30 (m, 1H), 2.00 (s, 3H). ^{13}C NMR (500 MHz, D_2O) δ 175.9, 174.9, 103.3, 102.0, 76.4, 76.3, 75.2, 74.7, 72.5, 72.4, 71.9, 68.0, 57.3, 51.6, 22.3. HRMS (ESI): *m/z*: calcd for $C_{15}H_{22}NNa_4O_{18}S_2$ [M + Na]: 659.9864; found: 659.9873.

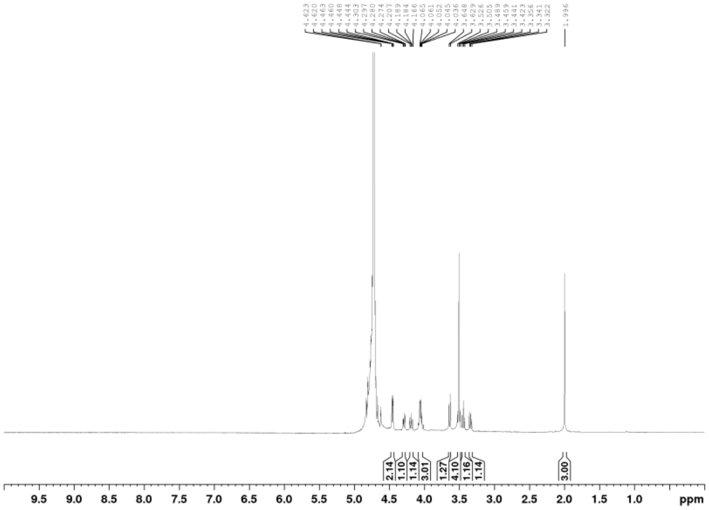

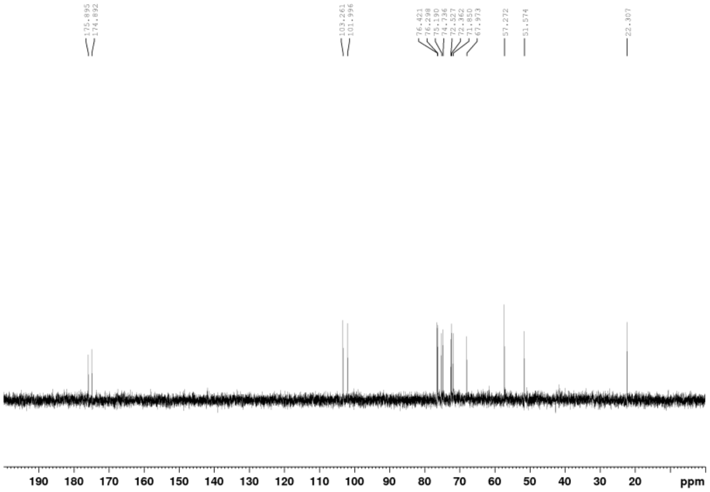

Protected dimer D4-A2

^{1}H NMR (500 MHz, $CDCl_3$) δ 7.86 (dd, *J* = 15.0, 7.6 Hz, 4H), 7.49 (dd, *J* = 14.1, 7.3 Hz, 2H), 7.38 – 7.27 (m, 9H), 6.76 (d, *J* = 6.7 Hz, 1H), 5.66 – 5.56 (m, 2H), 5.52 (t, *J* = 9.6 Hz, 1H), 5.40 (dd, *J* = 9.4, 7.7 Hz, 1H), 4.88 (dd, *J* = 24.4, 8.0 Hz, 2H), 4.78 (dd, *J* = 10.9, 3.3 Hz, 1H), 4.52 (dd, *J* = 26.6, 11.7 Hz, 2H), 4.20 (d, *J* = 9.9 Hz, 1H), 3.94 (q, *J* = 14.9 Hz, 2H), 3.86 (t, *J* = 5.9 Hz, 1H), 3.75 (s, 3H), 3.56 (d, *J* = 5.9 Hz, 2H), 3.49 (s, 3H), 3.46 – 3.38 (m, 1H), 2.13 (s, 3H). ^{13}C NMR (500 MHz, $CDCl_3$) δ 169.9, 166.5, 165.9, 165.5, 164.7, 162.2, 137.7, 133.5, 133.5, 129.8, 129.8, 128.7, 128.4, 128.4, 127.8, 127.7, 100.4, 99.3, 91.9, 73.6, 73.4, 72.8, 72.1, 72.0, 71.5, 70.7, 69.2, 68.7, 57.4, 56.5, 53.0, 40.2, 20.7. HRMS (ESI): *m/z*: calcd for $C_{41}H_{41}Cl_4NNaO_{16}$ [M + Na]: 966.1072; found: 966.1068.

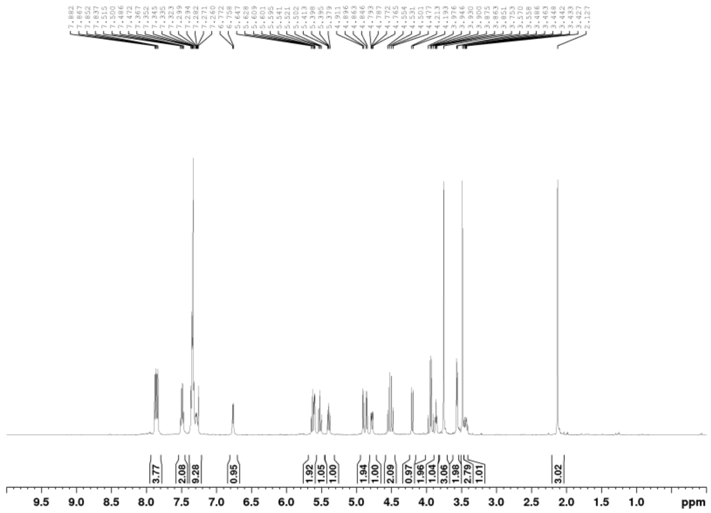

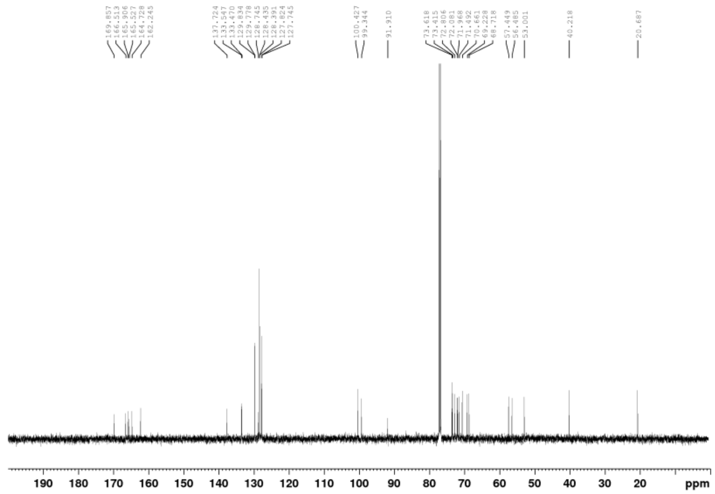

Compound 46f

^{1}H NMR (500 MHz, $CDCl_3$) δ 7.90 (dd, *J* = 24.9, 7.5 Hz, 4H), 7.59 – 7.47 (m, 2H), 7.40 – 7.22 (m, 9H), 5.64 (t, *J* = 9.6 Hz, 1H), 5.53 – 5.34 (m, 4H), 4.93 (d, *J* = 8.2 Hz, 1H), 4.85 (d, *J* = 7.6 Hz, 1H), 4.78 (dd, *J* = 10.8, 3.3 Hz, 1H), 4.51 (q, *J* = 11.7 Hz, 2H), 4.15 (d, *J* = 9.9 Hz, 1H), 3.84 (t, *J* = 5.8 Hz, 1H), 3.76 (s, 3H), 3.55 (ddd, *J* = 17.0, 10.2, 5.9 Hz, 2H), 3.45 (s, 3H), 3.13 (dt, *J* = 10.5, 7.8 Hz, 1H), 2.10 (s, 3H), 1.93 (s, 3H), 1.43 (s, 3H). ^{13}C NMR (500 MHz, $CDCl_3$) δ 171.1, 170.0, 169.2, 166.8, 165.5, 164.7, 137.9, 133.5, 133.4, 129.8, 129.7, 129.0, 128.6, 128.6, 128.4, 128.4, 127.7, 127.6, 100.9, 99.7, 75.3, 73.5, 72.9, 72.6, 72.3, 71.6, 69.2, 57.1, 55.6, 52.8, 34.0, 23.0, 20.8, 20.4. HRMS (ESI): *m/z*: calcd for $C_{41}H_{45}NNaO_{16}$ [M + Na]: 830.2631; found: 830.2640.

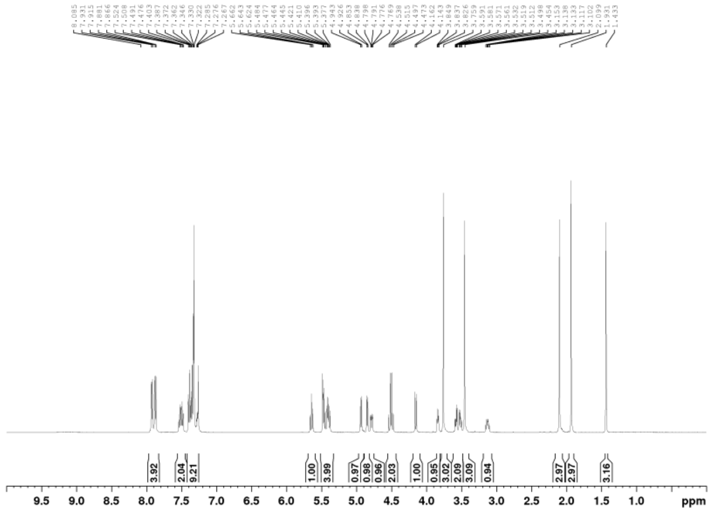

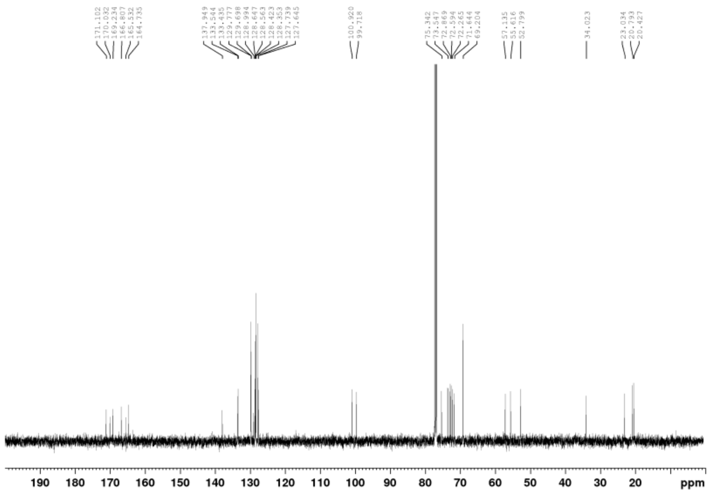

Compound 48f

^{1}H NMR (500 MHz, DMSO) δ 7.78 (d, *J* = 8.1 Hz, 4H), 7.63 – 7.55 (m, 2H), 7.45 (dd, *J* = 16.3, 8.0 Hz, 4H), 5.77 – 5.68 (m, 1H), 5.31 – 5.22 (m, 3H), 4.58 – 4.49 (m, 2H), 4.29 (dd, *J* = 12.1, 2.9 Hz, 1H), 4.22 – 4.15 (m, 2H), 3.85 (m, 3H), 3.70 (s, 3H), 3.11 (s, 3H), 1.98 (s, 3H), 1.90 (s, 3H), 1.59 (s, 3H). ^{13}C NMR (500 MHz, DMSO) δ 170.0, 169.1, 168.8, 167.4, 165.0, 164.5, 133.7, 133.2, 129.3, 129.2, 129.1, 128.7, 128.5, 128.3, 101.2, 99.5, 76.0, 73.0, 72.6, 72.2, 71.8, 71.1, 69.1, 64.6, 54.9, 52.6, 50.1, 22.6, 20.7, 20.2. HRMS (ESI): *m/z*: calcd for $C_{34}H_{38}NO_{19}S$ [M – Na]: 796.1764; found: 796.1760.

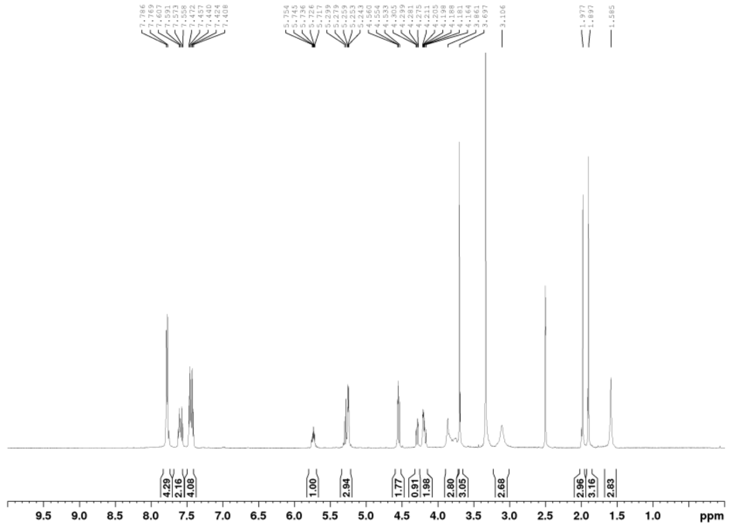

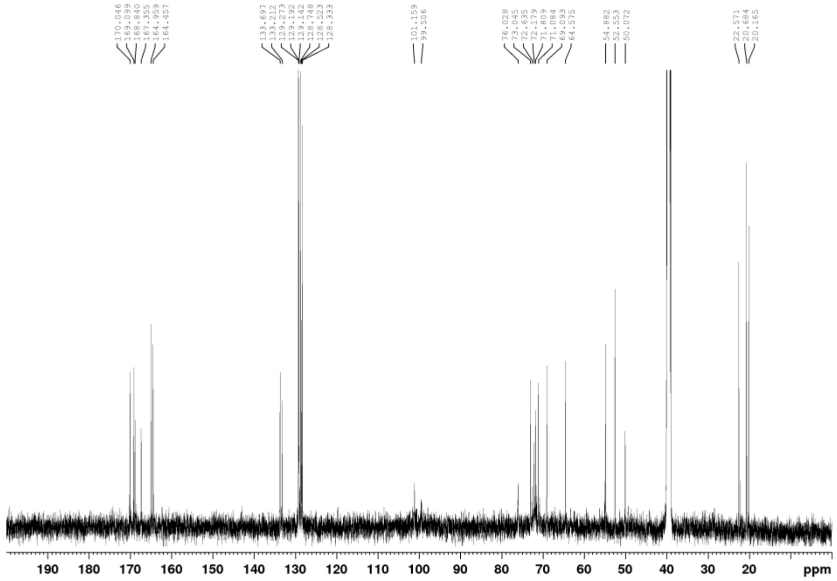

Compound 49f

^{1}H NMR (500 MHz, D_2O) δ 4.46 (d, J = 7.9 Hz, 1H), 4.40 (d, J = 8.6 Hz, 1H), 4.22 – 4.13 (m, 3H), 3.99 (dd, J = 10.8, 8.7 Hz, 1H), 3.89 (dd, J = 7.6, 4.4 Hz, 1H), 3.81 (dd, J = 10.9, 3.2 Hz, 1H), 3.68 – 3.63 (m, 1H), 3.47 (d, J = 3.7 Hz, 3H), 3.46 – 3.41 (m, 2H), 3.32 – 3.25 (m, 1H), 1.97 (s, 3H). ^{13}C NMR (500 MHz, D_2O) δ 175.95, 174.91, 104.09, 102.13, 80.08, 76.16, 75.40, 72.75, 72.68, 71.82, 67.70, 67.63, 57.10, 50.95, 22.27. HRMS (ESI): *m/z*: calcd for $C_{15}H_{23}NNa_3O_{15}S$ [M + Na]: 558.0476; found: 558.0467.

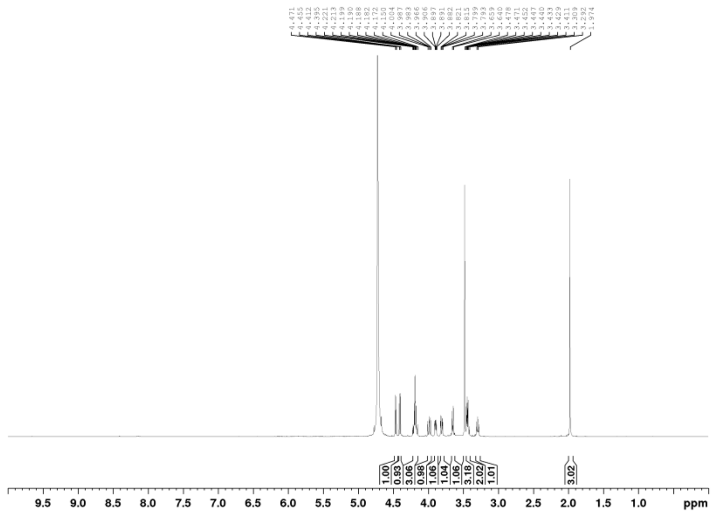

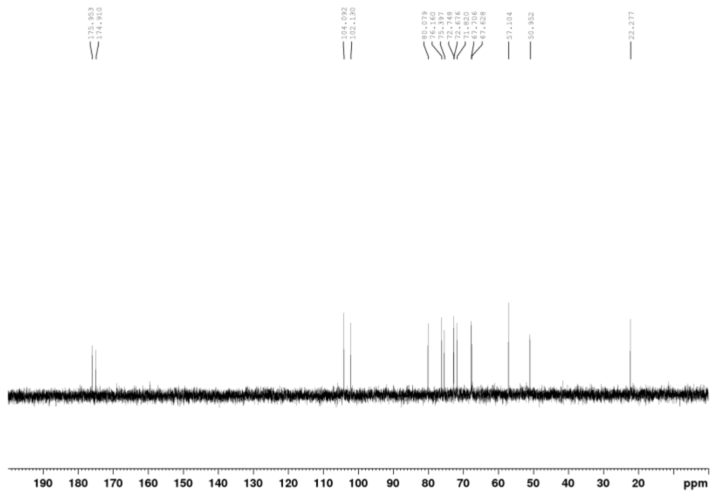

Protected dimer D4-A3

^{1}H NMR (500 MHz, $CDCl_3$) δ 7.87 (dd, *J* = 20.2, 7.3 Hz, 4H), 7.51 – 7.44 (m, 4H), 7.39 – 7.30 (m, 7H), 6.84 (d, *J* = 6.5 Hz, 1H), 5.71 (t, *J* = 9.6 Hz, 1H), 5.55 – 5.44 (m, 2H), 5.03 (d, *J* = 11.4 Hz, 1H), 4.95 (d, *J* = 7.8 Hz, 1H), 4.82 – 4.76 (m, 2H), 4.73 (d, *J* = 11.4 Hz, 1H), 4.28 (d, *J* = 9.9 Hz, 1H), 4.22 (dd, *J* = 11.1, 6.6 Hz, 1H), 4.11 (d, *J* = 2.6 Hz, 1H), 4.04 (dd, *J* = 11.2, 6.3 Hz, 1H), 3.96 (q, *J* = 14.9 Hz, 2H), 3.80 (s, 3H), 3.71 (t, *J* = 6.4 Hz, 1H), 3.59 – 3.50 (m, 1H), 3.43 (s, 3H), 2.00 (s, 3H). ^{13}C NMR (500 MHz, $CDCl_3$) δ 170.5, 166.6, 166.2, 165.5, 164.8, 162.5, 138.1, 133.6, 133.5, 129.9, 129.8, 129.0, 128.7, 128.5, 128.4, 128.3, 128.3, 127.7, 101.2, 99.1, 91.9, 76.6, 74.9, 74.9, 71.9, 71.8, 71.4, 70.9, 62.6, 57.2, 56.3, 53.0, 40.2, 20.7. HRMS (ESI): *m/z*: calcd for $C_{41}H_{41}Cl_4NNaO_{16}$ [M + Na]: 966.1072; found: 966.1068.

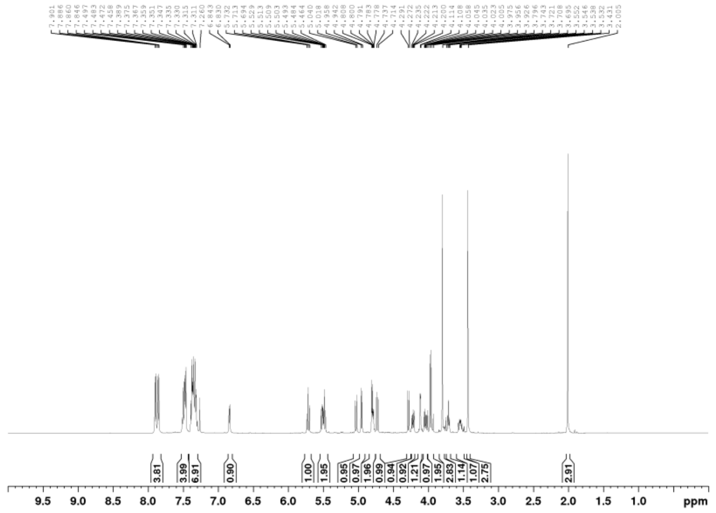

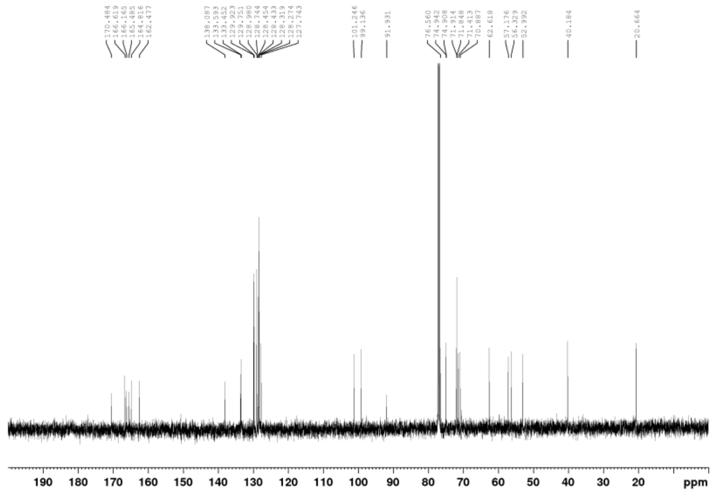

Compound 46g

^{1}H NMR (500 MHz, $CDCl_3$) δ 7.93 (dd, *J* = 23.4, 7.5 Hz, 4H), 7.57 – 7.26 (m, 11H), 5.74 (t, *J* = 9.7 Hz, 1H), 5.52 – 5.39 (m, 2H), 5.30 (d, *J* = 6.6 Hz, 1H), 5.01 (d, *J* = 11.6 Hz, 1H), 4.90 (d, *J* = 7.9 Hz, 1H), 4.81 (dd, *J* = 13.0, 5.2 Hz, 2H), 4.71 (d, *J* = 11.6 Hz, 1H), 4.23 (d, *J* = 10.0 Hz, 1H), 4.16 (dd, *J* = 11.1, 6.7 Hz, 1H), 4.02 (d, *J* = 1.6 Hz, 1H), 3.97 (dd, *J* = 11.2, 6.1 Hz, 1H), 3.78 (s, 3H), 3.67 (t, *J* = 6.3 Hz, 1H), 3.40 (s, 3H), 3.19 (dt, *J* = 10.7, 7.8 Hz, 1H), 1.96 (d, *J* = 3.1 Hz, 6H), 1.49 (s, 3H). ^{13}C NMR (500 MHz, $CDCl_3$) δ 171.4, 170.5, 169.4, 167.0, 165.6, 164.6, 138.4, 133.5, 129.8, 129.6, 129.2, 129.0, 128.6, 128.5, 128.2, 127.6, 102.0, 99.6, 78.2, 74.9, 74.6, 72.3, 72.0, 71.8, 70.6, 69.4, 62.9, 56.8, 55.6, 52.8, 23.3, 20.7, 20.4. HRMS (ESI): *m/z*: calcd for $C_{41}H_{45}NNaO_{16}$ [M + Na]: 830.2631; found: 830.2626.

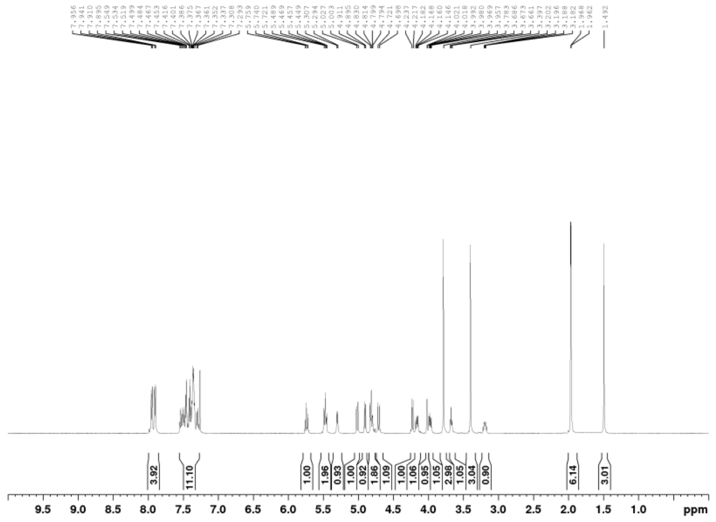

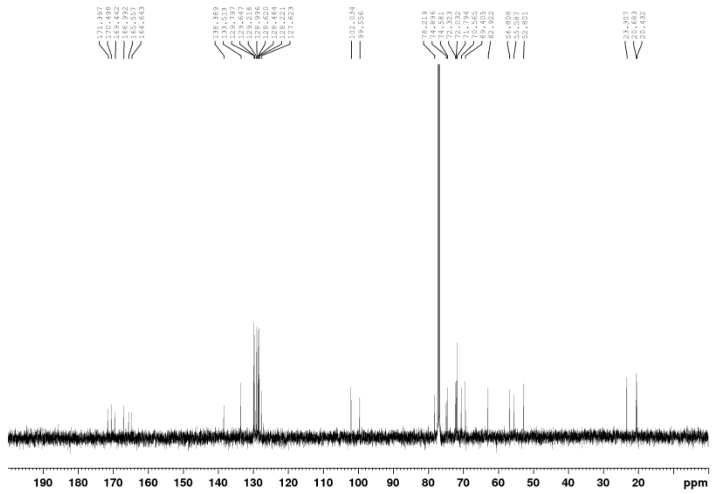

Compound 48g

^{1}H NMR (500 MHz, DMSO) δ 7.78 (d, *J* = 6.9 Hz, 4H), 7.66 – 7.53 (m, 2H), 7.44 (dt, *J* = 15.7, 7.7 Hz, 4H), 5.73 (t, *J* = 8.9 Hz, 1H), 5.34 – 5.18 (m, 3H), 4.61 – 4.48 (m, 2H), 4.29 (dd, *J* = 12.0, 2.7 Hz, 1H), 4.23 – 4.09 (m, 2H), 3.85 (dd, *J* = 24.3, 7.7 Hz, 3H), 3.70 (s, 3H), 3.11 (s, 3H), 1.98 (s, 3H), 1.90 (s, 3H), 1.57 (s, 3H). ^{13}C NMR (500 MHz, DMSO) δ 170.0, 169.1, 168.8, 167.5, 164.9, 164.5, 133.7, 133.2, 129.3, 129.2, 129.1, 128.7, 128.5, 128.3, 101.1, 99.6, 76.2, 73.0, 72.2, 71.7, 71.1, 69.1, 64.5, 54.9, 52.6, 50.1, 22.5, 20.7, 20.1. HRMS (ESI): *m/z*: calcd for $C_{34}H_{38}NO_{19}S$ [M – H]: 796.1764; found: 796.1771.

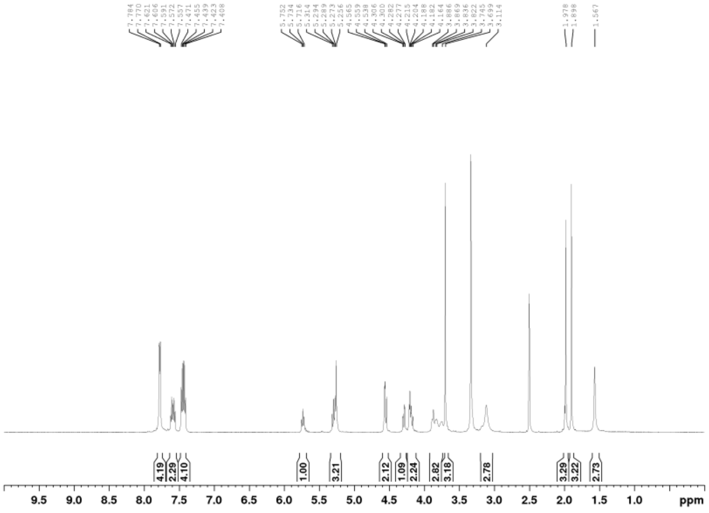

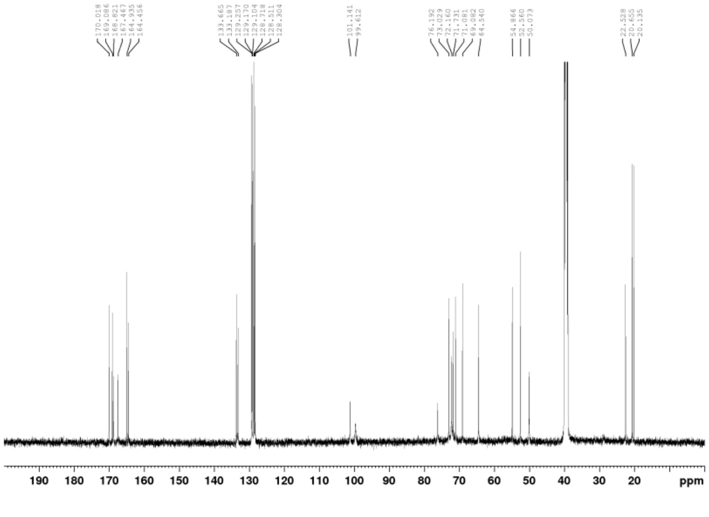

Compound 49g

^{1}H NMR (500 MHz, D_2O) δ 4.43 (dd, J = 7.7, 3.7 Hz, 2H), 4.05 – 3.96 (m, 2H), 3.83 – 3.71 (m, 3H), 3.62 (d, J = 9.6 Hz, 1H), 3.54 – 3.46 (m, 4H), 3.42 (t, J = 9.1 Hz, 1H), 3.36 – 3.29 (m, 1H), 1.98 (s, 3H). ^{13}C NMR (500 MHz, D_2O) δ 175.9, 174.9, 103.3, 102.1, 76.5, 76.4, 75.2, 74.9, 74.5, 72.5, 71.8, 61.1, 57.2, 51.7, 22.3. HRMS (ESI): m/z: calcd for $C_{15}H_{23}NNa_3O_{15}S$ [M + Na]: 558.0476; found: 558.0483.

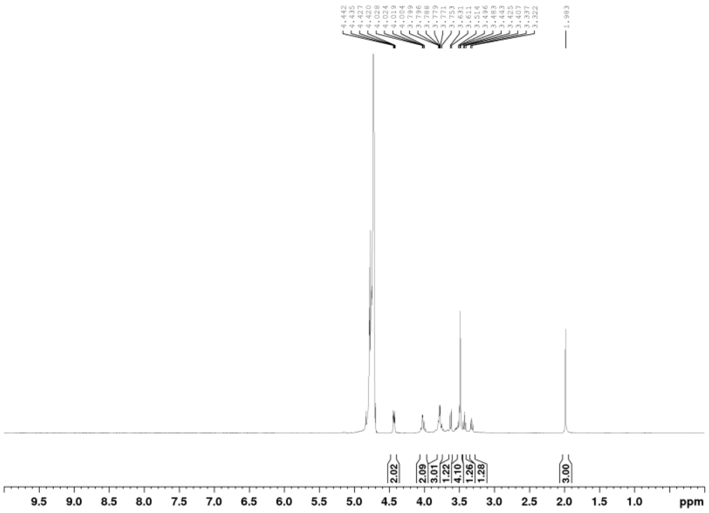

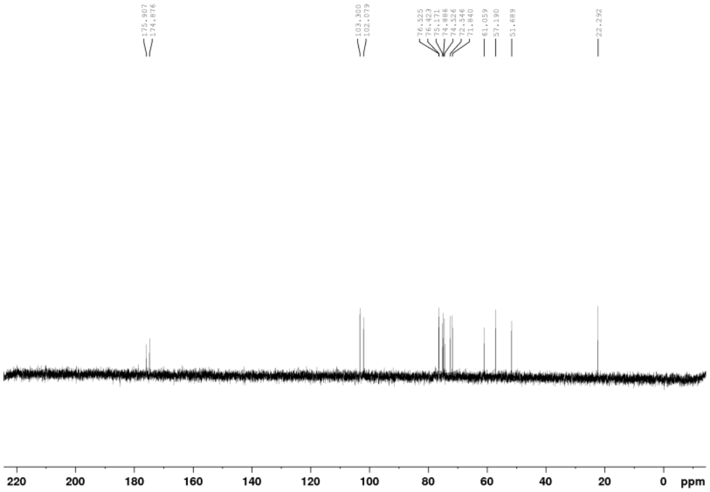

Protected dimer D4-A4

^{1}H NMR (500 MHz, $CDCl_3$) δ 7.86 (dd, *J* = 15.8, 7.6 Hz, 4H), 7.49 (q, *J* = 7.3 Hz, 2H), 7.34 (q, *J* = 7.8 Hz, 4H), 6.77 (d, *J* = 6.7 Hz, 1H), 5.63 (t, *J* = 9.5 Hz, 1H), 5.56 – 5.46 (m, 2H), 5.39 (dd, *J* = 9.4, 7.7 Hz, 1H), 4.88 (dd, *J* = 22.1, 7.9 Hz, 2H), 4.81 (dd, *J* = 10.9, 3.4 Hz, 1H), 4.22 (d, *J* = 10.0 Hz, 1H), 4.13 (ddd, *J* = 18.5, 11.5, 6.4 Hz, 2H), 3.92 (dd, *J* = 15.9, 6.7 Hz, 3H), 3.81 (s, 3H), 3.48 (s, 3H), 3.43 (dd, *J* = 7.3, 2.8 Hz, 1H), 2.15 (s, 3H), 2.07 (s, 3H). ^{13}C NMR (500 MHz, $CDCl_3$) δ 170.5, 169.8, 166.5, 165.9, 165.5, 164.7, 162.3, 133.6, 133.5, 129.8, 129.8, 128.7, 128.4, 128.4, 100.5, 99.3, 91.9, 73.1, 72.1, 71.9, 71.5, 71.2, 70.7, 68.8, 62.2, 57.4, 56.4, 53.0, 40.2, 20.7, 20.6. HRMS (ESI): *m/z*: calcd for $C_{36}H_{37}Cl_4NNaO_{17}$ [M + Na]: 918.0708; found: 918.0701.

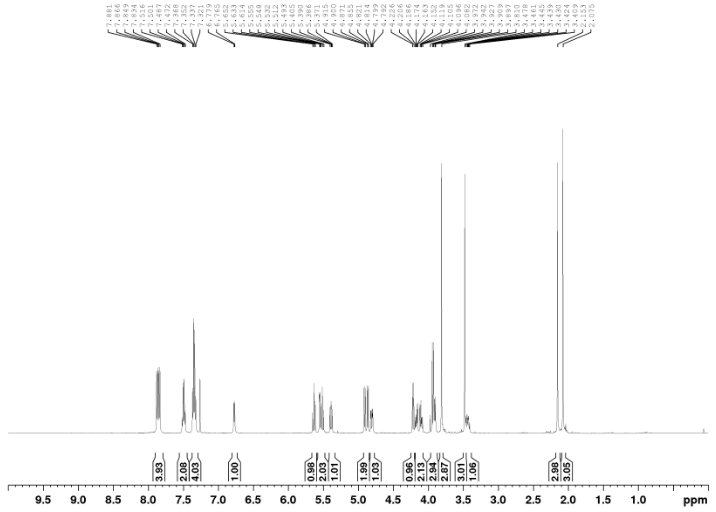

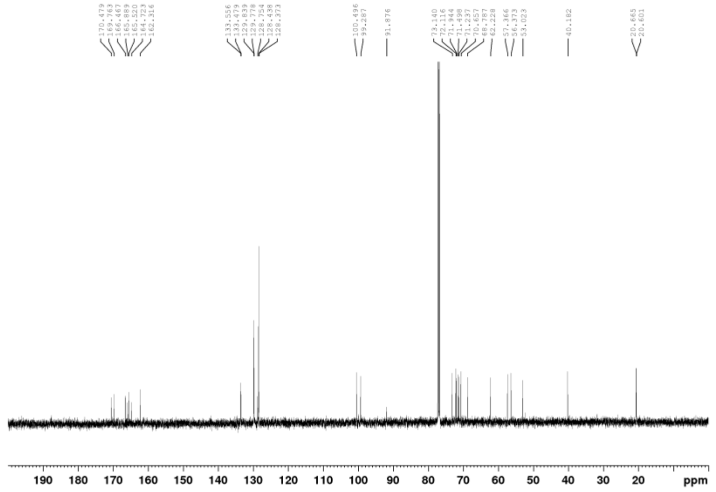

Compound 46h

^{1}H NMR (500 MHz, $CDCl_3$) δ 7.99 – 7.80 (m, 4H), 7.57 – 7.42 (m, 2H), 7.40 – 7.29 (m, 4H), 5.65 (t, *J* = 9.6 Hz, 1H), 5.50 – 5.34 (m, 4H), 4.93 (d, *J* = 8.3 Hz, 1H), 4.85 (d, *J* = 7.6 Hz, 1H), 4.80 (dd, *J* = 10.8, 3.4 Hz, 1H), 4.20 – 4.12 (m, 2H), 4.04 (dd, *J* = 11.6, 7.3 Hz, 1H), 3.89 – 3.84 (m, 1H), 3.79 (s, 3H), 3.44 (s, 3H), 3.12 (dt, *J* = 10.7, 7.8 Hz, 1H), 2.12 (s, 3H), 2.05 (s, 3H), 1.93 (s, 3H), 1.44 (s, 3H). ^{13}C NMR (500 MHz, $CDCl_3$) δ 171.2, 170.5, 169.9, 169.2, 166.8, 165.5, 164.7, 133.5, 133.4, 129.8, 129.7, 129.0, 128.6, 128.6, 128.4, 101.0, 99.7, 75.1, 72.6, 72.2, 71.6, 71.2, 69.2, 68.7, 62.6, 57.0, 55.5, 52.8, 23.0, 20.7, 20.4. HRMS (ESI): *m/z*: calcd for $C_{36}H_{41}NNaO_{17}$ [M + Na]: 782.2267; found: 782.2271.

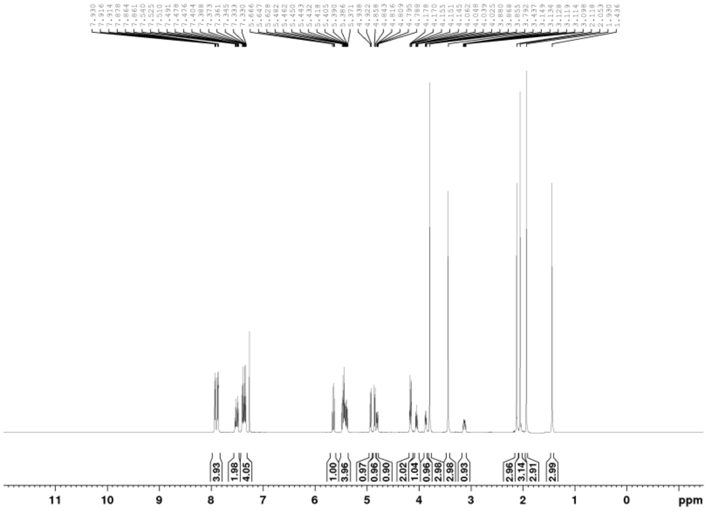

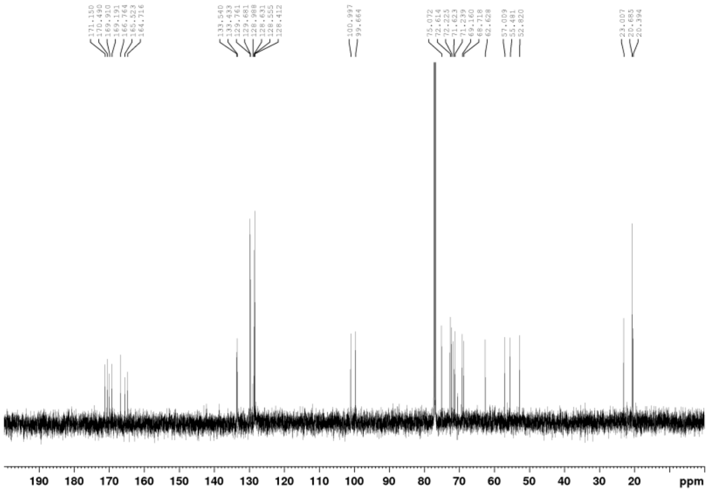

Compound 49h

^{1}H NMR (500 MHz, D_2O) δ 4.43 (d, *J* = 7.9 Hz, 1H), 4.36 (d, *J* = 8.5 Hz, 1H), 4.11 (d, *J* = 3.0 Hz, 1H), 3.95 (dd, *J* = 10.8, 8.7 Hz, 1H), 3.78 – 3.70 (m, 3H), 3.62 (dd, *J* = 8.3, 4.1 Hz, 2H), 3.45 (s, 3H), 3.41 (dd, *J* = 5.7, 3.3 Hz, 2H), 3.29 – 3.22 (m, 1H), 1.95 (s, 3H). ^{13}C NMR (500 MHz, D_2O) δ 175.9, 174.9, 104.1, 102.2, 80.3, 76.2, 75.3, 74.9, 72.7, 71.8, 67.8, 61.1, 57.0, 51.0, 22.2. HRMS (ESI): *m/z*: calcd for $C_{15}H_{24}NNa_2O_{12}$ [M + Na]: 456.1088; found: 456.1083.

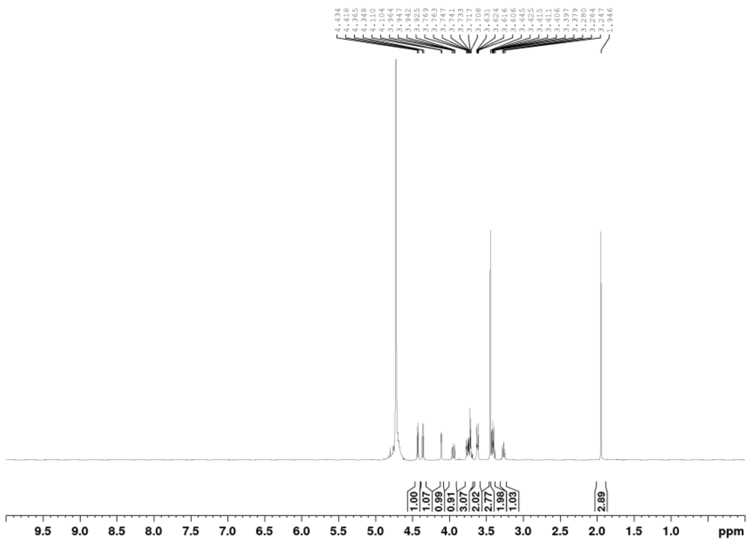

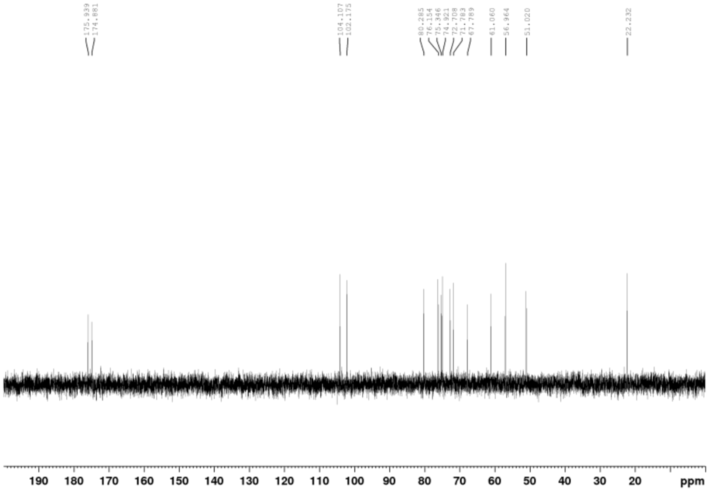

Supplement: Supplementary Information [file srep14355-s1.pdf]
